# Glycomimetics for the inhibition and modulation of lectins

**DOI:** 10.1039/d2cs00954d

**Published:** 2023-05-26

**Authors:** Steffen Leusmann, Petra Ménová, Elena Shanin, Alexander Titz, Christoph Rademacher

**Affiliations:** a Chemical Biology of Carbohydrates (CBCH), Helmholtz-Institute for Pharmaceutical Research Saarland (HIPS), Helmholtz Centre for Infection Research 66123 Saarbrücken Germany alexander.titz@helmholtz-hzi.de; b Department of Chemistry, Saarland University 66123 Saarbrücken Germany; c Deutsches Zentrum für Infektionsforschung (DZIF) Standort Hannover-Braunschweig Germany; d University of Chemistry and Technology, Prague, Technická 5 16628 Prague 6 Czech Republic; e Department of Pharmaceutical Sciences, University of Vienna, Josef-Holaubek-Platz 2 1090 Vienna Austria christoph.rademacher@univie.ac.at; f Department of Microbiology, Immunobiology and Genetics, Max F. Perutz Laboratories, University of Vienna, Biocenter 5 1030 Vienna Austria

## Abstract

Carbohydrates are essential mediators of many processes in health and disease. They regulate self-/non-self- discrimination, are key elements of cellular communication, cancer, infection and inflammation, and determine protein folding, function and life-times. Moreover, they are integral to the cellular envelope for microorganisms and participate in biofilm formation. These diverse functions of carbohydrates are mediated by carbohydrate-binding proteins, lectins, and the more the knowledge about the biology of these proteins is advancing, the more interfering with carbohydrate recognition becomes a viable option for the development of novel therapeutics. In this respect, small molecules mimicking this recognition process become more and more available either as tools for fostering our basic understanding of glycobiology or as therapeutics. In this review, we outline the general design principles of glycomimetic inhibitors (Section 2). This section is then followed by highlighting three approaches to interfere with lectin function, *i.e.* with carbohydrate-derived glycomimetics (Section 3.1), novel glycomimetic scaffolds (Section 3.2) and allosteric modulators (Section 3.3). We summarize recent advances in design and application of glycomimetics for various classes of lectins of mammalian, viral and bacterial origin. Besides highlighting design principles in general, we showcase defined cases in which glycomimetics have been advanced to clinical trials or marketed. Additionally, emerging applications of glycomimetics for targeted protein degradation and targeted delivery purposes are reviewed in Section 4.

## Abstract 2

Carbohydrates are essential mediators of many processes in health and disease. The development of glycomimetics as inhibitors and modulators of their lectin receptors has been a success story with numerous molecules reaching clinical stages.

## Graphical abstract

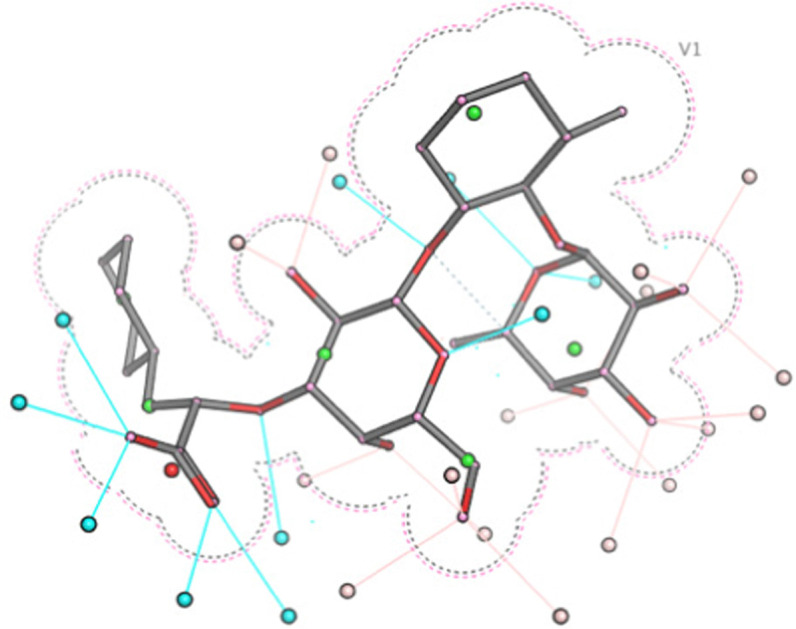

## 1. Introduction

Carbohydrates are a class of natural products with wide-ranging functions in nature. As a central energy source they empower life and serve as an integral constituent of bacterial, fungal & plant cell walls and of exoskeletons in insects and crustaceans. Additionally, recognition of carbohydrates plays an important role in a diverse set of intra- and intercellular processes in health and disease.^1^

The main characteristic of carbohydrates, especially oligosaccharides, is their three-dimensional complexity. Monomers vary in ring size and stereochemistry and the synthesis of oligosaccharides leads to linear or branched products as the glycosidic linkage can occur at one or multiple hydroxy groups of a monomer. The coding spatial information of carbohydrates is further complicated by two possible isomers at the glycosidic linkage (anomers) and possible additional modifications such as sulfation, methylation or acetylation. Cumulatively, a staggering structural diversity can be achieved despite only a limited number of monomers being employed.^2^

All living cells are decorated with a matrix of glycoproteins and glycolipids collectively referred to as the ‘glycocalyx‘, that differs between tissue and cell types in multicellular organisms.^3,4^ Glycoproteins and glycolipids are conjugation products of carbohydrates and proteins or lipids, which can be further divided based on their linkage. *N*- and *O*-glycoproteins are most common, in which the carbohydrate is linked to the asparagine side chain and hydroxy groups of serine/threonine, respectively. On the other hand, phosphate-linked glycans or *C*-mannosides belong to the rare glycosylation types.^5,6^ As a further form of glycosylation glycosylphosphatidylinositol (GPI) anchors are used for extracellular presentation of proteins.^7,8^ A GPI anchor consists of an inositol phospholipid linked to a glucosamine, followed by a trisaccharide and an ethanolamine phosphate, to which the C-terminus of the protein is bound *via* an amide bond. After transport, the modified protein is presented extracellularly, being anchored to the cell *via* the phospholipid of the GPI. Apart from the role of carbohydrates as recognition motif, glycosylation of macromolecules is involved in protein folding, stability and activity regulation.^9,10^

As glycosylation, and therefore composition of the glycocalyx, is a complex, non-templated process sensitive to changes in the metabolic and biosynthetic microenvironment of cells, diseases are frequently associated with an altered glycocalyx.^11^ While the resulting aberration may aggravate or contribute to the progression of the disease, it also presents an opportunity for diagnosis and therapy at the same time. A prominent example is cancer, in which abnormal expression of glycans, especially *O*-glycans, serves as prognostic marker and plays a role in tumour growth and metastasis.^12–14^

The recognition of glycans by carbohydrate-binding proteins, the so-called lectins, is a key mechanism in biology. Due to the abundance of lectins, different classification systems based on ligand specificity, protein structure and localisation have been established.^15^ In mammals, *inter alia* calcium-dependent C-type lectins (CTLs), calcium-independent I-type lectins, named based on their homology to the immunoglobulin superfamily, and galectins represent lectin families with significant importance in the context of disease and therapy.

A vital intercellular system that strongly relies on lectin-mediated processes is the immune system. Of significant importance in the innate immune response and early stages of an adaptive immune response is the CTL DC-SIGN (dendritic cell-specific intercellular adhesion molecule-3-grabbing non-integrin, CD209).^16^ DC-SIGN is involved in antigen uptake and presentation in dendritic cells (DCs), as well as regulation of toll-like receptors in a subpopulation of macrophages.^17^ Another calcium-dependent lectin with similar function of pathogen recognition and antigen presentation is langerin, found in a subset of epidermal DCs in skin Langerhans cells.^18^ Once an immune response has been triggered, another class of CTLs, the selectins (CD62-E, CD62-L, CD62-P) assist migration of immune cells to the site of infection^19^ and they are also crucial for inflammatory processes and cancer metastasis.^20^ Furthermore, the CTLs langerin and the asialoglycoprotein receptor (ASGPR), an uptake receptor for glycoproteins expressed on hepatocytes, have attracted growing interest as targets for drug delivery in recent years.^21^

Of outstanding interest among the I-type lectins is the family of Siglecs (sialic acid-binding immunoglobulin-type lectins), which mediate regulation of the immune response. Consequently, Siglec-1 (CD169), Siglec-2 (CD22), Siglec-3 (CD33), Siglec-4 (myelin associated glycoprotein/MAG), Siglec-8, and Siglec-15 have been in the focus of glycomimetic drug development.

The family of the galectins differs from the previously mentioned membrane-bound lectins with respect to localization and function. Besides membrane-bound galectins, there are also soluble galectins found in the nucleus, cytosol or extracellular space. Diverse functions such as regulation of the immune system, pre-mRNA splicing, cell signalling, apoptosis, cell adhesion, wound healing as well as cancer progression and metastasis have been reported for members of the galectin family.^22^ Clinically relevant targets include Gal-1, -3, -8, and -9.

Equally important, the recognition of glycans is also exploited by pathogens for infection. For example, viruses such as HIV,^23^ hepatitis C,^24^ SARS-CoV-2^25^ or Ebola^26^ possess heavily glycosylated capsids and rely on recognition by host lectins, *e.g.* DC-SIGN, to facilitate host cell entry. The clinical relevance of lectins as drug targets is, however, not limited to mammalian lectins. Bacteria and viruses frequently employ own lectins for adhesion to host cells, determining their host cell tropism.^27^ Binding to host cell glycans and subsequent cell entry allows the pathogens to evade the immune system while providing the machinery or a nutrient rich environment for replication. Additionally, bacterial lectins are often involved in biofilm formation, a resistance mechanism shielding bacteria against the immune response and antibiotics.^28^ Consequently, these lectins significantly contribute to the virulence of pathogens and their inhibition with drugs is a promising approach to anti-infective drug research.^29^ The progress of drug development against the bacterial lectins FimH and FmlH of pathogenic *Escherichia coli*, LecA and LecB from *Pseudomonas aeruginosa*, BambL, BC2L-A and BC2L-C of *Burkholderia* species, as well as recent advances for inhibitors of Influenza A and C hemagglutinins are covered in this review.

Additionally, many bacterial and plant toxins (including ricin, cholera toxin, Shiga toxin as well as tetanus and botulinum neurotoxins) rely on lectin subunits to enter cells, where they exert their detrimental effects.^30–32^ Therefore, some of these lectin-containing toxins have also served as drug targets, *e.g.* Shiga toxin.^33–36^

Targeting lectins is therefore a highly promising, yet underexplored strategy to develop new therapies against a wide array of pathophysiological conditions ranging from autoimmune diseases and cancer to infections and neutralization of toxins. Additional therapeutic value can be gained by exploiting lectins for targeted delivery of imaging agents, drugs or vaccines.

**Fig. 1:**
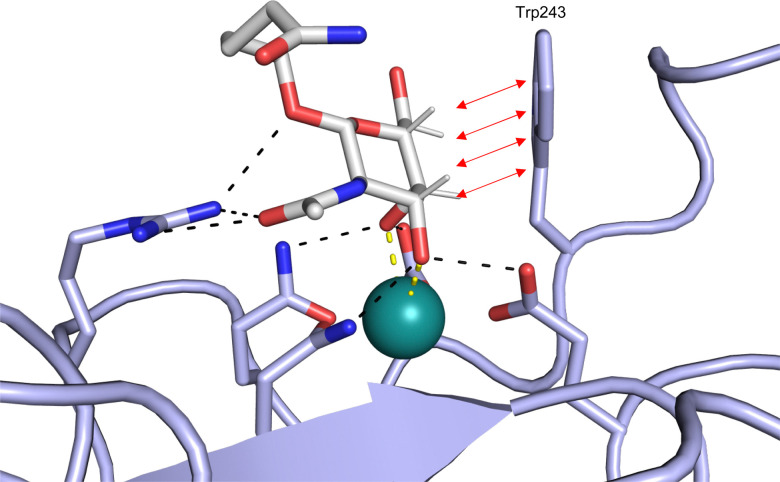
Interactions between lectins and carbohydrates represented by binding of a GalNAc derivative to ASGPR (PDB code 6YAU). Binding of carbohydrates is governed by H bonds (black), calcium ion complexation (yellow) and CH–π-interactions (red arrows).

While there is an undoubted importance of carbohydrate–lectin interactions in disease, only a limited number of drugs targeting these processes have been approved so far. One reason for this are the challenges during drug design posed by lectins themselves and their native ligands (Fig. 1). In general, lectins show a low affinity for carbohydrates in a micromolar to millimolar range.^37–39^ A major setback to high-affinity binding are the often shallow and solvent-exposed carbohydrate-binding sites (CBSs), as well as the hydrophilic natural ligands requiring costly desolvation upon binding. Cabani *et al.* estimated that desolvation of a single ligand hydroxy group requires 26 kJ mol^−1^, although carbohydrates may profit from a reduced desolvation penalty of vicinal hydroxy groups due to a shared hydrogen bond network (17 kJ mol^−1^).^40^ This cost of free energy is only partially compensated for by the formation of a single new hydrogen bond, which yields approximately 18 kJ mol^−1^ of free Gibbs energy.^41,42^ Consequently, each hydroxy group of carbohydrates must engage in more than one H bond with the protein to contribute positively to binding. Considering the high directionality of hydroxy groups and their interactions, as well as the close proximity of several OH groups in carbohydrates, these requirements for high-affinity binding are rarely met. However, the defined steric requirements for favourable interactions of the numerous OH groups also grant selectivity.

An additional feature of CBSs is an increased prevalence of aromatic amino acids, especially a 9-fold increased presence of tryptophan.^43^ Furthermore, it was shown that more electropositive C–H bonds of pyranosides engage more frequently in CH–π interactions. Because the electronic properties of specific CH bonds depend on the general stereochemistry of the monosaccharide, CH–π interactions may also contribute to selectivity of lectins. Nevertheless, the desolvation penalty associated with the high polarity of carbohydrate ligands offsets the gain of binding strength by hydrophobic interactions. High-affinity binding of carbohydrates is further hampered by an entropic penalty originating from a reduction of conformational freedom upon binding. Overall, lectins are generally considered challenging targets with a low druggability index.^44^

A further problem of targeting carbohydrate-binding proteins is selectivity. As many different lectins recognize the same minimal binding motif, *e.g.* mannose,^44^ a poorly designed carbohydrate-based drug may bind not only to the intended target, but also to off-targets. To prevent adverse drug reactions from the off-target effect, generation of specific lectin–ligand interactions is key and special attention should be paid to target selectivity during drug development.

The targeting of lectins is not only demanding because of the low affinity interactions of lectins and native ligands, but also due to the binding kinetics and global pharmacokinetic properties of native ligands. In general, carbohydrate–lectin interactions suffer from slow association (*k*_on_) and fast dissociation (*k*_off_) kinetics. While optimisation of *k*_on_ is necessary to improve binding affinity, optimisation of *k*_off_ should not be ignored. High off rates equal to reduced residence times at the target, contributing to limitation of the effective drug duration and may result in adverse drug effects due to lower selectivity.^45^ A 2018 study by Fernández-Montalván showed a significant discrepancy in the residence time of drugs under development and FDA-approved drugs with longer residence times for approved drugs, highlighting the importance of *k*_off_ for drug development.^46^ Additionally, the unfavourable pharmacokinetic properties of native carbohydrates are a major obstacle for carbohydrate-based drugs.^44^ In accordance with Lipinski's ‘rule of 5′,^47^ they often lack oral bioavailability by passive diffusion across membranes due to their polarity and active uptake in the small intestine is not guaranteed. Once systemic availability is achieved *via* parenteral routes of administration, degradation by glycosidases or fast renal elimination can severely limit the circulation half-life of native carbohydrates.^44^

In Nature, multivalency is frequently used to overcome the challenges of low affinity.^48–50^ Spatial clustering of receptors or expression of oligomeric lectins allows the simultaneous binding of multivalent ligands. In particular, the apparent binding affinity to each single binding site is increased *via* statistical rebinding or chelate effects.^51^ A striking example for the benefit of multivalency is represented by the human lectin DC-SIGN. While mannose as one of its monomeric ligands binds monomeric DC-SIGN in the millimolar range (*K*_D_ = 3.5 mM),^52^ multivalent binding of the viral glycoprotein gp120 of HIV to DC-SIGN-expressing cells occurs with nanomolar affinity.^53^ Binding of oligo- and polysaccharides may also profit from interactions with sites adjacent to the carbohydrate-recognition domain (CRD), leading to improved affinity and specificity, as demonstrated by a 130-fold affinity increase of the oligosaccharide Man_9_GlcNAc_2_ for DC-SIGN compared to monomeric mannose.^54^

**Fig. 2:**
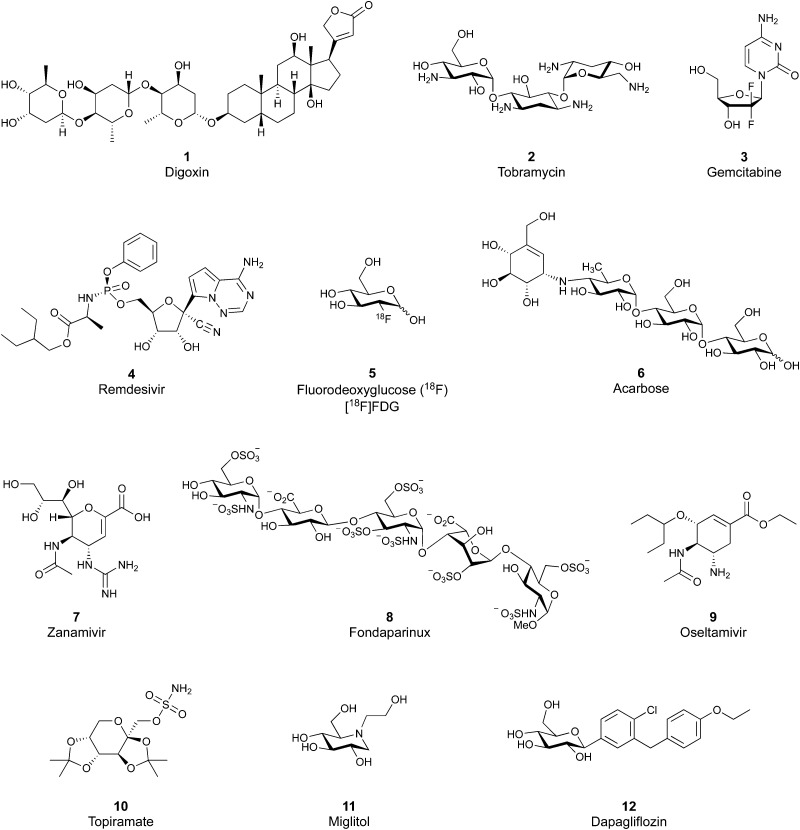
Examples of carbohydrate-based drugs in clinical use. These compounds do not interact with lectins as their primary mode of action.

Despite the mentioned drawbacks of native carbohydrates as pharmaceutical agents, a growing number of carbohydrate-based drugs is receiving approval.^55^Fig. 2 shows selected examples of carbohydrate-based drugs in clinical use. On the one hand, carbohydrates are common constituents of natural products. In these, carbohydrates may be encountered as modification of a large aglycon, *e.g.* in cardiac glycosides such as digoxin (1),^56^ or represent the major component, for example in aminoglycoside antibiotics, such as tobramycin (2).^57^ On the other hand, modification of native carbohydrates during drug design allows the development of novel carbohydrate-based drugs. The resulting compounds, referred to as ‘glycomimetics’, generally show improved drug-like properties such as affinity, selectivity or bioavailability. A class of glycomimetics with great importance are nucleoside/nucleotide analogues used in treatment of cancer and viral infections (gemcitabine (3)^58^ and remdesivir (4)^59^ as examples). The diagnostic potential of glycomimetics is exemplified by [^18^F]fluorodeoxyglucose (5) frequently used as probe in positron emission tomography (PET).^60^ In some glycomimetics, systemic distribution is not required, such as in acarbose^61^ (6) and zanamivir^62^ (7) due to a desired local effect. In others, a sufficient therapeutic effect is achieved after parenteral administration due to improved metabolic stability, for which the heparin glycomimetic fondaparinux (8) serves as example.^63^ However, even oral bioavailability can be achieved for glycomimetics, as proven by oseltamivir (9),^64^ topiramate (10),^65^ miglitol (11),^66^ and the class of gliflozine antidiabetic drugs (*e.g.*, dapagliflozin (12)).^67^ Although not lectin-binders, these compounds seeing widespread use in therapy nicely illustrate that carbohydrates are suitable starting points for drug development. In this review, we focus on the progress of the development of glycomimetics targeting lectins in the last 20 years.

## 2. Design principles for glycomimetics

Glycomimetics are structural and functional mimics of carbohydrates that can replace native carbohydrates in their interactions with target proteins. They are designed to show enhanced chemical and enzymatic stability, improved drug-like properties (bioavailability) and the same or possibly better affinity and selectivity for the target. The most efficient lectin antagonists reported to date typically contain a natural carbohydrate or carbohydrate-like scaffold, which serves as an anchor to direct the ligand to the lectin CRD. One common modification of the carbohydrate scaffold is deoxygenation, in which the oxygen atom or hydroxy groups not essential for binding are removed or replaced by a different atom or group. These modifications often lead to changes in polarity, stability, conformation, ring flexibility and hydrogen bond patterns. Importantly, deoxygenation leads to a reduction of polar surface area, which may in turn enhance binding affinity by generation of new hydrophobic interactions with the protein and reduction of the enthalpic cost of ligand desolvation. The carbohydrate or carbohydrate-like scaffold is very often further decorated with other non-carbohydrate moieties that contribute to additional interactions with the target, thus enhancing the ligand's binding affinity and specificity for the target lectin. Furthermore, these additional scaffolds also decrease ligand polarity, and thus improve its drug-like properties. Another concept frequently used in the design of glycomimetics is conformational preorganization of a molecule, which has been shown to significantly reduce entropic penalty associated with ligand binding.^68,69^

In this section, we will classify glycomimetics according to their structural features and briefly mention their general characteristics. Some approaches to the design and synthesis of glycomimetics have been reviewed recently by Bernardi,^70,71^ Janetka,^72^ Hevey,^73,74^ Vidal,^75^ and others. While these reviews focus on selected lectin targets only, in the following sections, we aim at providing a comprehensive overview of the design and application of glycomimetics for most known clinically relevant lectin targets.

### 2.1 Modification of the *O*-glycosidic linkage

**Fig. 3:**
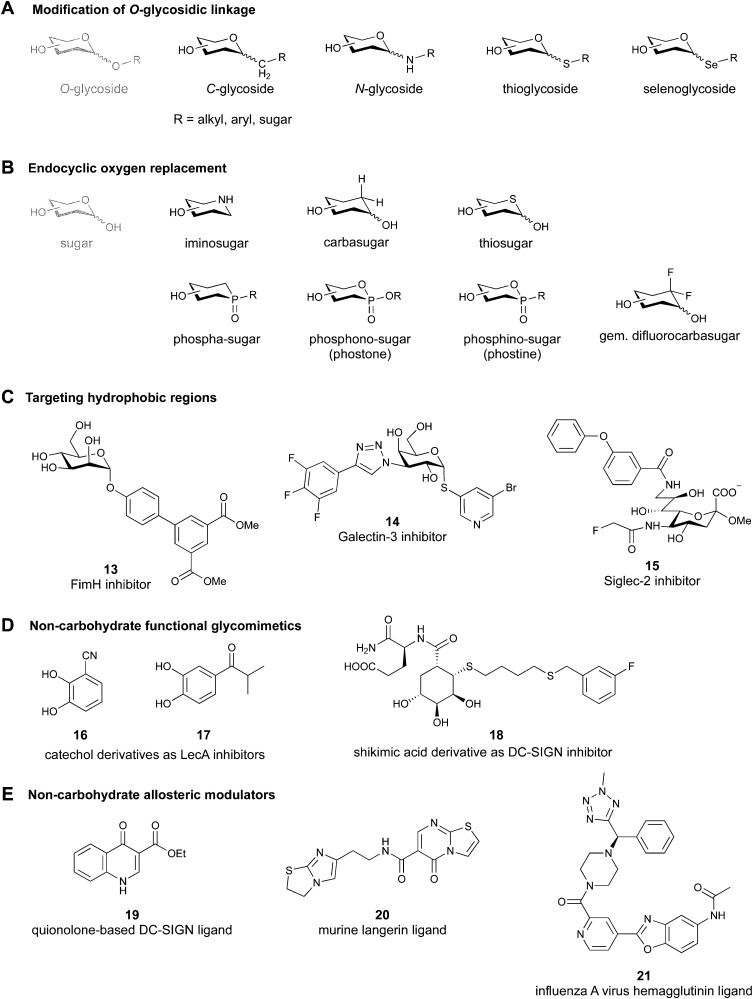
Common modifications of the native carbohydrate structure (A and B) and examples of lectin-binding glycomimetics following the different design strategies (C–E) discussed in Section 2.

Since native *O*-glycosides are prone to chemical and enzymatic hydrolysis *in vivo*, a commonly used strategy to improve pharmacokinetic properties such as bioavailability and serum half-life is replacement of oxygen by an atom, which would form a more stable linkage, such as nitrogen, carbon, sulfur or selenium (Fig. 3A). Acylated *N*-glycosides are extensively used in the glycosylation of peptides, both natural and synthetic.^76–78^ In contrast, *N*-glycosidic bonds as an *N*,*O*-aminal between two carbohydrate units are relatively labile and therefore only scarcely described.^79^ While *C*-glycosides^80^ and *C*-acylglycosides (reviewed in ref. 70) are hydrolytically stable glycoside mimics, the introduction of the carbon atom leads to a loss of the exo-anomeric effect and can induce undesirable conformational changes. Thioglycosides serve as more stable analogues of *O*-glycosides owing to the fact that sulfur is less basic than oxygen and thus, an *S*-glycosidic bond is more resistant towards hydrolysis. Apart from showing various biological activities,^81–83^ selenoglycosides have been used as tools for the crystallographic investigation of carbohydrate–protein interactions.^84–88^ In addition, both seleno- and thioglycosides are used as glycosyl donors in the synthesis of oligosaccharides.^89–92^

### 2.2 Endocyclic oxygen replacement

Replacement of the endocyclic oxygen atom with nitrogen, carbon, sulfur, and phosphorus leads to imino-, carba-, thio-, and phosphorous-based glycomimetics, respectively (Fig. 3B).

Iminosugars are the largest group of monosaccharide mimics reported so far,^93,94^ they occur in nature and can be found in different plants and microorganisms.^95,96^ Iminosugars can be divided into two groups: monocyclic (pyrrolidines, piperidines, azepanes) and bicyclic (pyrrolizidines, indolizidines, nortropanes). At physiological pH, the endocyclic nitrogen atom is positively charged; iminosugars can hence mimic the charged oxocarbenium transition state of processing enzymes and have found clinical use as glycosylhydrolase and -transferase inhibitors.^97^

Carbasugars (cyclitols) lack the typical anomeric reactivity, which leads to their increased metabolic stability towards glycosidases and glycosyltransferases.^98,99^ Moreover, replacement of the endocyclic oxygen with carbon prevents the anomeric effect and changes the hydrogen-bond pattern, flexibility and conformation of the ring.^100^ Thiosugars^101,102^ are more hydrophobic in nature than their oxo-counterparts and can sometimes show enhanced affinity through hydrophobic interactions with the protein.^103^ Phosphorus-based glycomimetics contain a phosphorus atom in place of the anomeric carbon or in place of the endocyclic oxygen.^104,105^ Three main classes of such compounds can be distinguished: phospha-, phosphono- (phostones), and phosphino-sugars (phostines, 1,2-oxaphosphinanes). The phosphinolactone group (O–P=O) in phostones and phostines serves as a bioisostere of hemiacetals (O–C–OH) with phosphorus replacing the anomeric carbon. Fluorinated glycomimetics are also attracting ever increasing attention. Recently, it has been shown that replacing the endocyclic oxygen with a CF_2_ group can imitate the anomeric effect.^106,107^

### 2.3 Replacement of OH functional groups

Deoxygenation, *i.e.* OH to H transformation, leads to a reduction of polar surface area and thus, favours desolvation and may establish new hydrophobic contacts with the protein. Additionally, the removal of an electron-withdrawing group increases electron density of the scaffold and may make other OH groups more nucleophilic, and even strengthen interactions such as metal coordination or hydrogen bonding.^108^

The OH group can be replaced by its bioisosteres, such as F, OCH_3_, SH, SeH, and NH_2_. The high electronegativity of fluorine results in a high polarization of the C–F bond. The presence of a fluorine atom can increase lipophilicity,^109^ decrease p*K*_a_ values of neighbouring OH groups, and modulate the hydrogen-bond donor/acceptor properties.^70^ Additionally, fluorine atoms as electronegative substituents destabilize the oxocarbenium transition state, which is present in enzymatic glycosidic bond hydrolysis.^74,110^ Etherification of an OH group is widely used to assess the binding requirements for ligand interactions and can sometimes even be a requirement for recognition by the lectin.^111^ Since sulfur and selenium are larger and more polarizable than oxygen, their introduction leads to enhanced lipophilicity, weaker H-bond donor properties and better π-interactions. However, thiol or selenol replacement is rare due to their challenging synthesis and redox instability. Finally, the amino group becomes positively charged at physiological pH, and therefore poorly mimics the neutral OH group.

### 2.4 Addition of lipophilic fragments

When designing glycomimetics, targeting peripheral regions of the binding site with lipophilic fragments has been often successfully exploited to generate new hydrophobic interactions with the protein surface. Representative examples are FimH antagonists targeting a tyrosine gate (*e.g.*13),^112–114^ Siglec-2 inhibitors binding a hydrophobic area through modification at C-9 (*e.g.*15),^115^ and Gal-3 inhibitors (*e.g.*14) interacting with arginine residues in the binding site (Fig. 3C)^116,117^ and will be discussed in detail below.

To this end, biphenyl moieties have become a popular scaffold in many glycomimetics following the anticipation that the biphenyl motif is a replacement for a disaccharide. However, the biphenyl substituent is frequently deeply buried in a hydrophobic cleft (*e.g.* Siglec-1, -2, DC-SIGN and FimH), being engaged in π-stacking and hydrophobic interactions that increase binding affinity. Nevertheless, in the case of FimH the biphenyl residue indeed occupies a binding site in the tyrosine gate that is normally occupied by mannose residues, although in a slightly different conformation.^112,114^ The biphenyl residue has also found application in FmlH glycomimetics, where it extends interactions within the binding pocket, thereby increasing potency.^118,119^ Furthermore, biphenyls have been introduced into MAG antagonists as scaffolds to position two Neu5Ac residues in their bioactive conformation.^120^

### 2.5 Other approaches

Non-carbohydrate functional glycomimetics are an interesting alternative to traditional glycomimetics. These are compounds that do not contain a carbohydrate scaffold, but still bind to the carbohydrate binding site and functionally mimic carbohydrates. Representative examples include catechols (*e.g.*16, 17) and hydroxamic acids as LecA inhibitors,^121,122^ and shikimic acid derivatives as DC-SIGN inhibitors (*e.g.*18, Fig. 3D).^123^

Another strategy to circumvent the drawbacks of carbohydrate-based drugs is targeting allosteric sites with higher druggability using a better suited chemical scaffold. Upon binding, these allosteric ligands modify the CBS, thereby affecting carbohydrate recognition. Recent experimental data suggest existence of such druggable secondary sites, especially in mammalian C-type lectins and galectins.^124–128^ Examples of allosteric inhibitors include 4-quinolones^129^ as DC-SIGN inhibitors (*e.g.*19), thiazolopyrimidines (*e.g.*20)^126^ as murine langerin inhibitors and pyridinylbenzoxazols (*e.g.*21) as stabilizers of the prefusion state of hemagglutinin from Influenza A^130^ (Fig. 3E).

A further strategy frequently used, especially in targeting of bacterial and viral lectins, is multivalency, although mostly terminal native mono- or oligosaccharides are employed. Multivalent glycosides (*e.g.*, glycodendrimers, glycoclusters, glycopolymers, glyconanoparticles) contain multiple sugar moieties and are designed to bind either one lectin at multiple binding sites or several lectins of the same type, promoting receptor clustering or aggregation. While these compounds usually show high affinity,^51,131,132^ they can also suffer from pharmacokinetic drawbacks due to their large size, high polarity, lack of oral bioavailability, and high likelihood of off-target effects and eliciting an unwanted immune response.^133^ Consequently, in this review we focus mainly on monovalent glycomimetics and only highlight those multivalent ligands that are of interest for our subjective focus. For details on multivalency, the reader is further referred to other reviews in this issue and elsewhere.^51,131,132^

## 3. Inhibitors of lectin function

Lectins are attractive targets for chemical biology and medicinal chemistry and consequently a large number of approaches for the inhibition and modulation of various animal, bacterial and viral lectins have been pursued during the last 40 years. The most common approach is the development of glycomimetics based on the native carbohydrate ligand of the targeted lectin, and the more recent advances of the last two decades will be reviewed in Section 3.1. However, recent research has also led to the discovery of novel glycomimetic scaffolds devoid of a carbohydrate motif as direct binders of the carbohydrate recognition site and these are described in Section 3.2. Finally, allosteric modulators have now also been reported for lectins and their development is summarized in Section 3.3.

The various lectins of interest from animal, bacterial and viral sources will be introduced in the following sections when they are first described. Not surprisingly, a number of lectins are addressed by multiple approaches. For example, carbohydrate-derived glycomimetics and novel glycomimetic scaffolds have been developed for bacterial lectins, and C-type lectin receptors proved suitable for allosteric modulators in addition to carbohydrate-derived glycomimetics.

### 3.1 Progress towards carbohydrate-derived glycomimetic drugs over the last 20 years

#### 3.1.1 C-type lectins

CTLs are the largest family of carbohydrate binding proteins in mammals and are subdivided into 16 groups based on their phylogenetic relationships and domain structure.^134^ They mediate many processes, for example cellular adhesion, self/non-self recognition and glycoprotein turnover.^135–137^ In particular, their role in immune regulation has led to several approaches targeting CTLs for the development of anti-inflammatory or anti-microbial drugs.^44,135,138,139^

**Fig. 4:**
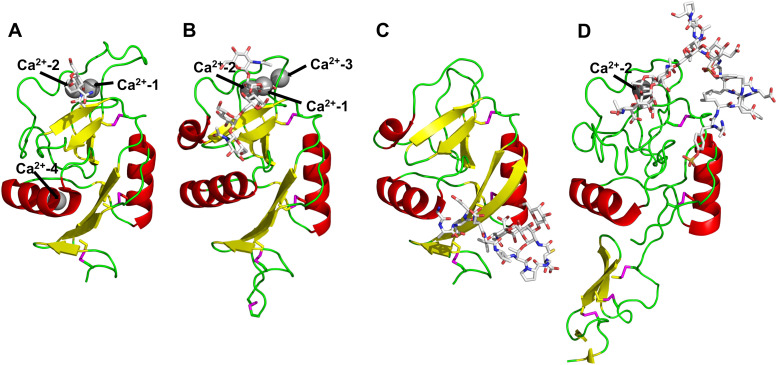
C-type lectin domain fold. Calcium ions are shown as grey spheres and named according to Zelensky and Gready.^142^ (A) ASGPR in complex with GalNAc derivative 22 (PDB code 6YAU, see Fig. 43) showing only the resolved part of the trivalent ligand. (B) A complex of DC-SIGN/GlcNAc_2_Man_3_ highlights the extended secondary site for larger oligosaccharides. Additional contacts beyond the Ca^2+^ coordination play an important role for oligosaccharide specificity and affinity (PDB code 1K9I). (C) CLEC-2 in complex with a sialylated glycopeptide revealing a non-canonical carbohydrate recognition site (PDB code 3WSR). (D) Complex of PSGL-1 peptide and P-selectin showing the extended binding site (PDB code 1G1S). Disulfide bridges are highlighted in magenta.

All CTLs share the C-type lectin domain (CTLD),^140–143^ a looped structure with N- and C-terminal antiparallel β-sheets connected by two flanking α-helices (Fig. 4). The hydrophobic centre of this domain is built by a three-stranded antiparallel β-sheet, stabilized by at least two pairs of highly conserved disulfide bridges. The core harbours a remarkably high number of tryptophans, one of them being part of the conserved ‘WIGL’ motif,^140,141^ providing stability to the fold.^142^ The majority of CTLs bind carbohydrates by coordinating their hydroxy groups *via* a name-giving central Ca^2+^ ion. The ‘WND’ motif, located in the β-sheet, couples the canonical Ca^2+^ cage to a hydrophobic core of the CTLD.^142^ Besides this central Ca^2+^ cage, the CTLD can host up to three additional Ca^2+^ ions. Overall, all four Ca^2+^ sites are located in the upper loop and are referred to as Ca^2+^ sites 1 to 4, with Ca^2+^-2 site being located in the carbohydrate site. Taken together, these stabilizing elements generate a high hydrophobicity of the core, while the disulfide bridges and the Ca^2+^ cages provide remarkable stability to the CTLD. This leads to a high tolerance for a sequence variation, a necessity for immune cell receptors coevolving with pathogens.^144^

**Fig. 5:**
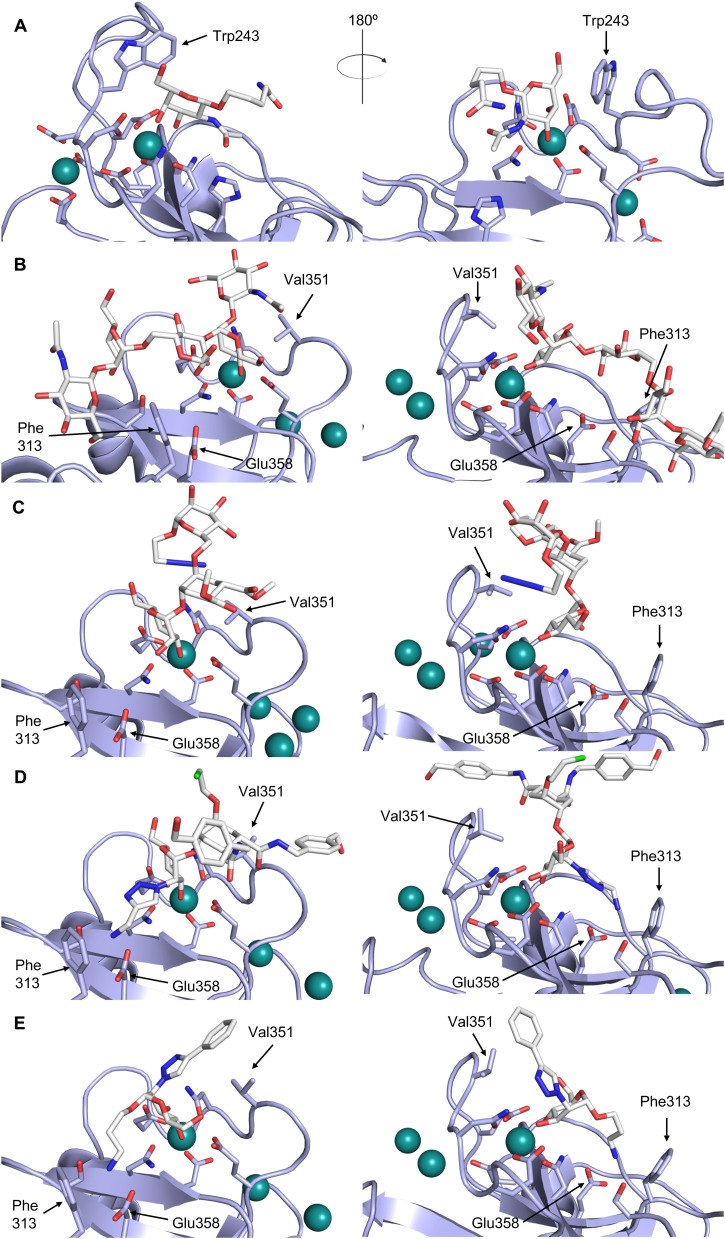
C-type lectin canonical carbohydrate binding site. (A) ASGPR in complex with GalNAc derivative 22 emphasizes the affinity gain by CH–π interaction with Trp243 (PDB code 6YAU). (B–E) DC-SIGN in complex with (B) GlcNAc_2_Man_3_ (PDB code 1K9I), (C) pseudo-trisaccharide 23 (PDB code 2XR6), (D) pseudo disaccharide 24 (PDB code 6GHV), and (E) glycomimetic 25 (PDB code 7NL7). As a common design principle, a significant gain of affinity and specificity is achieved by interactions with Phe313 and Val351 in close proximity to the carbohydrate recognition site.

CTLs recognize their carbohydrate ligands by coordinating two vicinal hydroxy groups of a central monosaccharide motif (Fig. 5). The canonical Ca^2+^-2 site is either embedded in an EPN or QPD amino acid sequence motif, dictating mannose/fucose/glucose or galactose specificity, respectively.^134,145^ This motif is located in the evolutionary and structurally variable long loop. The two hydroxy groups of the carbohydrate ligand can be coordinated by the Ca^2+^ in two orientations of the core scaffold being 180° flipped. However, the monosaccharide recognition alone provides neither sufficient affinity nor specificity in a biological context. Therefore, oligosaccharide specificity arises from secondary sites close to the canonical carbohydrate binding site. These sites allow for the recognition of extended oligosaccharide structures,^146^ a principle found in many lectins that originate from convergent evolution.^147^ For efficient design of glycomimetics, this important feature was used during the design of selectin antagonists. This circumvented the low carbohydrate affinities coming from high desolvation costs of the hydrophilic recognition site, and entropic costs originating from loop rigidification upon binding.^148,149^

For endocytic CTLs, the Ca^2+^ has not only a stabilizing function for the CTLD, but also provides means for ligand release in the endosome since it is also the structural element generating pH sensitivity for release of the cargo.^136,150,151^ At low pH and active Ca^2+^ export of the endosomal environment, some CTLs may recycle back to the plasma membrane with a remarkably high speed and efficiency.^152,153^ This is an important feature of this receptor class making them attractive for targeted delivery approaches using glycomimetics (see Section 4).

To the best of our knowledge, there are only a few exceptions of CTL binding carbohydrates in a non-canonical site, in the absence of Ca^2+^ coordination: dectin-1 and CLEC-2.^154,155^ Some CTLs provide secondary carbohydrate sites in addition to their canonical primary site. Examples are SIGN-R1 carrying a binding site for repetitive microbial polysaccharides and dextran sulfate on the opposite side of the CRD^156^ and langerin recognizing larger glycosaminoglycans in a remote site.^157,158^ With respect to the development of glycomimetics, these secondary sites might play an important role as they provide opportunity to target sites not amenable to traditional medicinal chemistry.^44,159–162^

Further mammalian lectin targets related to health and disease include the proinflammatory CLEC9A receptor group^163^ as well as DEC205 (CD205), a receptor responsible for self-antigen uptake, especially in the context of cell apoptosis.^164^

##### 3.1.1.1 Selectins

The protein family of CTLs has been focus of very active drug research for many years, spearheaded by early attempts to target the subfamily of the selectins, which has resulted in glycomimetics advancing into Phase III clinical trials.

Selectins are a subfamily of CTLs consisting of P-, E- and L-selectin. They are homing receptors involved in cell adhesion and leukocyte trafficking and are conserved between species. They have first been identified on epithelia, platelets and leukocytes, and thus, named E-, P-, and L-selectin or CD62E, -P, and -L. These proteins are essential for the inflammatory response and mediate the recruitment of leukocytes and lymphocytes into inflamed tissue. Thus, the selectins have been identified as potential drug targets in all diseases with excessive inflammatory response.^44^ One indication where glycomimetic selectin antagonists are under development is sickle cell disease, with anti-selectin antibodies already in clinical use.^165^ Furthermore, the selectins also mediate cancer metastasis opening new anti-cancer treatment options.^166^

**Fig. 6:**
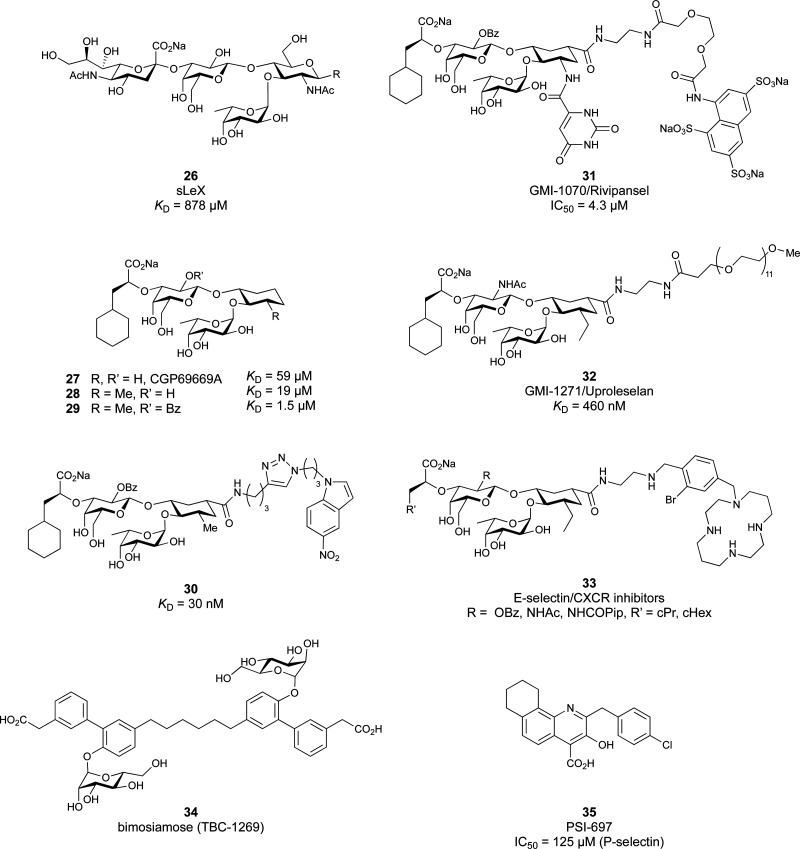
Sialyl LewisX (26) and its glycomimetic analogues. While major modifications or replacement of the sialic acid and GlcNAc motifs of sLeX are viable strategies in the design of selectin antagonists only minor modifications to the fucose and galactose are tolerated. Indicated *K*_D_s are for the binding to E-selectin if not stated otherwise.

These type I transmembrane proteins carry a CRD directly connected to an epidermal growth factor (EGF)-like domain. The minimal binding motif of E-, P- and L-selectin is the tetrasaccharide sialyl LewisX (sLeX, 26, Fig. 6) binding to the canonical carbohydrate binding site, while a secondary site in P-selectin binds sulfated tyrosines generating specificity and affinity for PSGL1 (Fig. 4-D), its natural glycoprotein ligand.

**Fig. 7:**
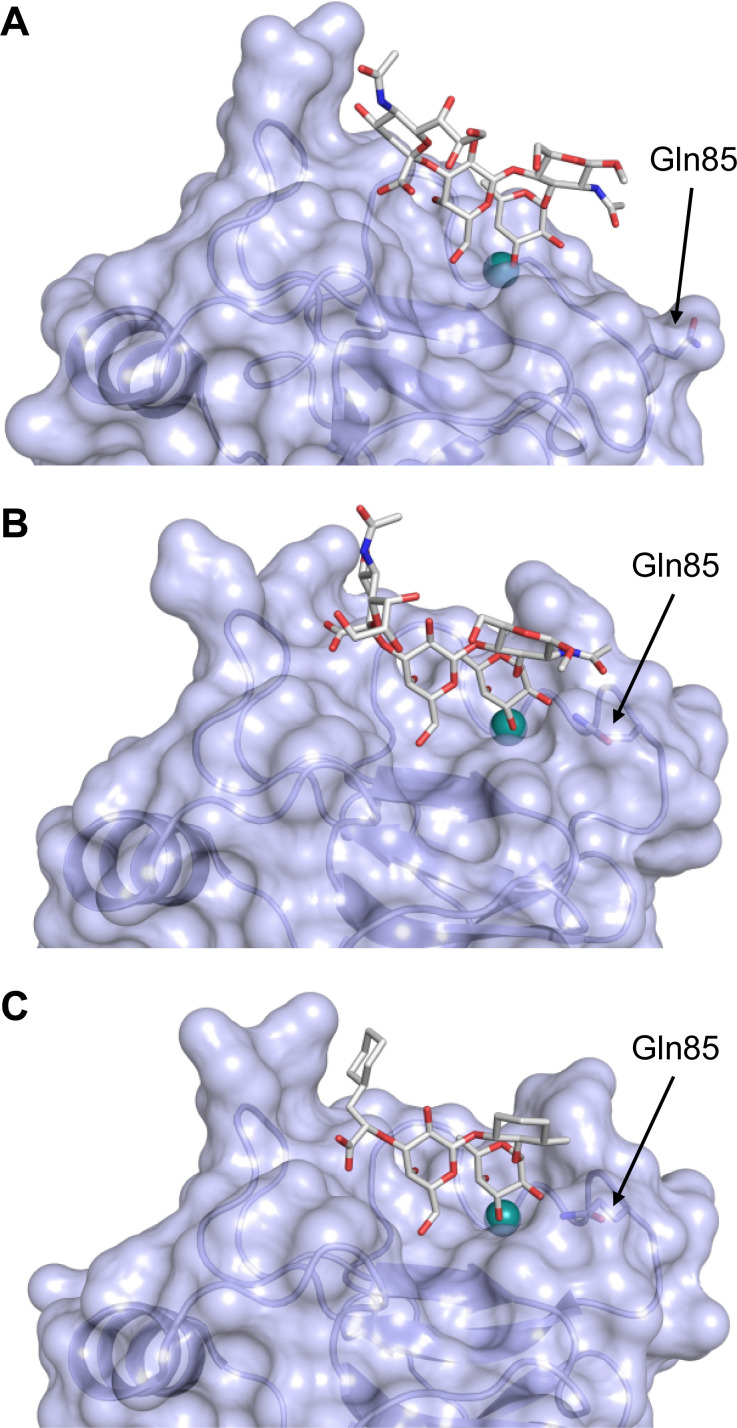
E-selectin in complex with sLeX (A and B) and glycomimetic 28 (C). The structures of the tetrasaccharide sLeX soaked into crystals (A, PDB code 1G1T) or cocrystallised with E-selectin (B, PDB code 4CSY) reveal drastic conformational differences of protein and ligand highlighted by relocation of Gln85 shown as stick. Glycomimetic 28 cocrystallised with E-selectin (C, PDB code 4C16).

This is supported by a crystal structure of E-selectin with sialyl LewisX which originated from soaking experiments (Fig. 7A).^167^ Moreover, a PSGL-1 glycopeptide fragment bound to P-selectin induced an extended conformation suggesting a conformational coupling of the secondary and the primary sites (see Section 3.3.3).^167^ However, a co-crystal structure of a four-domain fragment comprising the CRD, the EGF and two additional short consensus repeat (SCR) domains in presence of sialyl LewisX shows the extended conformation with conformational stretching of E-selectin (Fig. 7B). This extended conformation is essential for the catch-bond underlying the molecular recognition responsible for leukocyte recruitment.^149^ Strikingly, in the new ligated structure the long loop changes its conformation, in particular Gln85 is relocated by about 10 Å. Consequently, the low-affinity structure of E-selectin, present in solution, recognizes the carbohydrate.^149,167^ This recognition allows the transition to a high affinity, extended state, already in the absence of flow conditions, suggesting a two-state model.^149^

Because all three proteins share sLeX as minimal binding epitope, this tetrasaccharide motif has served as a lead for the development of new glycomimetics as pan-selectin antagonists (Fig. 6).^44^ sLeX binds to the selectins’ rather flat carbohydrate binding site through its sialic acid, galactose and fucose residues.^149,167^ Ernst and co-workers reported that the binding of sLeX to E-selectin is fully entropy-driven and provided the concerted displacement of several protein bound water molecules by the ligand's hydroxy groups as a rationale.^168^

Development of selectin-inhibiting glycomimetics started in the mid-1990s and has flourished owing to the fact that numerous major pharma companies were active in the field, *e.g.* Hoechst/Sanofi, Ciba/Novartis and Wyeth.^169,170^ The essential pharmacophores of sLeX were identified and it became evident that its fucose and galactose residues as well as the carboxylic acid group of sialic acid are essential for selectin binding, while the rest of the sialic acid and the GlcNAc could be replaced.^44^ The bioactive conformation of this molecule has been determined by NMR spectroscopy^171^ and it was reasoned that inhibitors must have pre-organized pharmacophores in order to be effectively bound by the selectins under the prevailing flow in blood vessels.^68^

In the early lead molecule CGP69669A (27), (*S*)-cyclohexyllactic acid was chosen as a sialic acid substitute and the GlcNAc residue was replaced with cyclohexanediol.^68^ This molecule was over 10-fold more active than sLeX and showed efficacy on disrupting leukocyte rolling *in vivo*.^172^ The introduction of additional substituents on the cyclohexanediol adjacent to the fucose moiety further improved activity, *e.g.* in compound 28, which was assigned to an increased steric compression of the molecule leading to lower entropy costs upon binding.^69,173^ The crystal structure of 28 in complex with E-selectin has recently been solved and demonstrates the interactions of the cyclohexyllactic acid with the protein (Fig. 7C). By keeping the 2-hydroxy group of the galactose residue benzoylated, affinity was further increased 10-fold, resulting in the single digit micromolar selectin antagonist 29. Finally, a carboxylate substituent was introduced at the cyclohexanediol,^173^ which allowed the addition of second site binders resulting in the 30 nM E-selectin inhibitor 30.^159^ This development finally led to the synthesis of GMI-1070 (31, Rivipansel), which possesses an orotamide substituent adjacent to the fucose and a distant naphthalene trisulfonate.^174^ The latter pharmacophore is necessary to target the additional anion recognition site in P-selectin (Fig. 4D), which efficiently binds to its native ligand PSGL-1 through the sLeX carbohydrate epitope and an additional sulfate present in PSGL-1. GMI-1070 was moved to clinical development for the treatment of vaso-occlusive crises in sickle cell anaemia, but failed at the latest stage, in Phase III clinical trials for lack of efficacy to meet its primary endpoint,^175^ although beneficial tendencies were observed according to Magnani.^176^

Recently, Ernst and co-workers addressed the pharmacokinetic properties of 27 and 28 in a prodrug approach aiming at oral bioavailability of the active substance.^177^ To this end, several linear, branched and cyclic alkyl esters have been synthesized and evaluated. This modification indeed led to an increase in permeability through artificial membranes and across Caco-2 cells *in vitro*, but oral bioavailability could still not be achieved in mice. On the down side, the authors also noted that the promising cyclohexylmethyl ester of 28 was rather quickly metabolized by liver microsomes and the resulting hydroxylated derivative was a poor substrate for the esterase cleavage. The latter is however essential to liberate the active selectin inhibitor 28.

The company Glycomimetics Inc. has two further sialyl LewisX mimetics in development with distinct traits for specific indications. The first compound GMI-1687 (structure undisclosed) is a 2.4 nM E-selectin binding compound with s.c. bioavailability and efficacy in an animal model of thrombosis at 40 μg kg^−1^ administered twice daily.^178^ This trait improves its route of administration from the clinics to also outside the hospital and should help patients at an earlier stage of sickle cell disease. The compound will enter first in human trials in 2022.^179^

The second drug candidate is Uproleselan (32, GMI-1271), a selective E-selectin antagonist (IC_50_ = 2.4 μM) devoid of binding to P-selectin up to 10 mM and 5 mM binding to L-selectin. This compound is designed to address E-selectin function in cancer.^180,181^ The structure is also derived from CGP69669A with the 2-*O*-benzoylated galactose residue now replaced by a GalNAc and the cyclohexanediol residue further conformationally stabilized by an equatorial ethyl group; furthermore, an 11 mer-PEG chain is attached to the carboxylic acid to improve serum half-life and reduce plasma protein binding.^181^ Presumably, the lack of P-selectin binding results from loss of the naphthalenetrisulfonate residue that is present in the pan-selectin inhibitor Rivipansel (GMI-1070). Uproleselan was active in a mouse model of acute myeloid leukaemia (AML).^182^ GMI-1271 is currently in Phase III clinical trials for the treatment of AML (NCT05054543) and Phase I/II trials for COVID-19 pneumonia (NCT05057221).

As an advancement in the field, bifunctional inhibitors of E-selectin and the chemokine receptor CXCR4 have been developed to synergistically counteract osteosarcoma and a patent reports the conjugation of a CGP69669A derivative with cyclam,^183^ a tetraaza-crown ether that binds to carboxylates within CXCR4.^184^ GMI-1359 belongs to this class of bifunctional compounds (exact structure undisclosed, general structure 33) and has proven effective in animal models of bone metastasis, pancreatic cancer and AML when co-administered with chemotherapy.^185–187^ GMI-1359 has been studied in a Phase I clinical trial (NCT04197999) for the treatment of breast cancer and results remain to be disclosed.

Another glycomimetic molecule, bimosiamose (34, TBC-1269) originating from Texas Biotech Corporation, was under development as pan-selectin antagonist. This molecule is a symmetric dimer of a sLeX mimetic where the sialic acid is replaced by an acetic acid linked to a biphenyl, the latter serving as a spacer to a mannose residue that mimics the fucose in sLeX.^188^ Interestingly, this sLeX mimic showed no effect on leukocyte rolling *in vivo* suggesting a distinct mechanism of action.^189^ TBC-1269 was tested under the lead of Revotar in three Phase II clinical trials focusing on human allergen challenge model of asthma,^190^ reducing inflammation in COPD,^191^ and on ozone-induced airway inflammation.^192^ However, bimosiamose's current development status is unknown and Revotar was terminated in 2017.

Furthermore, the naphthalene carboxylate PSI-697 (35) originated from a screening campaign followed by optimisation and has been developed as a P-selectin antagonist.^193^ This molecule showed inhibition of P-selectin binding to immobilized PSGL-1 by SPR *in vitro* (IC_50_ = 125 μM) and inhibited leukocyte rolling in murine blood vessels. Interestingly, the mode of action of PSI-697 with P-selectin is unknown. It remains to be elucidated if this compound that completely lacks any carbohydrate character is a direct competitor to sLeX, acts on the secondary site bound by the sulfated tyrosine, acts as an allosteric inhibitor, or has another mode of action. Further, PSI-697 showed some efficacy in mouse models of atherogenesis and vascular injury^193^ as well as in thrombosis.^194^ The molecule was then moved to Phase I clinical trials in inflammatory diseases, and administered per orally in scleritis (NCT00367692) in 2006 and, furthermore, in a 2008 study by Pfizer for platelet aggregation with monocytes in smokers (NCT03860506). In the latter study, the results revealed the inactivity of PSI-697 on the inhibition of platelet aggregation whereas a P-selectin antibody was effective.^195^ These data urge for the structural determination of the mode of action of PSI-697 with P-selectin and, furthermore, demonstrate the challenges associated with the development of glycomimetics.

##### 3.1.1.2 DC-SIGN

One of the most studied members of the CTL family is DC-SIGN.^196,197^ This homotetrameric receptor has a broad expression profile and can be found on monocytes, dermal DCs, macrophages and platelets amongst others.^198,199^ The latter finding renders the *in vivo* application of high affinity, multivalent DC-SIGN ligands likely risky for the induction of thrombosis. Since its discovery as an uptake receptor for HIV allowing the hijacking of the mucosal DCs for dissemination of the virus and subsequently transinfecting CD4^+^ T cells, DC-SIGN became a prime target for the development of antagonists.^44,200,201^ However, a clear experiment for target validation *in vivo* is missing, because of the absence of a suitable animal model.^138^ Many pathogens have been found to be recognized by DC-SIGN such as *Mycobacterium tuberculosis*, *Leishmania*, Ebola virus, and *Candida albicans*.^26,202–204^ Recently, DC-SIGN has also been identified as a co-receptor for SARS-CoV-2, together with a number of other CTLs L-SIGN, LSECtin, ASGPR1, and CLEC10A, recognizing the spike protein.^25,205^ Lectin engagement with the virus induces proinflammatory responses and this early event during infection is correlated with COVID-19 severity. These findings have again spurred interest limiting the lectin-induced hyperactivation by the application of lectin inhibitors as a potential target for COVID-19 therapy.

Two routes have been followed to generate glycomimetic ligands for DC-SIGN. For targeting the primary carbohydrate binding site, carbohydrate-based ligands were derived starting from a monosaccharide or disaccharide unit. Alternatively, there are several reports on non-carbohydrate ligands, likely targeting secondary sites that may even allosterically modulate the primary carbohydrate site (see Section 3.3).^13,24–26^

Carbohydrate-derived glycomimetic inhibitors for DC-SIGN have been developed showing affinities in the lower two-digit micromolar range.^206–213^ The development highlights the challenges and opportunities during the process of designing inhibitors for mammalian lectins due to their shallow and hydrophilic binding sites. Moreover, starting from a promiscuous starting point such as a monosaccharide, keeping potential off-targets in mind is a prime concern. Mannose (36) has low affinity as monosaccharide (*K*_D_ = 3.5 mM) and fucose is only slightly better.^197,214–216^

**Fig. 8:**
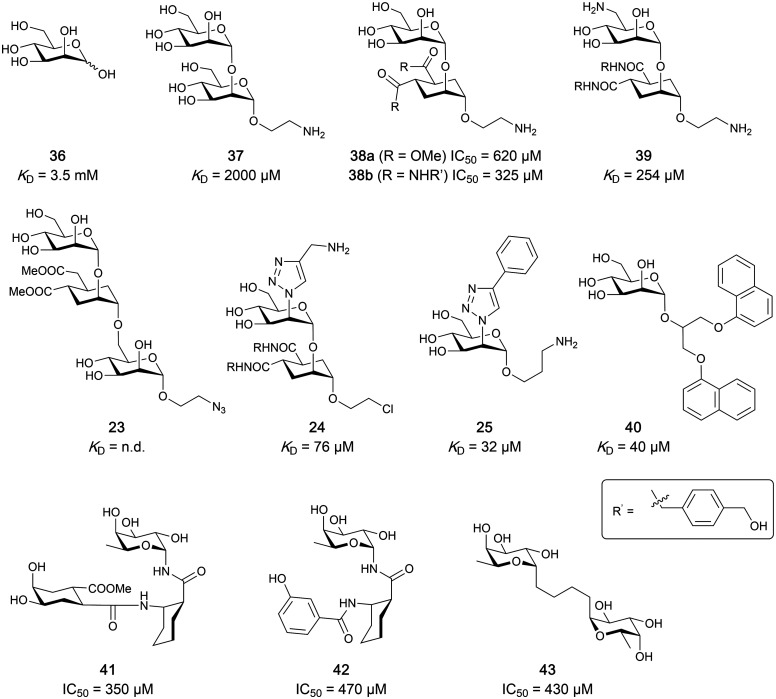
Carbohydrate-based glycomimetic DC-SIGN inhibitors. The ligands are derivatives of mannose (25, 40), mannobiose (24, 37–39) and mannotriose (23) and fucose (41–43).

One of the most advanced routes for the development of a carbohydrate-based glycomimetic inhibitor is based on the ethanolamine modified Manα(1,2)Man disaccharide 37, recently nicely summarized elsewhere.^138,139^ Briefly, disaccharide 37 does not provide the basis for specific recognition by DC-SIGN over other CTLs such as langerin, dectin-2, mannose receptor, and mannose binding lectin. Consequently, additional modifications were introduced to increase specificity (Fig. 5 and 8). The substitution of the reducing end mannose substructure with a cyclohexane in 38a led to improvement of specificity and at the same time increased affinity with positioning the aliphatic ring onto Val351 (Fig. 5C and 8).^208,210,217^ Next, rational design led to the expansion of the cyclohexane by additional substituents leading in 38b to a two-fold affinity increase, but strikingly to even higher selectivity over langerin, one of the potential off-target receptors (Fig. 8).^211,212,218^ With additional substituents at the non-reducing end mannose, an additional affinity and selectivity gain was achieved (39, 24).^210^Fig. 5D shows 24 in complex with DC-SIGN. This was a critical step for biophysical and biochemical data analysis, since additional substitutions at the mannose core have previously been reported to enhance protein aggregation in activity assays and consequently mislead the mode of action analysis of such inhibitors.^213^ This was accounted for using appropriate controls such as analytical ultracentrifugation to carefully monitor protein aggregation.^210^

In a similar design approach, Ernst and co-workers explored the periphery of mannose with a focused library, also making use of the hydrophobic interactions with Val351, a charge-assisted hydrogen bond to Glu358 and cation–π contact with Phe313, as proposed earlier (Fig. 5E and 8).^210,219^ Taken together, this led to 25 with an affinity of 32 μM for DC-SIGN and full selectivity over langerin.^219^ Earlier studies tried to make use of the hydrophobic environment of Phe313 starting from the anomeric position of mannose as a scaffold^209^ and achieved similar affinities (40, *K*_D_ = 40 μM). However, clear evidence for the orientation of 40 in the binding site is still missing, in particular taking into account aggregation effects^213^ and secondary sites.^160,162^ Along the same lines, in an elegant approach using phage display, primary site binding mannose was used to anchor a secondary site-binding peptide yielding high specificity and a 15-fold increase in affinity over the initial monosaccharide.^220^ Yet, despite the progress made in the development of carbohydrate-based DC-SIGN inhibitors, only multivalent display of the presented compounds demonstrated inhibitory constants sufficient for the successful application as anti-infectives in preclinical models, such as inhibition of trans-infection of ACE2^+^ cells by SARS-CoV-2 spike protein-expression vesicular stomatitis virus or SARS-CoV-2 isolates.^219,221^

It is interesting to note that albeit fucose has a higher affinity for DC-SIGN compared to mannose,^216,222,223^ it has less often been explored for carbohydrate-based glycomimetic design. Chemical tractability is likely one of the main reasons for this. Building on the LewisX trisaccharide several fucose-based glycomimetics such as 41 and later 42 were developed.^216,222^ Capitalizing on statistical rebinding, a divalent structure 43 was evaluated as potent DC-SIGN inhibitor among a *C*-glycoside series of mannose and fucose derivates.^223^

#### 3.1.2 Galectins

Galectins are a family of soluble lectins that specifically bind β-galactosides, such as lactose (Lac, Gal-β(1,4)-Glc) or *N*-acetyllactosamine (LacNAc, Gal-β(1,4)-GlcNAc).^224^ The CRD of galectins has *ca.* 130 amino acids.^225^ Out of these, around 20 amino acids are highly conserved among the galectin family, including eight residues shown to be involved in glycan binding. Most galectin CRDs also contain a variable number of free cysteine residues, which led to the denomination as S-type lectins. Because of these cysteine residues, some galectins require a reducing environment for their carbohydrate-binding activity.^226^

To this date, a total of 16 mammalian galectins have been identified.^227^ They are defined by a conserved CRD and a common structural fold.^226^ Galectins are categorized into three groups according to the organization of their CRD: prototype galectins (Gal-1, -2, -5, -7, -10, -11, -13, -14, -15, -16) contain one CRD and form homodimers in solution *via* noncovalent interaction through their CRDs; tandem-repeat galectins (Gal-4, -6, -8, -9, -12) comprise two distinct CRDs in their *N*- and *C*-termini that are tethered by a linker of variable length (5–50 amino acids), and the chimera galectin Gal-3 has a CRD at the C-terminus and a short non-lectin peptide motif at the N-terminus, rich in proline, glycine, and tyrosine residues through which it can form oligomers.

Galectins play various roles in diverse biological processes, such as cell–cell adhesion,^228^ cell signalling,^229^ regulation of apoptosis^230,231^ or cellular activation and mitosis.^75,232^ In addition, some galectins are involved in pathological processes, such as inflammation,^233–235^ tumour progression,^236–239^ and cancer cell migration.^240,241^ The ability of galectins to modulate different events in tumorigenesis and metastasis makes them attractive targets for cancer therapy.^242^ Two Gal-3 inhibitors are currently in clinical trials: Olitigaltin (55) is undergoing a Phase IIb trial for idiopathic pulmonary fibrosis and GB1211 (14) Phase I/IIa trials for non-alcoholic steatohepatitis (NASH). In addition, Gal-1 inhibitor OTX008 (62) completed a Phase I trial as inhibitor of angiogenesis.

**Fig. 9:**
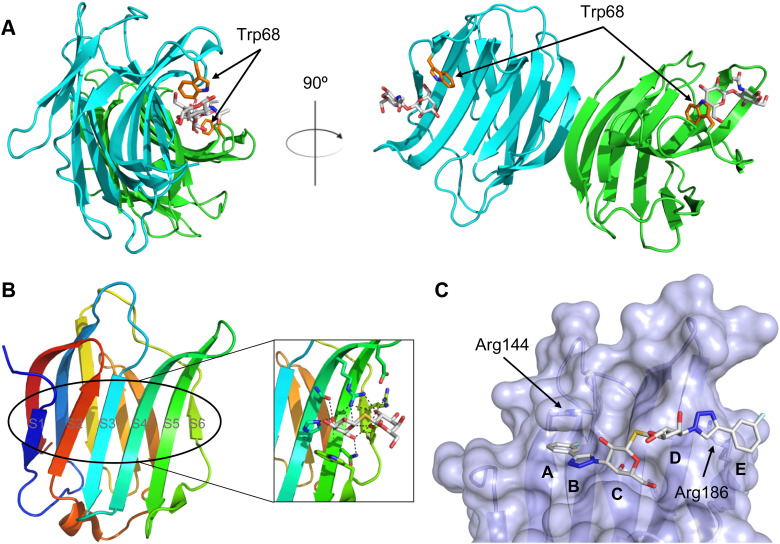
Galectin-1 and -3. (A) Gal-1 in complex with LacNAc (PDB code 4XBL).^244^ The essential tryptophan of the carbohydrate binding site is shown as stick. (B) Gal-3 CRD and in complex with a thiodigalactoside (PDB code 4JC1) (see lit.^245^). (C) Complex of Gal-3 with TD139 (55, PDB code 5H9P). While the carbohydrate core of TD139 interacts with the subsite C and D as seen for native sugar ligands, the hydrophobic substituents interact favourably with neighbouring subsites, increasing affinity and specificity.

A number of crystal structures of various galectins in complexes with natural glycan ligands are known, *e.g.* complexes of lactose with Gal-1 (PDB code 3M2M), Gal-2 (PDB code 5DG2), Gal-3 (PDB code 3ZSJ), Gal-4N (PDB code 5DUV), Gal-4C (PDB code 4YM3), Gal-8N (PDB code 2YXS), Gal-9N (PDB code 3LSE) and many others. In these structures, the galectin CRD is arranged in a slightly bent β-sandwich. Its concave side comprises six- and the convex side five-stranded antiparallel β-sheets and the carbohydrate is bound to the concave side.^243^ In most dimeric proteins, such as Gal-1 and -2, the subunits are related by a two-fold rotational axis perpendicular to the plane of the β-sheets. The glycan binding sites are located at opposite ends of the dimer (Fig. 9A).

The galectin CRDs can be formally divided into five subsites A–E (Fig. 9C).^243^ Subsite C is the galactose binding site and the neighbouring subsite D accommodates a further carbohydrate moiety, for example *N*-acetylglucosamine (GlcNAc) in the case of LacNAc. The binding of a galactose residue in site C is the most conserved feature of galectin binding, whereby six out of eight amino acids interact with the Gal residue *via* hydrogen bonds. In addition, van der Waals interactions through ring stacking between Gal and a highly conserved Trp residue are key interactions in galectin–ligand recognition and are characteristic for the galectin family.

Because the CRD of mammalian galectins is highly conserved, with only minor variations between members of the family, designing selective glycomimetics is particularly challenging. A common practice is to introduce new moieties into a carbohydrate scaffold to extend the interactions to other sites (B or E), thereby increasing affinity and selectivity (Fig. 9C).^246^ The interaction of galectins with β-galactosides involves a CH–π stacking of the galactose α-face with a conserved Trp moiety and hydrogen-bonding interactions through its 4 and 6 OH groups. Therefore, positions 1, 2 and 3 are available for further modification.

Considering the numerous functions of galectins, it comes as no surprise that these proteins have become prominent targets in drug discovery. Among the 16 known galectins, Gal-1 and Gal-3 have been studied most.^247–249^ In recent years, Gal-9 and Gal-8 have also gained attention, since inhibitors of these galectins have the potential for the treatment of cancer and fibrosis.

##### 3.1.2.1 Galectin-3

In the galectin family, the pro-inflammatory Gal-3 is unique in its structure as the only chimeric galectin in humans, which is typically present as a monomer in solution, but assembles into a pentamer in the presence of multivalent ligands, in a process mediated through the N-terminal domain.^250^ At low concentrations, Gal-3 is present as monomer and inhibits adhesion, but at high concentrations it forms large complexes that promote adhesion. This makes Gal-3 an attractive therapeutic target. In particular, there is a tight correlation between Gal-3 expression levels and various types of fibrosis. Gal-3 is a key mediator of transforming growth factor β (TGF-β), a central mediator of fibrogenesis, through which Gal-3 plays an important role in the fibrosis in numerous organs, such as liver,^251^ kidney,^252^ and lungs.^253,254^ Gal-3 is thus involved in non-alcoholic fatty liver disease (NAFLD), non-alcoholic steatohepatitis (NASH), and cirrhosis. Moreover, Gal-3 is overexpressed in many tumours and supports tumour proliferation and metastasis. Since many cancers use Gal-3 to avoid immune recognition, the inhibition of Gal-3 is considered supportive in restoring the immune system's ability to fight cancer.^226^

With respect to pharmacological intervention, Gal-3 inhibitors can be divided into three categories: peptide-derived inhibitors, carbohydrate-derived multivalent inhibitors and carbohydrate-derived monovalent inhibitors. The first two categories are beyond the scope of this review and have been reviewed elsewhere.^247^ Here, we only mention representative compounds from both categories and concentrate on the third category, monovalent glycomimetic antagonists.

###### Peptides and proteins

G3-C12 is a Gal-3 binding peptide with an outstanding affinity (*K*_D_ = 88 nM).^255^ Its i.v. administration significantly reduced metastatic cell deposition in several mice models.^256^ It is also being studied as targeting molecule to deliver peptide–polymer–drug conjugates to cancer cells.^257–259^

###### Polysaccharide-based multivalent inhibitors

Some of the earliest Gal-3 inhibitors are based on the structure of pectin, a complex plant polysaccharide rich in anhydrogalacturonic acid, galactose, and arabinose that binds Gal-3 in a multivalent manner.^260^

GCS-100 (developed by La Jolla) is a modified citrus pectin derivative that was evaluated in several clinical trials.^261,262^ In 2009, a Phase II safety study in patients suffering from relapsed chronic lymphocytic leukaemia was conducted (NCT00514696).^263^ A Phase IIb study (NCT02312050) conducted in 2014 in patients with chronic kidney disease caused by diabetes showed significant improvement of the kidney function.^264^ Although GCS-100 performed well in the studies, its development was discontinued, the main reason being its chemical composition complexity and unknown mechanism of action.

Belapectin (44, GR-MD-02, developed by Galectin Therapeutics) is a 50 kDa galactoarabino-rhamnogalacturonan polysaccharide^265,266^ and a good inhibitor of Gal-3 and Gal-1 (*K*_D_ = 2.8 and 8.0 μM, respectively).^245^ It is currently in clinical trials for the treatment of liver fibrosis and resulting portal hypertension; Phase II was successfully completed in 2017 (NCT02462967)^267^ and Phase IIb/III is ongoing (NCT04365868). A Phase IIa study using belapectin for the treatment of psoriasis was also conducted (NCT02407041). In parallel, belapectin is currently undergoing two clinical trials for a combination drug with immune checkpoints inhibitors for the treatment of metastatic melanoma: the anti-PD-1 mAb pembrolizumab (Phase Ib study, NCT02575404)^268^ and the anti-CTLA-4 mAb ipilimumab (Phase I, NCT02117362, was completed in 2018).

Besides pectin derivatives, β-d-(1,4)-galactomannans have also been studied for galectin inhibition. Davanat (45, GM-CT-01, invented by Pro-Pharmaceuticals, developed by Galectin Therapeutics)^269^ is a natural galactomannan with an average molecular weight of up to 60 kDa in which the polymannoside backbone is branched with galactose residues. It was developed for co-administration with 5-fluorouracil (5FU) (NCT00110721) and later on with 5FU, leucovorin (folinic acid) and bevacizumab as third- or fourth-line therapy for metastatic colorectal cancer (NCT00388700). Its development was discontinued in 2011 due to financial constraints and a decline in the use of 5FU.

###### Carbohydrate-derived monovalent inhibitors

In the initial development of carbohydrate-based monovalent Gal-3 inhibitors, chemical modifications were introduced into the structure of natural galectin ligands, such as the disaccharides lactose (Lac) and *N*-acetyllactosamine (LacNAc). The Nilsson group first introduced LacNAc derivatives bearing aryl carboxamides at C3 of galactose. The best compound from the series, amide 46, had an IC_50_ of 4.4 μM, rendering this compound 50 times more potent than LacNAc.^270^ According to the crystal structure of an inhibitor–Gal-3 complex, the increase in binding affinity resulted from interactions between the aromatic benzamido moiety and the Arg144 side chain in the binding site.^271^ This interaction is entropically favourable by the displacement of a water molecule. Based on this finding, a second generation of LacNAc derivatives carrying aromatic amides at galactose-C3 was synthesized. The most potent compound 47, bearing 3-carboxy-2-naphthamide, showed a *K*_D_ of 320 nM.

Another strategy developed by the Nilsson group was to modify galactose by the introduction of a benzamido group to position 3 and an anionic substituent to position 2 (*H*-phosphonate, benzyl phosphate, sulfate), both substituents acting as tweezers for Arg144. These compounds displayed moderate affinities for Gal-3, with sulfate being superior to the other O2 substituents (compound 48, *K*_D_ = 87 μM).^272^ In a follow-up study, the same compound was studied for affinity for other galectin receptors (*K*_D_ = 370 μM for Gal-1, 1000 μM for Gal-7, 900 μM for Gal-8N, and 370 μM for Gal-9N, the N-terminal domain of Gal-9).^273^ Although this strategy proved successful for target binding, it should be noted that anionic compounds typically do not possess optimal pharmacological properties and the design of neutral prodrugs might be necessary.

Introducing a hydrophobic aromatic moiety to position 2 of LacNAc (*e.g.* compound 49)^274,275^ or Lac (*e.g.* compound 50)^276^ also led to compounds with low micromolar affinity for Gal-3. Molecular modelling showed beneficial interactions between the aromatic ester/amide moiety and arginine guanidinium groups present in both Gal-1 and Gal-3. The positively charged guanidinium and its poor solvation make it an ideal interaction partner for aromatic systems. Such arene–guanidinium interactions have been intensely studied and it was shown that they can be as strong as cation–anion interactions.^277^ Interestingly, the modification of galactose C3 with further bulky aromatic groups led to improved selectivity for Gal-3 over Gal-1 (compound 51).^275^

**Fig. 10:**
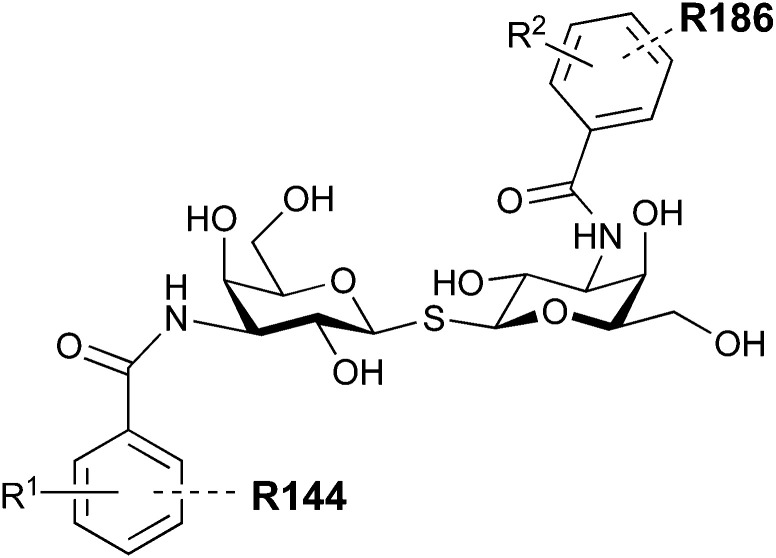
Cation–π-interaction of 3,3′-diamido-thiodigalactoside derivatives with arginine side chains of Gal-3. See also Fig. 9C where the positions of the two arginines, Arg144 and Arg186 are highlighted.

In 2005, the Nilsson group introduced a non-natural, hydrolytically stable thiodigalactoside (TDG) scaffold and its derivatives as some of the most prominent small-molecule inhibitors of Gal-3 (compound 52).^278^ The compounds are *C*_2_-symmetric and possess aromatic amides at C3 of galactose. One of these amides interacts with Arg144 and the other with Arg186, thereby further increasing the affinity for Gal-3 through double arginine–arene interactions (Fig. 9 and 10). Thiodigalactosides bind to subsites C and D, thus mimicking the interaction with lactosides while being hydrolytically more stable. Compound 53 was studied also with other members of the galectin family: while it showed a remarkable affinity for Gal-3 (*K*_D_ = 46 nM) and medium affinities for Gal-1, Gal-7 and Gal-9N (*K*_D_ = 4.7, 17, 0.9 μM, respectively), its affinity for Gal-8N was very weak (*K*_D_ > 1000 μM). This can be explained by the fact that Gal-7 and Gal-9N both contain arginine residues in both relevant positions, Gal-1 contains only one arginine corresponding to Arg186 and Gal-8N only the arginine corresponding to Arg144, while an arginine corresponding to Arg186 is missing.^117^

Next, substituents on C3 were introduced through azide–alkyne cycloaddition forming a C3-triazole with affinities comparable to aromatic C3 amides, yet easier to synthesize (*e.g.* compound 54).^279^ The compounds also showed high selectivity over other studied galectins (Gal-7, 8N, 9N). Substitution of 4-alkylcarbamoyl groups on the triazol with 4-aryl further improved affinity for Gal-3 (and Gal-1) and selectivity over other galectins (Gal-2, 4N, 4C, 7, 8N, 8C, 9N, 9C). The most potent compound from the series, compound 55, bears a 3-fluorophenyl moiety. X-Ray structural analysis of a complex of compound 55 with Gal-3, as well as a study on Gal-3 mutant, revealed that both the aryltriazolyl moieties and fluoro substituents are involved in key interactions responsible for extraordinary affinity for Gal-1 and -3. Compound 55^280^ (known initially under the code TD139 and later renamed GB0139 and Olitigaltin) has been studied as a potential drug candidate against idiopathic pulmonary fibrosis (IPF), an irreversible and ultimately fatal lung disease characterized by progressive decline in lung function. The compound is now under development by Galecto Biotech (currently called Galecto Inc., after the merge of Galecto Biotech and PharmAkea). Phase I/IIa clinical studies, administering TD139 as dry powder *via* inhalers, were conducted in 2014 and 2015 and showed that TD139 is safe and well tolerated. Furthermore, it effectively engages with Gal-3 in the alveolar space and leads to improvement in several markers of inflammation.^281,282^ A Phase IIb study with GB0139 in individuals with IPF started in February 2019 (GALACTIC-1: NCT03832946). It is also being further evaluated in a Phase IIa trial as an experimental medicine against COVID-19 (NCT04473053).^283^

One flaw of GB0139 is that it is not specific for Gal-3 and binds to Gal-1 with equal potency. Interestingly, the introduction of 4-phenoxyphenyl as a triazol substituent provided compound 56 with 230-fold higher affinity for Gal-3 over Gal-1, though the affinity for Gal-3 was 25 times lower than that of compound 55.^284^

Since its discovery, the TDG scaffold has been further decorated with a number of substituents to increase affinity and selectivity for Gal-3 or other members of the galectin family. The modifications studied and their effects on affinity and selectivity have recently been reviewed elsewhere.^245,285^

**Fig. 11:**
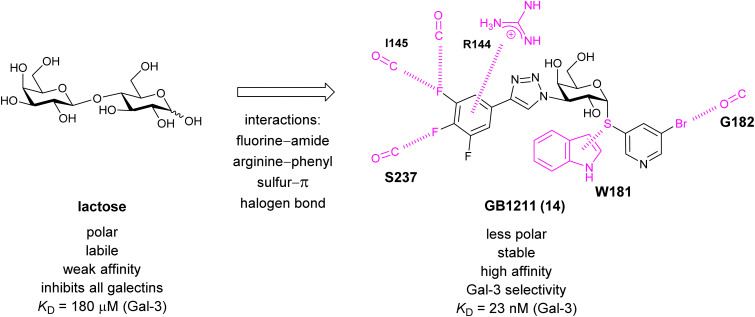
Comparison of lactose and GB1211 (14) and depiction of the Gal-3–GB1211 interactions contributing to its high affinity.

A different high-affinity scaffold for Gal-3 antagonists is based on α-d-galactopyranoside as a monosaccharide in compound 14.^286^ Its high affinity and selectivity for Gal-3 stems from the utilization of several types of non-covalent interactions: fluorine–amide, phenyl–arginine, sulfur–π, and halogen bonds, spread across subsites B, C and D (Fig. 9 and 11). Compound 14 (known as GB1211, introduced by Galecto Biotech)^287^ is a substituted pyridinyl α-d-thiogalactopyranoside which demonstrated an anti-cancer effect and antifibrotic activity in multiple preclinical models.^288,289^ GB1211 is now tested as an orally available drug candidate for non-alcoholic steatohepatitis (NASH), characterized by inflammation of the liver with concurrent fat accumulation, leading to liver cirrhosis. In a Phase I trial, GB1211 was well-tolerated and showed dose-dependent pharmacokinetics (NCT03809052). A Phase I/IIa clinical trial (GULLIVER-2: NCT05009680) was initiated in September 2021, with expected end in April 2023. In 2021, Galecto Biotech announced entering an agreement with Roche for a Phase IIa trial of GB1211 in combination with a PD-1/-L1 checkpoint inhibitor atezolizumab for the treatment of cancer (Tecentriq, start in 2022, GALLANT-1: NCT05240131).^290^

Recently, Bristol Myers Squibb reported novel triazolyl-substituted monosaccharide derivatives, represented by 57, that were identified through molecular modelling and optimized by an in-depth SAR study.^291^ The binding mode for these Gal-3 ligands was suggested by molecular modelling and experimentally confirmed in an X-ray co-crystal structure with human Gal-3 (PDB code 7XFA). The compounds bind to both mouse and human Gal-3 and show high selectivity for Gal-3 over Gal-1 and Gal-9. The affinity of 57 is comparable to that of 55 and the compound also has high oral bioavailability. For an overview of the above-mentioned Gal-3 inhibitors and their binding affinities, see Table 1.

**Table 1:** Selected Gal-3 inhibitors and their binding affinities to galectins

No	Name	Structure	Binding studies	Ref.
44	GR-MD-02	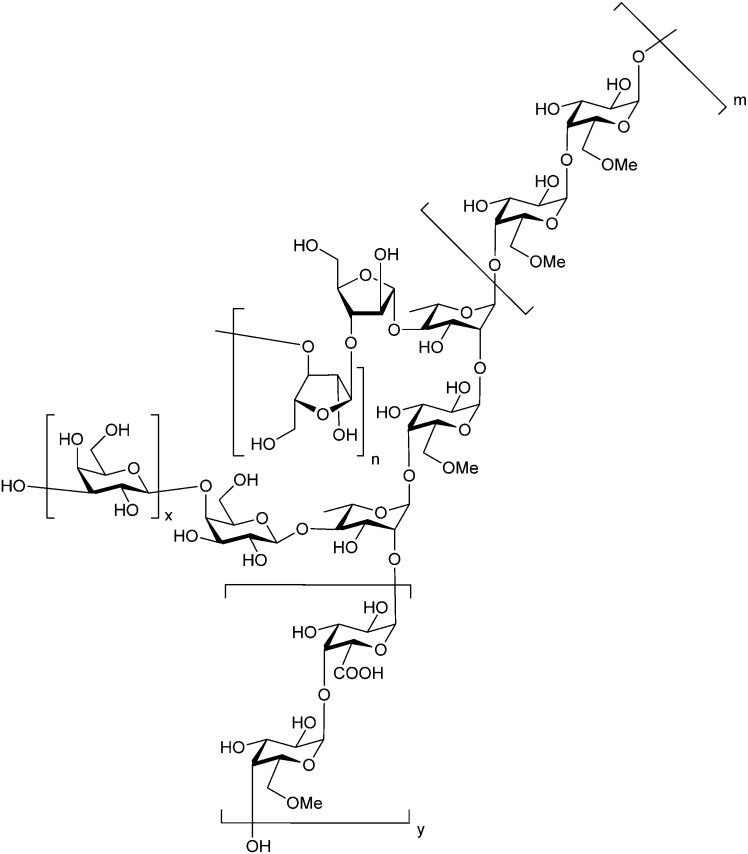	NMR *K*_D_ = 8.0 μM (Gal-1) *K*_D_ = 2.8 μM (Gal-3)	Chan *et al.*^245^
45	DAVANAT GM-CT-01	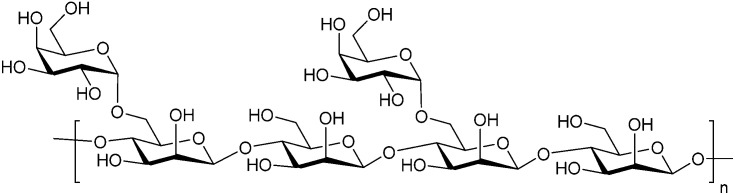	NMR *K*_D_ = 10.0 μM (Gal-1) *K*_D_ = 2.9 μM (Gal-3)	Chan *et al.*^245^
46		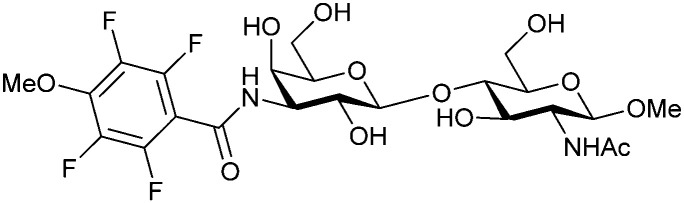	ELISA IC_50_ = 4.4 μM	Sörme *et al.*^116^
47		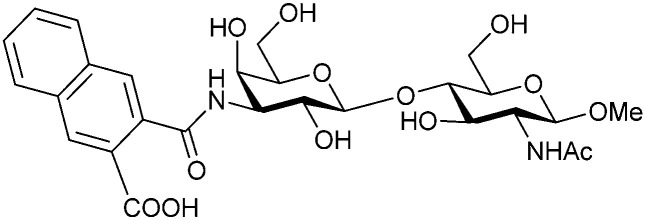	Competitive fluorescence polarization assay *K*_D_ = 320 nM	Sörme *et al.*^271^
48		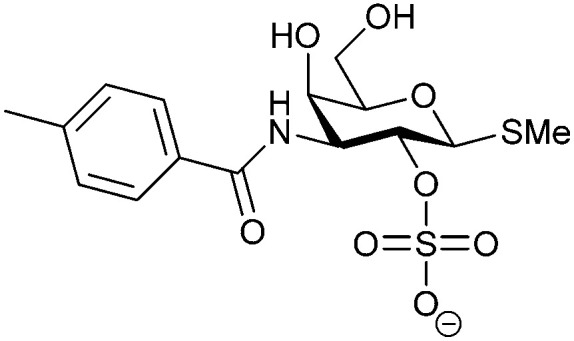	Competitive fluorescence polarization assay *K*_D_ = 370 μM (Gal-1) *K*_D_ = 87 μM (Gal-3)	Öberg *et al.*^272^
49		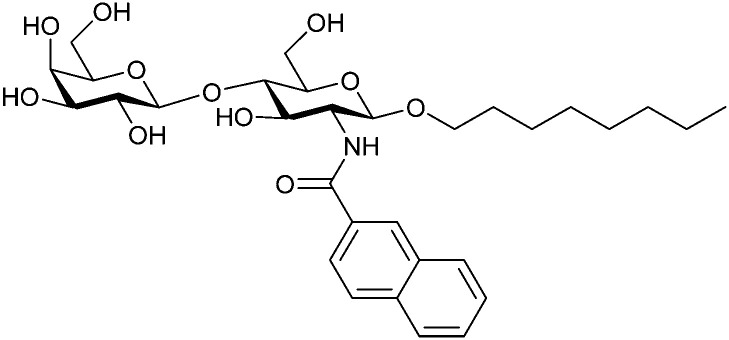	Frontal affinity chromatography/mass spectrometry *K*_D_ = 10.6 μM	Hindsgaul *et al.*^274^
50		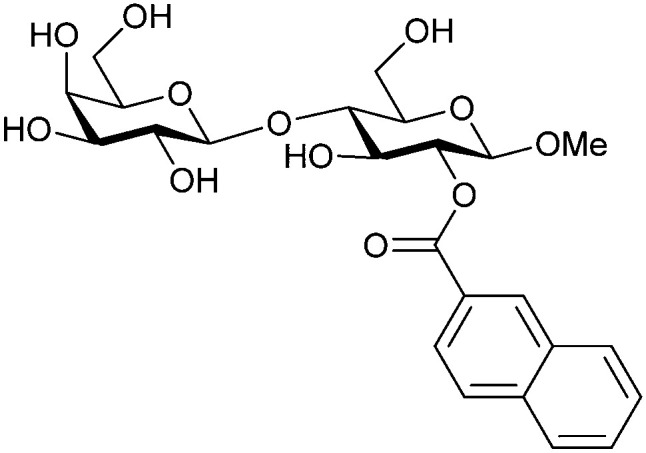	Competitive fluorescence polarization assay *K*_D_ = 14 μM (Gal-1) *K*_D_ = 2.5 μM (Gal-3)	Cumpstey *et al.*^276^
51		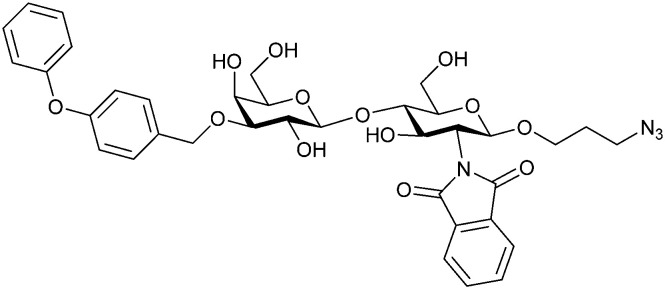	Competitive fluorescence polarization assay *K*_D_ = 280 μM (Gal-1) *K*_D_ = 1.2 μM (Gal-3)	Pieters *et al.*^275^
52		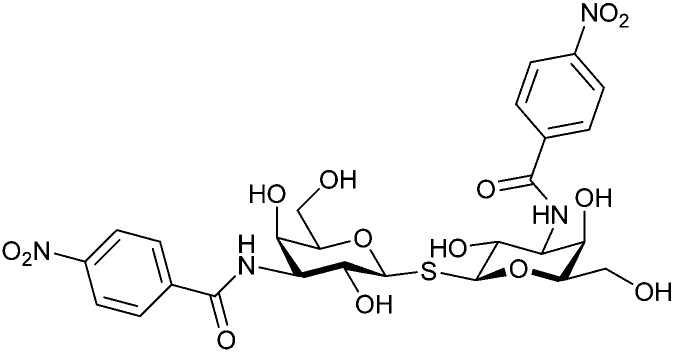	Competitive fluorescence polarization assay *K*_D_ = 33 nM	Cumpstey *et al.*^278^
53		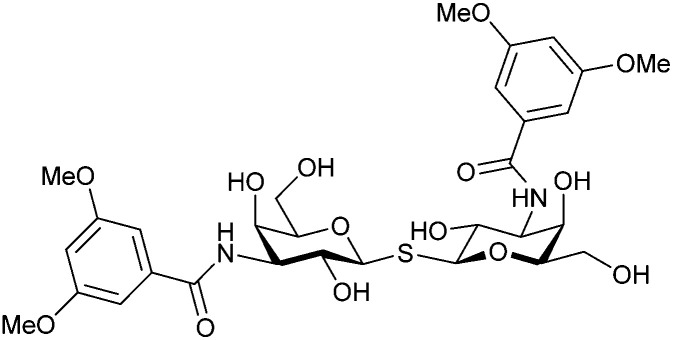	Competitive fluorescence polarization assay *K*_D_ = 4.7 μM (Gal-1) *K*_D_ = 46 nM (Gal-3) *K*_D_ = 17 μM (Gal-7) *K*_D_ high (Gal-8N) *K*_D_ = 0.9 μM (Gal-9N)	Cumpstey *et al.*^117^
54		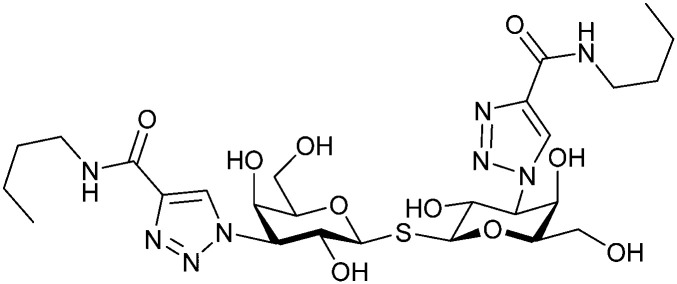	Competitive fluorescence polarization assay *K*_D_ = 29 nM (Gal-3) *K*_D_ = 5.4 μM (Gal-7) *K*_D_ = 58 μM (Gal-8N) *K*_D_ = 1.1 μM (Gal-9N)	Salameh *et al.*^279^
55	TD139, GB0139, Olitigaltin	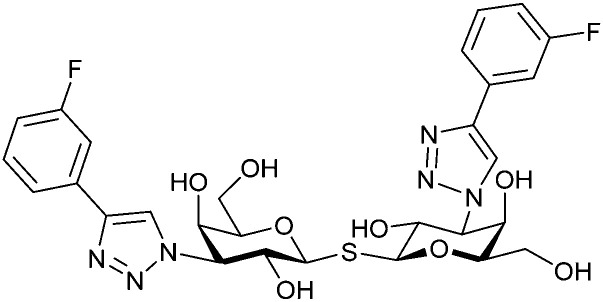	Competitive fluorescence polarization assay *K*_D_ = 12 nM (Gal-1) *K*_D_ > 5 μM (Gal-2) *K*_D_ = 14 nM (Gal-3) *K*_D_ = 0.17 μM (Gal-4N) *K*_D_ = 0.14 μM (Gal-4C) *K*_D_ = 1.9 μM (Gal-7) *K*_D_ = 86 μM (Gal-8N) *K*_D_ = 0.68 μM (Gal-9N) *K*_D_ = 0.12 μM (Gal-9C)	Delaine *et al.*^292^
56		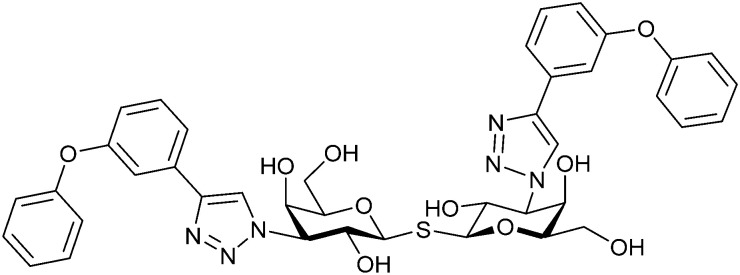	Competitive fluorescence polarization assay *K*_D_ = 84 μM (Gal-1) *K*_D_ = 0.36 μM (Gal-3)	Pieters *et al.*^275^
14	GB1211	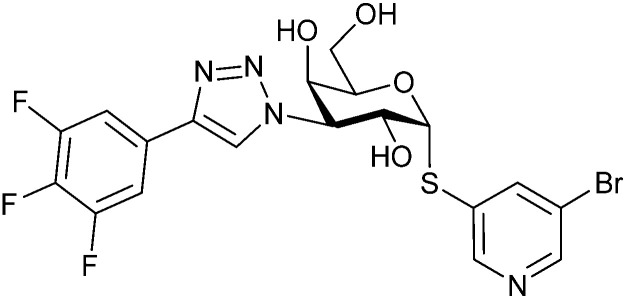	Competitive fluorescence polarization assay *K*_D_ = 23 nM (Gal-3)	Brimert *et al.*^288^
57		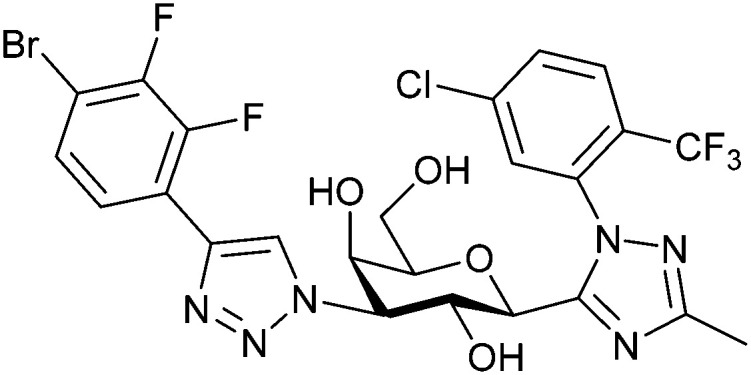	Fluorescence binding assay IC_50_ = 6.9 nM (Gal-3) IC_50_ = 2660 nM (Gal-1) IC_50_ = 2500 nM (Gal-9)	Liu *et al.*^291^

##### 3.1.2.2 Galectin-1

Gal-1 is a prototype galectin that exists as a dimer with two identical CRDs. It is involved in cell growth, differentiation, and signalling. Being highly expressed in the thymus, lymph nodes, as well as in immune cells such as T cells and activated macrophages, Gal-1 plays a key role in immune response regulation. Its expression is increased in tumour tissues as compared to normal healthy tissues.^293^ High expression of Gal-1 favours growth and progression of tumours and metastases by suppressing the immune response^242^ and by modulating cell migration, adhesion and angiogenesis.^294–296^ It also plays a key role in promoting escape from T cell-dependent immunity.^295^ Furthermore, Gal-1 can selectively induce apoptosis in activated Th1 and Th17 cells, thereby turning down the T-cell immunity, which is the basis of its anti-inflammatory activity.^230,297^ Additionally, HIV-1 exploits the host's Gal-1 to increase its attachment to host cells, thus increasing its overall infectivity in susceptible cells.^229,298–300^

Most compounds developed as Gal-3 inhibitors also show significant affinity for Gal-1 (see Table 1). Although selective inhibition of Gal-1 was not the prime focus of the above-mentioned studies, the reported data suggest that thiodigalactoside, modified pectins and galactomannans all inhibit Gal-1. Introducing an aromatic heterocycle to position 4 of C3-triazolo-thiogalactosides and triazolo-thiodigalactosides provided compounds with single-digit nM Gal-1 affinity and almost 10-fold Gal-1 selectivity over Gal-3 (compound 58, Table 2). X-Ray crystallography of the complex showed that the heterocycle is positioned deeper in a pocket between Ser29 and Asp123 of Gal-1 than the six-membered phenyl ring in the non-selective Gal-1/Gal-3 ligands.^301^ Various other carbohydrate-based scaffolds have been studied, out of which 3-deoxy-3-methyl-gulosides^302^ and aryltriazolylmethyl *C*-galactopyranosides^303^ act as selective Gal-1 inhibitors.

**Table 2:** Gal-1 inhibitors and their binding studies

No	Name	Structure	Binding studies	Ref.
58		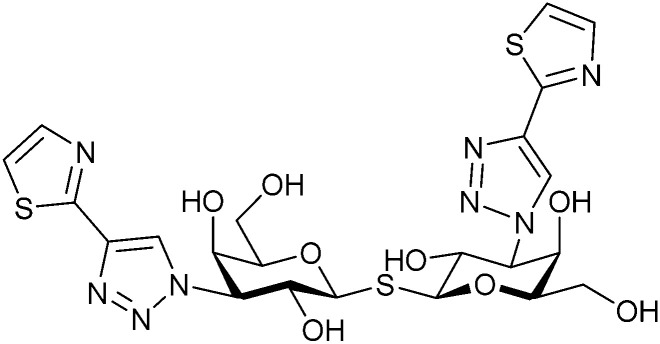	Competitive fluorescence polarisation assay *K*_D_ = 6.1 nM (Gal-1) *K*_D_ = 59 nM (Gal-3)	Peterson *et al.*^301^
59	Anginex	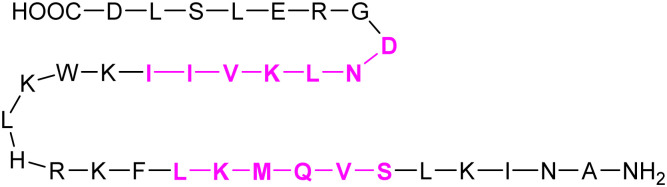	Competitive fluorescence polarisation assay *K*_D_ = 25 nM (Gal-1)	Griffionen *et al.*^305^
60	6DBF7	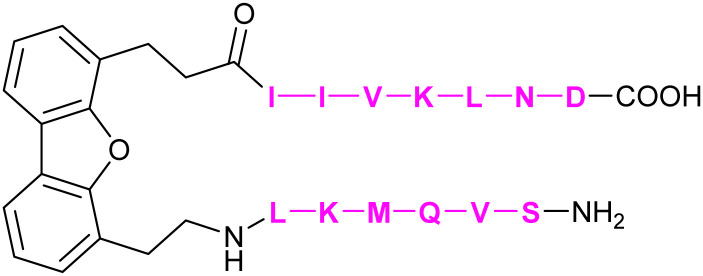		Mayo *et al.*^308^
61	DB16	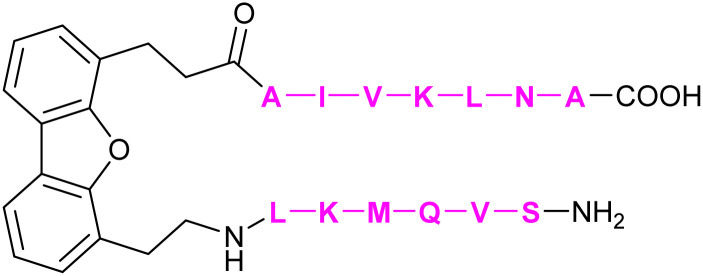		Dings *et al.*^128^
62	KM0118 OTX008	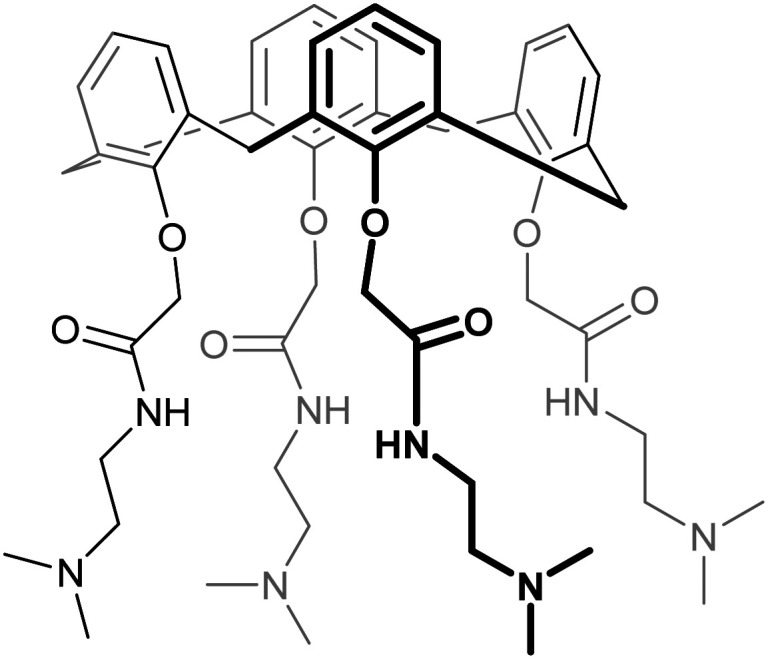		Dings *et al.*^309^

In addition to the carbohydrate-based glycomimetics mentioned above, peptides and peptidomimetics have been intensely studied as selective Gal-1 inhibitors to block angiogenesis. Anginex (59) is a 33-mer synthetic peptide that was originally designed to resemble the β-sheet structure of antiangiogenic proteins such as platelet factor 4 (PF4) and interleukin (IL)-8.^304–306^ Apart from Gal-1, 59 was reported to bind to some other galectins, namely Gal-2, -7, -8N, -9N, but not to Gal-3, -4, and -9C.^307^ Compounds 6DBF7 (60) and DB16 (61) are partial peptidomimetics of Anginex and have six amino acid residues at the N-terminus and seven at the C-terminus linked by a hydrophobic dibenzofuran (DBF) scaffold to achieve a β-sheet peptide conformation. When compared to Anginex, the compounds exhibit better angiostatic properties.^308^ However, Anginex and its partial peptidomimetics are peptides and prone to hydrolysis by proteases. In order to overcome this metabolic instability and maintain the hydrophobic and hydrophilic faces of the molecule, a non-peptidic topomimetic OTX008 (Calixarene 0118, PTX008, 62), was designed.^309^^1^H–^15^N HSQC NMR spectroscopy studies showed that the compound binds at a site different from the CBS and thus acts as a non-competitive allosteric inhibitor of Gal-1 (see Section 3.3.2).^310^ The compound downregulated cancer cell proliferation, invasion, and tumour angiogenesis in a variety of tumour cells.^311^ In addition, it has also shown synergistic effects with the tyrosine kinase inhibitor sunitinib in human ovarian carcinoma and glioblastoma.^312^ In May 2013, OTX008 was successfully evaluated in a Phase I clinical trial (OncoEthix, NCT01724320).^313^ In 2014, OncoEthix was acquired by Merck & Co. and since then, no further clinical studies have been performed according to clinicaltrials.gov. For an overview of the above-mentioned Gal-1 inhibitors and their binding affinities, see Table 2.

##### 3.1.2.3 Galectin-9

**Fig. 12:**
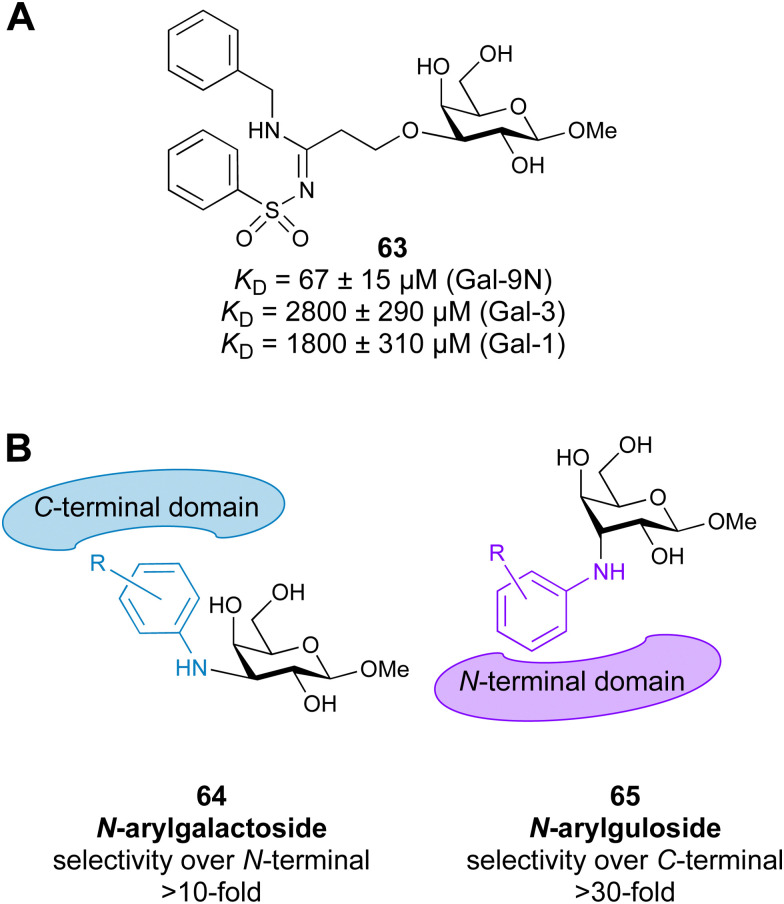
Gal-9 inhibitors. (A) Galactoside-*N*-sulfonyl amidine, (B) selectivity switch between 3-*N*-aryl galactosides and 3-*N*-aryl gulosides.

The tandem-repeat galectin Gal-9 has N- and C-terminal carbohydrate-binding domains connected by a peptide link. Gal-9 interacts with programmed cell death protein 1 (PD-1, CD279) and T cell immunoglobulin and mucin-domain containing-3 (TIM-3). While PD-1 is an immune checkpoint that promotes apoptosis of antigen-specific T-cells in lymph nodes and reduces apoptosis in regulatory T cells, TIM-3 is a T cell checkpoint inhibitor. TIM-3 expression on T cells, together with other check-point molecules, in chronic infections and cancers can hinder productive immune responses. The interaction of Gal-9 with PD-1 and TIM-3 regulates T cell death, making these promising targets for cancer immunotherapy.^314^ The activity of the TIM-3/Gal-9 checkpoint can be modulated in two ways: (i) blockage of TIM-3 with monoclonal antibodies or small molecules and (ii) blockage of Gal-9.^315^ Three monoclonal antibodies to TIM-3 are already in clinical trials for the treatment of solid tumours. Each of the programmes is evaluating the safety and efficacy either as monotherapy or in combination with anti-PD-1 antibodies in patients with advanced solid tumours or hematologic malignancies.^228^ In 2016, the Nilsson group reported galactoside-*N*-sulfonyl amidines (*e.g.* compound 63) with good affinities and selectivity towards Gal-9N over other galectins (Fig. 12A).^316^ Later on, they showed an interesting epimer switch of Gal-9 domain selectivity: while 3-*N*-aryl galactosides 64 bind the C-terminal domain, the respective gulosides 65 bind the N-terminal domain (Fig. 12B).^317^

##### 3.1.2.4 Galectin-8

**Fig. 13:**
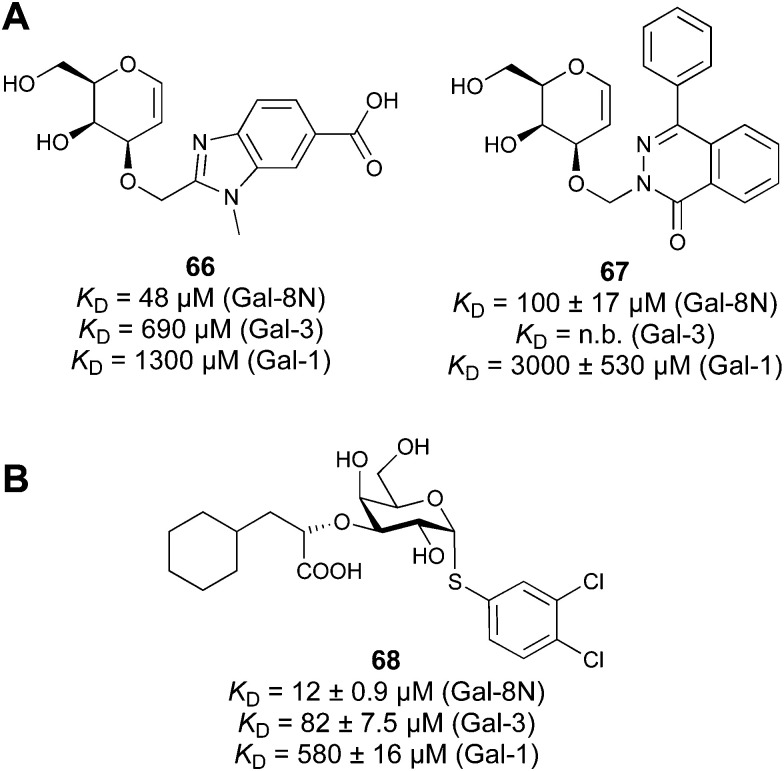
Gal-8N inhibitors and their binding affinities for Gal-8N, Gal-3 and Gal-1, highlighting the selectivity for Gal-8N. (A) d-Galactals, (B) 3-lactoyl-α-d-thiogalactopyranoside. *n.b.* = no binding.

Gal-8 is a tandem repeat galectin consisting of two CRDs, one N-terminal and the other C-terminal. These two domains share 35% sequence identity and bind differently to the natural ligands. Recently, Gal-8 has emerged as a potential pharmacological target for the treatment of various diseases, including cancer^318^ and inflammation.^319,320^ Unique functions of Gal-8 have been linked to the specificity of its N-terminal CRD. Therefore, there is a high demand for selective and high-affinity Gal-8N ligands that could potentially have anti-tumour and anti-inflammatory properties. To date, several galactose-based Gal-8N inhibitors have been reported, but mostly suffer from poor selectivity.^321–324^ Recently, d-galactal derivatives (*e.g.* compounds 66 and 67, Fig. 13A) have shown not only high affinity, but also good selectivity over other galectins.^325,326^ Other recently developed antagonists include 3-lactoyl-α-d-thiogalactopyranosides (*e.g.* compound 68, Fig. 13B).^327^

#### 3.1.3 Siglecs

Sialic acid-binding immunoglobulin-type lectins (Siglecs) are a large subfamily of the immunoglobulin superfamily cell surface receptors that share interaction with sialic acids. Usually, these nine carbon sugars are present as terminal monosaccharides on *N*- and *O*-glycans and glycolipids.^328–330^ The majority of the members of the Siglec family are expressed on immune cells with Siglec-4 being an exception. On these cells, they orchestrate important immune cell functions by activation or inhibition of primary immune signals.^329–331^ As key regulators of self/non-self recognition, various Siglecs are found central to innate and adaptive immune cell functions and are involved in inflammation, cancer, autoimmunity, and infectious diseases.^330^

All Siglecs share a similar overall protein architecture. An N-terminal V-Ig like domain harbours the ligand binding site for sialic acid recognition. This domain is extended away from the cell surface by a series of one to sixteen C1 or C2-Ig like domains.^332,333^ A C-terminal intracellular domain is present in some Siglecs and contains an immunoreceptor tyrosine-based inhibition motif (ITIM) for cell signalling inhibition (*i.e.* CD22). Alternatively, positively charged amino acids located in the transmembrane region enable association with Dap12 carrying an immunoreceptor tyrosine-based activation motif (ITAM) for cell signalling activation.^334^ Overall, Siglecs can be divided into two groups with classic Siglecs being more conserved among species and CD33-related Siglecs having a higher sequence variability between homologues.^329^

Individual Siglecs have a restricted expression pattern on immune cells, rendering them suitable targets for cell-specific therapies. Additionally, many, if not all, are endocytic receptors allowing the delivery of various cargos to immune cells.^329,335^ This concept was successfully followed for several Siglecs: Siglec-8 is expressed on eosinophiles and mast cells and is explored for therapies of asthma and allergies,^336^ Siglec-15 is expressed on osteoclasts and is a target for the treatment of osteoporosis.^329,337^ For myelin associated glycoprotein (MAG/Siglec-4),^333^ glycomimetics have been developed for the enhancement of neurite outgrowth, whereas a completely orthogonal approached was reported recently in which PPSGG (PN-1007, Polyneuron Pharmaceuticals), an undisclosed glycomimetic, was claimed to prevent anti-MAG IgM autoantibodies to deplete MAG expressing cells (NCT04568174). CD22 (Siglec-2) is highly expressed on B cells and has been successfully used as a target for the treatment of B cell lymphoma. The anti-CD22 antibody drug conjugate (ADC) inotuzumab ozogamicin (Besponsa, Pfizer/UCB) is used for the treatment of acute lymphoblastic leukaemia.^338^ The anti-CD33 (Siglec-3) antibody gemtuzumab ozogamicin (Mylotarg, Pfizer) has been approved since 2000 for the treatment of acute myeloid leukaemia.^339^ Moreover, a CD33 glycomimetic might be an effective therapy against late-onset Alzheimer disease (AD), which increases the uptake of the toxic amyloid-β (Aβ) peptide into microglial cells and thus might promote clearance of the Aβ peptide causing AD progression.^340^ For a summary of current antibodies against Siglecs the reader is referred to a more in-depth review.^341^

With respect to the development of glycomimetic ligands for Siglecs, one has to take into account that the sialic acid family is a large and diverse group of sugars.^342,343^*N*-Acetyl neuraminic acid (Neu5Ac) is the most common nine-carbon representative of the family and can potentially carry a large variety of substitution patterns, such as sulfation and acetylation.^328^ Additionally, Neu5Ac can be further sialylated in the 3-, 6-, 8-, and 9-position. Consequently, the Siglec protein structure provides grounds for a high level of sialic acid specificity based on the linkage of the sialic acid or substitution pattern. For carbohydrate-based glycomimetics, this translates into the basis for many design approaches starting from extended carbohydrate epitopes, up to trisaccharides to reduce off-targets within the family. From this, a significant loss of drug-likeliness and less favourable pharmacological properties arise. To the best of our knowledge, there are no reports on replacing the core sialic acid with a drug-like scaffold.

**Fig. 14:**
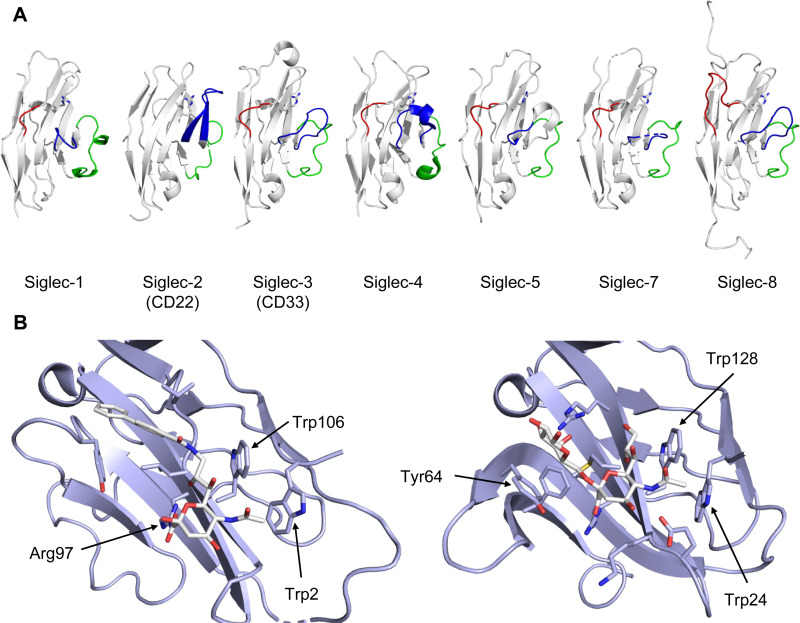
Siglec V-Ig domain fold. (A) Siglec-1, CD22 (Siglec-2), CD33 (Siglec-3), Siglec-4, Siglec-5, Siglec-7, Siglec-8, C’D loop region (green), G strand (red), CC′ loop (blue), essential arginine shown in stick (PDB codes 1QFP, 5VKM, 6D49, 5FLV, 2ZG2, 2G5R, 2N7B, respectively). (B) (left) Siglec-1 in complex with 69 showing 5’ site stacking of Trp2 and stabilization of the glycerol side chain of the Neu5Ac by Trp106.^350^ This pose leads to the extension of the anti-parallel β-sheet and is further stabilized by a salt bridge to the so-called “essential arginine” (here Arg97). The GG′ loop harbours Val109 in Siglec-1 providing affinity for the biphenyl substituent in 69 (PDB code 1ODA). (right) CD22 does not have a GG′ loop and is less restricted in this position. However, compared to Siglec-1, the role of the CC′ loop is much more pronounced. In CD22, Tyr64 located in this loop does a stacking interaction explaining the strong linkage preference of CD22 for α-2,6 linked sialic acid ligands (PDB code 5VKM).

The development of carbohydrate-based glycomimetics for Siglecs was pioneered by early work reporting on sialic acid derivatives substituted in position 9 to gain affinity for several targets.^344^ The general approach of expanding the core Neu5Ac using hydrophobic substituents in position 9 was later followed by attempts to grow this central element into C-2, -4, -5 direction and finds its justification in a number of distinct structural features of the Siglec architecture.^345–347^ Firstly, a central arginine is present in a conserved position of the antiparallel β-sheet making interactions with the carboxylate of the sialic acid (Fig. 14). Secondly, there is an interesting feature of the sialic acid recognition: the monosaccharide is essentially a peptidomimetic. It extends the anti-parallel β-sheet with its hydrogen-bond donors and acceptors mimicking the next antiparallel β-strand (Fig. 14B).^348^ Next, this GG′ strand to which the sialic acid aligns like a peptide has high structural variability amongst the Siglecs exactly when extending from position 9.^332^ The biological role of this variability is not fully described, but it can be speculated to be involved in generating specificity for substitution patterns of the 7, 8, and 9 position of the Neu5Ac core. For the carbohydrate-based glycomimetics, this implies high likelihood for gaining both affinity and specificity when extending from this position. Additionally, the same holds true for the CC′ loop which can extend towards the 9 position of the carbohydrate binding site (Fig. 14A). Interestingly, the CC′ loop can also dictate the linkage specificity as evidenced for CD22 and Siglec-7.^332,349^ Other positions that have been explored for extending the carbohydrate core such as the 5 position show lower structural variability compared to the 9 position. Overall, some Siglecs have preformed binding sites devoid of any conformational selection upon ligand binding, such as CD22 and Siglec-1, while others do have such as Siglec-4 and -7. Capitalizing on these structural features of the protein, and the advancement of sialic acid synthesis, a number of reports have shed light on carbohydrate-based glycomimetics for several Siglecs using focused library screening.^335,345–347,350–356^

An additional challenge for the development of inhibitors for many Siglecs are *cis*-ligands. These are sialosides present on the same cell and although the protein structure extends the active site away from the cell surface, Siglecs are exposed to these sialic acids, which can block the active site. These *cis*-ligands are present in high local concentrations, which are estimated to be in the 100 mM range, imposing a massive competition for potential glycomimetics.^329,357^ These ligands can even be present on the same receptor, leading to the formation of homooligomeric receptor assemblies on the cell, *e.g.* CD22.^358^ Taken together, the vast majority of glycomimetics for Siglecs are carbohydrate-based extending of a limited set of substitution vectors benefiting from the local site variability to gain specificity and lipophilicity to gain affinity. However, none of these molecules has made it into the clinics or as monovalent chemical probe into *in vivo* preclinical development, likely because of the high affinity necessary. For a more detailed summary of small molecule inhibitors of Siglecs the reader is also referred to other excellent reviews.^335,341,355^

##### 3.1.3.1 Siglec-1

**Fig. 15:**
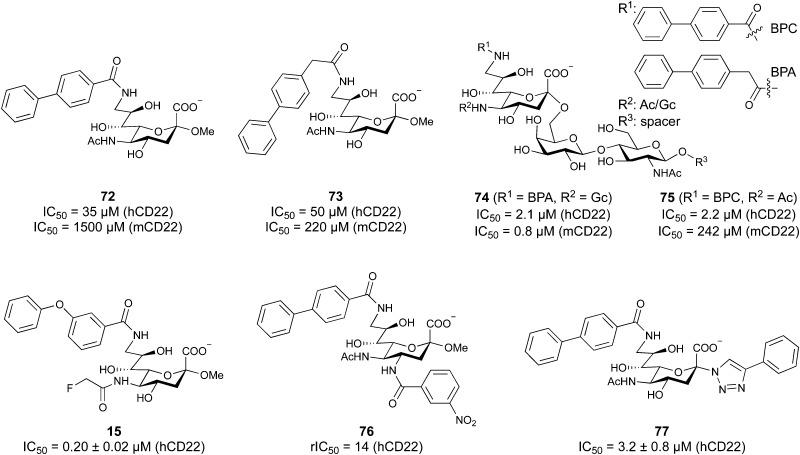
Murine and human CD22 antagonists. A selectivity gain towards human CD22 (hCD22) over murine CD22 (mCD22) is achieved by BPC substitution of the 9’ position and acetylation of position 5. C4 modification resulted in further improved affinity. 76 here is reported in relative IC_50_ compared to 72 (IC_50_ = 3 μM) in the corresponding assay setup with rIC_50_ = IC_50_ (72)/IC_50_ (76).^345^

Siglec-1 (sialoadhesin, CD169) is expressed on macrophages and is the largest member of the Siglec family, with 17 Ig repeats extending this protein far into the extracellular space. It was the first Siglec for which the X-ray structure was solved, teaching us many of the above-mentioned structural characteristics of the recognition process: the essential arginine, the extension of the anti-parallel beta sheet.^359^ Later, the complex between Siglec-1 and 9-*N*-BPC-Neu5Acα2OMe (69, Table 3) was solved by X-ray crystallography and the affinity gain through interaction with the GG′ loop became apparent (Fig. 14B).^350^ The biphenyl substituent results in a 13-fold affinity increase over the Neu5Ac monosaccharide.^350,360^ A clear specificity gain was seen when included in the trisaccharide structure 9-*N*-BPC-Neu5Acα(2,3)Galβ(1,4)GlcNAc (70) compared to the 9-*N*-BPC-Neu5Acα(2,6)Galβ(1,4)GlcNAc (75, Fig. 15) being a CD22 ligand provided by the α(2,3) over the α(2,6) glycosidic linkage.^361^ However, used in a multivalent display for targeted delivery on liposomes *in vivo* the compound failed because of insufficient specificity.^362^ Following up on this work, using rational design and virtual screening a focused library of substituents in the 9 position, a thienochromene substituent was identified (71, Table 3) leading to more than one order of magnitude affinity increase and importantly sufficient specificity to be applied in mice as part of a liposomal formation.^353^ This affinity and specificity increase can likely be rationalized by the higher shape complementarity of the thienochromene group extending into the GG′ region. This *in vivo* specificity was later used for the delivery of αGal ceramide loaded lipid nanoparticles to Siglec-1 expressing macrophages and activation of iNKT cells through CD1d.^363^ Furthermore, the model antigen ovalbumin (OVA) was delivered to Siglec-1^+^ macrophages.^364^

**Table 3:** Siglec-1 inhibitors and their binding affinities

No	Structure	Binding affinity	Ref.
69	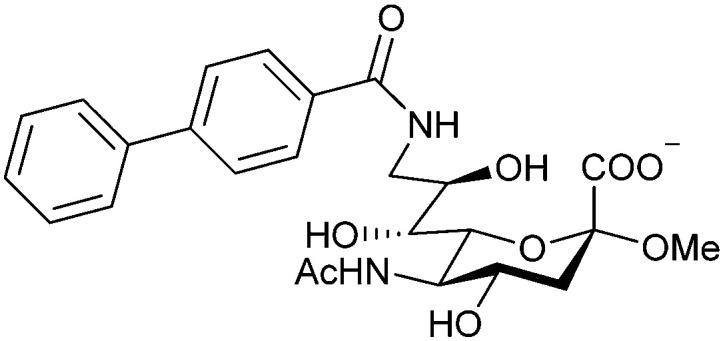	Not reported	Zaccai *et al.*^350^
70	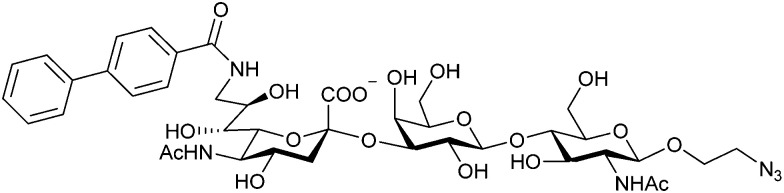	IC_50_ = 4.82 ± 0.14 μM	Nycholat *et al.*^353^
71	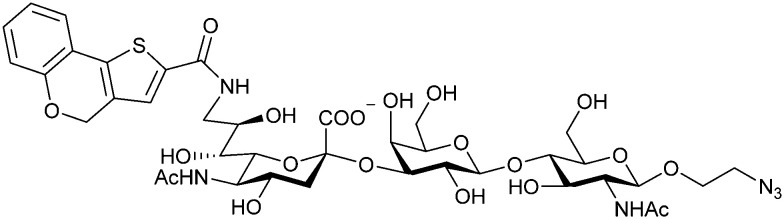	IC_50_ = 0.38 ± 0.04 μM	Nycholat *et al.*^353^

##### 3.1.3.2 Siglec-2/CD22

CD22 (Siglec-2) is a 140 kDa single transmembrane protein carrying six C Ig-like domains and one V-Ig domain that extends CD22 like a tilted rod into the extracellular space.^332^ This inhibitory immune cell receptor is highly expressed on B cells, with reported low expression on mast cells and conventional dendritic cells.^365^ This restricted expression pattern has advanced CD22 as an attractive target for immune therapy, early validated using anti-CD22 antibodies.^338^

Major advancement in the development of carbohydrate-based glycomimetics targeting CD22 came from early work of Kelm *et al.*, and later was followed by Paulson and co-workers reporting a 224-fold increase in affinity for 9-BPC-Neu5Acα2Me (72) for the human CD22 and a 123-fold increase for 9-BPA-Neu5Acα2Me (73) for the murine homolog, compared to their unsubstituted parent monosaccharides, respectively (Fig. 15).^344,345,366^ Since the murine CD22 has a strong preference for *N*-glycolyl neuraminic acid (Neu5Gc), including this moiety in the design led to an over 250-fold potency gain when combined with this 9’ substitution in the framework of the trisaccharide 74 compared to 75.^351^ Compared to the α(2-6)-sialyllactose core scaffold, with an affinity of 281 ± 10 μM as assessed by isothermal titration calorimetry (ITC), this 9-BPA-NeuGcα(2-6)Galβ(1-4)GlcNAcβ-spacer (74) achieved more than three orders increase in affinity.^332,351^ Taken together, this advancement in the 5 and 9 positions provided sufficient affinity and specificity to target murine CD22 using multivalent display on nanoparticles *in vivo*.^367,368^ For the human homolog, a suitable ligand with sufficient affinity and specificity came from focused library screening of the 9 position, being 6‘MBP-5F-Neu5Ac (15).^115^ Although CD22 glycomimetic recognition is mediated by a largely preformed binding site, NMR analysis highlights that the role of the biphenyl substituent is more complex and provides room for future improvement.^332,369^

Besides advancement in the 5 and 9 position, further improvement came from a C-4 modified structure-based design of 9-BPC-4-*m*NPC-Neu5Acα2Me (76) with a sub-micromolar affinity or C-2 modifications (77).^345,346^

To the best of our knowledge the only non-carbohydrate CD22 ligand is the peptide PV3, which was derived from epratuzumab Fab and binds CD22 with 9 μM affinity.^370^ The peptide binding site is unrelated to the canonical carbohydrate binding site and does not have to overcome the *cis*-ligand challenge. Applied as a multivalent probe for targeted delivery on a liposome, it allows for *in vivo* delivery when high receptor density is available.^370^

#### 3.1.4 Bacterial lectins

Antimicrobial resistance is rapidly rising, and therefore, complementary new approaches are needed to fill the antibiotic pipeline.^371^ One compelling approach that seeks to circumvent resistance development is the use of pathoblockers or anti-virulence drugs. Unlike antibiotics these agents do not kill the bacteria, but instead prevent pathogenicity by interfering with virulence factors.^372,373^ To this end, bacterial lectins are attractive targets for the development of new antibiotic-sparing anti-infective drugs, some of which are already in clinical development.

One important pathogenicity factor containing lectin domains are bacterial toxins comprising Shiga toxin, present in haemorrhagic Enterobacteriaceae. In these AB_5_ toxins, the pentameric B-domain repeat is a lectin and responsible for carbohydrate-dependent binding to the cells, followed by internalisation to exert their intracellular toxicity. Due to their geometry, these AB_5_ toxins have been successfully inhibited with complementary pentavalent ligands, such as STARFISH or DAISY containing native oligosaccharide epitopes.^33,374^ However, their further development as antitoxins has stalled for unknown reasons.

Furthermore, in many bacteria, surface exposed lectins serve as adhesins for host colonization and persistence. Several lectins of the most problematic Gram-negative bacteria are therefore currently targeted with glycomimetics.

##### 3.1.4.1 *Escherichia coli* fimbrial adhesin FimH

One intensively studied lectin is FimH, an adhesin at the tip of *E. coli* type 1 fimbriae that is important for tissue binding in urinary tract infections and inflammatory bowel disease. Thus, clinical candidates that inhibit FimH are being developed for both conditions.

With about 150 million cases annually, urinary tract infections (UTIs) are among the most frequent bacterial infections.^375,376^ Notably, up to 80% of all uncomplicated UTIs are caused by uropathogenic *E. coli* (UPEC).^377,378^ UPEC utilize type 1 fimbriae to adhere to urothelial cells which line the host bladder, a pivotal step to establish an infection.^379^ Host cell adhesion allows UPEC to avoid clearance during micturition and additionally invasion of host cells is triggered. Once inside of host cells, UPEC again utilize FimH to form biofilm-like structures, called intracellular bacterial communities (IBCs), to replicate and hide from the immune system or antibiotic treatment. In the last step of the bacterial infection cycle, the bacteria disperse from the IBCs and exfoliate to invade neighbouring cells.^380^ Expression of type 1 fimbriae was not only shown to be essential for cell adhesion, but also for formation of IBCs.^381^

Furthermore, the increased prevalence of adherent-invasive *E. coli* (AIEC) in patients suffering from specific inflammatory bowel diseases, such as Crohn's disease (CD), has attracted growing attention for development of new treatment strategies.^382,383^ Like UPEC, AIEC adhere to intestinal epithelial cells *via* FimH. The chronic inflammation elicited by the intestinal dysbiosis in CD leads to tissue damage and progressing organ degradation.

Therefore, the development of high affinity FimH ligands presents an opportunity to complement antibiotic use and improve the treatment of Crohn's disease and UTIs.

**Fig. 16:**
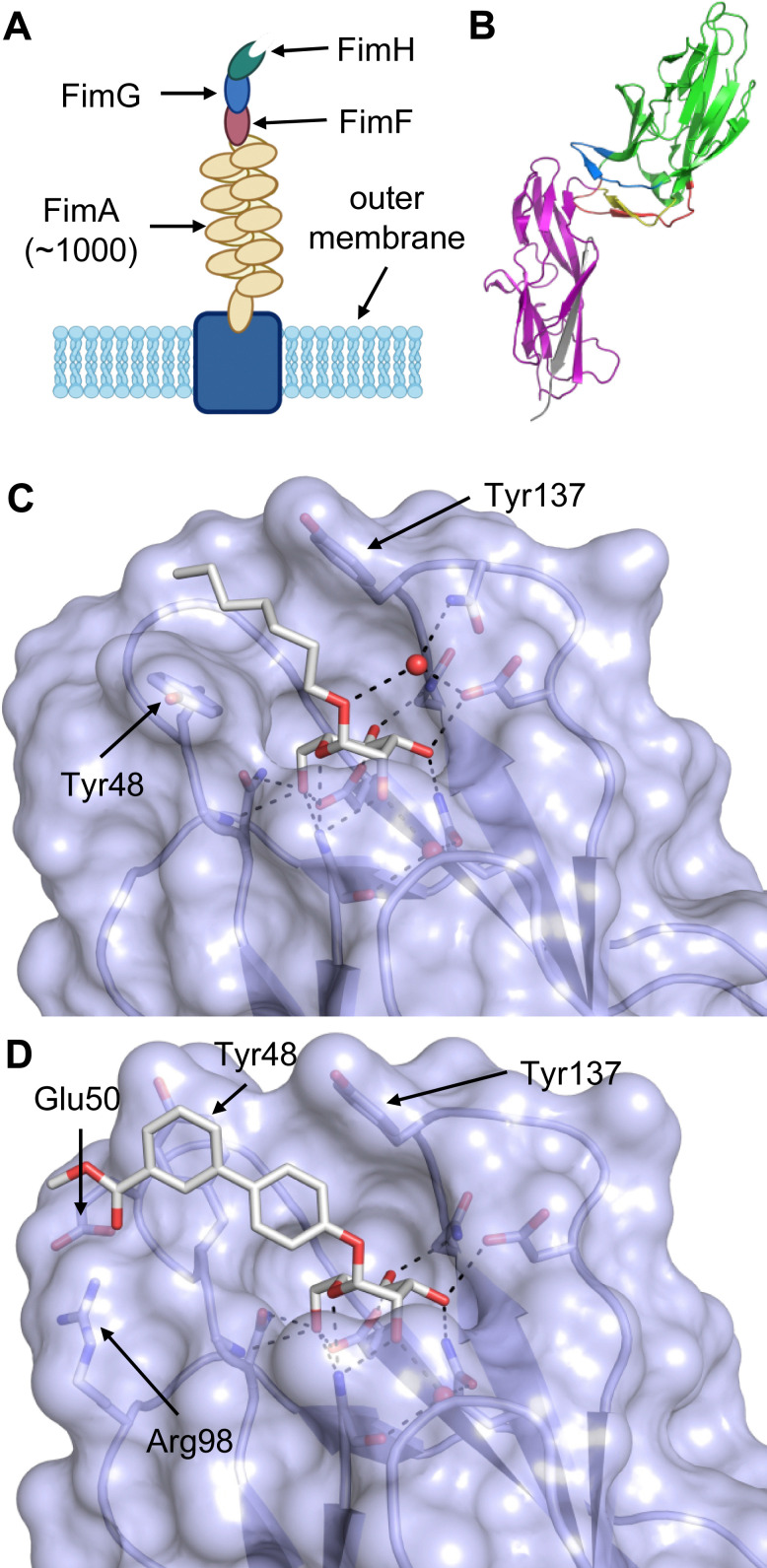
(A) Structure of a type 1 fimbrium: anchored to the membrane ∼1000 FimA subunits form a helical rod to which the mannose-binding subunit FimH is attached *via* the subunits FimF and FimG.^408^ (B) Two-domain fimbrium subunit FimH (PDB code 4XOE).^389^ The binding affinity of the carbohydrate-binding domain FimH_L_ (green) is influenced by contacts of the swing loop (blue, aa 23–33), linker loop (yellow, aa 151–158) and insertion loop (red, aa 112–125) to the fimbrial domain FimH_P_ (pink) enabling the formation of catch-bonds. Donor strand to complete the Ig-fold of FimH_P_ shown in grey. (C) Ligand heptyl α-d-mannoside (78) bound to FimH (PDB code 4XOE).^389^ The mannose moiety engages in extensive hydrogen bonding in a deep binding pocket while the alkyl chain interacts hydrophobically with Tyr48 and Tyr137 of the tyrosine gate in the in-docking mode. Water shown in red. (D) Biphenyl ester 79 in complex with FimH (PDB code 3MCY).^409^ Hydrophobic interactions of the aglycon with the tyrosine gate as well as interaction with the salt-bridge of Glu50-Arg98 significantly contribute to high affinity binding. In contrast to heptyl α-d-mannoside, 79 interacts with FimH in the out-docking mode.

Type 1 fimbriae are 0.1–2 μm long filaments on the bacterial surface.^384,385^ They consist of approximately 1000 copies of the subunit FimA, forming a right-handed helical rod, which attaches to a single FimF and FimG subunit, and is capped at the tip by the carbohydrate-binding adhesin, FimH (Fig. 16A).^384,385^ Notably, the subunits engage in a donor-strand completion mechanism, in which the incomplete immunoglobulin-fold of each subunit is completed by an N-terminal extension of the next subunit.^386,387^ Structurally, the 30 kDa protein FimH consists of two domains: the amino-terminal carbohydrate-binding FimH_L_ (aa 1–158) and the carboxyl-terminal FimH_P_ (aa 159–279), which connects FimH_L_ to the following subunit FimG and therefore to the pilus (Fig. 16B). Importantly, the domain interaction of FimH_L_ and FimH_P_ allows formation of so-called catch-bonds, characterised by an increased binding strength under shear stress, *e.g.* during micturation.^388–390^ Those catch bonds help UPEC to avoid clearance during urination, while preserving the ability to migrate and infect new cells in absence of a tractive force.^391^

The carbohydrate-binding adhesin domain of FimH_L_ is located at the distal end of a β-sheet opposite of the FimH_P_ domain. In absence of shear stress, three loops of FimH_L_ contact FimH_P_, resulting in distortion of the β-sheets, affecting the CRD. Consequently, the binding pocket widens, leading to weakened ligand interactions and therefore reduced ligand affinity and quicker ligand dissociation. In contrast, tensile force leads to domain separation of FimH_L_ and FimH_P_, enabling long-lived binding events with high affinity.^389,392^ This mechanism of allosteric regulation by domain interaction is underlined by the work of the Maier and Glockshuber groups.^389^ A 3300-fold higher affinity for *n*-heptyl α-d-mannoside (HM, 78) was reported for isolated FimH_L_ (representing the high affinity conformation) compared to full length FimH in the low affinity conformation (*K*_D_ = 3.0 ± 0.2 nM *vs.* 9900 ± 150 nM). Natural ligands of FimH are α-d-mannosides of *N*-glycans, such as the uroepithelial glycoprotein uroplakin 1a or CEACAM6 (CD66c) in the ileum.^393,394^ Importantly, the amino acids of the CRD of FimH are highly conserved among different *E. coli* isolates and many further Enterobacteriaceae, suggesting a reduced risk of resistance development.^395–398^ Studies with deletion mutants of *fim*H validated FimH as potential drug target. The *fim*H mutants lost the ability to bind to human and murine bladder cells and a lowered bacterial survival rate in murine kidney and bladder was reported.^399,400^ In addition, the therapeutic value of targeting FimH was further underlined by vaccination against FimH and use of antibodies directed against FimH, both resulting in significantly reduced colonization of murine bladder cells *in vivo*.^400,401^

**Fig. 17:**
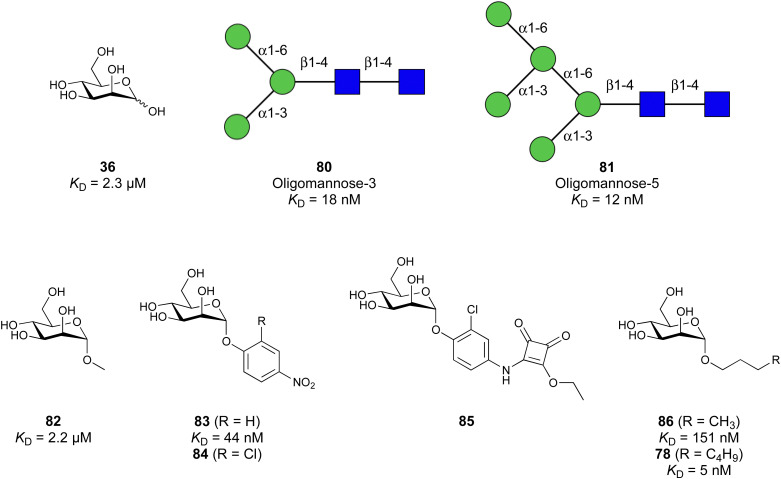
Natural occurring mannosides and early synthetic FimH ligands. Alkyl and aryl mannosides profit from additional hydrophobic interactions with the tyrosine gate surrounding the CRD compared to d-mannose (36). A small substituent in *ortho* position of the phenyl aglycon further improves affinity.

The mannose ligand is bound by FimH in a deep, negatively charged pocket formed by Asn46, Asp47, Asp54, Gln133, Asn135, Asn138 and Asp140.^395^ An extensive network of direct and water-mediated hydrogen bonds, is formed between FimH and mannose (36), resulting in an unusual high affinity (*K*_D_ = 2.3 μM, determined by SPR)^402^ for a monovalent lectin–carbohydrate interaction (Fig. 17).^395^ In this complex, every hydroxy group of mannose establishes direct hydrogen bonding except for hemiacetal O1, which engages in indirect H bonding *via* a water.^402^

α-Mannosides are bound with the aglycon pointing outwards of the binding pocket towards the protein surface, where a hydrophobic rim comprised of Phe1, Ile13 and Phe142 as well as the so-called ‘tyrosine gate’ of Tyr48, Ile52 and Tyr137 surround the entry to the deep, hydrophilic CRD.^402^ In particular, the tyrosine gate allows for differentiation between different mannose oligosaccharides and favourable interaction with the tyrosine gate is a key prerequisite for the development of high-affinity FimH inhibitors. The extraordinary affinities of the naturally occurring branched mannosides oligomannose-3 (80) and oligomannose-5 (81) (*K*_D_ = 18 nM and 12 nM, determined by SPR) highlight the possibility to achieve nanomolar binding to FimH.^403^ Notably, Tyr48 of the tyrosine gate is conformationally flexible, resulting in an ‘in-docking’ pose (*e.g.* oligomannose-3, PDB code 2VCO^404^ or HM, PDB code 4XOE, Fig. 16C) and an ‘out-docking’ mode frequently reported for synthetic FimH inhibitors (*e.g.*3MCY,^112^Fig. 16D).^405^ Replacement of the mannose moiety by different sugars such as glucose leads to a significant loss of affinity, resulting in millimolar binding.^402^ Notably, some affinity is retained for binding of fructose (*K*_D_ = 31 μM, determined by SPR).^406^ Further important features of the FimH binding pocket relevant for drug development are the Arg98–Glu50 salt bridge and a small hydrophobic pocket next to the CRD.^407^

Because of the extraordinary binding specificity of FimH for α-d-mannosides, the development of FimH antagonists mainly focused on the introduction of aglycons to mannose to improve binding by hydrophobic interactions. A notable exception to this is the report on septanoses to replace the mannose moiety, by Peczuh and Ernst *et al.* resulting in compounds with low micromolar IC_50_.^410^ Generally, the potency of compounds reported by different working groups cannot be directly compared due to a variety of different *in vitro* assays and *in vivo* models. An overview of the different *in vitro* and *in vivo* assays employed as well as a comprehensive summary of affinity data was published by Mydock-McGrane *et al.* in 2017.^407^

###### Early glycomimetics

Development of FimH inhibitors started in 1977, when methyl α-d-mannoside (MeαMan, 82) was reported as inhibitor of *E. coli* cell adhesion to human mucosal cells *in vitro* (Fig. 17).^411^ A 1979 study in mice revealed that MeαMan could prevent and reverse bacterial adhesion to bladder cells *in vivo.*^412^ Thereafter, MeαMan was used as reference for the potency of new FimH inhibitors. Sharon and Ofek, who conducted those studies, also postulated the existence of a hydrophobic region in close proximity to the CRD in 1983. In an aggregation assay of guinea pig erythrocytes with *E. coli p*-nitrophenyl α-mannoside (*p*NPαMan, 83) was found 125-fold more potent than MeαMan.^413^ Further investigation of aromatic α-mannosides by Sharon *et al.*, led to the key discovery that phenyl *ortho*-substituents improve inhibitory potency. Inhibition of adhesion of *E. coli* O128 to guinea pig ileal epithelial cells by *o*-chloro-*p*-nitrophenyl α-mannoside (84) surpassed the inhibitory activity of MeαMan by a factor of 470, and importantly, the activity of *p*NPαMan (without *ortho*-substituent) almost 7-fold.^414^ Despite these early key observations in the structure–activity relationship of FimH inhibitors no significant progress in the development of aromatic α-mannosides was made until Lindhorst and colleagues published a novel scaffold in 2006, in which a squaric acid moiety was introduced to the *para* position of the phenyl aglycon.^113^ The resulting antagonist 85 showed further improved inhibitory activity due to enhanced interaction with the tyrosine gate. In an ELISA based assay a relative potency of 6900 (with reference to MeαMan) was achieved, an almost 35-fold increase compared to 84.

The affinity gain achieved by interacting with the hydrophobic tyrosine gate also led to the discovery of alkyl mannosides as FimH inhibitors. In crystallisation experiments by Knight and De Greve *et al.*, butyl α-d-mannoside (BM, 86) was found in the binding pocket of FimH, although no sugar was added during crystallisation.^402^ The Luria-Bertani (LB) medium used for protein expression was hypothesized as origin of the high affinity ligand BM (*K*_D_ = 151 nM, SPR), and after further investigation of simple alkyl mannosides, heptyl α-d-mannoside (HM, 78) was reported as potent binder of FimH (*K*_D_ = 5 nM, SPR).^402^ Despite the high affinity for isolated FimH, a concentration of 1 mM HM was needed to completely prevent bacterial binding to bladder cells *in vitro*. Additionally, HM only showed a significant reduction of bacterial binding and cell invasion in a murine cystitis model when the uropathogenic *E. coli* strain UTI89 was incubated with a high concentration of 5 mM HM before inoculation.^404^

###### FimH inhibitors to treat and prevent UTI

A milestone in the development of FimH antagonists was the introduction of the biphenyl aglycon to mannosides. Following a structure-based drug design approach aided by docking experiments, the first compounds of this new class were reported by Janetka *et al.* and Ernst *et al.* in 2010.^112,114^ The highest affinities were reported for derivatives carrying the second phenyl ring (B ring) in *para* position of the first phenyl ring (A ring). Furthermore, a loss of affinity was reported for *O*-glycoside derivatives carrying one to three methylene units as a spacer between the mannose and aromatic aglycon, highlighting the close proximity of the CRD and the tyrosine gate.^112,114^

**Fig. 18:**
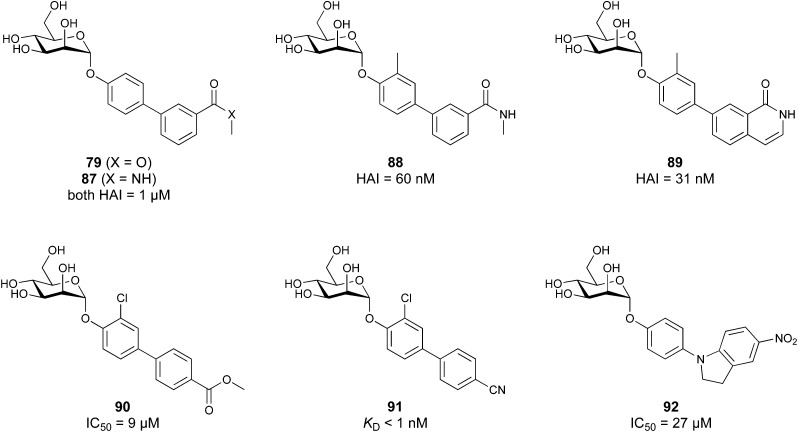
Aromatic FimH inhibitors developed by the Janetka (79, 87 to 89) and Ernst (90 to 92) groups. Biphenyl aglycons and their heterocyclic analogues engage in favourable interactions with the tyrosine gate of FimH, explaining the high affinity and efficacy of the respective mannosides.

Exploration of the substitution pattern on the B ring by Janetka *et al.* revealed a preference of *meta* substituents over *para* and *ortho*, attributed to improved interaction with Tyr48 of the tyrosine gate and Arg90.^112^ To quantify the activity of the synthesized derivates, the Janetka group determined the 90% inhibition titers of guinea pig erythrocyte hemagglutination (HAI titer), of which biphenyl ester 79 and methyl amide 87 were potent inhibitors (HAI titers = 1 μM), an improvement of more than 1000-fold compared to MeαMan (HAI titer >1 mM), and 15-fold compared to HM (HAI titer = 15 μM) (Fig. 18). The co-crystal structure of FimH and ester 79 revealed the basis for the greatly improved activity. While the mannose residue was bound in the CBS as previously reported,^402^ the biphenyl moiety engaged in π-stacking with Tyr48 and hydrophobic interactions with Tyr137 of the tyrosine gate. Importantly, the methyl ester in *meta* position of the second ring (B ring) contributed to binding *via* an interaction with the salt bridge of Arg98 and Glu50 at the outer rim of the binding region (Fig. 16D).^112^

Methyl amide 87 was chosen as the preferred lead structure due to a higher metabolic stability than ester 79. Introduction of a methyl group as *ortho*-substituent on the A ring resulted in the orally bioavailable lead compound 88, with improved affinity (HAI titer 60 nM) (Fig. 18).^415^ Dosed at 50 mg kg^−1^ by peroral administration (p.o.), 88 was able to reduce the bacterial burden in a murine chronic UTI model by approximately 3 log_10_ units 6 h after infection.^416,417^ Furthermore, establishment of an UTI with multidrug-resistant *E. coli* ST131 was prevented by 88, an effect not achieved by preventive administration of the antibiotic combination trimethoprim/sulfamethoxazole.^418^ These excellent results in the chronic UTI model were further surpassed by a reduction of 4 log_10_ units by isoquinolone 89 (HAI titer 31 nM), a cyclic amide analog of 88.^416^ Notably, derivatives of isoquinolone 89 showed further improved *in vitro* activity, in the low nanomolar range, but lacked significant efficacy *in vivo* in mice (50 mg kg^−1^ p.o.).

From a series of *para* substituted biphenyl mannosides the Ernst group identified ester 90 as early lead compound. 90 reached an IC_50_ value of 9 μM in an *E. coli* disaggregation assay from guinea pig erythrocytes compared to the IC_50_ value of 77 μM reported for HM.^114^ Importantly, the carboxylate metabolite does not significantly lose activity (IC_50_ = 10 μM). After extensive *in vitro* studies on binding affinity and pharmacokinetic properties, as well as the first *in vivo* PK study, the efficacy of lead compound 90 was tested in a murine UTI model. Dosed at 50 mg kg^−1^ p.o. before transurethral infection with the UPEC strain UTI89, 90 led to a decrease of bacterial burden in urine by 2 log_10_ colony forming units (CFU) and in the bladder by 4 log_10_ CFU. Importantly, the same reduction was achieved by intravenous (i.v.) application of the carboxylate metabolite of ester 90 (50 mg kg^−1^) confirming oral bioavailability and potency of lead mannoside 90.^114^ Optimisation of the PK/PD profile, with special focus on the plasma half-life to prolong the effective duration resulted in the bioisostere nitrile 91, with steady renal excretion over 8 hours.^419^ Preventive administration of 91 (10 mg kg^−1^, p.o.) was more effective than administration of the antibiotic ciprofloxacin (8 mg kg^−1^, subcutaneous, s.c.) in reducing the bacterial count in the bladder (reduction of 2.7 log_10_*vs.* 2.4 log_10_ CFU). Further, fluorination of the B ring led to sub-nanomolar binders to the isolated (high-affinity) FimH domain, but efficacy data were not reported.^420^

To allow binding in the “in-docking” mode, Ernst *et al.* synthesized a series of phenyl triazole compounds with varying spacer length and modifications to the anomeric centre.^421^ No improvement of the binding affinity compared to HM or biphenyl aglycons was achieved and, furthermore, triazole mannosides are not predicted to be orally bioavailable due to limited membrane permeation.

Replacement of the B ring phenyl with heterocycles led to the discovery of 92 as highly potent FimH inhibitor (Fig. 18).^422^ Despite dosage limits due to low solubility, the concentration to prevent 90% of bacterial adhesion by UPEC (EC_90_) was exceeded for more than 8 hours after i.v. administration of 1 mg kg^−1^ in a PK mouse study. Efficacy of 92 was proven by preventive administration in a mouse model. Dosed at 1 mg kg^−1^ (i.v.), 92 achieved a 10 000-fold reduction of CFU in the bladder of mice, an effect equally potent to the antibiotic ciprofloxacin (8 mg kg^−1^, s.c.) commonly used to treat UTIs. Despite higher *in vitro* potency of the analogue carrying an additional chloro substituent in *ortho* position of the proximal phenyl ring (ring A), the compound was not evaluated *in vivo* due to limited solubility.^422^

Due to the plethora of diverse mannose-binding proteins in humans (*e.g.* DC-SIGN, mannose binding protein, dectin-2, langerin), selectivity of glycomimetics for their intended target is of crucial importance. A selection of compounds, including HM and biphenyl mannosides, showed a 100 000-fold higher affinity for FimH compared to the tested human mannose binding proteins, indicating binding selectivity is not a problem in the development of monovalent FimH inhibitors.^423^

**Fig. 19:**
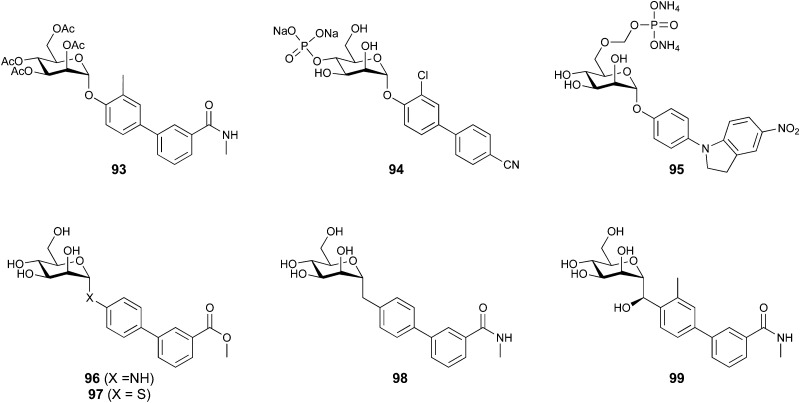
Strategies to improve oral bioavailability of aromatic FimH inhibitors include prodrugs (top) and modification of the *O*-glycosidic bond (bottom).

A general problem of biphenyl mannosides is their low solubility, limiting the therapeutic dose, and their low bioavailability, at least partially due to the low stability of the *O*-glycosidic bond. Janetka and colleagues investigated acetate and phosphate esters, as well as a glycine derivative as prodrug strategies to improve oral bioavailability and prolong compound exposure, with positive results for tetraacetate (93) and 6-phosphate mannoside prodrugs (Fig. 19).^424^

In particular, the use of phosphate prodrugs is commonly applied to drugs suffering from limited solubility. The phosphate prodrug lacks membrane permeability and acts as soluble reservoir for the active agent, which is released by enzymatic hydrolysis prior to uptake. Thereby, the concentration of free, poorly soluble drug is limited, but a high dose can be administered. Following this strategy, the Ernst group synthesized a series of mannosides as phosphate esters (94 and 95) reaching excellent aqueous solubility (125-fold improvement for heterocyclic 92; 15-fold improvement for nitrile 91, >3000 μg mL^−1^*vs.* 192 μg mL^−1^) (Fig. 19).^425^

Depending on the position of the phosphate ester, the rate of hydrolysis varied. Compounds carrying the phosphate at O4 or O6 of mannose displayed a desired longer half-life (*t*_1/2_ > 40 min) compared to O2 or O3 derivatives (*t*_1/2_ < 15 min). In an *in vivo* PK study in mice, a doubling of the urine AUC_0-24_ was achieved by p.o. administration of the O4 phosphate ester prodrug (94) of lead nitrile 91 (Fig. 18 and 19).

Efforts to improve metabolic stability of the mannosides included replacement of the glycosidic oxygen by carbon, nitrogen, and sulfur (96–98).^424,426,427^ In a seminal work, Janetka and colleagues reported on *C*-mannosides with an (*R*)-hydroxy methylene unit linking the carbohydrate and its biphenyl aglycon.^424^ This replacement resulted in potent inhibitors of same or higher affinity, with significantly improved metabolic stability compared to the *O*-glycoside. In a murine chronic cystitis model, *C*-mannoside 99 dosed at 25 mg kg^−1^ (p.o.) reduced the CFU in the bladder by 3 log_10_ units after 12 hours, while *O*-glycoside analogue 88 did not result in a significant reduction of bacterial burden. Furthermore, introduction of the (*R*)-configured hydroxy methylene unit to inhibitors bearing an isoquinolone moiety (as 89) resulted in additional promising preclinical candidates. The pronounced stereochemical selectivity for the (*R*)-epimer is due to a water-mediated hydrogen bond to Asp140 and Asn135 formed by the (*R*)-hydroxy group, an interaction that is not possible for the (*S*)-epimer, as predicted by docking.

In 2012, Janetka and Hultgren co-founded Fimbrion Therapeutics which further pursues the development of FimH inhibitors, since 2016 in cooperation with GlaxoSmithKline. With financial support by CARB-X, the first clinical Phase I evaluation of the joint lead compound GSK3882347 (undisclosed structure) in healthy individuals was successfully completed in May 2021 (NCT04488770). A second Phase I study to investigate the pharmacokinetics and microbial response in women with uncomplicated acute UTI was started in May 2022 and is expected to end in December 2023 (NCT05138822).

###### FimH inhibitors to treat Crohn's disease

**Fig. 20:**
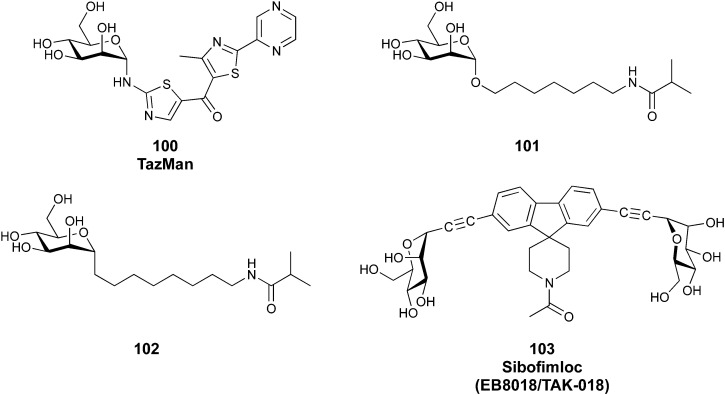
FimH inhibitors under investigation for the treatment of Crohn's disease. 100 is a representative of the thiazolylaminomannoside (TazMan) class of FimH inhibitors, which noticeably interact with the tyrosine gate in a more favourable way compared to biphenyl aglycons. Terminal modification of the heptyl chain in 101 significantly improved the *in vivo* efficacy compared to HM. Replacement of the anomeric oxygen improved metabolic stability (102). Divalent C-mannoside Sibofimloc has been advanced to Phase IIa clinical trials.

In the context of Crohn's disease (CD), the site of infection is the intestine, requiring alternative strategies for inhibitor design with regards to pharmacokinetics: while treating UTIs requires enteral absorbance and renal excretion of the parent drug or of an active metabolite, no systemic bioavailability is required to treat CD in the gut. Gouin and colleagues investigated thiazolylaminomannosides (TazMan) with heterocyclic aglycons to treat CD.^428^ Notably, lead 100 (relative inhibitory concentration compared to HM, 0.2) engages in more favourable fashion with Tyr48 and Tyr137 of the tyrosine gate, compared to the phenyl moiety in biphenyl aglycons (Fig. 20). Variation of the linker length and replacement of the anomeric nitrogen by carbon or sulfur improved stability but resulted in less potent compounds.^429^

Additionally, Gouin *et al.* investigated further alkyl mannosides. They hypothesized unfavourable pharmacokinetic properties caused by the unmodified alkyl chain in HM as cause for its poor activity *in vivo.* By terminal functionalization amide derivative 101 was obtained, which in contrast to HM showed promising results of reduced gut colonization and inflammation *in vivo* in a murine model of Crohn's disease (Fig. 20).^430^ Exchanging the anomeric oxygen for a methylene in 102 increased metabolic stability and simultaneously lowered systemic bioavailability from 25% to 16%, as desired for the treatment of Crohn's disease. Importantly, compound 102 (10 mg kg^−1^, p.o.) was able to eradicate AIEC from the ileum of transgenic mice. This observation serves as proof of concept that patients suffering from Crohn's disease with increased prevalence of AIEC can be treated orally with FimH antagonists.^431^

A major industrial player in the preclinical development of FimH inhibitors is Vertex Pharmaceuticals, who filed patents on a plethora of diverse mannose-based FimH inhibitors, including disaccharides and divalent ligands, with varying aglycons, intended for the treatment of UTIs and inflammatory bowel diseases.^432–436^ The portfolio was licensed to Enterome in 2016, where Sibofimloc (EB8018/TAK-018, 103), a divalent and gut-restricted FimH ligand, was identified as lead compound for the treatment of Crohn's disease (Fig. 20). *In vitro* adhesion of AIEC to primary ileal cells and T84 epithelial cells was blocked by 1 μM Sibofimloc, resulting in reduced inflammation and tissue damage.^383^ After successful completion of Phase I studies on safety and tolerability (NCT02998190) as well as pharmacokinetics (NCT03709628), a Phase IIa study to investigate the preventive effect of Sibofimloc on the postoperative recurrence of Crohn's disease was initiated in cooperation with Takeda Pharmaceutical in 2020 (NCT03943446). However, problems to recruit suitable participants led to termination of the study in August 2022.

##### 3.1.4.2 *E. coli* F9 fimbrial adhesin FmlH

In addition to type 1 fimbriae and the FimH adhesin, *E. coli* often utilize the related UPEC F9 fimbriae with the adhesin FmlH to adhere to galactose and *N*-acetyl galactosamine conjugates, such as the Thomsen–Friedenreich (TF) antigen on kidney and bladder tissue in urinary tract infections.^437^ F9 fimbriae are homologous to type 1 fimbriae, and as such, FmlH is also related to FimH, although it displays a different carbohydrate-binding specificity.

**Fig. 21:**
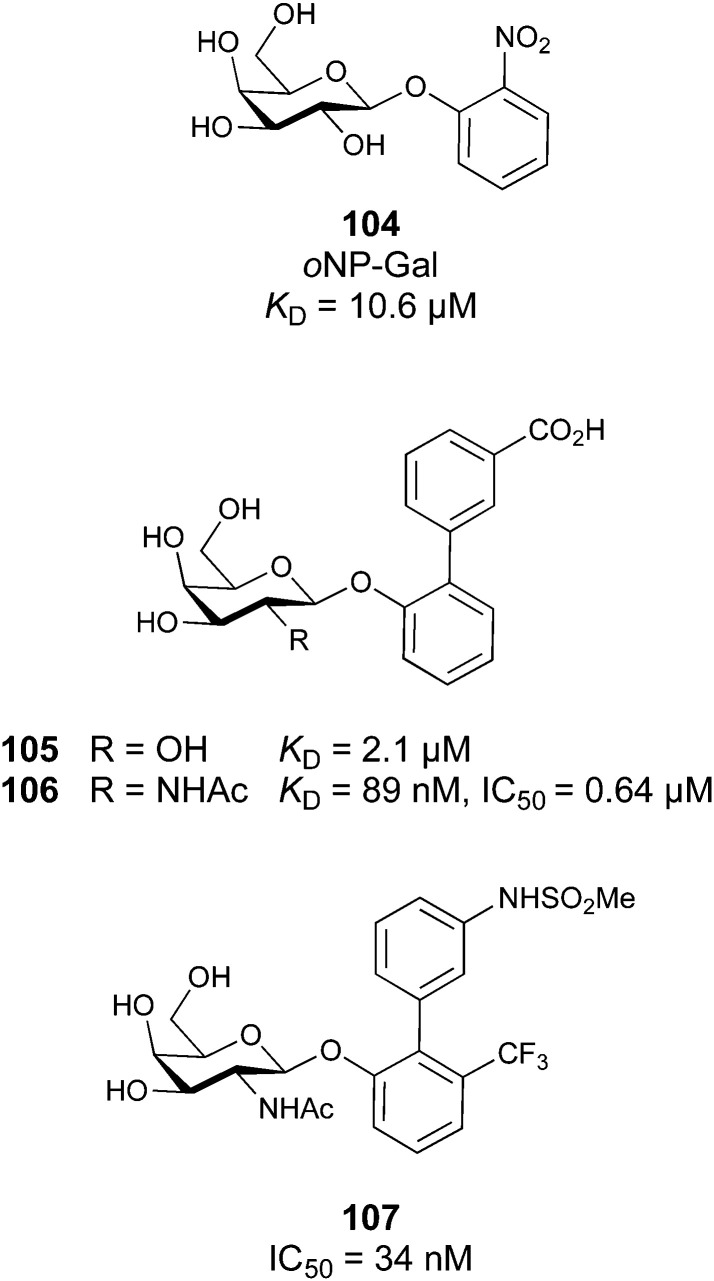
Inhibitors of the F9 fimbrial adhesin FmlH of uropathogenic *E. coli*. In contrast to its namesake FimH, the adhesin FmlH shows the highest affinity for *ortho*-biphenyl substituted galactosides and GalNAc glycosides.

Complementary to their FimH research, Janetka and Hultgren have therefore embarked on the synthesis of FmlH inhibitors.^118^ To this end, a number of GalNAc glycosides with varying aglycons have been synthesized and tested for inhibition of FmlH binding to immobilized TF-antigen, followed by infection studies in mice. The crystal structures of FmlH in complex with the TF-antigen or *o*NP-Gal (104) revealed a tight coordination of the galactose moiety in both ligands and demonstrated the possibility of varying the aglycon (Fig. 21). It was further demonstrated that substitution of galactose (*K*_D_ = 694 μM) with GalNAc (*K*_D_ = 189 μM) enhances binding affinity of FmlH inhibitors, as determined by bio-layer interferometry. An extensive synthesis campaign also revealed substituted biphenyl glycosides as potent binders of FmlH. The most potent ligand was the β-GalNAc biphenyl carboxylate 106 with a *K*_D_ of 89 nM, and for its galactose analogue 105, a 50-fold lower binding of *K*_D_ = 2.1 μM was determined. This selectivity of FmlH for GalNAc was rationalized by an attractive hydrogen-bonding interaction of the acetamide with a protein-bound water molecule in the co-crystal structure with the protein. In a murine cystitis model, it was demonstrated by transurethral instillation that the potent FmlH inhibitor 106 synergizes with a FimH inhibitor (mannoside 4Z269) in reducing colonization of the kidney, while in the bladder no synergy was observed and the FimH inhibitor dominated (Fig. 21).

The team then further optimised these compounds and identified a methylsulfonamide as a bioisostere of the carboxylate, as well as a beneficial effect of an additional CF_3_ group in the proximal phenyl *meta* to the carbohydrate.^119^ The resulting compound 107 showed an IC_50_ of 34 nM in a competitive, ELISA based binding assay, which was 20-fold more potent than the initial lead 106 (Fig. 21). The co-crystal structure of 107 with FmlH revealed that both the additional CF_3_ group and the sulfonamide residue form attractive interactions with the protein, providing an explanation for the observed activity increase. Importantly, compound 107 displayed an increased metabolic stability and an extended plasma half-life, but unfortunately, also a low bioavailability of only 1% after p.o. administration and only a low renal clearance were observed, requiring further optimisation of its PK properties.

##### 3.1.4.3 The soluble lectins LecA and LecB of *Pseudomonas aeruginosa*

The Gram-negative bacterium *P. aeruginosa* is currently the most critical bacterial pathogen as defined by the WHO priority pathogen list. This bacterium is difficult to treat due to excessive development of resistance to antibiotics and its abundant biofilm formation.^438^ The latter is a major resistance determinant of this pathogen since the biofilm shields embedded bacteria from chemotherapy and host defence. Therefore, several approaches to identify new anti-infectives against this bacterium aim to block biofilm formation.^439^

*P. aeruginosa* utilises the two lectins LecA (PA-IL) and LecB (PA-IIL) for initial adhesion to the host, for biofilm formation and as virulence factors.^440–442^ Both proteins have first been identified by Gilboa-Garber *et al.* and their carbohydrate binding specificity was determined.^443^ In particular, it was shown that both lectins inhibit ciliary beating,^444,445^ wound healing^446,447^ and impact on cell physiology^448,449^ and immunity.^450,451^ In addition, LecA was shown to promote host cell invasion by *P. aeruginosa*.^452^ In first-in-human studies, aerosols containing the monosaccharides d-galactose and l-fucose as ligands of LecA and LecB, respectively, have shown beneficial effects on patients with *P. aeruginosa* lung infections after inhalative administration.^453,454^ Further, adjunctive therapy in mice with the antibiotics ceftazidine or ciprofloxacin revealed synergistic effects when galactose, mannose and fucose were co-administered with the bacterial inoculum to the murine lung.^455^

**Fig. 22:**
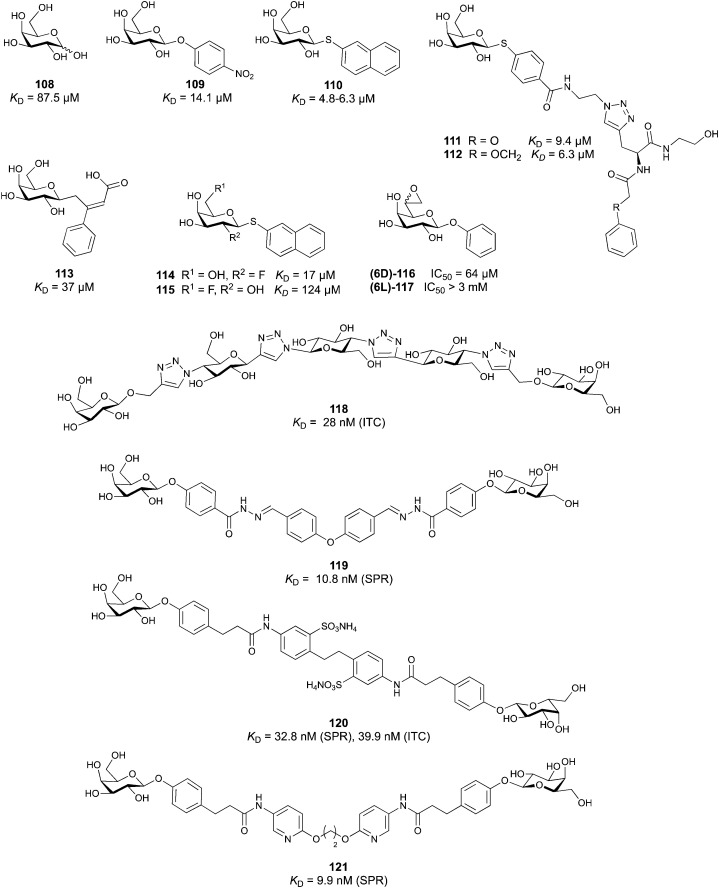
Glycomimetic inhibitors derived from galactose for the bacterial lectin LecA. Notably, (6D)-116 is an irreversible LecA inhibitor as nucleophilic attack of Cys62 to the epoxide warhead leads to covalent binding.

**Fig. 23:**
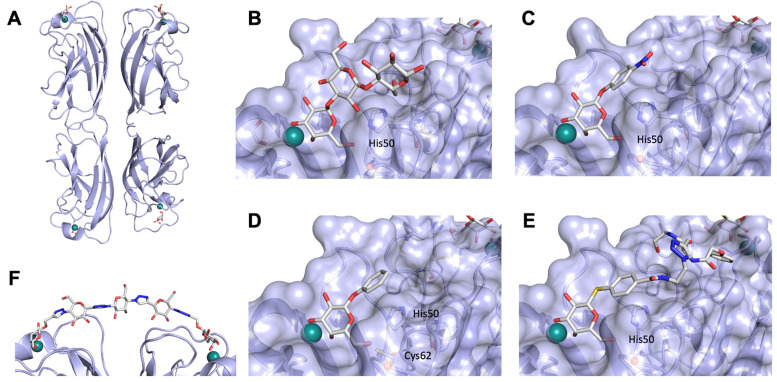
LecA forms a homotetramer with the carbohydrate binding sites arranged on the vertices of a rectangle (A, PDB code 1OKO). Binding sites occupied with various ligands are magnified: Gal-α(1,3)-Gal-β(1,4)-Glc (B, PDB code 2VXJ), 109 (C, PDB code 3ZYF), epoxide (6D)-116 (D, PDB code 5MIH), and central pocket targeting ligand 111 (E, PDB code 7FIO). The divalent ligand 118 bridges two adjacent binding sites in the LecA tetramer (F, PDB code 4YWA). His50 establishes CH–π contacts with aryl aglycons in (C–E) and forms a hydrogen bond with O6 in LecA ligands. In the crystal structure of LecA with (6D)-116 obtained at pH 4.6 (D), Cys62 does not form the covalent bond observed in solution at physiological pH and the epoxide remains intact. Calcium ions are depicted as green spheres, a tightly coordinated water molecule is shown as red sphere.

LecA binds d-galactose (108, Fig. 22) and conjugates thereof and its native ligand is presumably the glycosphingolipid Gb3 that was identified from a glycan array screen.^456^ The structure of LecA in complex with galactose was solved by X-ray crystallography (Fig. 23A).^457^ This structure reveals the oligomerization of LecA into a homotetramer with the binding sites on the vertices of a rectangle and further demonstrates the calcium ion-mediated recognition of the carbohydrate ligand: the 3- and 4-hydroxy groups coordinate to the protein-bound calcium ion whereas the 6-hydroxy group is recognized in a small water-occupied pocket and involved in direct hydrogen bonding with His50 and Gln53, as well as with Gln53 and Pro51 *via* water-mediated hydrogen bonds. In addition, the 2-, 3-, and 4-hydroxy groups form hydrogen bonds with Asn107 and Asp100.

The search for inhibitors of LecA surprisingly identified β-aryl galactosides (*e.g. p*NP-Gal 109) as more potent inhibitors for LecA compared to d-galactose or α-galactosides (Fig. 23B).^458^ In a crystallographic study, the molecular basis has been attributed to a CH–π interaction between the aryl aglycone and the imidazol side chain of His50 (Fig. 23C),^459^ and consequently the naphthyl galactoside 110 showed high binding to LecA (*K*_D_ = 4.2 μM), while d-galactose has a *K*_D_ of 87.5 μM. Various SAR studies on an aromatic aglycone have been reported and in general, substitution is tolerated on the phenyl ring, but major additional increase in affinity has not been achieved to date.^459–461^ Recently, the Titz group identified a further druggable pocket located between two LecA monomers and close to the carbohydrate binding site and adjacent to the His50-bound phenyl aglycon.^462^ To this end and inspired by a multivalent system of Winssinger *et al.*,^463^ they systematically elongated the aglycon with flexible spacers and attached aryl groups for their ligation to the central pocket as predicted by docking. The crystal structure of 111 (*K*_D_ = 9.4 μM) in complex with LecA indicated the phenyl pharmacophore close to the entry of the central pocket (Fig. 23E). Further, a ligand with an increased chain length (112, *K*_D_ = 6.3 μM) showed a high enthalpy of binding (−50 kJ mol^−1^) which was counterbalanced by high entropic costs (20.7 kJ mol^−1^) indicating a conformationally restricted bound state. However, further experiments are necessary to validate its binding inside the central pocket.

Replacing the glycosidic oxygen to increase metabolic stability by introducing thioglycosides (see 110) has been pursued and is well tolerated by LecA.^458,460^ Another work reports on the synthesis of β-*C*-galactosides with positioning of a phenyl ring further away from the galactose residue which resulted in significant loss of binding affinity with one of the derivatives reaching a *K*_D_ of 37 μM (compound 113, Fig. 22).^464^

Giguère and co-workers reported on the systematic single and multiple exchange of all hydroxy groups with fluorine atoms in galactosides as tools to assess lectin binding.^465^ It was shown that the 2-deoxy-2-fluoro galactoside 114 resulted in an approximately 3-fold loss in binding affinity by ITC when compared to the parent galactoside (*e.g.* 17 μM *vs.* 4.8 μM for the thionaphthyl glycoside 110). The enthalpy of LecA-binding is strongly reduced in those fluoro-analogues by approx. 7–10 kJ mol^−1^. However, this work also demonstrates the lower entropic costs by 4–6 kJ mol^−1^ for binding of these fluoroderivatives compared to the galactosides as a result of different desolvation penalties which to some extent counterbalances the unfavourable enthalpy. For the 6-deoxy-6-fluoro analogue 115, the binding affinity was drastically reduced and 25-fold lower affinities were reported. A similar observation of an affinity reduction has been made when the 2-OH group in d-galactose was replaced by NHAc in GalNAc.^458,466^

Binding kinetics are often fast for carbohydrate–lectin interactions and consequently, the binding affinity of a given ligand is lowered when fast dissociation rates are in place (lit.^467^ and references therein). Furthermore, a sufficiently long residence time of a given inhibitor on a receptor has been identified as crucial for success *in vivo*.^468^ In some cases, the success of glycomimetic inhibitors resulted from their extended receptor-residence times, *e.g.* for the FimH antagonists^467^ or LecB inhibitors.^469,470^ However, for LecA even some of the best monovalent ligands with *K*_D_s <3 μM showed a very fast dissociation from the protein.^471^

Therefore, the covalent inhibition of a given target can provide an opportunity to overcome fast dissociation by irreversibly blocking its function. Covalent inhibition is an approach with increasing success in clinical use for inhibition of unrelated proteins, *e.g.* proteases or kinases.^472^ Inspection of the crystal structure of LecA in complex with a galactoside reveals the presence of a cysteine residue in close proximity to the carbohydrate. Cysteines are nucleophiles and therefore often targeted using electrophilic warheads, for example in covalent inhibitors of cysteine proteases. In the case of LecA, Cys62 is located in close proximity to the hydroxymethyl group of galactose, and therefore this position was selected for the introduction of an electrophile in LecA inhibitors.^473^ Since this part of galactose is accommodated in a rather small pocket in LecA, epoxides were chosen as sterically least demanding electrophiles. To this end, phenyl galactoside was extended to the diastereomeric heptose epoxides 116 and 117 (Fig. 22). Biophysical analysis of these molecules with LecA revealed a weak but selective inhibition of LecA by one diastereomer (116, IC_50_ = 64 μM), whereas the other diastereomer 117 was inactive. Protein mass spectrometry then unambiguously demonstrated the covalent bond formed between Cys62 and the epoxide in 116 under physiological conditions, providing the experimental proof for the first-in-class covalent lectin inhibitor. Surprisingly, crystallographic analysis of LecA in complex with 116 revealed its non-covalent ligation with the epoxide ring intact (Fig. 23D). This unexpected observation could be explained by the acidic conditions for LecA crystallisation which lower the cysteine's nucleophilicity.

In general, numerous attempts to improve binding affinity for LecA on the monovalent galactoside level have delivered an in-depth knowledge on the SAR. Unfortunately, all those studies resulted in only moderate affinity increases and the best compounds consist of β-galactosides carrying naphthyl (110) or coumaryl aglycones. These substituents may, however, encounter problems in further translation due to toxicity.

To overcome this rather moderate affinity of the small molecules, numerous approaches have exploited the quaternary structure of LecA as target rather than focussing on a single binding site. To this end, oligo- and multivalent galactosides presented on a large diversity of scaffolds were developed which are beyond the scope of this review (the interested reader is referred to references^75,131,474^ and references of this ChemSocRev issue). However, most of these multivalent ligands have been studied for lectin binding only. Only few molecules were tested further: galactosylated peptide dendrimers have shown good potency in antibiofilm experiments *in vitro*^475^ and galactosylated calixarenes showed *in vivo* efficacy to reduce lung damage in an acute murine lung infection model in a preventive treatment regime using co-administration of bacteria and inhibitor.^476^ Another approach worth mentioning here are divalent precision ligands containing two terminal galactosides tailored to match the simultaneous binding to two adjacent galactose-binding sites in LecA. This approach was pioneered by the Pieters lab and exploits the close proximity of these two sites.^477–480^ One molecule that consists of two galactosides spaced by a number of triazoles and glucose moities, 118, could even be cocrystallised with LecA and the chelating binding mode was proven (Fig. 23F). Non-carbohydrate spacers have been developed by Titz *et al.* to enable rapid synthetic access and implement drug-like properties, yielding a highly potent divalent LecA inhibitor 119 (*K*_D_ = 10.8 nM) that showed a high selectivity over human Galectin-1 as potential off-target.^471^ Recently, these bisacylhydrazone spacers have been bioisosterically replaced with amides to overcome the drawbacks of this labile and potentially toxic linker function.^446^ The resulting molecules showed superior metabolic stability and much higher aqueous solubility (*e.g.* for 120 up to >1.5 mM *vs.* 1.6 μM for the bisacylhydrazones) and the best derivative 121 bound to LecA with a *K*_D_ of 9.9 nM. More importantly, the increased solubility now enabled ITC analysis and evaluating the molecules’ antivirulence properties in cell culture and *in vitro* infection experiments.

**Fig. 24:**
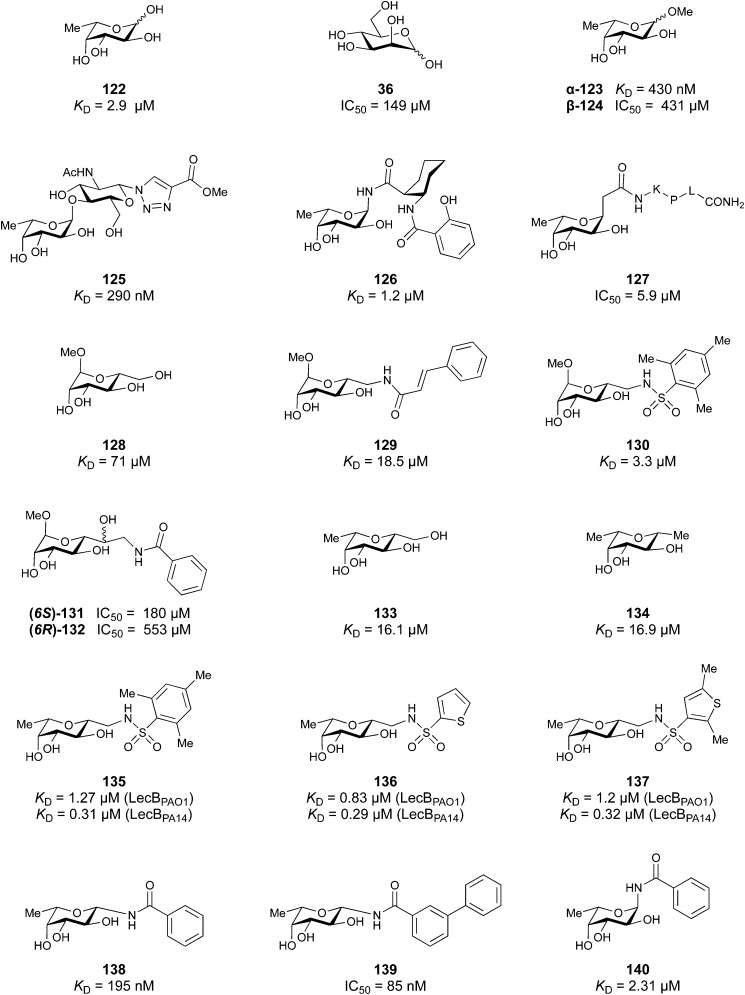
Inhibitors of the *P. aeruginosa* lectin LecB are derived from l-fucose (122), d-mannose (36) or a hybrid of both (133).

**Fig. 25:**
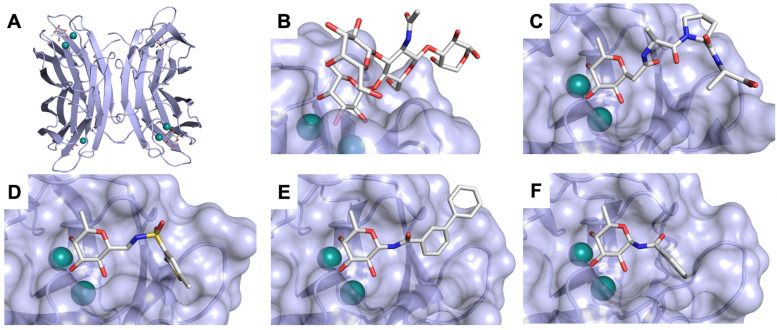
LecB forms a homotetramer with the carbohydrate binding sites arranged on the vertices of a tetrahedron (A, PDB code 1GZT). Binding sites occupied with various ligands are magnified: LewisA (B, PDB code 5A6Z), *C*-fucosyl peptide 127 (C, PDB code 3DCQ), fucose–mannose hybrid 137 (D, PDB code 5MAZ), β-*N*-fucoside 139 (E, PDB code 8AIY), and α-*N*-fucoside 140 (F, PDB code 8AIJ). Calcium ions are depicted as green spheres.

In contrast, LecB binds to l-fucose 122 and d-mannose 36 and their conjugates (Fig. 24). Following a glycan array screening, the blood group antigen LewisA has been identified as ligand with the highest affinity.^481,482^ The crystal structure of LecB from *P. aeruginosa* PAO1 has been solved in complex with its ligands (Fig. 25)^483,484^ and a recent neutron diffraction structure of LecB in complex with l-fucose allowed the analysis of ligand and protein protonation states.^485^ As observed for galactophilic LecA and despite the unrelated primary protein sequences, LecB also forms homotetramers albeit with a different orientation of the binding sites. In LecB, the carbohydrate binding sites are located on the vertices of a tetrahedron and thus, spatially maximally apart from each other (Fig. 25A). Surprisingly, the crystal structure revealed that two calcium ions are located inside a single carbohydrate binding pocket and these ions mediate the binding of the carbohydrate ligand with the protein. The 2-, 3-, and 4-hydroxy groups of fucose are directly coordinated to the two calcium ions with the 3-hydroxy group simultaneously serving as a μ-bridging ligand to both metal ions. In addition, the ligand is also heavily involved in hydrogen bonding with the protein. The presence of the two metal ions as well as an additional lipophilic contact of the C6 methyl group of fucose served as an explanation for the unusual high affinity of a monosaccharide for a lectin (*K*_D_ = 2.9 μM).^482^d-Mannose possesses the same relative orientation of its three ring hydroxy groups as those in l-fucose. d-Mannose can therefore also bind in a similar orientation to LecB, but with the absence of the lipophilic interaction of fucose, the affinity for d-mannose is reduced.

Importantly, despite the fact that *P. aeruginosa* is a highly variable organism at the genomic level, the genes encoding for LecA and LecB are part of the more conserved core genome.^486^ Nevertheless, the sequence of LecB varies among clinical and environmental isolates.^487,488^ Interestingly, the sequence of LecB has been established as correlative marker for the two major clades in *P. aeruginosa* phylogeny, one containing the clinical isolate PAO1 and the other containing the highly virulent clinical isolate PA14.^487^ It was demonstrated by glycan array analysis and biophysical characterization that the carbohydrate-specificity in LecB from PA14 is conserved, despite a high number of amino acid variations, some of which located in the carbohydrate binding site. Moreover, it was shown that the binding affinity of LecB_PA14_ for its ligands is even 3- to 7-fold higher than the one of LecB_PAO1_. As observed from mammalian glycan array binding data, LecB from both strains potently binds to a broad diversity of glycans carrying either fucose residues, or mannose residues or both, which suggests a decisive role of LecB for bacterial adhesion to the host.

Due to the high affinity of LecB for l-fucose, the search for LecB inhibitors focussed on this deoxyhexose as a starting point (Fig. 24).^439,489^ In eukaryotes, l-fucose is generally an α-linked terminal carbohydrate in glycoconjugates. Therefore, α-linked fucosides have initially been at the centre of attention and methyl α-l-fucoside 123 revealed a strong affinity for LecB_PAO1_ (*K*_D_ = 0.43 μM).^482^ In fact, the anomeric isomer methyl β-l-fucoside 124 is an up to 500-fold weaker ligand for LecB.^487^ Since the trisaccharide LewisA (Fuc-α-1,4-(Gal-β-1,3)-GlcNAc, Fig. 25-B) showed the highest potency for LecB_PAO1_ (*K*_D_ = 0.21 μM)^482^ and its interaction with LecB revealed no contact with the galactose residue, Fuc-α-1,4-GlcNAc truncates with triazoles at the reducing end of GlcNAc have been synthesized and their affinity (*e.g.*125, *K*_D_ = 0.29–0.31 μM) remained nearly as potent as the one of LewisA.^490^ Along these lines, Bernardi, Imberty *et al.* reported α-linked fucosyl amides that were predicted by docking to establish an additional hydrogen bond between their carbonyl oxygen and Ser23.^491^ The best derivative 126 showed low micromolar affinity with a *K*_D_ of 1.2 μM. α-*C*-fucosides have also been described to implement chemical/metabolic stability and allow conjugation to peptide dendrimers.^492^ The affinity of the tripeptide conjugate cFuc-Lys-Pro-Leu-NH_2_ (127) was equipotent to nitrophenyl α-l-fucoside (IC_50_ = 5.3–5.9 μM), which was twice better than the reducing l-fucose in the same assay (Fig. 25C).

Since terminal α-l-fucosides are abundant in humans and numerous endogenous lectins bind to fucosides,^1^ carefully addressing the selectivity of LecB inhibitors is highly relevant, especially when used in multivalent systems. To address this concern, the Titz group has chosen methyl mannoside 128 as a starting point for derivatization.^493^ The methylene-linked 6-OH group is an equatorial substituent in mannose and therefore allows contact to LecB, in contrast to the aglycones of α-fucosides that are axial substituents and point towards the solvent. The latter could serve as an explanation that no increase in affinity for α-fucosides has been achieved.

In the first set of molecules, the 6-hydroxy group was replaced by an azide that yielded a library of triazoles and after reduction amides, sulfonamides and amines. Sulfonamides have been identified as the most potent derivatives and the binding affinity was increased from 71 μM (*K*_D_ for methyl α-d-mannoside) to a *K*_D_ of 3.3 μM for sulfonamide 130 and cinnamide 129 also showed moderate affinity (*K*_D_ = 18.5 μM). It was demonstrated that the increased binding affinity of sulfonamides and cinnamides results from an approx. 10–25-fold decreased dissociation rates of those derivatives from their complex with LecB (*k*_off_ = 0.6–1.8 × 10^3^ s^−1^) compared to MeαMan (*k*_off_ = 15.5 × 10^3^ s^−1^).^469^ In the next step, these mannosides were homologized to mannoheptoses with two aims: (i) those mannoheptoses have a free 6-OH that could engage in hydrogen bonding with Ser23 as observed for mannose in the crystal structure^494^ with LecB and (ii) the (sulfon-) amide substituent could be moved further towards the identified subpocket in this direction.^495^ Both diastereomers were synthesized and analysed for LecB inhibition: although a selectivity for the (6*S*)-heptose 131 over its (6*R*)-diastereomer 132 was demonstrated, IC_50_ values remained high (IC_50_ = 82–217 μM). In another work, they further analysed the function of this OH group, originating from Man-O6 which establishes a hydrogen bond to Ser23 in the crystal, to quantify its contribution to binding in solution.^496^ Furthermore, they set out to test the hypothesis of a possible synergistic effect of the Fuc methyl group and said Man-O6 hydroxy group in LecB inhibitors. To this end, a hybrid of l-fucose and d-mannose was synthesised, resulting in compound 133 and deoxygenated to 134. Both molecules were tested in a competitive binding assay and by ITC for LecB binding and surprisingly, they showed very similar thermodynamic profiles with comparable *K*_D_s of 16–17 μM. These data led to the conclusion that synergism for binding affinity is not obtained and that the hydrogen bond formed between mannose-6-OH and Ser23 in the solid state does not contribute to the binding affinity in solution.

However, these molecules are *C*-glycosides which increases their chemical and metabolic stability. Furthermore, their combination with the sulfonamide substituents likely also increases selectivity since the resulting molecules are neither terminal α-linked fucosides nor terminal mannosides, the common ligands for many endogenous lectins. Therefore, the Titz group designed and synthesized a series of these derivatives as sulfonamides and amides.^470,497^ As observed for the mannosides, especially the sulfonamide derivatives 135, 136 and 137 showed very potent binding of LecB_PAO1_ (*K*_D_ = 0.83–1.27 μM) and LecB_PA14_ (*K*_D_ = 290–320 nM). The crystal structure of those molecules in complex with LecB revealed that the aromatic substituents are ligated to a subpocket adjacent to the carbohydrate binding site and form extensive contacts with various hydrophobic amino acids, *e.g.* Val69, and methyl-substituted derivatives 135 and 137 revealed a deeper binding in the pocket (Fig. 25D). With regards to binding kinetics, the ligands’ off-rate could be further reduced to *k*_off_ = 0.41 × 10^3^ s^−1^ which results in receptor-residence times of 28 min, compared to 45 s for MeMan. Importantly, two derivatives were then assessed for selectivity over the human lectin langerin. While the thermodynamic affinity of 135 for LecB is 1.27 μM (from PAO1) or 310 nM (from PA14), the *K*_I_ for the fucose-binding langerin was 2.2 mM, defining a selectivity window of a factor 1700–7000. In addition, 135 and other glycomimetics were also tested more globally on their effect on the immune response by quantifying the TNF-α response from primary murine spleen cells upon addition of various immune stimuli acting *via* diverse immune response pathways. No effect of the sulfonamide glycomimetics 130 or 135 (up to 1 mM) on the production of TNF-α was observed after stimulation, further supporting their selectivity.

Several glycomimetics were then tested in a biofilm inhibition assay using *P. aeruginosa* PA14 constitutively expressing mCherry and quantification of biofilm mass by confocal fluorescence microscopy. In 48 h biofilm formation experiments, a dense biofilm was observed in absence of LecB inhibitor, whereas in the presence of 100 μM glycomimetics biofilm formation was inhibited by 50–90%, and *C*-glycosidic sulfonamides 135 and 136 showed the highest potency of inhibition (80–90%). Importantly, in presence of MeαMan or even the very potent LecB ligand MeαFuc (*K*_D_ = 202 nM for LecB_PA14_)^487^ biofilm mass was only non-significantly reduced under identical conditions, which impressively demonstrates the advantages of glycomimetics over native glycosides.

Because these glycomimetics showed nanomolar inhibition of LecB_PA14_, prevention of *P. aeruginosa* biofilm formation and excellent *in vitro* ADME/toxicity properties, 135 and 136 were then analysed in a murine pharmacokinetics study dosed at 10 mg kg^−1^ i.v. or p.o. Both molecules were orally bioavailable and the experiments revealed their renal excretion. However, large differences after intravenous administration in exposure (AUC 1.72 μg mL^−1^ h^−1^ and 7.40 μg mL^−1^ h^−1^ for 135 and 136, respectively) and half-lives (*t*_1/2_ = 0.28 h and 0.57 h for 135 and 136, respectively) have been detected with 136 exhibiting a superior pharmacokinetic profile, also after per oral administration. Consequently, these molecules are currently under investigation in murine models of *P. aeruginosa* infection.

In the search for new glycomimetics addressing the subpocket adjacent to the anomeric centre of the bound fucose, Titz *et al.* have recently reported *N*-linked β-fucosyl amides as potent LecB ligands.^498^ In contrast to the α-linked derivatives reported by Bernardi before (*e.g.*126), these β-anomers revealed an unexpected high potency for LecB of IC_50_s down to 85 nM and a *K*_D_ of 195 nM for β-fucosyl benzamide 138 as determined by a competitive binding assay and ITC. Thus, these compounds have shown a >10-fold increase in binding affinity for LecB compared to the isomeric α-fucosyl benzamide 140, which has a *K*_D_ of 2310 nM by ITC. The increased potency results from an increased entropy of binding of the β-anomer, while the enthalpy of binding was comparable for both. The crystal structure of β-amide 139 in complex with LecB revealed additional interactions of the aglycon with the protein when compared with the crystal structure of α-amide 140 in complex with LecB (Fig. 25E and F). Attractive interactions of the aglycon can only be established for the β-anomer, explaining at least in part the higher binding affinity while the increased binding entropy probably results from favourable release of water from the protein surface. ADME/Tox evaluation of these compounds revealed good metabolic stability in mouse and human plasma, as well as in presence of liver microsomes from both species. Also, plasma protein binding was generally low and all compounds have been further analysed for cytotoxicity against 3 cell lines *in vitro*. Interestingly, a differential picture of cytotoxicity for some derivatives was found with HepG2 cells (human liver), while the compounds were generally non-toxic against CHO (hamster ovaries) and A549 (human lung) cells.

As for LecA, most multivalent LecB-targeting ligands were analysed for lectin binding only. Some were tested further and the tetravalent *C*-fucoside FD2 by Reymond *et al.* presenting ligand 127 has shown good potency in antibiofilm experiments^492^ and restored antibiotic activity *in vitro*.^499^ The current development status of FD2 is unknown. In addition, calixarene-mounted fucosides by Vidal *et al.* have been tested in an acute lung infection model with *P. aeruginosa* in mice and showed efficacy in preventing lung colonisation and reducing lung injury, their current development status is also unknown.^476^

##### 3.1.4.4 The lectins from Burkholderia cenocepacia

The *Burkholderia cepacia complex* comprises several species of human pathogenic bacteria which often cause lung infections, for example in cystic fibrosis (CF) patients.^500^ These bacteria express a number of lectins of which the β-propeller lectin BambL from *Burkholderia ambifaria* and two LecB-homologs, BC2L-A and BC2L-C, have been structurally characterized and inhibitors are being developed.

**Fig. 26:**
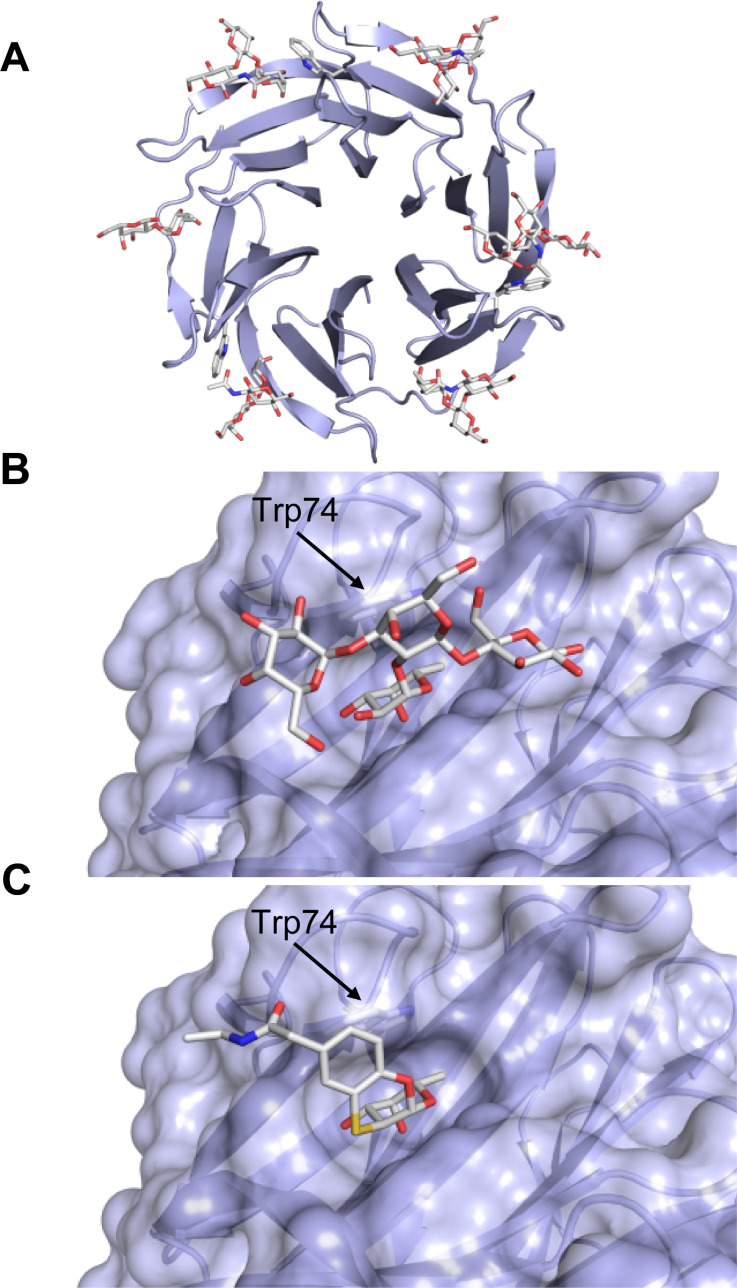
Quarternary structure and binding site of BambL. (A) BambL forms a homotrimer resulting in a β-propeller fold with two slightly different types of fucose binding sites per monomer, one site inside one monomer and one site between two monomers (PDB code 3ZW2). Magnified binding site within one monomer occupied by (B) blood group B antigen (PDB code 3ZWE) or (C) glycomimetic 141 (PDB code 6ZFC).

**Fig. 27:**
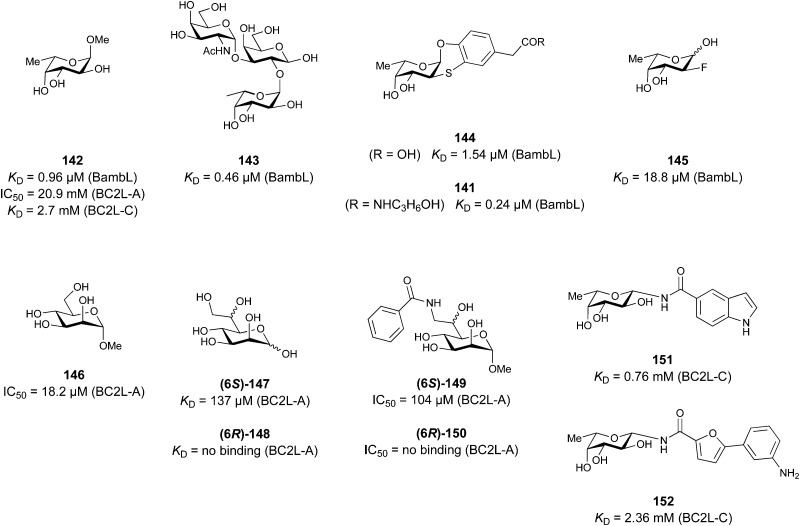
Inhibitors of the *Burkholderia* lectins BambL are derived from fucose, whereas BC2L-A inhibitors derive from mannose. BC2L-C consists of two domains, the N-terminus binds fucosides and the C-terminus is homologous to LecB and binds to mannosides.

BambL is a six-bladed beta propeller lectin consisting of a trimeric repeat of its 87 amino acid polypeptide.^501^ The protein binds fucose with high affinity in two distinct binding sites per repeat, one within one protomer and one at the interface between two protomers (Fig. 26). In contrast to LecB, ligated fucose is deeply buried in BambL with an interaction of its lipophilic α-face with the side chain indole of Trp74, a recognition motif commonly observed in galectins. The *K*_D_ for Me-α-l-Fuc (142) is 0.96 μM and the best known ligand of BambL is blood group A trisaccharide (143, *K*_D_ = 0.46 μM) (Fig. 27). Richichi *et al.* developed the bicyclic fucose derivative 144 which showed very potent binding to BambL with a *K*_D_ of 1.54 μM. This compound was also tested with LecB but no binding was observed. Further, the compound was also unable to inhibit DC-SIGN, suggesting a selectivity of this fucomimetic for BambL.^502^ Extension of the molecule at its carboxylic acid group to 3-hydroxy propylamide resulted in 141 with 240 nM affinity for BambL.^503^ Later, a virtual screening was performed with BambL and two ligands were experimentally confirmed.^504^ One of these was the glycomimetic 2-deoxy-2-fluoro fucose 145 with an IC_50_ of 19.9 μM (*K*_D_ = 18.8 μM) and selectivity over LecB (IC_50_ > 1 mM).

The *bclBCA* gene cluster encodes for three lectins BC2L-A, BC2L-B and BC2L-C. Their names were chosen due to their homology to LecB previously called PA-IIL. These three *Burkholderia* lectins are adhesins and play a role in biofilm development as reported by Eberl *et al.*, who demonstrated that a knock-out of this gene cluster in the CF isolate *B. cenocepacia* H111 resulted in hollow biofilm microcolonies when grown under flow conditions.^505^ Furthermore, it was shown that reversion of the *bclBCA*-deficient strain's biofilm phenotype required complementation with all three genes, while *bclA*, *bclB*, or *bclAC* alone were insufficient.

**Fig. 28:**
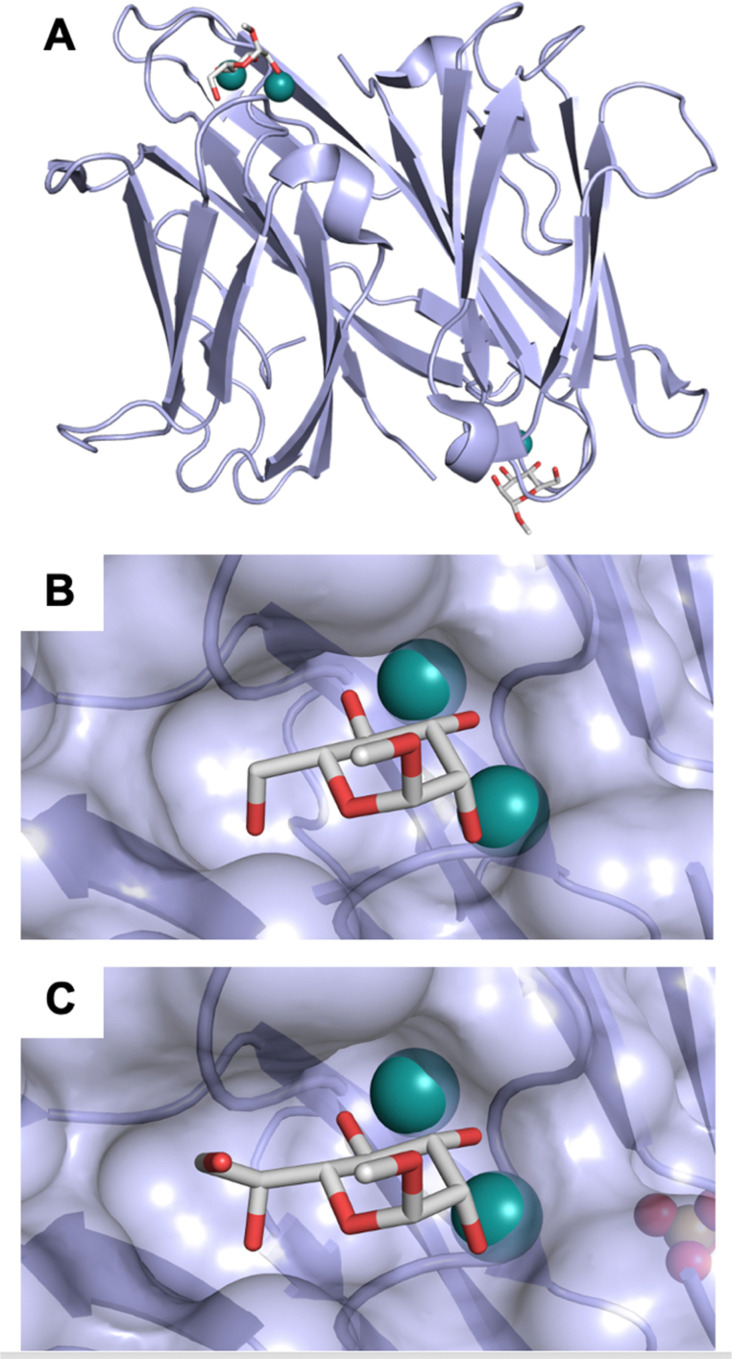
Quarternary structure and binding site of BC2L-A. (A) BC2L-A forms a homodimer with the carbohydrate binding sites oriented in opposite directions (PDB code 2VNV). Magnified binding site occupied with (B) methyl mannoside 146 (PDB code 2VNV) or (C) mannoheptose 147 (PDB code 4AOC). Despite being a LecB ortholog, BC2L-A shows a pronounced selectivity for mannosides over fucosides explained by hydrogen bonding of the mannose 6 OH. Calcium ions are depicted as green spheres.

The structure of BC2L-A has been solved as a dimer and it was shown that this protein preferably binds mannosylated glycans (Fig. 28).^506,507^ In contrast to its homolog LecB, the IC_50_ of Me-α-d-Man 146 with BC2L-A is 18.2 μM, while the corresponding value for Me-α-l-Fuc 142 is 20.9 mM, indicating a 1000-fold selectivity of this lectin for mannosides. These observations can be explained by the crystal structure of the complex with Me-α-d-Man: the 6-OH of mannose deeply enters the protein (Fig. 28B) and is fully hydrogen-bonded to Ala30, Glu31 and Asp110. Since fucosides have no analogous hydroxy group, the 6-OH group is the main driver of the selectivity of BC2L-A. In addition, the side chain of His112 could be responsible for a steric clash with the aglycon in fucosides, further contributing to selectivity. It was shown by Imberty, Silipio and co-workers that BC2L-A also binds with good potency to heptoses present in bacterial lipopolysaccharides.^508^ The l-*glycero*-d-manno-heptose 147 binds to BC2L-A with a *K*_D_ of 137 μM (Fig. 28C) while the diastereomeric d-*glycero*-d-manno-heptose 148 is not recognized, demonstrating the impact of the stereochemistry of the 6-OH group. A series of synthetic mannose and mannoheptose analogues was analyzed for their BC2L-A inhibition.^509^ In this study, the essential role of this OH group was further confirmed by substitution with the halogens Cl, Br, or I and with an amino group, none of these derivatives inhibited BC2L-A. Also, when the ring hydroxy groups were exchanged with fluorine or altered by *O*-methylation, a loss of activity was observed. Interestingly, the glycomimetic (6*S*)-mannoheptoses with amide and especially sulfonamide substituents at C7 were superior inhibitors of BC2L-A (IC_50_ = 13.8–116 μM, *e.g.*149) while the (6*R*)-diastereomers, *e.g.*150, were inactive, consistent with the data for unmodified heptoses by Marchetti *et al.*

**Fig. 29:**
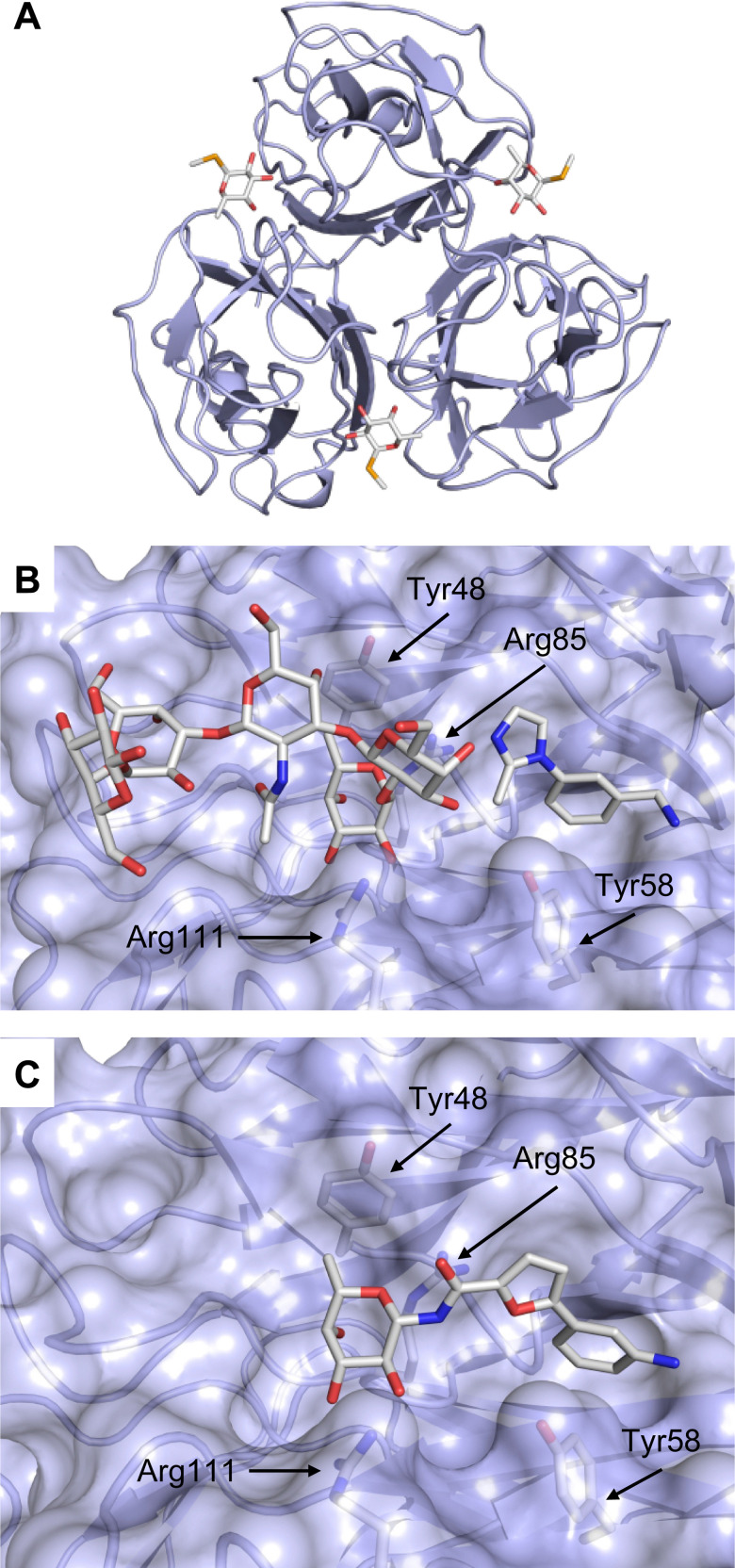
*B. cenocepacia* BC2L-C is a lectin composed of an N-terminal fucose-binding domain and a C-terminal LecB-orthologous mannose-binding domain. (A) The N-terminal domain of BC2L-C trimerizes and binds fucosides at the interface of two monomers (PDB code 2WQ4), (B) a bound fragment was identified in a second site of BC2L-C N-terminal domain adjacent to the CRD ligated with the oligosaccharide Globo H (PDB code 6ZZW), (C) β-fucosylamide glycomimetic 152 cocrystallized with BC2L-C N-terminal domain (PDB code 7OLW). Residues involved in binding to fucose are highlighted: Tyr48 forms CH–π interactions with Fuc-C6, Arg85 coordinates to Fuc-4-OH and -5-O, Arg111 binds Fuc-2-OH and -3-OH. The fragment in (B) and the aglycon in (C) is recognized in a hydrophobic cleft and forms attractive interaction with Tyr58.

BC2L-C was also crystallised^510,511^ and this protein consists of two domains: an N-terminal TNF-like lectin domain and a C-terminal LecB-domain, which then assemble into hexamers. Small angle X-ray scattering and transmission electron microscopy data allowed to postulate a model where the hexamer consists of two trimeric N-terminal domains (Fig. 29A). and three dimeric C-terminal domains. This complex heterobifunctional superlectin was shown to possess proinflammatory activity and to stimulate IL-8 expression in cell culture. The N-terminal domain of BC2L-C binds a diverse set of fucosylated oligosaccharides with LewisY and H-type 1 antigen as most potent binders (*K*_D_s of 53.9 and 77.2 μM, respectively) and the Lewis blood group antigens LeA, LeB, and LeX have *K*_D_s of 132, 213, and 196 μM. Its affinity for Me-α-l-Fuc is significantly lower (*K*_D_ = 2.7 mM). In contrast, the C-terminal domain is homologous to BC2L-A and binds selectively (high)mannose, mannoheptose and conjugates thereof. Affinities were determined by ITC for mono-, di-, and trisaccharides and *K*_D_s ranged from 27.6 to 88.1 μM. Because BC2L-A was shown to bind to *B. cenocepacia* cells, BC2L-C was postulated to crosslink bacteria and host cells *via C*- and N-terminus, respectively.

In the N-terminal domain of BC2L-C, a second site has been identified in close proximity to the fucose binding site.^512^ After a large virtual screening for this second site, shortlisted fragments have been analysed for binding to BC2L-C N-terminus by differential scanning fluorometry and STD-NMR. Finally, a co-crystal structure has been obtained of this domain in complex with the tetrasaccharide Globo H and one fragment residing in the second site (Fig. 29B). These results set the stage for the development of fucose-derived glycomimetics where fucose and fragments have been linked *via* different linker chemistries. Fucosylamide 151 with an attached indole carboxamide showed an affinity of 0.76 mM, which is a >3-fold improvement compared to MeFuc with 2.7 mM.^513^ Glycomimetic 152 could be cocrystallized with BC2L-C, demontrating the tight binding of the aglycone in the second site (Fig. 29C). Interestingly, these molecules are structurally similar to the very potent LecB inhibitors 138 and 139 and might therefore constitute a class of glycomimetics that simultaneously fight two pathogens which often co-infect patients.

#### 3.1.5 Monovalent influenza virus hemagglutinin inhibition

Many viruses employ carbohydrate receptors on host tissue for infection.^514^ Since all human and animal tissue is highly glycosylated and the resulting glycocalyx covering each cell often carries a high density of glycans, these epitopes serve as recognition motifs for viral lectins.

Sialic acids are negatively charged carbohydrates based on 9-carbon atom α-ketoacids and *N*-acetyl neuraminic acid (Neu5Ac) is the most abundant sialic acid monosaccharide in humans that also exists with distinct chemical modifications, *e.g.* acetylation.^343^ These sialic acids are frequently used as primary receptors by many viruses for establishing infection, among them are influenza viruses, some coronaviruses and many others.^515–517^ Therefore, the design of inhibitors of viral agglutinins is a well-studied field, which is however relying almost exclusively on the multivalent display of native Neu5Ac to exploit the avidity of the trimerizing influenza hemagglutinin.^518,519^ In a recent example, a large multivalent display of Neu5Ac with additional neuraminidase inhibitors has been designed to wrap around the viral particle and hereby block infection.^520^ In influenza A, these hemagglutinins differ in their sequence, which results in numerous serotypes of the influenza virus.^514^ These differences also impact on the recognition of the Neu5Ac moiety in its natural glycoconjugates, as demonstrated by the selectivity for α-2,3-linked glycans in avian influenza and for α-2,6-linked Neu5Ac in human influenza.

**Fig. 30:**
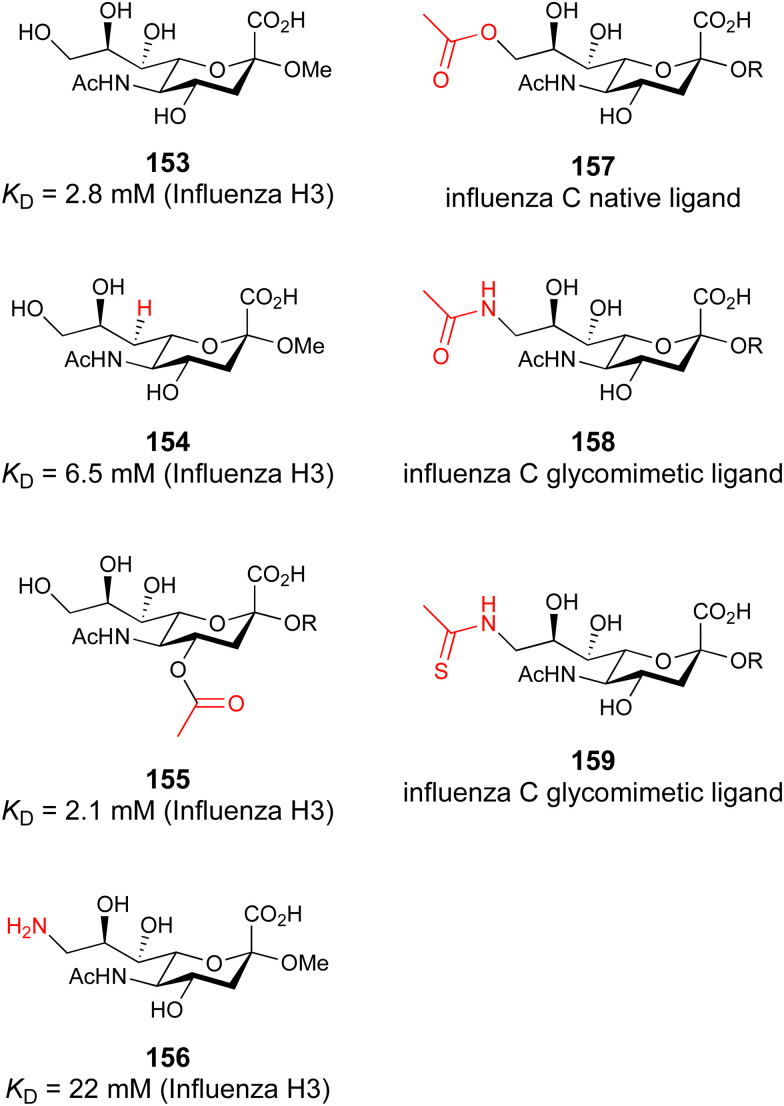
Glycomimetics of Neu5Ac as inhibitors of viral hemagglutinins from influenza A and hemagglutinin-esterase from influenza C.

An early study on modifying the Neu5Ac residue itself and probing its interaction with influenza hemagglutinin H3 was performed by Sauter *et al.*^521^ In this important study, it was shown for the methyl glycoside of Neu5Ac (153) that its non-interacting 7-OH could be removed in 154 without impact on binding to H3 and modifying 4-OH by esterification (155) or 9-OH by transforming into an amine (156) was possible (Fig. 30). Binding of these modified monosaccharides was in the low mM range and thus comparably potent to the parent methyl glycoside of Neu5Ac, 153. It was also observed that removal of the hydrogen bond donor in position 9 by acetylation entirely abolished binding (*K*_D_ > 100 mM). Further, acetylation of 7-OH or exchange of the acetamide with an azide also abolished recognition by H3. Thus, this work provided an early structure–activity relationship for Neu5Ac recognition in one influenza hemagglutinin.

In contrast to influenza A hemagglutinin H3, the influenza C hemagglutinin–esterase fusion protein recognises 9-*O*-acetyl Neu5Ac conjugates 157 as its native ligands and substrates. Paulson and co-workers have reported the synthetic 9-acetamido 9-deoxy Neu5Ac derivative 158 that was enzymatically conjugated for display on erythrocytes. Cells carrying this glycomimetic conjugate then successfully agglutinated influenza C,^522^ while influenza A or B were not agglutinated. A small structure activity–relationship study showed that erythrocytes carrying Neu5Ac derivatives with position 9 modified as azide, amine or hexanoyl amide did not agglutinate influenza C. This work was further extended by demonstrating that the 9-deoxy 9-thioacetamido Neu5Ac 159 is indeed a glycomimetic for influenza C hemagglutinin function but prevents further processing by the esterase function of the fusion protein.^523^

The knowledge obtained from these SAR studies on glycomimetics of Neu5Ac for hemagglutinins will allow the generation of receptor/strain specificity of inhibitors. The mandatory high affinity for efficient viral trapping will be achieved after multivalent display of those glycomimetics.

## 3.2 Novel glycomimetic scaffolds for Ca^2+^-dependent lectins

In the early 2000s, the Kiessling group designed one of the first non-carbohydrate glycomimetics for DC-SIGN. The aim was to mimic the canonical carbohydrate recognition mediated by a central Ca^2+^ ion using a shikimic acid-derived scaffold.^524–527^ This core structure was supposed to provide a collection of diverse ligands for targeting a range of C-type lectin receptors. Interestingly, the shikimic acid-derived scaffold targeted the carbohydrate-binding site of DC-SIGN through the hydroxy groups at positions 3 and 4 similarly to mannose. Thus, it has been considered as a good mimetic of mannose for Ca^2+^-dependent lectins. Additionally, the carboxylic acid and thiols offer two potential points of diversification, which were exploited to introduce different substituents and to synthesize a library of 192 compounds. Their ability to compete with immobilized mannan for binding to the fluorophore-labelled extracellular domain of DC-SIGN was tested in a fluorescence-based competition assay. The best shikimic acid–based glycomimetic of the library showed only weak affinity for DC-SIGN (18, Table 4, IC_50_ = 3.2 mM^525^). However, the potency of the glycomimetic was later enhanced by three orders of magnitude following multivalent presentation (IC_50_ = 2.9 μM^527^).

**Table 4:** Novel glycomimetic scaffolds for Ca^2+^-dependent lectins and their binding affinities

No	Structure	Protein	Binding affinity	Ref.
18	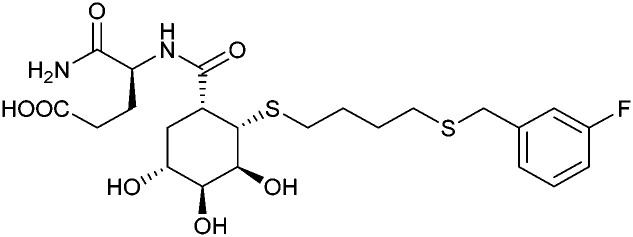	DC-SIGN	IC_50_ = 3.2 mM	Garber *et al.*^525^
161	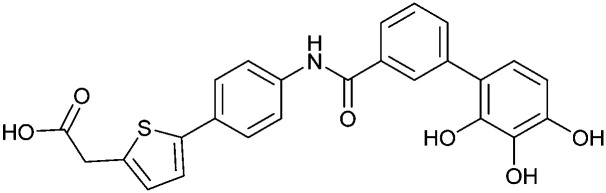	P-selectin	IC_50_ = 0.57 μM	Kranich *et al.*^528^
16	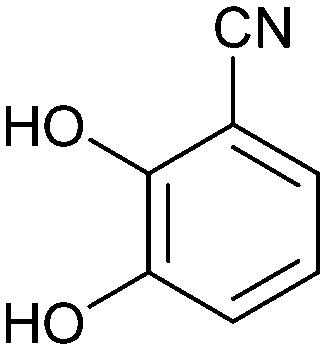	LecA	*K*_D_ = 1.11 ± 0.07 mM	Kuhaudomlarp *et al.*^121^
160	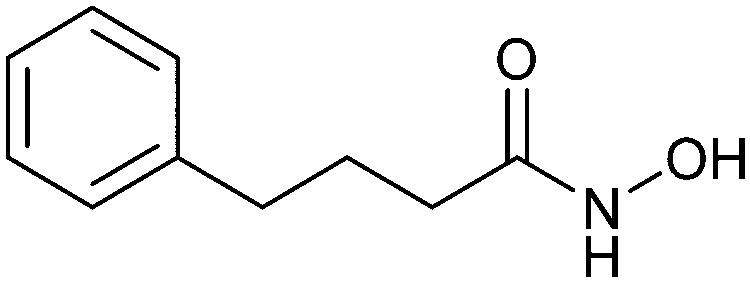	LecA	*K*_D_ = 4.6 ± 0.9 mM	Shanina *et al.*^122^
162	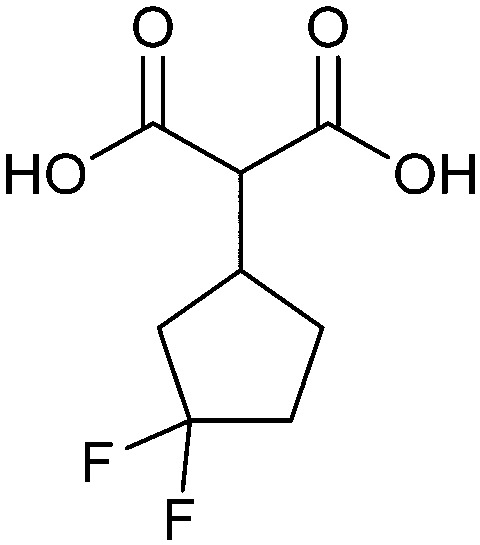	LecB	*K*_D_ = 1.2 ± 0.4 mM	Shanina *et al.*^122^
		DC-SIGN	*K*_D_ = 1.2 ± 0.5 mM	

Another class of glycomimetics was discovered for P-selectin by Revotar.^528^ A rational design approach led to the development of non-carbohydrate P-selectin antagonists mimicking sLe^X^. These glycomimetics contain the hydroxy groups in positions 4, 3 and 2 of an attached pyrogallol, intended to mimic the hydroxy groups of fucose. A comprehensive structure–activity relationship study provided the upper range nM inhibitor of P-selectin 161 (Table 4, IC_50_ = 0.57 μM), which showed high inhibition values up to 83% in the HL-60 cell attachment assay.

**Fig. 31:**
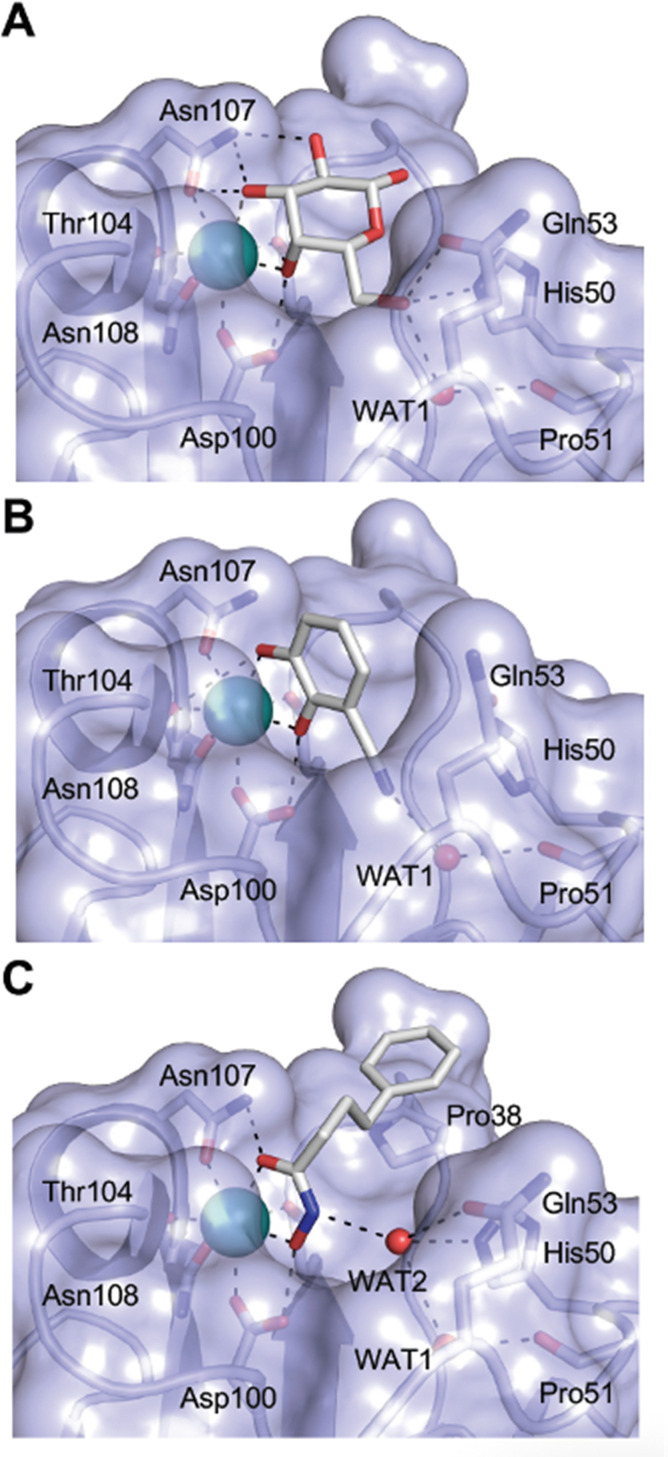
Non-carbohydrate glycomimetics for the Ca^2+^-dependent lectin LecA. Crystal structures reveal the interactions between LecA and (A) galactose (PDB code 1OKO), (B) catechol 16 (PDB code 6YO3), and (C) hydroxamic acid 160 (PDB code 7FJH). The non-carbohydrates 16 and 160 bind to LecA *via* calcium complexation in similar fashion to the native ligand galactose. Calcium ions depicted as green spheres and tightly coordinated water molecules as red sphere.

A further example of targeting a Ca^2+^ dependent lectin using non-carbohydrate small molecules has been recently reported for LecA from *P*. *aeruginosa*.^121^*In silico* screening of the National Cancer Institute (NCI) Diversity IV database and validation of hits by several biophysical assays identified catechols as ligands taking the canonical coordination of the central Ca^2+^ ion in this lectin. Notably, this compound class is also known as part of the pan-assay interference substances (PAINS^529^), which are prone to undergo unspecific interaction with proteins.^530^ Despite the fact that catechols can be oxidized to reactive quinones, Imberty, Titz and co-workers proved that electron-deficient catechols are stable under the conditions tested and in their interaction with LecA. Thus, catechols are not necessarily ‘bad actors’. A crystal structure in complex with LecA confirms the catechol 16 as a mimic of carbohydrates in Ca^2+^-binding. Similar to the LecA–galactose complex (Fig. 31A), the catechol coordinates the Ca^2+^ ion through two vicinal hydroxy groups, which mimic the 3 and 4 hydroxy groups of galactose (Fig. 31B). In particular, this catechol derivative forms H-bonds with Asn107 and Asp100 and the backbone oxygen of Tyr36. Even though this exemplifies that small molecules can coordinate the ions in the carbohydrate-binding site of a lectin, the Ca^2+^ coordination alone is not responsible for the binding. The nitrile group in catechol 16 plays a crucial role and mimics the hydroxy group of galactose in position 6. It uses a conserved water molecule (WAT1) to form a H-bond bridge between the nitrogen atom of the nitrile group in catechol and the oxygen atom of the carbonyl group of Pro51 in LecA. Its removal or shifting to the neighbouring carbon atom abrogated or decreased the binding efficiency measured by SPR and the inhibition in a fluorescence polarization (FP) assay, respectively. Despite the millimolar range binding affinities of catechols (16, Table 4), they bind tightly enough relative to their size (MW of 135–155 g mol^−1^) and number of heavy atoms (HA).^531^ Given the fact that catechols show ligand efficiency (LE) values of 0.4, they provide a good basis for future compound growing to increase their binding potency for LecA. Moreover, it was shown that catechols may target the carbohydrate-binding site of other Ca^2+^-dependent lectins, such as langerin. However, the mechanism of action remains to be confirmed for CTLs. Overall, electron poor catechols challenge the paradigm of ‘undruggable’ lectins as non-carbohydrate glycomimetics and show the potential of the structure-based design studies with the aim to improve their potency and drug-like properties as potential antimicrobial agents.

In another attempt to identify non-carbohydrate pharmacophores for Ca^2+^-dependent lectins by Rademacher *et al.*, an NMR-based screening of a chemically diverse library of 1000 fragments identified a compound with a hydroxamic acid moiety that coordinates the Ca^2+^ ion in the carbohydrate binding site of LecA (160, Table 4).^532^ The structure–activity relationship (SAR) study revealed a sterically optimal presentation of the hydroxamic acid group as demonstrated by a crystal structure of LecA in complex with 160 (Fig. 31C). Similar to galactose and catechols, the hydroxamic acid moiety coordinates to the Ca^2+^ ion. In particular, the nitrogen and the two oxygen atoms form H-bonds with His50, Gln53, Pro51 *via* a water molecule (WAT2) and Asn107, Asp100 mimicking the hydroxy groups of galactose in positions 6, 4 and 3, respectively. Notably, the phenyl moiety of the hydroxamic acid compound 160 forms CH–π interaction with Pro38 that is not present in catechols.

Inspired by the novel scaffolds, Rademacher and co-workers explored the application of a metal-binding pharmacophore (MBP) library aiming to improve the targeting of Ca^2+^-dependent lectins.^122^ A ^19^F NMR screening was performed to compare the hit rates of three fragment libraries against four Ca^2+^-dependent lectins (LecA, LecB, langerin and DC-SIGN). Notably, the MBP library showed superior hit rates compared to previous screening attempts against these targets. In particular, 1D and 2D NMR studies demonstrated the potential of a malonic acid scaffold in targeting the carbohydrate-binding sites of LecA, LecB and DC-SIGN (162, *K*_D_ = 1.2 mM for LecB and DC-SIGN, Table 4). Even though the group has demonstrated the Ca^2+^-dependency of these interactions, the binding mechanism remains to be confirmed by crystallography studies.

Altogether, three non-carbohydrate glycomimetic scaffolds and an MBP library have been proposed to improve the targeting of the ‘undruggable’ site of the Ca^2+^-dependent lectins. Since these molecules are small (MW < 300 Da) and coordinate the Ca^2+^ ion, they are promising starting structures for the design of drug-like non-carbohydrate glycomimetics of lectins.

### 3.3 Allosteric modulation of lectins

Allosteric modulators have gained increasing interest in the development of selective and potent agonists and antagonists of protein function. While many drug targets share a common architecture of the primary active site with other members in their protein family, allosteric sites are less conserved between members of the same protein family and hence likely offer a better starting point for the reduction of off-target effects. Additionally, allosteric drugs can provide novel ways of action, such as changing protein levels, localization within the cell or even target activation.^533–535^ In this interplay between distal target sites, the mechanism by which orthosteric and allosteric site are coupled can be diverse ranging from larger protein rearrangements to alteration of vibrational modes of amino acid side chains that promote reciprocal interaction of the two sites.^536^ The concept of allostery in drug design has been widely and successfully explored for GPCRs and other intrinsically dynamic, allosteric drug targets and is more and more applied to other targets such as kinases, phosphatases and other protein classes with low selectivity.^537,538^ For carbohydrate-binding proteins, the concept of allostery in the design of non-carbohydrate glycomimetics has been investigated only sparsely, giving rise to new opportunities for the future. Allosteric sites can offer favourable properties for the development of functional glycomimetics, such as better physicochemical properties and the absence of competition with endogenous ligands, as highlighted for Siglecs (see Section 3.1.3). Here, we summarize information on allosteric mechanisms, how they were identified and how, if at all, they were put into the design process.

#### 3.3.1 Bacterial lectins

**Fig. 32:**
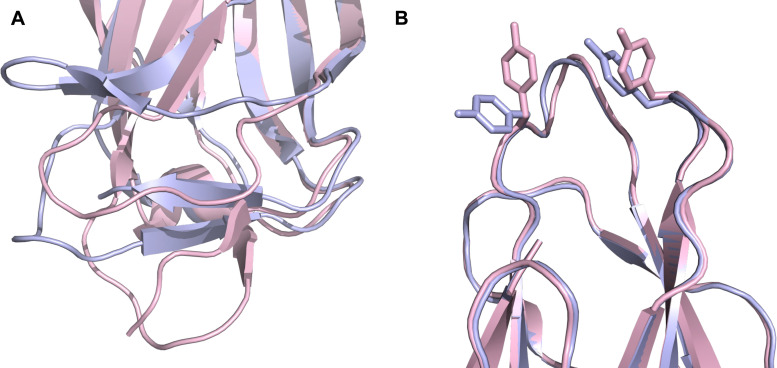
FimH allostery. The close-up of the low affinity (light blue) and high affinity (light pink) conformation of FimH at the domain interface (A) reveals significant conformational differences which affect the carbohydrate binding site (B) *via* long-range effects. In detail, this results in alterations of amino acids sidechains, in particular Tyr48 and Tyr137 (shown as stick), in the carbohydrate binding site. Figure adopted from lit.^546^

In the multidomain protein FimH, the anchoring (fimbrial) domain connects the lectin FimH domain to the fimbrium. The interdomain interaction between the two domains introduces a twist in the sandwich fold of the mannose-binding lectin domain, which reduces affinity and locks the adhesin in the low-affinity state. Upon the application of force, the two domains separate and the twist of the beta sheet is removed. Consequently, mannose is recognized with higher affinity.^389^ This is an interesting observation since most receptor–ligand interactions break under force or high flow conditions, while FimH increases association under increasing tensile mechanical force. This phenomenon is known as catch bond and describes in this specific case FimH as a protein that undergoes larger conformational rearrangements introduced by force of its natural environment in the bladder (Fig. 32).^392,539^ These rearrangements were studied using single point mutations combined with conformational-state specific antibodies directed against allosteric sites distal from the mannose recognition site.^540,541^ These antibodies have clearly shown that different states can be locked, a great utility to study FimH allostery. In particular, the antibody mab21 stabilizes the tight conformation by binding a loop away from the orthosteric site, interfering with the interdomain interaction to unlock the high affinity state.^542,543^ This overall allosteric behaviour under mechanical regulation was further confirmed by the introduction of a disulfide bridge that converted the high affinity into a low affinity state.^544^ Taken together, instead of following the design of high affinity mannose-based glycomimetics targeting the carbohydrate binding site, allosteric antagonists stabilizing the low affinity conformation of FimH lectin domain might be a viable alternative for therapy.^44,545^

**Fig. 33:**
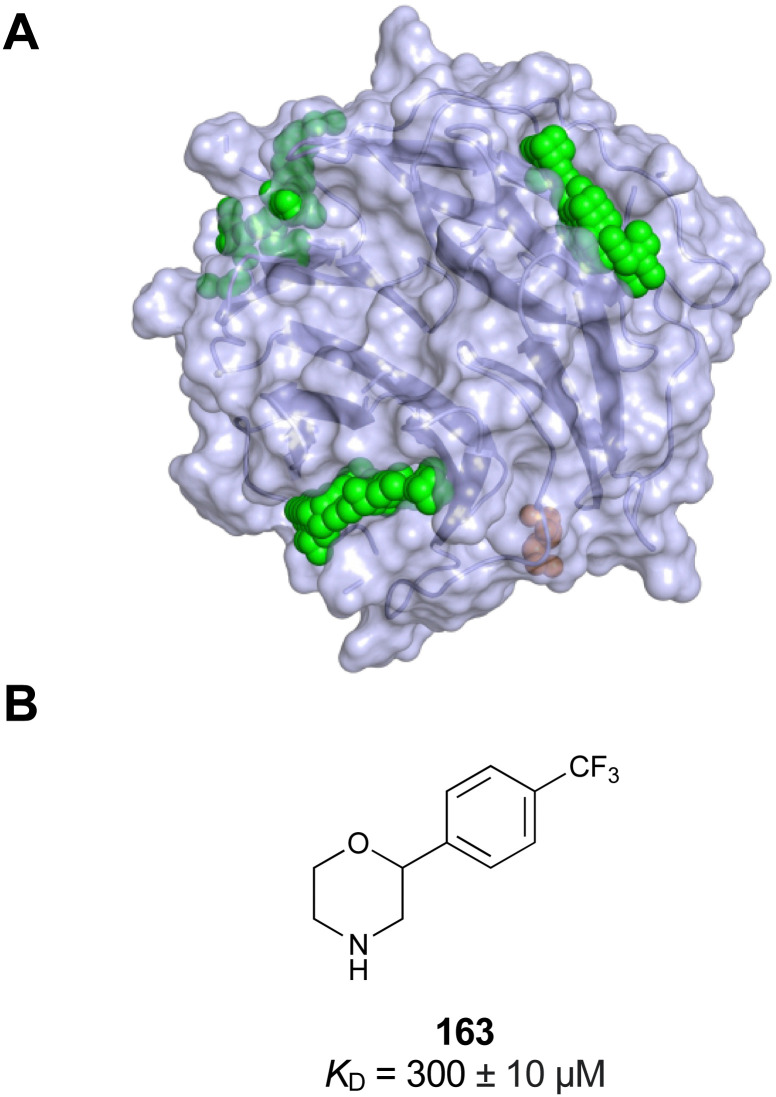
Surface representation of BambL (A, PDB code 3ZW0) and the binding region of fragment-like molecules such as 163 (B). The proposed allosteric pocket is indicated by green spheres. Carbohydrate-binding site shown in orange.^125^

Compared to FimH, allostery is less apparent and a less explored phenomenon for other bacterial lectins. Cholera toxin is an AB5 toxin with a lectin domain promoting cell tropism and uptake. This lectin domain is well studied and its recognition of the ganglioside GM1 has been described in detail. However, a second carbohydrate binding site has been discovered and this site is proposed to be allosterically coupled to the primary site.^547^ To the best of our knowledge this has not been followed for the design of glycomimetics. In contrast, coming from a fragment-based screening approach, several sub-millimolar inhibitors of BambL have been recently identified.^548^ BambL is a lectin of *B. ambifaria*, causing chronic infections.^501^ Evidence from biophysical analysis and single point mutations introduced into the protein suggest an allosteric mode of action. A binding site was identified distal to the orthosteric fucose recognition site (Fig. 33). In this study, ^1^H–^15^N HSQC data are presented showing that changes of the chemical environment in secondary sites induced either by weak small molecule binding or by mutation are transferred to remote sites in the protein.^125^ Higher affinity ligands are necessary to move these allosteric glycomimetics into further assessment.

#### 3.3.2 Galectins

**Fig. 34:**
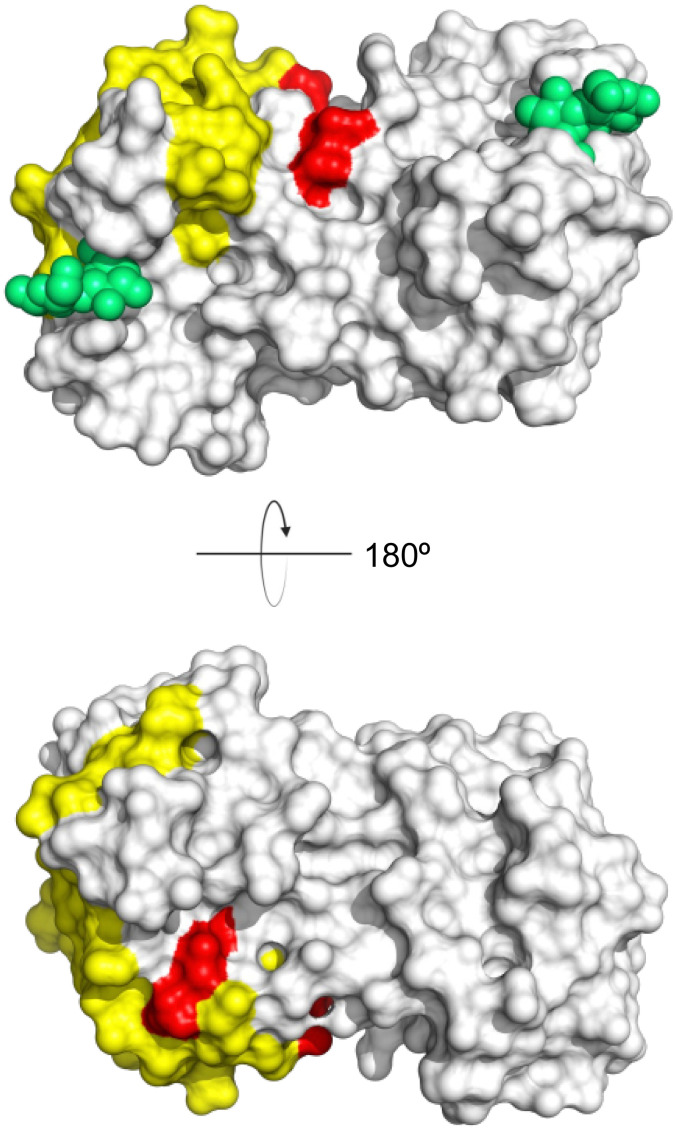
Surface representation of the binding region of allosteric modulators for Gal-1. Residues undergoing chemical shift perturbation upon 62 binding are shown in red.^127^ Yellow indicates amino acids interacting with DB16 (61) at the edge of the monomer.^128^ Carbohydrate ligand indicated by green spheres. Structures of 61 and 62 are shown in Table 2.

**Fig. 35:**
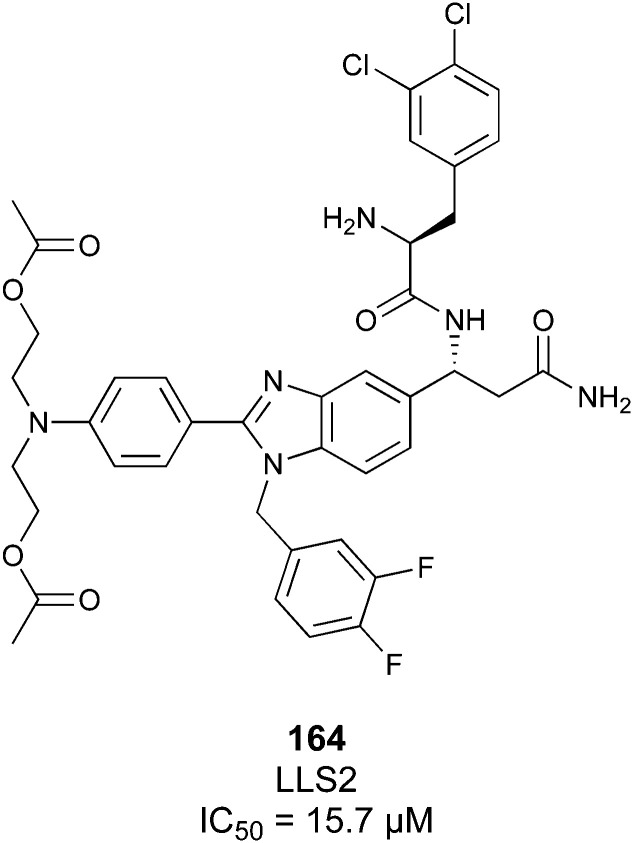
LLS2 (164) is a proposed allosteric inhibitor of Gal-1 with a reported inhibitory constant from an *in vitro* cell killing assay using SKOV3 cells.^550^

For the galectin family, the development of allosteric glycomimetics initiated not with the existing knowledge on protein flexibility but rather with the discovery of anginex (59, Table 2), a 33-mer cytokine-like artificial peptide binding to Gal-1 with three to four orders higher affinity compared to small carbohydrate ligands.^305,307^ 6DBF7 (60) and two more potent peptide mimetics DB16 (61) and DB21 followed. For DB16 (61), the binding site was inferred from ^1^H–^15^N HSQC experiments being at the edge of the Gal-1 monomer, strongly suggesting an allosteric inhibition mechanism (Fig. 34).^128^ The calixarene PTX008 (62) was identified from screening a smaller library of topomimetics of anginex (59) and recruits its anti-angiogenic and antitumor activity from Gal-1 binding.^127,549^ Data suggested that this inhibitor binds to the back face of the β-sandwich, allowing attenuation of lactose binding.^549^ For the recently reported small heterocyclic molecule LLS2 (164, MW = 808 Da, Fig. 35) identified from a one-bead two compound library screening, a non-confirmed allosteric mechanism was hypothesized for Gal-1 inhibition.^550,551^ Taken together, the allosteric mechanism underlying Gal-1 inhibition needs further exploration, but data suggest that common characteristics of the galectin fold could be involved, such as the modulation of the β-sheet or the interference with dimer formation.

**Fig. 36:**
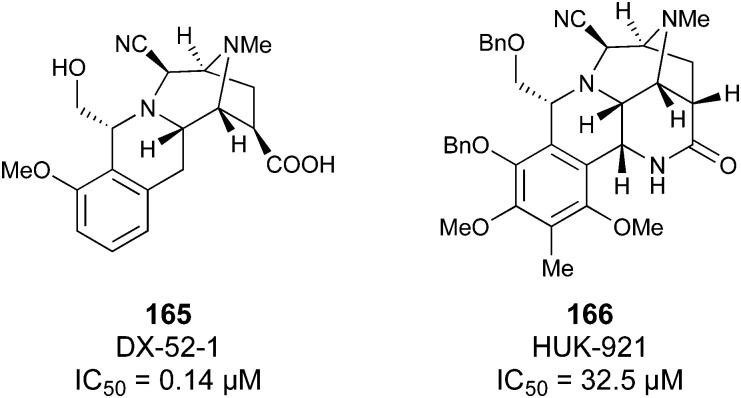
Allosteric Gal-3 inhibitors DX-52-1 (165) and HUK-921 (166) with reported inhibitory constants from epithelial cell migration assays.^552,554^

The notion that galectins are amenable to allosteric modulation is further supported by two reported Gal-3 inhibitors, both analogues of tetrahydroisoquinoline natural products (Fig. 36).^552^ DX-52-1 (165) was suggested to covalently bind to Gal-3 and both compounds bind Gal-3 outside of its β-galactoside-binding site and inhibit cell migration. The exact binding site is not reported. In light of these potentially allosteric inhibitors for Gal-1 and -3, it is not surprising that also for Gal-7 allosteric communication was described. Longer-range communication of loops 1, 2 and 5 perturbing protein–protein interaction was suggested from protein engineering and biophysical characterization.^553^

#### 3.3.3 C-type lectins

In contrast to the galectins, for CTLs it was already reported prior glycomimetic development that this lectin family harbours intrinsic dynamics in their protein structure. Carbohydrate recognition is Ca^2+^ dependent, which in turn is associated with pH sensitivity provided by sensitive amino acids close to or in the active site as well as concerted loop mobilities for some receptors.^555–559^*cis* to *trans* isomerization of the canonical proline of the EPN/QPD motif may prevent cargo rebinding by slowing down these dynamics^150,555,560–562^ and CTL oligomerization modulates the avidity of some of the receptors – a dynamic process tied to the cell physiological environment of the CTL. Endocytic pH has been reported to initiate loss of oligomerization for some CTLs such as DC-SIGN. Only a minority of the members of the CTL family recognize carbohydrates^142^ and of those, two are reported to do so in a Ca^2+^-independent manner utilizing a secondary site remote from the canonical site, pointing towards proximal functional sites being available in this fold.^154,155^ Hence, it is not surprising to find functional secondary sites being reported in CTLs as well.^158,167,215,563–565^ For E-selectin, larger multidomain rearrangements are coupled to carbohydrate recognition.^149^ Even though the structural biology and dynamics of CTLs are well documented for individual cases, these insights were rarely leveraged for drug discovery. As with many drug targets, allostery came into the design process by mode of action analysis of an existing series of compounds rather than a focused search,^566,567^ and allostery emerged late as a potential route for the modulation of CTLs.^568,569^ In the following paragraphs we will give a few examples how allostery influenced the glycomimetic design process targeting CTLs, how it was discovered by unfocused screening and mode of action analysis.

Selectins are type I membrane proteins that mediate cell adhesion, assist in leukocyte tethering and rolling, and exhibit a catch bond behaviour with a shear threshold feature.^570–574^ For P-selectin two states, having a bent or extended EGF orientation, are reported. These states undergo dynamic conformational exchange, sampled by forced induced allostery, initially thought to be limited to the interaction of the CRD and the adjacent EGF-like domains.^575^ Later, these insights were extended into long scale allostery over the entire protein structure, now including the consensus repeat (CR) domains.^576^ Knowing that such long-range interactions exist has expanded our view on potential functional sites for the development of allosteric glycomimetics. This opens doors for modulation of the carbohydrate recognition process with new chemical matter.

A good example of how protein dynamics and allostery are connected to the design of a carbohydrate-based glycomimetic is sLeX (26) recognition by E-selectin (Fig. 7). These data became available when the pan-selectin antagonist GMI-1070 (31) was already in Phase II trials.^149^ While it was known in the time of development that the first domain was sufficient to allow binding,^577^ affinity increase with increasing number of CR domains was also reported similar to P-selectin.^578^ The breakthrough how long-range force induced allostery can directly relate to carbohydrate-based glycomimetic recognition came from work employing an extended protein construct design and a co-crystallisation approach of 28 rather than soaking of a two-domain construct.^149,167^ This resulted in three regions of the lectin domain being affected upon ligand binding: (1) the long loop structure (residues 81 to 89) rearranging, (2) the so-called bridging region mediating the (3) transition motion originating from the pivot region connected to the EGF-like domain. For the design of the carbohydrate-based glycomimetic, the alteration of the long loop structure by almost 10 Å was most obvious (Fig. 7). While such a large-scale rearrangement is often observed for CTLs upon Ca^2+^ binding and release,^150,555,560–562^ the remarkable observation for E-selectin was that the central fucose got covered by the long loop.^149^ The fucose occupies the canonical carbohydrate site, coordinating the Ca^2+^ with its 3- and 4-hydroxy groups, now also interacting with the long loop, leading to formation of a hydrogen bond of the 2-hydroxy group in the extended conformation of E-selectin. This switch between the open and closed conformation of the loop is suggested to happen spontaneously after ligand recognition.^579^ Moreover, solution state, label-free small angle X-ray scattering (SAXS) data strongly suggest that the structural transition to an extended conformation can be initiated even by small molecule recognition.^149^ These data explained the previously reported, notorious importance of the fucose 2-hydroxy group for the activity of GMI-1070 (31) series and other glycomimetics.^580,581^ In analogy to P-selectin, these new insights into conformational transitions open the path for the development of novel allosteric glycomimetics.

A look at the long loop mobility in the CTL langerin complements the picture that arose from E-selectin. Langerin is a type II transmembrane receptor that forms homotrimers, promoted by the neck domain extending it 24 nm from the plasma membrane. Compared to the type I transmembrane selectins, there is no EGF-like domain, and instead the long helical neck domain of langerin renders E-selectin-like long-range conformational transitions unlikely. Moreover, interdomain interactions between the langerin CRDs can be excluded, as no difference for the carbohydrate affinity of the monomer and the trimer was detected.^582^ Such interdomain interactions have not been reported for other hetero- or homooligomeric CTLs. However, in analogy to E-selectin for which the mobility of the long loop structure was reported, micro- to millisecond dynamics was inferred from NMR studies.^582^ Furthermore, a short loop structure of langerin, called the bridging region for the selectins, is coupled to the long loop mobility.^582,583^ The cleft between the two loops harbours His294, the pH sensor of this CTL.^582,583^ Combining molecular dynamics simulations, NMR and mutational scanning of the CRD, a conserved allosteric network of communicating amino acids was identified for human langerin, which was further substantiated by a Ca^2+^-independent activation upon recognition of larger heparin fragments.^158^ Overall, a similar picture of the coupled loop mobilities emerges for langerin and E-selectin.

**Fig. 37:**
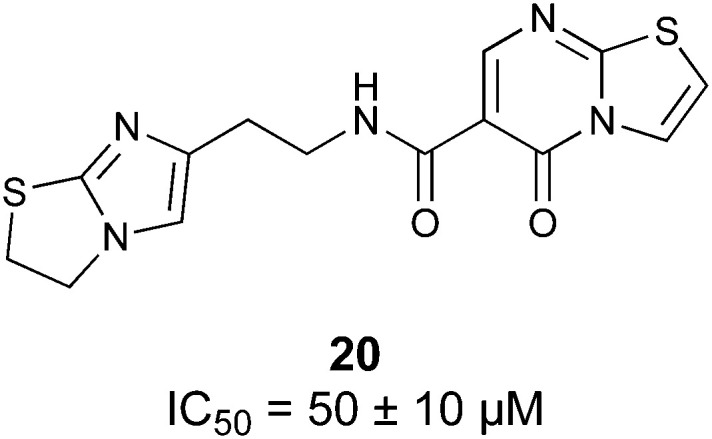
Allosteric inhibitor of murine langerin 20.^126^

**Fig. 38:**
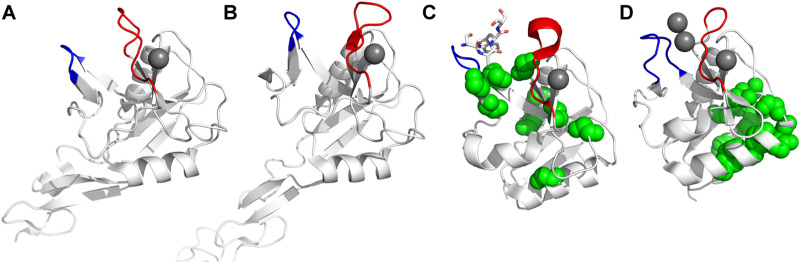
Long loop (red) and short loop (blue) structures in E-selectin, langerin and DC-SIGN. (A and B) Short loop, long loop of E-selectin with (A) open conformation (PBD code 1G1T) and (B) closed conformation (PBD code 4C16). (C) Langerin in complex with a peptide in the cleft (‘bridging region’) between the two loops (PBD code 3P7H).^582^ Amino acids involved in an allosteric network are highlighted with green spheres.^582^ (D) One allosteric site of DC-SIGN is indicated with green spheres (PBD code 1SL4).^162^ Calcium ions are shown as grey spheres.

Taking the next step, a fragment-based design approach was conducted against murine langerin and several allosteric modulators were identified, some such as 20 with two-digit micromolar potency as measured in an inhibition assay in which lectin binding to a modified lipid surface was monitored (Fig. 37).^161^ In recent follow-up on this work, the binding site for this compound class was determined using NMR and computational methods.^584^ The allosteric site of langerin is located in the cleft between the long and short loop, the so-called bridging region (Fig. 38). Moreover, this study suggested long loop mobility upon carbohydrate recognition (Fig. 38A and B).^584^ Whether the inhibition mechanism of the thiazolopyrimidines is based on the interference with the long loop, similar to what has been observed for E-selectin, covering the central fucose, remains to be elucidated. Interestingly, this cleft between the long and the short loop was previously reported to harbour a short peptide, suggesting it is amenable to modulation *via* small molecule inhibitors.^585^ Additionally, the short loop itself is involved in formation of langerin/langerin interactions in the endosomal compartment, forming Birbeck granules, a langerin-specific organelle.^586^ Taken together, several lines of evidence support the notion that allosteric inhibition of langerin through intercalation in the bridging region is possible. The next step will be to develop this lead into higher affinity ligands to explore their utility in functional, cell-based assays and *in vivo* studies.

**Fig. 39:**
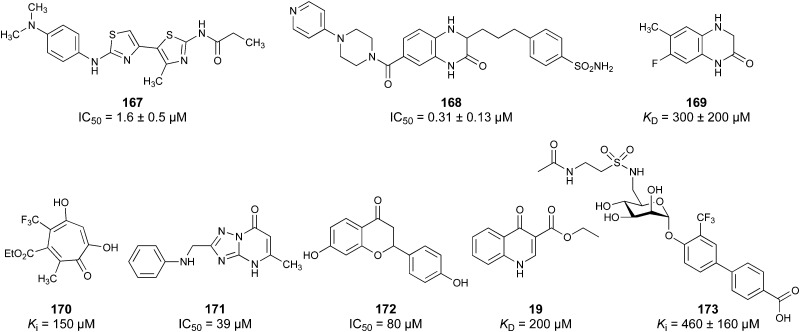
Non-carbohydrate-based glycomimetics for DC-SIGN. For 173, the anomeric biphenyl substituent was identified to bind to a secondary site without significant contributions from the original mannose scaffold.^162^

For DC-SIGN, an orthogonal approach to carbohydrate-based inhibitor design was pioneered by the Kiessling group reporting on a high throughput screening over 30 000 small molecules against DC-SIGN.^587^ This led to the discovery of quinoxalinones and thiazoles (167) as drug-like inhibitors in the low micromolar range (Fig. 39). In a follow-up study by the same group, these ligands were optimized to overcome the liabilities of the previous scaffolds such as the oxidation of the quinoxalinones’ thioether functionality resulting in IC_50_ values of 0.3 μM for 168.^587,588^ As these compounds lacked functional groups to complex Ca^2+^ ions, a carbohydrate binding site-independent mechanism of interaction was suggested.^44,138^

**Fig. 40:**
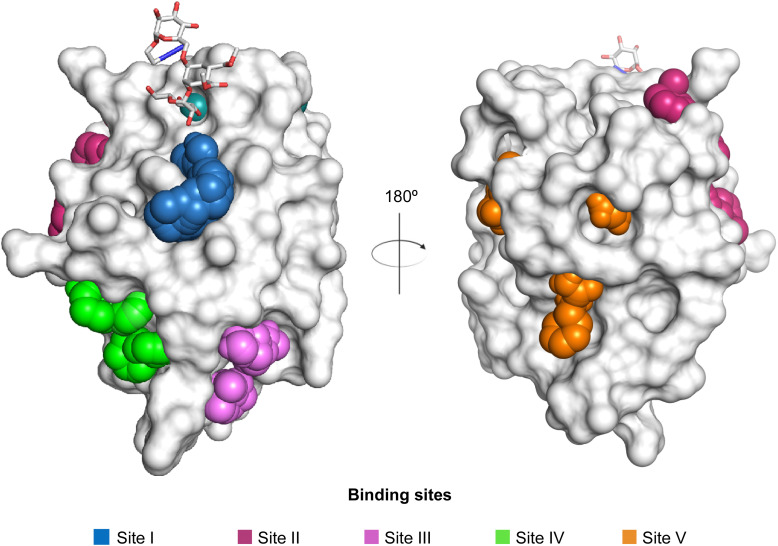
Potential secondary sites available for drug-like molecules to bind to DC-SIGN. The location of the carbohydrate-binding site is indicated by the orthosteric ligand 23 and the calcium ion (shown as green sphere).^160^

More insight into the recognition of non-carbohydrate ligands came from a series of studies describing fragment screening against DC-SIGN.^160,568,589–591^ One report identified several secondary sites from a fragment-based screening using ligand-based NMR, SPR, microarray and an ELLA-type plate-based assay (Fig. 40) and revealed compound 169.^160^ Additionally, a library coming from diversity-oriented synthesis was screened using ^19^F NMR and yielded high micromolar ligands (170) (Fig. 39).^589^ Orthogonal to that, cell-based fragment screening led to the identification of low micromolar inhibitors of DC-SIGN.^590^ Several compounds structurally related to the previously reported quinoxalinones were identified. IC_50_ values of 39 μM and 80 μM were reported for 171 and 172, respectively (Fig. 39).^590^ Overall, these fragments were of considerably lower molecular weights than the HTS hits and revealed several interesting starting scaffolds. These compounds have reasonably high affinities with respect to their overall molecular weight, the so-called ligand efficiency (LE).^124^ Moreover, this led to the discovery of additional binding sites for small molecules, distal to the primary carbohydrate site (Fig. 40).^160^ For a compound of 168 series a secondary site close to the primary site was suggested to inhibit carbohydrate binding by steric repulsion. However, inspired by the quinoxalinone scaffold, a series of quinolones such as 19 were explored as inhibitors for DC-SIGN and close inspection suggested an allosteric inhibition, opening the question for a mode of action for the quinoxalinones again.^591^ Moreover, one of these secondary sites was further supported by insights coming from crystal packaging of carbohydrate-based glycomimetics (24, Fig. 8).^210^ The carbohydrate-based glycomimetic 24 was found to cross-link two DC-SIGN monomers, one with the mannose, the other one with the hydroxymethylphenyl substituent as evidenced by X-ray crystallography. However, cross-linking of a cell surface receptor should not be excluded as a viable mechanism route to inhibition as evidenced by dimerization of the Siglec CD22 or the CTL LOX-1.^592,593^

In a recent report, carbohydrate-based glycomimetic 173 was designed for langerin to benefit from a biphenyl extending of the anomeric position (Fig. 39). 173 showed high affinity for langerin (*K*_D_ = 250 ± 70 μM); however, excellent binding to DC-SIGN expressing cells when 173 was presented on liposomal formulations was reported when analysing off-target recognition.^162^ Closer inspection revealed that this molecule has two functions: the primary Ca^2+^ site is engaged by the mannose scaffold and the aromatic substituent is bound to a previously proposed secondary site (Fig. 40).^160^ Binding of the biphenyl substituent to the secondary site led to the allosteric activation of the primary carbohydrate site. A site around Met270 binds the biphenyl agylcon of an initially envisioned carbohydrate-based glycomimetic and this site as well as the mode of action were supported by several biophysical assays, mutational analysis and molecular dynamics simulation (Fig. 38D).

Taken together, DC-SIGN is amenable to allosteric modulation and first ligands are explored to make use of it, albeit these compounds are still of low affinity and more experiments are necessary to elucidate the pathways allowing coupling of the orthosteric and allosteric sites.

#### 3.3.4 Siglecs

For the Siglecs no functional non-carbohydrate glycomimetics have been reported, likely because no screening of larger libraries of drug-like molecules has been reported. The only exception is the peptide PV3 that binds CD22 and since this binding site is not occupied by *cis*-ligands the threshold for biological activity to target CD22 on the cell surface is lower.^370^ Consequently, a serendipitous discovery of secondary sites is lacking and allosteric modulation of Siglecs is not reported. However, flexible structural elements such as the CC′ loop exist, and their role has only recently been investigated in more depth and molecules fixing their position have the potential to serve as allosteric modulators. For Siglec-7, a second sialic acid binding site was identified, potentially allosterically coupled to the canonical site. Further studies will be necessary to elucidate structural details of the proposed allosteric mechanism and whether the site is druggable or not.^594^

#### 3.3.5 Influenza A virus hemagglutinin

**Fig. 41:**
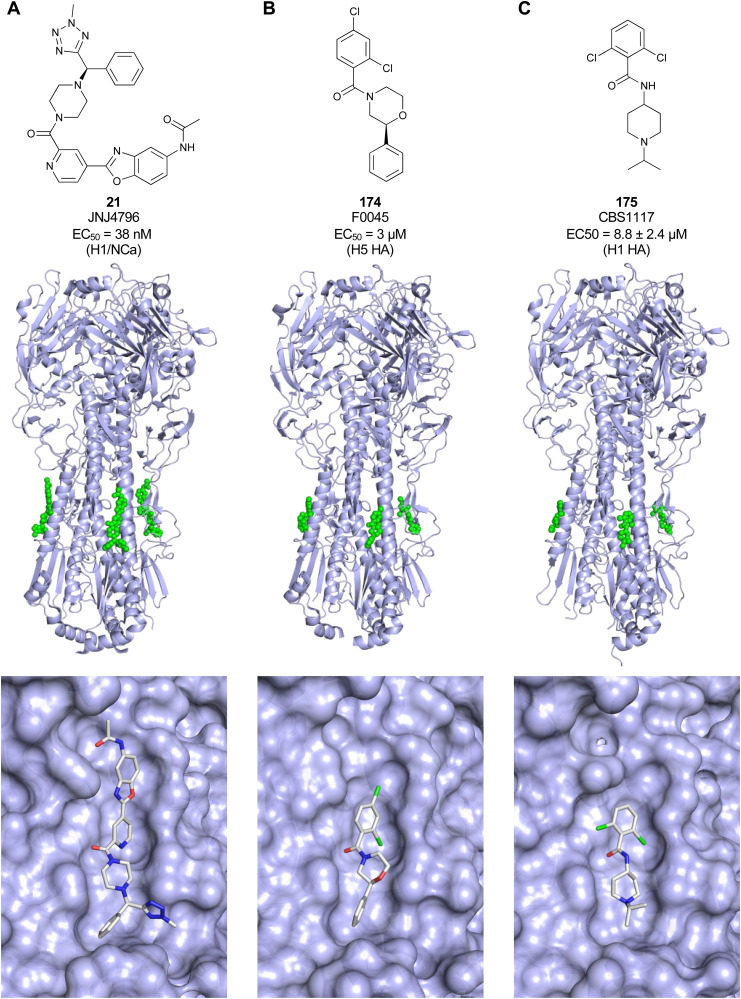
Allosteric inhibitors 21 (A, PDB code 6CFG), 174 (B, PDB code 6WCR) and 175 (C, PDB code 6VMZ) of influenza A virus hemagglutinin are shown (top) with their respective binding sites (bottom). Binding of these molecules in an allosteric site inhibits HA transition to the post-fusion state, thereby preventing endosomal escape of influenza A.

Another carbohydrate-binding protein for which larger conformational rearrangements are known to be essential for its function is hemagglutinin (HA) from influenza A virus. After HA binding to host cell surface sialic acid terminated oligosaccharides, endocytosis is triggered, and the acidification of the endosomal vesicle leads to structural transition of the pre-fusion state of the protein to allow fusion with the endosomal membrane and consequently endosomal escape of the virus. Inhibition of the process by stabilizing the prefusion state has long been envisioned and quite a number of small-molecule inhibitors have been reported.^595–599^ The reader is also referred to recent reviews covering the subject in-depth.^130,600^ Inspired by the success of neutralizing antibodies as well as peptides and protein-based therapeutics targeting a conserved region of the HA stem, several groups identified small-molecule HA ligands fulfilling the same function (21, 174, 175). However, this binding site is challenging compared to the carbohydrate binding site. It is a large and flat site, typically found for protein–protein interaction sites (Fig. 41). Nevertheless, several inhibitors of the pre- to post-fusion state transition have been identified, one (21) with oral bioavailability. Overall, these findings support the notion that allosteric inhibitors of carbohydrate binding proteins are viable options to target these challenging proteins.

Taken together, the development of functional glycomimetics following an allosteric drug design approach remains rarely pursued, yet the number of allosterically modulated lectins accumulates. This is not surprising as also for other drug target classes the use of allostery as a design principle emerged only within the last two decades and was often driven by serendipity.^566,567^ For galectins and CTLs, allosteric sites were identified during a mode of action analysis following a screening of a library of drug-like small molecules or peptides. For carbohydrate-binding proteins, an approach targeting a druggable allosteric site is very attractive compared to focusing on the orthosteric, hydrophilic, flat and solvent-exposed carbohydrate binding site.^44,569,601^ Finally, this opens the question for the biological relevance of such sites in lectins.

## 4. Novel applications of glycomimetics: receptor targeting

### 4.1 The asialoglycoprotein receptor (ASGPR) as target for drug delivery

The asialoglycoprotein receptor (ASGPR), also known as the Ashwell–Morell receptor, was the first mammalian lectin to be discovered.^602^ As a C-type lectin, it requires Ca^2+^ ions for the interaction with carbohydrate ligands. It recognizes galactose and *N*-acetylgalactosamine and clears desialylated glycoproteins with exposed Gal or GalNAc as non-reducing end groups. The mammalian ASGPR is composed of two homologous subunits, major subunit H1 and minor subunit H2, that are encoded by two distinct genes. In humans, the subunits form homo- and hetero-oligomers with different receptor configurations, among which a trimer with two H1 and one H2 is the most abundant.^602–604^ ASGPR is primarily expressed on hepatocytes and minimally on extra-hepatic cells. It belongs to the recycling receptors and is endocytosed and recycled constitutively every *ca.* 15 min, with or without the ligands.^605,606^ Thanks to its unique localization and high expression of up to 500 000 ASGPRs per hepatocyte,^607^ it is an attractive target for receptor-mediated drug delivery to the liver.^21,608,609^ Diseases such as hepatocellular carcinoma,^610^ hepatitis B^611^ and C,^612^ and malaria^613^ are all associated with hepatocytes. Indeed, infection with HBV or HCV may lead to chronic hepatitis, which can cause cirrhosis and liver cancer. Therefore, targeting hepatocytes through the interaction with ASGPR is a viable strategy to treat these diseases.

Two approaches are currently being used: ligand-anchored nanocarriers and drug–ligand conjugates. In addition, RNA interference (RNAi) is becoming increasingly popular to treat a number of liver-related diseases. Four commercially available drugs are currently on the market: givosiran to treat acute hepatic porphyria, lumasiran for severe primary hyperoxaluria type 1, inclisiran for hypercholesterolemia, and vutrisiran for transthyretin-mediated amyloidosis. Targeting these drugs to hepatocytes exploits a trivalent monosaccharide ligand. This application paved the way for least 25 other drug candidates currently in clinical development, and most importantly, it opened the door for novel strategies based on monovalent ligands.

#### Natural ligands

**Fig. 42:**
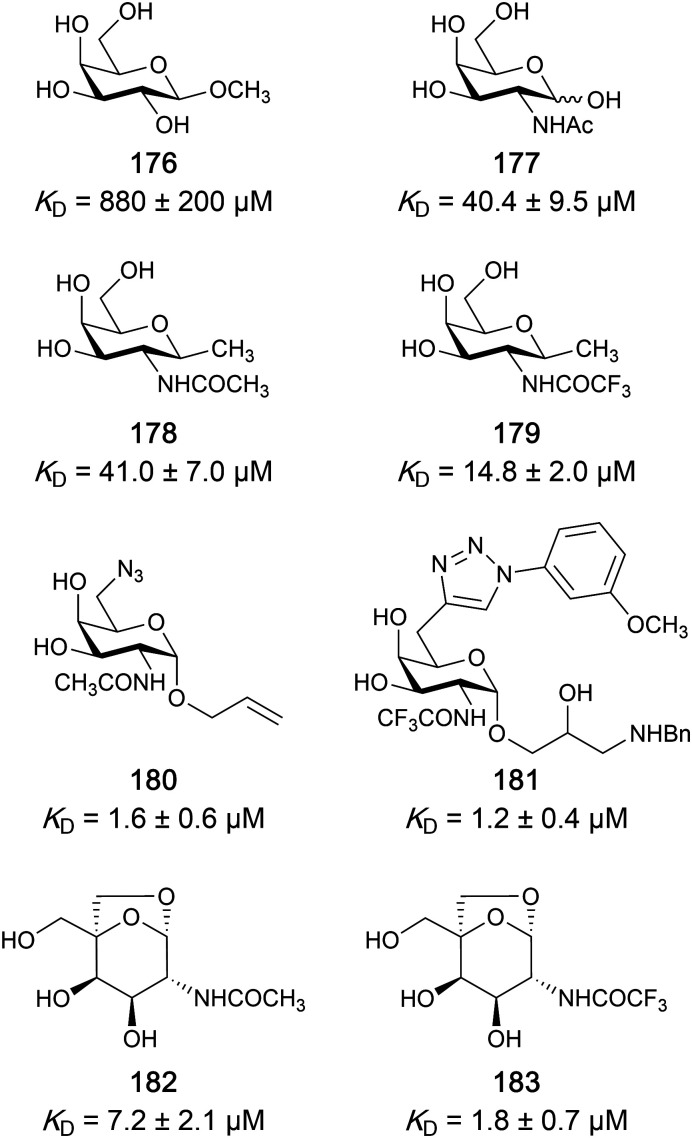
Monovalent ASGPR ligands and their binding affinities.

Natural monosaccharide ASGPR ligands Gal and GalNAc bind with low affinity (MeGal, 176*K*_D_ = 880 ± 200 μM, GalNAc, 177*K*_D_ = 40.4 ± 9.5 μM, Fig. 42).^614^ Binding occurs through hydrogen bonds with the ligand's 3- and 4-OH groups. What makes the ASPGR remarkable is the presence of Trp243 making a CH–π interaction with GalNAc (177) promoting the affinity to 40 μM compared to the millimolar affinities typical for other CTLs (Fig. 4A). ASGPR cannot discriminate between Gal and Glc configurations and can bind both with similar affinity.^615^ In competition assays, ASGPR exhibited 10- to 60-fold higher affinity for GalNAc over Gal.^616–618^

In contrast to monovalent ligands, multivalent ligands show a dramatically enhanced binding affinity for ASGPR. The affinity increases in the order mono- < bi- < triantennary ligand, with IC_50_ values of 1 mM, 1 μM and 1 nM, respectively.^619^ Only a minor improvement was observed when using more than three GalNAc units per ligand.^619,620^ A trivalent ligand is typically built on the molecule of tris(hydroxymethyl)aminomethane (Tris) to which carbohydrate or glycomimetic ligands are attached through amide, ester or ether-based linkers. The geometry of the molecule and the hydrophilic–hydrophobic balance have a great impact on ligand binding. It has been determined that the optimum distance between the three carbohydrate moieties to bind simultaneously to all three CBSs should be between 20 and 30 Å.^621^ The various structural and spatial aspects of multivalent ASGPR ligands have recently been reviewed by Huang *et al.*^622^

The glycoproteins asialofetuin (AF, 12 Gal and 3 GalNAc residues/mol(protein)) and asialoorosomucoid (ASOR, 20 Gal residues/mol(protein)) are endogenous glycoproteins known to bind strongly to ASGPR.^21^ Arabinogalactan and pullulan, a galactose- and glucose-based polymer, respectively, are polysaccharides extensively studied for ASGPR-mediated targeting.^623,624^ Pullulan relies on the ASGPR inability to discriminate between d-Gal and d-Glc.^21^ However, the uptake of pullulan is lower than that of arabinogalactan.^625^

#### Synthetic ASGPR ligands

A large amount of SAR data has been published for ASGPR ligands.^21,614,622^ As mentioned above, binding occurs through the interaction with 3- and 4-OH groups and the remaining positions are available for modification. It was proved that the anomeric configuration does not play any significant role in ligand binding.^626^ A trifluoroacetamido group in position 2 increases the binding affinity 3- to 20-fold compared to the acetamido group (178*vs.*179).^614^ C6 azido- and triazolo-derivatives were also stronger binders than the parent 6-OH compounds (180, 181).^614^ In 2017, Finn and Mascitti (Pfizer)^627,628^ published the bridged ketal structure 182 (substituted 6,8-dioxabicyclo[3.2.1]octane-2,3-diol) which was proposed based on the published crystal structure of the ASGPR binding domain (PDB code 1DV8).^629,630^ Its locked conformation allows for the interaction between the hydrophobic α-face of the pyranose and the tryptophan residue Trp243. The compound showed almost 6-fold better affinity than GalNAc (*K*_D_ = 7.2 *vs.* 40 μM) and an excellent LE of 0.45.^628^ The binding mode was confirmed by crystal structure analysis with ASGPR (PDB code 5JQ1). In line with previous studies, the introduction of the trifluoroacetamido group at C2 (183) improved binding affinity fourfold. However, the non-fluorinated derivative 182 was used for *in vivo* studies because of an uncertain long-term metabolic stability of the trifluoroacetamido group. A trivalent ligand derivative of 182 labelled with the fluorophore AlexaFluor647 showed an impressive affinity with a *K*_D_ of 30 pM. Internalization of the cargo attached to such a triantennary ligand was also confirmed.

#### Drug delivery strategies

Galactosylation of polymers and lipids allows for the design of drug-loaded carriers for hepatocyte-specific targeting. A detailed review on Gal-modified polymers and lipids was published in 2015.^21^ In recent years, polymer–drug conjugates have been studied for the delivery of anticancer drugs to hepatocytes to treat hepatocellular carcinoma (for a detailed review on doxorubicin-based nanotherapeutics, see ref. 631). Polymers with a positive charge, such as poly-l-lysine (PLL) are delivered to hepatocytes thanks to the interaction with the anionic groups of the ASGPR binding site.^632–634^

PK2 (FCE28069) is a copolymer based on *N*-(2-hydroxypropyl)methacrylamide (HPMA) with *N*-linked galactosamine and doxorubicin (DOX). In a preclinical study, the copolymer displayed a 5-fold reduction in cardiotoxicity relative to free DOX.^635^ A Phase I clinical trial study in patients with primary or metastatic liver cancer proved that liver-specific doxorubicin delivery utilizing a galactosamine-modified polymer is achievable.^636,637^ A biodistribution study showed that 24 h after administration, 17% of the administered dose of doxorubicin targeted to the liver while a doxorubicin–polymer conjugate without galactosamine showed no targeting. Although the dosage for Phase II trials was recommended, to the best of our knowledge, there was no further development of PK2.

Targeted nanoparticles take advantage of receptor-mediated endocytosis to deliver a high payload of a drug to the liver. In addition, they protect the drug from degradation and enable the transport of both hydrophobic and hydrophilic drugs. Biodegradability of nanocarriers to non-toxic components is important to preclude toxicity. Hydrophobic nanoparticles are readily cleared off by reticuloendothelial system (RES), and therefore, hydrophilic or stealth particles are preferentially used, with PEG being the most popular stealth agent. While liposomes^638^ are the most widely tested type of nanoparticles, other types are tested, too, *e.g.* micelles,^639,640^ or dendrimers.^641,642^ Another strategy is the direct synthesis of covalent drug–ligand conjugates. This strategy is studied for example for the delivery of radiopharmaceuticals to the liver (for a review, see ref. 21).

#### Delivery of therapeutic nucleic acids to the liver

A hot topic in clinical application of ASGPR-mediated targeting is gene silencing. The aim is to inhibit mRNA translation with short complementary RNA fragments. Antisense oligonucleotides (ASOs), which are single stranded RNA fragments, and small-interfering RNAs (siRNAs), composed of two short RNA fragments assembled in a double strand, are used to apply this concept. While ASOs bind to the complementary RNA directly and target its degradation by RNase, siRNA fragments are loaded into the RNA-induced silencing complex which then targets the mRNA for degradation. Ultimately, both approaches interrupt the production of the corresponding protein by triggering the degradation of the targeted mRNA.^643^ This approach has become very popular in the last decade since virtually any gene can be targeted by RNAi.

#### GalNAc–siRNA conjugates

**Fig. 43:**
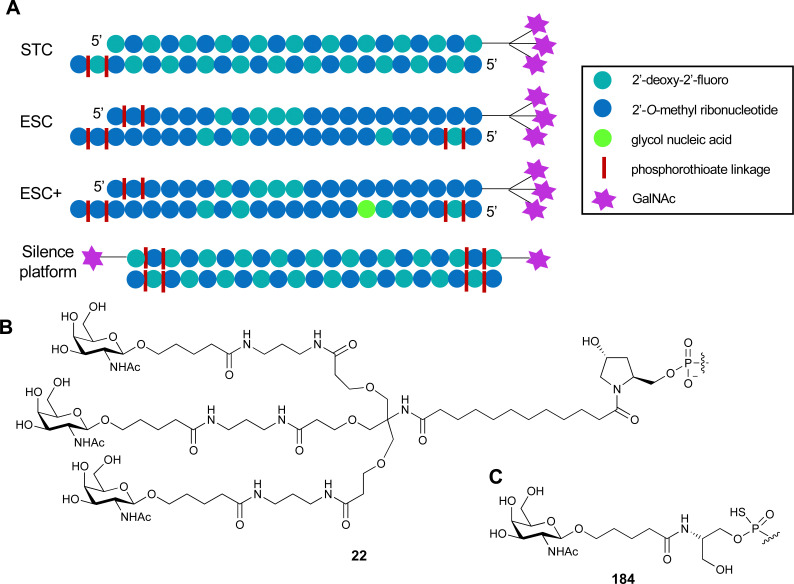
(A) Design of GalNAc–siRNA conjugates: STC-standard template chemistry, ESC-extended stabilization chemistry, ESC+-extended stabilization chemistry plus, Silence platform-single GalNAc positioned at opposite sides of the sense strand. The top strand is the sense strand and bottom the antisense strand. (B) Structure of triantennary GalNAc ligand 22 built on a Tris scaffold. (C) Structure of monovalent GalNAc ligand 184 built on a serinol scaffold.

Two 2014 pilot studies by Nair *et al.* and Prakash *et al.* proved that multivalent GalNAc-conjugated (22) siRNA localizes in hepatocytes and elicits robust RNAi-mediated gene silencing.^644,645^ It is crucial to protect the siRNA with chemical modifications against extra- and intracellular nucleases encountered *en route* from the site of administration (typically subcutaneous) to the cytosol of hepatocytes. Introducing extensive chemical modifications at the 2′ position of the nucleotides (2′-deoxy-2′-fluoro- or 2′-*O*-methyl) and replacing phosphodiester bonds with phosphorothioate (PS) bonds allows for achieving higher potency and longer duration of action.^646,647^ These modifications are referred to as enhanced stabilization chemistry (ESC). Such double stranded RNAs are stable enough to reach the liver *via* the ASGPR after intravenous or subcutaneous injection. The increased potency and durability achieved by the modified siRNA means a lower dose can be used, which decreases the chance of off-target toxicity. Alnylam has introduced another approach to off-target seed interaction – incorporation of a glycol nucleic acid (GNA).^648,649^ This new siRNA design is called ESC+ (Fig. 43).^650^ Arrowhead Pharmaceuticals uses the TRiM (Targeted RNAi Molecule) delivery platform, with GalNAc as hepatocyte targeting moiety that is directly conjugated to siRNA. In a 2020 paper, Weingärtner *et al.* from Silence Therapeutics introduced a novel design based on serinol-attached GalNAc unit(s) (184) either in a series (2–4 units) or two single GalNAc units positioned at opposite ends of the sense strand. This system showed improved stability, *in vivo* activity and duration of action compared with a traditional triantennary design.^651^

**Fig. 44:**
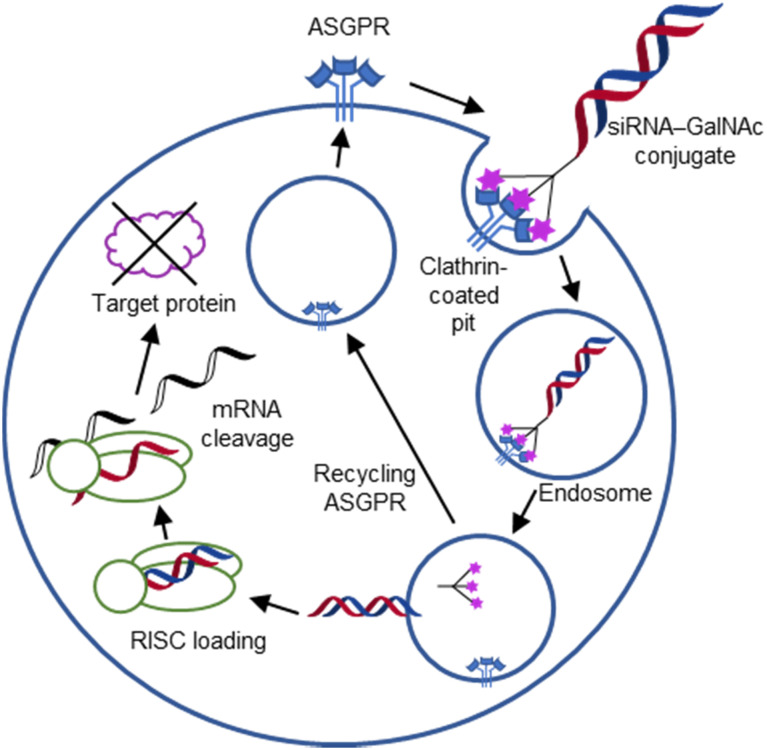
Mechanism of action of siRNA–GalNAc conjugates. After GalNAc-mediated asialoglycoprotein receptor (ASGPR) binding and uptake, the siRNA integrates into an RNA induced silencing complex (RISC) leading to degradation of the respective complementary mRNA. The mechanism for endosomal escape of the siRNA is still unknown.^650^ Reproduced from ref. 653 with permission from The American Society of Gene and Cell Therapy/Elsevier, copyright 2017.

GalNAc–siRNA conjugates bind to the ASGPR and are taken up in endosomes where the conjugate dissociates from the receptor. Additionally, the sugar moieties and branches are very quickly lysed from the oligonucleotide^652^ and then the oligonucleotide translocates to the cytoplasm by endosomal escape (Fig. 44).^650^

As of September 2022, four GalNAc–siRNA drugs are on the market (Table 5), five in the late stage of development (Phase III) and at least 20 in the early stages of development in Phase I or II (sources: clinicaltrials.gov and the pipeline data on the websites of the companies Alnylam, Dicerna, Novo Nordisk, Arrowhead Pharmaceuticals, Silence Therapeutics, and Arbutus Biopharma).^654–658^

**Table 5:** Marketed GalNAc–siRNA-based drugs

No	Name	Company	Indication	Target	Ref.
185	Givosiran (GIVLAARI), ALN-AS1	Alnylam	Acute hepatic porphyria	5-Aminolevulinic acid synthase 1 (ALAS1)	NCT03338816^659–661^
186	Lumasiran (OXLUMO), ALN-GO1	Alnylam	Primary hyperoxaluria type 1 (PH1)	Glycolate oxidase 1 (GO)	NCT03681184^662–665^
187	Inclisiran (LEQVIO), AlN-PCSsc	Alnylam/Novartis	Hypercholesterolemia (heterozygous familial and non-familial) or mixed dyslipidaemia	Proprotein convertase subtilisin–kexin type 9 (PCSK9)	NCT03397121, NCT03399370, NCT03400800^666–668^
188	Vutrisiran (AMVUTTRA), ALN-TTRsc02	Alnylam	Transthyretin-mediated amyloidosis (ATTR)	Transthyretin (TTR)	NCT03759379, NCT04153149^669^

### 4.2 Targeted protein degradation

**Fig. 45:**
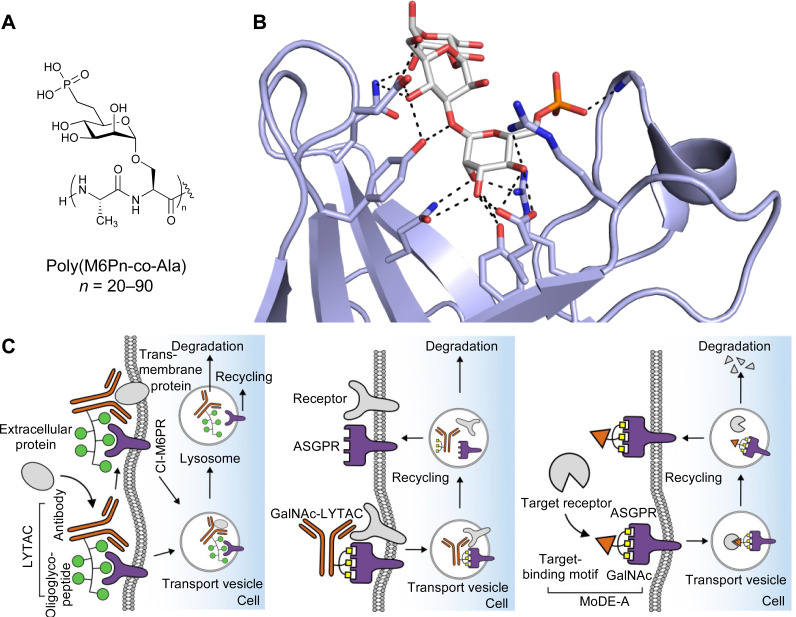
Lectin-mediated protein degradation. (A) Mannose-6-phosphonate peptide polymer used for CI-M6PR targeting.^673^ (B) Pentamannosyl phosphate in complex with bovine CI-M6PR (PDB code 1C39).^684^ (C) Targeted protein degradation through CI-M6PR using LYTAC (left),^673^ and the ASGPR based strategies of GalNAc-LYTAC (middle),^676^ and MoDE-A (right).^680^ While LYTACS rely on antibodies for binding of their extracellular degradation target, MoDE-As utilise small molecules.

Targeted protein degradation (TPD) has opened many avenues for the treatment of various diseases and provided multiple routes to study fundamental biology.^670,671^ Proteolysis-targeting chimeras (PROTACs) are amongst the best understood applications of TPD, now on their way through clinical trials.^672^ Briefly, the approach is based on the application of a heterobifunctional small molecule comprising a ligand for an E3 ligase of the ubiquitin proteasome system and a ligand for the protein of interest, connected by a linker modality. Cross-linking the two proteins then induces covalent tagging of the protein of interest with ubiquitin and consequently leads to its degradation in the proteasome. One major advantage of this technology is the catalytic elimination of the target, compared to blocking the target occupancy. However, this approach is limited to cytosolic proteins while omitting about 40% of the proteome being extracellular or membrane-associated targets. Lysosome-targeting chimeras (LYTACs) make use of lysosome shuttling receptors taking over the E3 ligase function and set the protein of interest *en route* to the lysosome for degradation. It is not surprising to find carbohydrate-binding receptors to be the first to be explored for such an application: the 300 kDa multidomain cation-independent mannose-6-phosphate receptor (CI-M6PR, Fig. 45A and B)^673,674^ and the 100 kDa heterotrivalent C-type lectin ASGPR.^675,676^

The first-generation LYTACs, developed by Bertozzi, targeted the CI-M6PR to exploit receptor-mediated endocytosis of extracellular proteins into the lysosome (Fig. 45C).^673^ The CI-M6PR is a P-type lectin that recognizes mannose-6-phosphate (M6P) residue caps on *N*-glycans of endogenous proteins with a micromolar affinity to transport them from the extracellular space into the lysosome, a system that has been used for treatment of lysosomal storage diseases for a long time.^677,678^ At lower pH in the endosomal compartment prior to the arrival in the lysosome, the cargo is released and CI-M6PR recycles back to the surface resembling an efficient system for cargo uptake. Mimicking the multivalent display of M6P on a glycoprotein cargo for the CI-M6PR, Bertozzi and colleagues used a known multivalent, biocompatible, phosphatase-inert mannose-6-phosphonate (M6Pn) to showcase efficient and constant uptake *via* CI-M6PR in absence of receptor degradation.^673^ These polymers were conjugated to antibodies specific for soluble components of the extracellular space as well as for membrane bound receptors and led to their targeted degradation. The CI-M6PR is amongst a number of potential lysosome trafficking receptors that could potentially be used for the LYTAC approach. Other receptors might provide tissue specificity.

In 2021, the research groups of Bertozzi, Spiegel and Tang independently reported chimeric molecules with triantennary GalNAc for targeted protein degradation in the liver *via* the ASGPR (Fig. 45C).^675,676,679^ This CTL is not ubiquitously expressed as the CI-M6PR and is found primarily on hepatocytes with minimal expression on other cells (see Section 4.1). As a proof of concept, Bertozzi introduced a second-generation LYTAC consisting of a 3.4 kDa peptide binder linked to a trivalent GalNAc ligand that degraded integrins and reduced cancer cell proliferation.^676^ Spiegel, in contrast, focused on small molecule-based lysosome-targeting degraders. He called the heterobifunctional molecules MoDE-As (molecular degraders of extracellular proteins through the asialoglycoprotein receptor, Fig. 45C) and proved the concept by inducing depletion of an antibody and a proinflammatory cytokine.^680^ Tang further confirmed that lysosomal degradation of protein targets through ASGPR is possible by both small molecule- and antibody-based lysosome-targeting degraders. In addition, he showed that molecular size plays an important role and that internalization through ASGPR is more efficient for smaller degrader–target protein complexes.^679^

Avilar Therapeutics focused on extracellular protein degradation and recently introduced the ATAC platform, designed to target diverse pathological proteins and exploit the natural ASGPR protein degradation pathway. ATACs (ASGPR-targeting chimeras) are bifunctional molecules composed of a ligand that binds to ASGPR, linked to a second ligand which binds to a disease-causing extracellular protein. The chemical nature of the ASGPR ligand used in ATACs has not been disclosed, and the company has synthesized hundreds of monosaccharide-based ASGPR ligands, out of which over 100 had a *K*_D_ ≤ 1000 nM and around 40 had a *K*_D_ ≤ 100 nM. In addition, they have over 20 X-ray structures of ASGPR–ligand complexes.^681^ The chosen ASGPR ligand has approximately 2000-fold higher affinity than GalNAc and >60-fold increase in affinity over bicyclic bridged 182. Compared to the previous generation of compounds^682^ which contained trivalent GalNAc ligands attached to an antibody, ATACs are based on bi- and mono-dentate ligands attached to a peptide or small molecule.^683^ For initial proof-of-concept studies, ATACs were designed to target two extracellular proteins with different concentration and kinetic properties: IgG (high plasma concentration and long half-life) and TNF-α (low plasma concentration and short half-life). The *in vitro* studies demonstrated ligand binding, ternary complex formation, cellular uptake, and degradation of the target proteins, IgG and TNF-α.

Taken together, glycomimetics with a proper design to take advantage of specific receptor recognition, but also excellent endosomal release properties to allow receptor recycling and cargo release, will certainly be opening the way for many other lysosome shuttling receptors to be explored.

### 4.3 CD22 targeting – Siglec-engaging tolerance-inducing antigenic liposomes (STALs)

The limited expression of CD22 on B cells and its role as an inhibitory receptor for B cell receptor (BCR) signalling made this Siglec an attractive target for the treatment of various B cell-associated diseases. The carbohydrate-based glycomimetics 6′-MBP-5F-Neu5Ac (15) and 9-BPA-NeuGcα(2-6)Galβ(1-4)GlcNAcβ-spacer (74) developed for human and murine CD22, respectively, were the door openers.^115,685^ As a first application, 9-BPA-NeuGcα(2-6)Galβ(1-4)GlcNAcβ-spacer (74) co-presented with nitrophenol as a model antigen on a polymer backbone was used to enforce colocalization of the BCR and CD22 leading not only to shutdown of BCR signalling and reduced anti-nitrophenol antibody secretion, but also to B cell apoptosis.^685^ In the next step, 9-BPC-NeuAcα(2-6)Galβ(1-4)GlcNAcβ-spacer (75), a ligand still lacking sufficient specificity for human CD22, was successfully applied to kill human B cell lymphoma cells in a mouse; however, still suffering from the unwanted Siglec-1 off-target effect in the animals.^686^ Conceptually, this work was key, since the delivery of doxorubicin encapsulated in liposomes allowed to draw important conclusions about the endocytic properties, alternative particles to overcome *cis*-ligands and the general principle of B cell killing through toxin delivery.

**Fig. 46:**
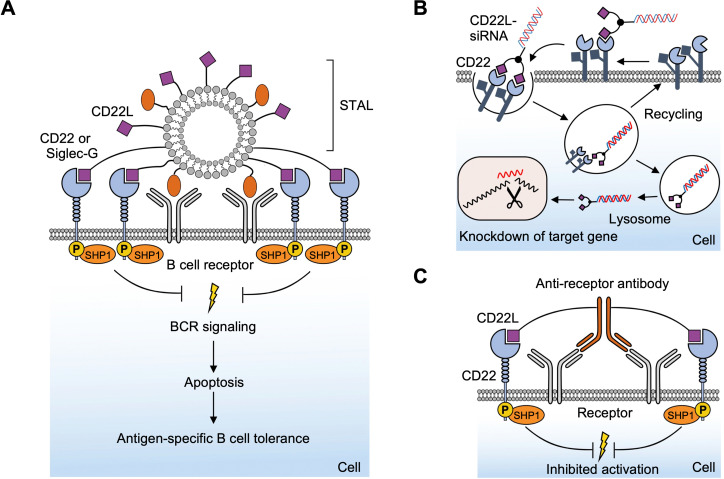
Targeting of the Siglec CD22. (A) STALs reduce antigen specific B cell response by colocalisation of the BCR and the inhibitory CD22. (B) After CD22-mediated uptake of a CD22 ligand–siRNA conjugate the expression of a target protein is suppressed *via* siRNA-mediated gene silencing. (C) Exploiting the same principle as STALs CD22 ligand–antibody conjugates reduce signalling of specific receptors. CD22L = CD22 ligand. Adopted from lit.^367,693,694^

These findings were brought to a new level by implementing Siglec-engaging tolerance-inducing antigenic liposomes (STALs): co-display of an antigen with the 9-BPA-NeuGcα(2-6)Galβ(1-4)GlcNAcβ-spacer (74) ligand for murine CD22 induced B cells apoptosis in mice (Fig. 46A).^367^ Using a haemophilia model in which autoantibody production against factor VII would lead to severe bleeding in mice, Paulson and co-workers showed significantly reduced phenotype by antigen specific B cell ablation.^367^ Similarly efficient, the application of STALs showed great utility of this approach to reduce citrulline-specific B cells *in vivo*. Anti-citrulline autoantibodies are a hallmark of rheumatoid arthritis, an autoimmune disease for which the glycomimetic-based STALs showed applicability.^687^

In a recent follow-up, Macauley and colleagues have shown that changing the carrier from a liposome to red blood cells by direct insertion of the glycomimetic lipid conjugate, similar effects on B cells could be induced. Inhibition of cellular activation, reduction of cytokine secretion and cellular proliferation were achieved.^688^ To even further enhance the peripheral tolerance induction, STALs were co-formulated with rapamycin and using the model antigen ovalbumin it was shown that the anti-OVA antibody production was even more reduced compared to the common STAL formulation.^689^ To expand the application to human disease, the glycomimetic 6′-MBP-5F-Neu5Ac (15) was applied in a human CD22 transgenic mouse in a peanut allergy oral sensitization model.^690^ Together with previous data supporting the coformulation of rapamycin,^689^ the co-administration of STALs with poly(lactic-*co*-glycolic acid)–rapamycin nanoparticles (PLGA-R) induced robust peripheral tolerance compared to PLGA-R particles alone in a model of spontaneous autoimmune arthritis to the self-antigen glucose-6-phosphate-isomerase (GPI).^691^

Overall, CD22 glycomimetics have been successfully applied *in vivo* in various systems to showcase utility and applicability for the delivery of various cargo to B cells, not limited to toxins,^686,692^ model antigens,^685,689^ immunosuppressants,^689,691^ antibodies,^693^ and RNA therapeutics (Fig. 46B and C).^694^ These molecules gave important insights into the role of B cells and the potential treatment of haemophilia,^367^ B cell lymphoma,^686,688^ rheumatoid arthritis,^687^ and allergies,^368,690,691,695^ lately also their application for cellular reengineering to allow natural killer cells to achieve tumour-specific CD22 targeting.^696^

### 4.4 Langerin targeting

**Fig. 47:**
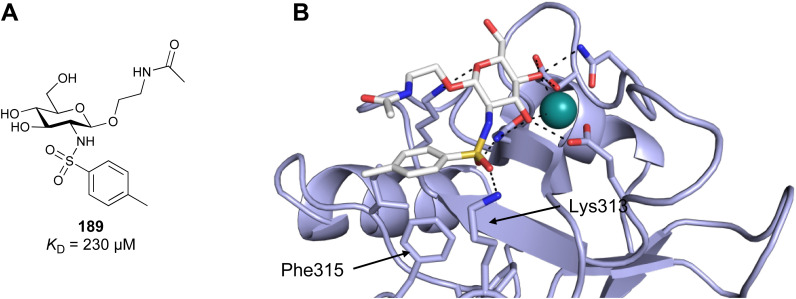
Carbohydrate-based glycomimetic 189 is a ligand for human langerin. (A) Chemical structure of 189.^711^ (B) Computational modelling of the complex between 189 and human langerin was supported by NMR data and shows interaction of Lys313 with the sulfonamide linker and a T-stacking interaction between Phe315 and the tosyl group. Adopted from lit.^711^

Langerin (CD207) is a CTL expressed on Langerhans cells (LCs), the major antigen presenting cells in the epidermis of the human skin. LCs are sentinels in the skin, protecting us against incoming threats,^697^ and central for the induction of an appropriate immune response.^698^ As an innate immune cell receptor, langerin is uniquely expressed by LCs and is involved in pathogen recognition promoting the uptake of several viruses such as HIV,^699^ measles,^700^ and influenza virus^701^ as well as fungi^702^ and mycobacteria.^703^ Langerin is very efficient in pathogen uptake since it is a fast recycling receptor, similar to the ASGPR and hence holds promise to be of similar utility for cell-specific delivery of therapeutics.^152,704^ The utility of langerin-based delivery has been explored previously using antibodies and has revealed that langerin mediated cargo uptake leads to cross-presentation of exogenous antigens, important for anti-viral and anti-cancer therapeutics.^705–710^ In this respect, the recent development of the carbohydrate-based glycomimetic 189 for human langerin was reported.^711^ The *N*-tosylated glucosamine 189 makes use of the Ca^2+^-mediated canonical hydroxy group coordination of the 3- and 4-OH groups. It was suggested that the tosyl group is involved in a T-stack interaction with Phe315 in the langerin binding site, resulting in an overall affinity of 230 μM including additional effects coming from the linker at the anomeric position (Fig. 47).^711^ Overall, these interactions add up to an overall 100-fold affinity gain over the parent glucose (*K*_i_ = 21 ± 4 mM). Remarkable affinity gains of the *N*-tosylated glucosamine could already be achieved by bivalent display on a DNA/PNA backbone with optimized ligand spacing^712^ and multivalent display on liposomes or proteins leading to highly specific delivery to LCs.^711,713,714^ When a protein antigen was directly conjugated to multiple glycomimetic ligands, LC specific delivery could also be shown *ex vivo* in intact human skin samples, unlocking new potentials for therapeutical exploring LC targeting and modulating the immune system.^715^

### 4.5 Selectin-targeting

Following the success of glycomimetics in various indications, they have consequently also found use as delivery agents. Besides the clinically used ASGPR ligands, the use of carbohydrates as targeting ligands has been widely studied in numerous applications using nanoparticles, *e.g.* for the imaging of inflammation in the brain *via* sialyl LewisX-decorated MRI active nanoparticles^716^ or galactosides as mono- or oligosaccharides for the targeting of galectins.^717^ Using the above-mentioned advantages of glycomimetics over natural glycosides, this concept has also been implemented for the targeting of the selectins.

Sialyl LewisX glycomimetics have been developed for the production of E-selectin targeting liposomes.^718^ To this end, 33 (Fig. 6) bearing an *N*-acetyl group in position 2 of the galactose residue was modified at its carboxylic acid and coupled to the lipid 1,2-distearoyl-*sn-glycero*-3-phosphoethanolamine (DSPE). Then, liposomes have been produced and were analysed for their E-selectin binding and targeting efficacy *in vitro* and *in vivo*. Targeted liposomes were shown to bind to E-selectin in an SPR experiment while untargeted liposomes were devoid of binding. Similar results were observed when fluorescent liposomes were assessed by FACS for binding to TNF-α-activated HUVEC cells where specificity was obtained upon E-selectin expression, further demonstrating the targeting efficacy in a cellular context. Last, the imaging of subcutaneously implanted non small cell lung carcinoma tumours in mice was successfully achieved with those glycomimetic liposomes, suggesting future applications for the delivery of loaded drug cargo.

### 4.6 Delivery of antibiotics *via* lectin targeting for *P. aeruginosa*

**Fig. 48:**
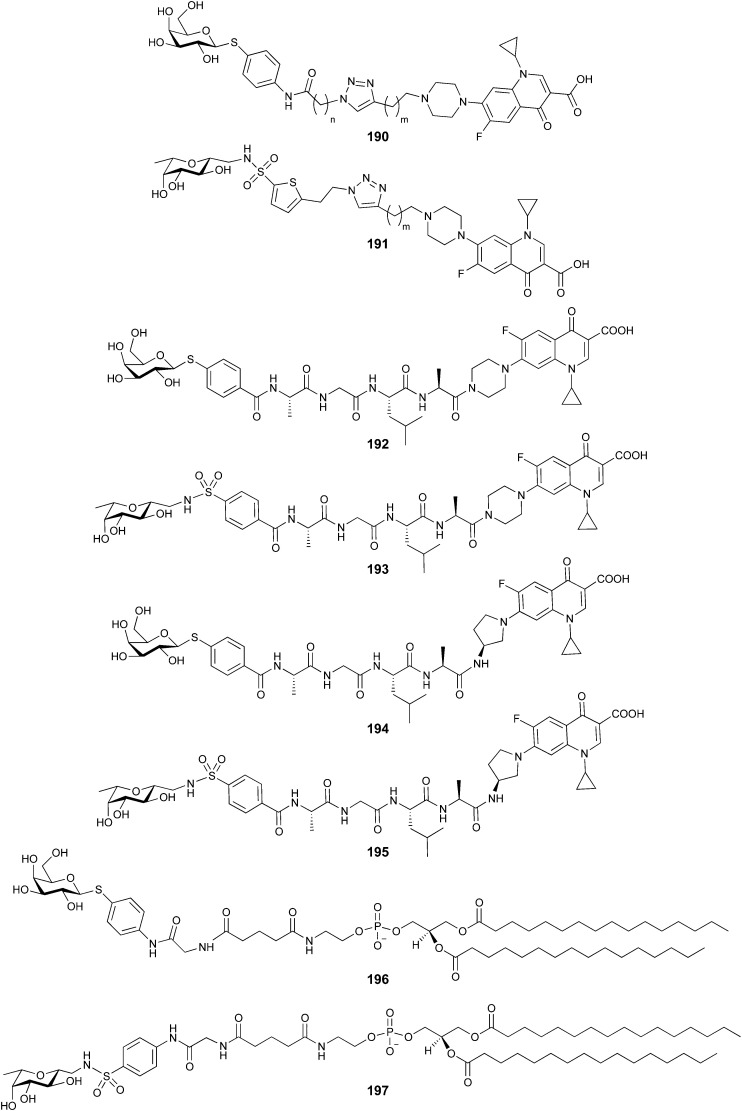
Targeting of *P. aeruginosa via* its lectins LecA and LecB. Glycomimetics 190 and 191 are uncleavable antibiotic conjugates, while 192–195 are their cleavable successors designed for *P. aeruginosa* triggered proteolytic release of the antibiotic. Liposomes containing targeting lipids 196 and 197 can be used for the delivery of diverse cargo towards *P. aeruginosa*.

Bacterial infections are increasingly difficult to treat due rising antimicrobial resistance of the infectious agents and their additional defense within biofilms, *e.g.* for *P. aeruginosa*.^439^ Simply increasing the antibiotic concentration to break resistance and achieve effective antibacterial affinity is, however, not a tractable approach in the patient due to the toxicity of the drugs. Thus, one way to overcome this drawback is to conjugate antibiotics to targeting moieties for the selective enrichment at the pathogen inside the patient, either *via* siderophore-mediated uptake into the bacteria^719,720^ or through binding to their surface *via* antibodies^721^ and thereby increase the drug's local concentration for efficient killing. As a proof-of-concept, it was demonstrated that the LecA-targeted glycomimetic 116 conjugated to a fluorescent probe can bind and image *P. aeruginosa* biofilms.^473^ To implement the targeting, glycomimetics developed for the inhibition of *P. aeruginosa* lectins have been exploited as targeting units in three approaches (Fig. 48).

First, the conjugation of LecA- and LecB-targeting moieties to the clinically used antibiotic ciprofloxacin, that suffers from rare but severe side effects, was reported.^722^ In this work, the targeting units were covalently attached to ciprofloxacin through a triazole ring and varying linker lengths resulting in conjugates 190 and 191. While the lectin binding of these conjugates was effective in the low μM range, the antimicrobial efficacy of the conjugates was reduced probably due to a reduced uptake to reach their intrabacterial targets, topoisomerase and gyrase. However, an enrichment of the conjugates at the *P. aeruginosa* biofilm and a reduced cytotoxicity of the conjugates compared to the free drug was achieved.

In a follow-up report, the linker was varied and the tetrapeptide sequence Gly-Ala-Leu-Ala was implemented as a scission motif for pathogen-specific activation by the secreted bacterial protease LasB to liberate the free drug at the infection site.^723^ The resulting conjugates 192–195 have been analysed for their degradation kinetics in various matrices and their antimicrobial activity. While all conjugates were stable in human blood plasma *in vitro*, they were rapidly cleaved in the presence of a *P. aeruginosa* culture supernatant containing the activating enzyme LasB. The degradation products from initial LasB scission were the lectin targeting moiety, available for blocking the respective lectin to weaken the biofilm, and a dipeptidyl antibiotic, which was further processed by plasma peptidases to liberate the free drug. Interestingly, the ciprofloxacin conjugates 192 and 193 were not fully cleaved into the free drug as a result of the tertiary amide at its piperazine moiety, resulting in reduced antimicrobial activity of the cleavage product. To overcome this drawback, fluoroquinolone derivatives with primary amides were used instead and a full proteolytic processing of conjugates 194 and 195 was observed and active drugs were liberated.

In a third approach by Titz and co-workers,^724^ the same LecA- and LecB-targeting ligands were attached to diacyl glycerol lipids (196 and 197) for incorporation into liposomes aiming at the delivery of unmodified antibiotics loaded into the nanoparticles. In an artificial biofilm model where the lectins LecA or LecB have been immobilized on an abiotic surface, it was shown that the targeted liposomes are specifically retained under flow conditions in a carbohydrate-dependent manner. Importantly, the binding was very tight as a result of the liposomes' multivalency which was demonstrated by the facts that for the LecA-targeted liposomes, 100 mM galactose in the buffer was required for displacement from the LecA surface, while for the LecB-targeted liposomes, even 250 mM fucose only partially displaced the glycomimetic liposomes and required the addition of EDTA to remove the calcium ions from LecB for full dissociation of the liposome from the lectin surface. Furthermore, it was demonstrated by SPR that the LecA-targeted liposome binding affinity is so strong due to the multivalency within the liposome that the dissociation of the liposomes from immobilised lectin is virtually absent. Thus, these glycomimetic liposomes constitute a promising delivery device for the targeting of antibiotics, which requires further research.

## 5. Conclusions and outlook

The more we learn about the biology of lectins, the more this exciting target class will move into the focus of drug discovery. As of now, lectins have slowly emerged as targets for pharmaceutical treatment of a number of diseases, either validated by the use of therapeutic antibodies or the persistent work of the community to develop small-molecule mimetics of their natural carbohydrate substrates.

Carbohydrates as natural ligands themselves have been challenging to promote into a pharmaceutical active ingredient. However, they are stereochemically rich starting structures with good ligand efficiencies, relating the low molecular weight of a monosaccharide and the affinity resulting from the high number of directed interactions. These often enthalpy-driven interactions provide specificity with respect to the many off-targets other than lectins themselves. Glycomimetics are not reported to have significant unwanted targets other than lectins. Still, within the lectins as a target group, specificity can be generated through the monosaccharide, by modification of the original scaffold only. Advancements in carbohydrate chemistry have made this compound class more accessible while still being challenging compared to other scaffolds. Additionally, while a growing number of glycomimetics has been developed into orally available drug candidates, several applications of glycomimetics are independent of oral application routes, similar to the treatment with therapeutic antibodies.

To overcome the limitations of maintaining the original carbohydrate scaffold, alternative approaches have emerged by replacing the sugar core by keeping only the most relevant pharmacophores to establish affinity and specificity leading to non-carbohydrate based, functional glycomimetics. Another approach that might lead to a modulation of lectins for therapeutic intervention are allosteric modulators as functional glycomimetics. These molecules might serve as antagonists or even agonists, *via* binding to allosteric sites, remote from the carbohydrate recognition site, rendering the necessity to resemble a carbohydrate obsolete. Our enthusiasm over this promising route to tackle lectins is fuelled by the advancement it brought to other challenging drug target classes such as kinases and phosphatases.

Infection processes are inherently linked to carbohydrate–lectin interactions. This fact therefore provides a valuable starting point for the development of new antiinfectives. To this end, the rich field of glycomimetics in the discovery phase has already led to numerous compounds in (pre-)clinical development, especially for bacterial infections by *E. coli* in urinary tract infections and inflammatory bowel disease.

Besides the intriguing biological functions of lectins in inflammation, cancer and infection, mammalian lectins are typically limited in their expression profile for defined cells, which makes them attractive targets for targeted delivery purposes. The ASGPR as an accessible hepatic receptor has spearheaded this development. The trivalent GalNAc ligand 22 has assisted to bring four drugs onto the market and led to numerous new therapeutics in clinical trials. Similarly, the CI-M6PR has been proposed as target protein for the development of LYTACs, based on simple mannose-6-phosphonate polymers that allow targeted degradation of extracellular proteins. For the future clinical application of this concept it will be helpful to identify more tissue specific, endocytic receptors with high expression profiles. These will be key towards the specific degradation of targeted membrane receptors.

Taken together, we anticipate a prosperous future for glycomimetics to modulate biological processes in chemical biology research but also as drugs in a large diversity of indications.

## Conflicts of interest

C. R. is co-founder, advisor, and shareholder of Cutanos GmbH, a company that is working on the commercialization of a glycomimetic-based targeting of Langerhans cells. C. R. and A. T. are inventors on patents covering glycomimetics.

## Supplementary Material

## References

[cit1] Varki A. , Cummings R. D., Esko J. D., Stanley P., Hart G. W., Aebi M., Mohnen D., Kinoshita T., Packer N. H., Prestegard J. H., Schnaar R. L. and Seeberger P. H., Essentials of Glycobiology, Cold Spring Harbor Laboratory Press, Cold Spring Harbor (NY), 4th edn, 2022 35536922

[cit2] Laine R. A. (1994). Glycobiology.

[cit3] Martínez-Palomo A. (1970). Int. Rev. Cytol..

[cit4] Costerton J. W., Irvin R. T., Cheng K.-J. (1981). Annu. Rev. Microbiol..

[cit5] Haynes P. A. (1998). Glycobiology.

[cit6] Crine S. L., Acharya K. R. (2022). FEBS J..

[cit7] Ferguson M. A. J., Williams A. F. (1988). Annu. Rev. Biochem..

[cit8] Ikezawa H. (2002). Biol. Pharm. Bull..

[cit9] Ge F., Zhu L., Aang A., Song P., Li W., Tao Y., Du G. (2018). Biotechnol. Lett..

[cit10] Harvey B. M. and Haltiwanger R. S., in Molecular Mechanisms of Notch Signaling, ed. T. Borggrefe and B. D. Giaimo, Springer International Publishing, Cham, 2018, pp. 59–78 10.1007/978-3-319-89512-3_4

[cit11] Reily C., Stewart T. J., Renfrow M. B., Novak J. (2019). Nat. Rev. Nephrol..

[cit12] Buffone, Jr. A., Weaver V. M. (2019). J. Cell Biol..

[cit13] Offeddu G. S., Hajal C., Foley C. R., Wan Z., Ibrahim L., Coughlin M. F., Kamm R. D. (2021). Commun. Biol..

[cit14] Lumibao J. C., Tremblay J. R., Hsu J., Engle D. D. (2022). J. Exp. Med..

[cit15] Radhakrishnan A. , Park K., Kwak I., Jaabir M. and Sivakamavalli J., in Lectins: Innate immune defense and Therapeutics, ed. P. Elumalai and S. Lakshmi, Springer Singapore, Singapore, 2021, pp. 51–72 10.1007/978-981-16-7462-4_3

[cit16] Engering A., Geijtenbeek T. B., van Vliet S. J., Wijers M., van Liempt E., Demaurex N., Lanzavecchia A., Fransen J., Figdor C. G., Piguet V., van Kooyk Y. (2002). J. Immunol..

[cit17] den Dunnen J., Gringhuis S. I., Geijtenbeek T. B. (2009). Cancer Immunol. Immunother..

[cit18] Romani N., Clausen B. E., Stoitzner P. (2010). Immunol. Rev..

[cit19] McEver R. P. (2015). Cardiovasc. Res..

[cit20] Krause T., Turner G. A. (1999). Clin. Exp. Metastas..

[cit21] D'Souza A. A., Devarajan P. V. (2015). J. Controlled Release.

[cit22] Yang R.-Y., Rabinovich G. A., Liu F.-T. (2008). Expert Rev. Mol. Med..

[cit23] Geijtenbeek T. B., Kwon D. S., Torensma R., van Vliet S. J., van Duijnhoven G. C. F., Middel J., Cornelissen I. L. H. A., Nottet H. S. L. M., KewalRamani V. N., Littman D. R., Figdor C. G., van Kooyk Y. (2000). Cell.

[cit24] Lozach P.-Y., Lortat-Jacob H., de Lacroix de Lavalette A., Staropoli I., Foung S., Amara A., Houles C., Fieschi F., Schwartz O., Virelizier J. L., Arenzana-Seisdedos F., Altmeyer R. (2003). J. Biol. Chem..

[cit25] Amraei R., Yin W., Napoleon M. A., Suder E. L., Berrigan J., Zhao Q., Olejnik J., Chandler K. B., Xia C., Feldman J., Hauser B. M., Caradonna T. M., Schmidt A. G., Gummuluru S., Muhlberger E., Chitalia V., Costello C. E., Rahimi N. (2021). ACS Cent. Sci..

[cit26] Alvarez C. P., Lasala F., Carrillo J., Muniz O., Corbi A. L., Delgado R. (2002). J. Virol..

[cit27] Lewis A. L. , Kohler J. L. and Aebi M., in Essentials of Glycobiology, ed. A. Varki, R. D. Cummings, J. D. Esko, P. Stanley, G. W. Hart, M. Aebi, A. G. Darvill, T. Kinoshita, N. H. Packer, J. H. Prestegard, R. L. Schnaar and P. H. Seeberger, Cold Spring Harbor Laboratory Press, 2022, ch. 37 10.1101/glycobiology.4e.37 27010055

[cit28] Flemming H.-C., Wingender J. (2010). Nat. Rev. Microbiol..

[cit29] Sharon N. (2006). Biochim. Biophys. Acta, Gen. Subj..

[cit30] Smith D. C., Lord J. M., Roberts L. M., Johannes L. (2004). Semin. Cell Dev. Biol..

[cit31] Pellizzari R., Rossetto O., Schiavo G., Montecucco C. (1999). Philos. Trans. R. Soc., B.

[cit32] Lord J. M., Roberts L. M., Robertus J. D. (1994). FASEB J..

[cit33] Kitov P. I., Sadowska J. M., Mulvey G., Armstrong G. D., Ling H., Pannu N. S., Read R. J., Bundle D. R. (2000). Nature.

[cit34] Neri P., Nagano S. I., Yokoyama S.-I., Dohi H., Kobayashi K., Miura T., Inazu T., Sugiyama T., Nishida Y., Mori H. (2007). Microbiol. Immunol..

[cit35] Kulkarni A. A., Fuller C., Korman H., Weiss A. A., Iyer S. S. (2010). Bioconjugate Chem..

[cit36] Yosief H. O., Iyer S. S., Weiss A. A. (2013). Infect. Immun..

[cit37] Sager C. P., Eriş D., Smieško M., Hevey R., Ernst B. (2017). Beilstein J. Org. Chem..

[cit38] Mullin N. P., Hitchen P. G., Taylor M. E. (1997). J. Biol. Chem..

[cit39] Schwarz F. P., Puri K. D., Bhat R. G., Surolia A. (1993). J. Biol. Chem..

[cit40] Cabani S., Gianni P., Mollica V., Lepori L. (1981). J. Solution Chem..

[cit41] Steiner T. (2002). Angew. Chem., Int. Ed..

[cit42] Jeffrey G. A. , An Introduction to Hydrogen Bonding, Oxford University Press, 1997

[cit43] Hudson K. L., Bartlett G. J., Diehl R. C., Agirre J., Gallagher T., Kiessling L. L., Woolfson D. N. (2015). J. Am. Chem. Soc..

[cit44] Ernst B., Magnani J. L. (2009). Nat. Rev. Drug. Discov..

[cit45] Copeland R. A. (2021). Expert Opin. Drug Dis..

[cit46] Georgi V., Schiele F., Berger B.-T., Steffen A., Marin Zapata P. A., Briem H., Menz S., Preusse C., Vasta J. D., Robers M. B., Brands M., Knapp S., Fernández-Montalván A. (2018). J. Am. Chem. Soc..

[cit47] Lipinski C. A. (2000). J. Pharmacol. Toxicol..

[cit48] Overeem N. J., Hamming P. H., Tieke M., van der Vries E., Huskens J. (2021). ACS Nano.

[cit49] Dubacheva G. V., Curk T., Mognetti B. M., Auzély-Velty R., Frenkel D., Richter R. P. (2014). J. Am. Chem. Soc..

[cit50] Dubacheva G. V., Curk T., Frenkel D., Richter R. P. (2019). J. Am. Chem. Soc..

[cit51] Fasting C., Schalley C. A., Weber M., Seitz O., Hecht S., Koksch B., Dernedde J., Graf C., Knapp E.-W., Haag R. (2012). Angew. Chem., Int. Ed..

[cit52] Holla A., Skerra A. (2011). Protein Eng., Des. Sel..

[cit53] Curtis B. M., Scharnowske S., Watson A. J. (1992). Proc. Natl. Acad. Sci. U. S. A..

[cit54] Taylor M. E., Drickamer K. (2014). Curr. Opin. Struc. Biol..

[cit55] Cao X., Du X., Jiao H., An Q., Chen R., Fang P., Wang J., Yu B. (2022). Acta Pharm. Sin. B.

[cit56] Patocka J., Nepovimova E., Wu W., Kuca K. (2020). Environ. Toxicol. Pharmacol..

[cit57] Schwarz C., Taccetti G., Burgel P.-R., Mulrennan S. (2022). Resp. Med..

[cit58] Heinemann V., Hertel L. W., Grindey G. B., Plunkett W. (1988). Cancer Res..

[cit59] Siegel D., Hui H. C., Doerffler E., Clarke M. O., Chun K., Zhang L., Neville S., Carra E., Lew W., Ross B., Wang Q., Wolfe L., Jordan R., Soloveva V., Knox J., Perry J., Perron M., Stray K. M., Barauskas O., Feng J. Y., Xu Y., Lee G., Rheingold A. L., Ray A. S., Bannister R., Strickley R., Swaminathan S., Lee W. A., Bavari S., Cihlar T., Lo M. K., Warren T. K., Mackman R. L. (2017). J. Med. Chem..

[cit60] Som P., Atkins H. L., Bandoypadhyay D., Fowler J. S., MacGregor R. R., Matsui K., Oster Z. H., Sacker D. F., Shiue C. Y., Turner H., Wan C. N., Wolf A. P., Zabinski S. V. (1980). J. Nucl. Med..

[cit61] Hillebrand I., Boehme K., Frank G., Fink H., Berchtold P. (1979). Res. Exp. Med..

[cit62] Woods J. M., Bethell R. C., Coates J. A., Healy N., Hiscox S. A., Pearson B. A., Ryan D. M., Ticehurst J., Tilling J., Walcott S. M. (1993). et al.. Antimicrob. Agents Chemother..

[cit63] Cheng J. W. M. (2002). Clin. Ther..

[cit64] Kim C. U., Lew W., Williams M. A., Liu H., Zhang L., Swaminathan S., Bischofberger N., Chen M. S., Mendel D. B., Tai C. Y., Laver W. G., Stevens R. C. (1997). J. Am. Chem. Soc..

[cit65] Maryanoff B. E., Nortey S. O., Gardocki J. F., Shank R. P., Dodgson S. P. (1987). J. Med. Chem..

[cit66] Lembcke B., Fölsch U. R., Creutzfeldt W. (1985). Digestion.

[cit67] Meng W., Ellsworth B. A., Nirschl A. A., McCann P. J., Patel M., Girotra R. N., Wu G., Sher P. M., Morrison E. P., Biller S. A., Zahler R., Deshpande P. P., Pullockaran A., Hagan D. L., Morgan N., Taylor J. R., Obermeier M. T., Humphreys W. G., Khanna A., Discenza L., Robertson J. G., Wang A., Han S., Wetterau J. R., Janovitz E. B., Flint O. P., Whaley J. M., Washburn W. N. (2008). J. Med. Chem..

[cit68] Kolb H. C., Ernst B. (1997). Chem. – Eur. J..

[cit69] Thoma G., Magnani J. L., Patton J. T., Ernst B., Jahnke W. (2001). Angew. Chem., Int. Ed..

[cit70] Tamburrini A., Colombo C., Bernardi A. (2020). Med. Res. Rew..

[cit71] Sattin S. and Bernardi A., Carbohydrate Chemistry, The Royal Society of Chemistry, 2016, vol. 41, pp. 1–25

[cit72] Damalanka V. C., Maddirala A. R., Janetka J. W. (2021). Expert Opin. Drug Disc..

[cit73] Hevey R. (2019). Pharmaceuticals.

[cit74] Hevey R. (2021). Chem. – Eur. J..

[cit75] Cecioni S., Imberty A., Vidal S. (2015). Chem. Rev..

[cit76] Colombo C., Bernardi A. (2011). Eur. J. Org. Chem..

[cit77] Wang W., Rattananakin P., Goekjian P. G. (2003). J. Carbohydr. Chem..

[cit78] Driller K. M., Libnow S., Hein M., Harms M., Wende K., Lalk M., Michalik D., Reinke H., Langer P. (2008). Org. Biomol. Chem..

[cit79] Cumpstey I., Agrawal S., Borbas K. E., Martín-Matute B. (2011). Chem. Commun..

[cit80] Yang Y., Yu B. (2017). Chem. Rev..

[cit81] Bijian K., Zhang Z., Xu B., Jie S., Chen B., Wan S., Wu J., Jiang T., Alaoui-Jamali M. A. (2012). Eur. J. Med. Chem..

[cit82] Sidoryk K., Rárová L., Oklešťková J., Pakulski Z., Strnad M., Cmoch P., Luboradzki R. (2016). Org. Biomol. Chem..

[cit83] McDonagh A. W., Mahon M. F., Murphy P. V. (2016). Org. Lett..

[cit84] Suzuki T., Makyio H., Ando H., Komura N., Menjo M., Yamada Y., Imamura A., Ishida H., Wakatsuki S., Kato R. (2014). Bioorg. Med. Chem..

[cit85] Pérez-Victoria I., Boutureira O., Claridge T. D., Davis B. G. (2015). Chem. Commun..

[cit86] André S., Kövér K. E., Gabius H.-J., Szilágyi L. (2015). Bioorg. Med. Chem. Lett..

[cit87] Sommer R., Makshakova O. N., Wohlschlager T., Hutin S., Marsh M., Titz A., Künzler M., Varrot A. (2018). Structure.

[cit88] Kostlánová N., Mitchell E. P., Lortat-Jacob H., Oscarson S., Lahmann M., Gilboa-Garber N., Chambat G., Wimmerová M., Imberty A. (2005). J. Biol. Chem..

[cit89] Demchenko A. V. , Handbook of chemical glycosylation: advances in stereoselectivity and therapeutic relevance, John Wiley & Sons, 2008

[cit90] Spell M., Wang X., Wahba A. E., Conner E., Ragains J. (2013). Carbohydr. Res..

[cit91] Furuta T., Takeuchi K., Iwamura M. (1996). Chem. Commun..

[cit92] Lian G., Zhang X., Yu B. (2015). Carbohydr. Res..

[cit93] Lahiri R., Ansari A. A., Vankar Y. D. (2013). Chem. Soc. Rev..

[cit94] Stütz A. E., Wrodnigg T. M. (2011). Adv. Carbohydr. Chem. Biochem..

[cit95] Gu X., Gupta V., Yang Y., Zhu J. Y., Carlson E. J., Kingsley C., Tash J. S., Schönbrunn E., Hawkinson J., Georg G. I. (2017). ChemMedChem.

[cit96] Paulsen H. (1966). Angew. Chem., Int. Ed. Engl..

[cit97] Dwek R. A., Butters T. D., Platt F. M., Zitzmann N. (2002). Nat. Rev. Drug Discov..

[cit98] Arjona O., Gomez A. M., Lopez J. C., Plumet J. (2007). Chem. Rev..

[cit99] Roscales S., Plumet J. (2016). Int. J. Carbohydr. Chem..

[cit100] López-Méndez B., Jia C., Zhang Y., Zhang L. H., Sinay P., Jiménez-Barbero J., Sollogoub M. (2008). Chem. – Asian J..

[cit101] Robina I., Vogel P., Witczak Z. J. (2001). Curr. Org. Chem..

[cit102] Witczak Z. J. (1999). Curr. Med. Chem..

[cit103] Liao X., Větvička V. c, Crich D. (2018). J. Org. Chem..

[cit104] Ito S., Yamashita M., Niimi T., Fujie M., Reddy V. K., Totsuka H., Haritha B., Maddali K., Nakamura S., Asai K. (2009). Heterocycl. Commun..

[cit105] Dayde B., Pierra C., Gosselin G., Surleraux D., Ilagouma A. T., Laborde C., Volle J. N., Virieux D., Pirat J. L. (2014). Eur. J. Org. Chem..

[cit106] Xu B., Unione L., Sardinha J., Wu S., Ethève-Quelquejeu M., Pilar Rauter A., Blériot Y., Zhang Y., Martín-Santamaría S., Díaz D. (2014). Angew. Chem., Int. Ed..

[cit107] Linclau B., Golten S., Light M., Sebban M., Oulyadi H. (2011). Carbohydr. Res..

[cit108] Hevey R. (2019). Biomimetics.

[cit109] Biffinger J. C., Kim H. W., DiMagno S. G. (2004). ChemBioChem.

[cit110] Withers S. G., MacLennan D. J., Street I. P. (1986). Carbohydr. Res..

[cit111] Wohlschlager T., Butschi A., Grassi P., Sutov G., Gauss R., Hauck D., Schmieder S. S., Knobel M., Titz A., Dell A., Haslam S. M., Hengartner M. O., Aebi M., Künzler M. (2014). Proc. Natl. Acad. Sci. U. S. A..

[cit112] Han Z., Pinkner J. S., Ford B., Obermann R., Nolan W., Wildman S. A., Hobbs D., Ellenberger T., Cusumano C. K., Hultgren S. J. (2010). J. Med. Chem..

[cit113] Sperling O., Fuchs A., Lindhorst T. K. (2006). Org. Biomol. Chem..

[cit114] Klein T., Abgottspon D., Wittwer M., Rabbani S., Herold J., Jiang X., Kleeb S., Lüthi C., Scharenberg M., Bezençon J., Gubler E., Pang L., Smiesko M., Cutting B., Schwardt O., Ernst B. (2010). J. Med. Chem..

[cit115] Rillahan C. D., Macauley M. S., Schwartz E., He Y., McBride R., Arlian B. M., Rangarajan J., Fokin V. V., Paulson J. C. (2014). Chem. Sci..

[cit116] Sörme P., Qian Y., Nyholm P. G., Leffler H., Nilsson U. J. (2002). ChemBioChem.

[cit117] Cumpstey I., Salomonsson E., Sundin A., Leffler H., Nilsson U. J. (2008). Chem. – Eur. J..

[cit118] Kalas V., Hibbing M. E., Maddirala A. R., Chugani R., Pinkner J. S., Mydock-McGrane L. K., Conover M. S., Janetka J. W., Hultgren S. J. (2018). Proc. Natl. Acad. Sci. U. S. A..

[cit119] Maddirala A. R., Klein R., Pinkner J. S., Kalas V., Hultgren S. J., Janetka J. W. (2019). J. Med. Chem..

[cit120] Schwizer D., Gäthje H., Kelm S., Porro M., Schwardt O., Ernst B. (2006). Bioorg. Med. Chem..

[cit121] Kuhaudomlarp S., Siebs E., Shanina E., Topin J., Joachim I., da Silva Figueiredo Celestino Gomes P., Varrot A., Rognan D., Rademacher C., Imberty A., Titz A. (2021). Angew. Chem., Int. Ed..

[cit122] Shanina E., Kuhaudomlarp S., Siebs E., Fuchsberger F. F., Denis M., da Silva Figueiredo Celestino Gomes P., Clausen M. H., Seeberger P. H., Rognan D., Titz A., Imberty A., Rademacher C. (2022). Commun. Chem..

[cit123] Garber K. C. A., Wangkanont K., Carlson E. E., Kiessling L. L. (2010). Chem. Commun..

[cit124] Aretz J., Wamhoff E.-C., Hanske J., Heymann D., Rademacher C. (2014). Front. Immunol..

[cit125] Shanina E., Kuhaudomlarp S., Lal K., Seeberger P. H., Imberty A., Rademacher C. (2022). Angew. Chem., Int. Ed..

[cit126] Aretz J., Anumala U. R., Fuchsberger F. F., Molavi N., Ziebart N., Zhang H., Nazaré M., Rademacher C. (2018). J. Am. Chem. Soc..

[cit127] Dings R. P., Van Laar E. S., Webber J., Zhang Y., Griffin R. J., Waters S. J., MacDonald J. R., Mayo K. H. (2008). Cancer Lett..

[cit128] Dings R. P., Kumar N., Miller M. C., Loren M., Rangwala H., Hoye T. R., Mayo K. H. (2013). J. Pharmacol. Exp. Ther..

[cit129] Zhang H., Daněk O., Makarov D., Rádl S., Kim D., Ledvinka J., Vychodilová K., Hlaváč J., Lefèbre J., Denis M., Rademacher C., Ménová P. (2022). ACS Med. Chem. Lett..

[cit130] Chen Z., Cui Q., Caffrey M., Rong L., Du R. (2021). Pharmaceuticals.

[cit131] Bernardi A., Jiménez-Barbero J., Casnati A., De Castro C., Darbre T., Fieschi F., Finne J., Funken H., Jaeger K. E., Lahmann M., Lindhorst T. K., Marradi M., Messner P., Molinaro A., Murphy P. V., Nativi C., Oscarson S., Penadés S., Peri F., Pieters R. J., Renaudet O., Reymond J. L., Richichi B., Rojo J., Sansone F., Schäffer C., Turnbull W. B., Velasco-Torrijos T., Vidal S., Vincent S., Wennekes T., Zuilhof H., Imberty A. (2013). Chem. Soc. Rev..

[cit132] Pieters R. J. (2009). Org. Biomol. Chem..

[cit133] Cecioni S., Imberty A., Vidal S. (2015). Chem. Rev..

[cit134] Cummings R. D. , Chiffoleau E., van Kyook Y. and McEver R. P., in Essentials of Glycobiology, ed. A. Varki, R. D. Cummings, J. D. Esko, P. Stanley, G. W. Hart, M. Aebi, A. G. Darvill, T. Kinoshita, N. H. Packer, J. H. Prestegard, R. L. Schnaar and P. H. Seeberger, Cold Spring Harbor Laboratory Press, 2022, ch. 34 10.1101/glycobiology.4e.34 35536922

[cit135] Brown G. D., Willment J. A., Whitehead L. (2018). Nat. Rev. Immunol..

[cit136] Drickamer K., Taylor M. E. (2015). Curr. Opin. Struct. Biol..

[cit137] Mayer S., Raulf M. K., Lepenies B. (2017). Histochem. Cell Biol..

[cit138] Cramer J. (2021). RSC Med. Chem..

[cit139] Valverde P., Martinez J. D., Canada F. J., Arda A., Jimenez-Barbero J. (2020). ChemBioChem.

[cit140] Weis W. I., Drickamer K., Hendrickson W. A. (1992). Nature.

[cit141] Weis W. I., Kahn R., Fourme R., Drickamer K., Hendrickson W. A. (1991). Science.

[cit142] Zelensky A. N., Gready J. E. (2005). FEBS J..

[cit143] Taylor M. E., Drickamer K. (1993). J. Biol. Chem..

[cit144] McMahon S. A., Miller J. L., Lawton J. A., Kerkow D. E., Hodes A., Marti-Renom M. A., Doulatov S., Narayanan E., Sali A., Miller J. F., Ghosh P. (2005). Nat. Struct. Mol. Biol..

[cit145] Drickamer K. (1992). Nature.

[cit146] Weis W. I., Taylor M. E., Drickamer K. (1998). Immunol. Rev..

[cit147] Taylor M. E., Drickamer K. (2014). Curr. Opin. Struct. Biol..

[cit148] Sager C. P., Eris D., Smiesko M., Hevey R., Ernst B. (2017). Beilstein J. Org. Chem..

[cit149] Preston R. C., Jakob R. P., Binder F. P., Sager C. P., Ernst B., Maier T. (2016). J. Mol. Cell Biol..

[cit150] Ng K. K., Park-Snyder S., Weis W. I. (1998). Biochemistry.

[cit151] Kawasaki T., Ashwell G. (1977). J. Biol. Chem..

[cit152] Mc Dermott R., Ziylan U., Spehner D., Bausinger H., Lipsker D., Mommaas M., Cazenave J. P., Raposo G., Goud B., de la Salle H., Salamero J., Hanau D. (2002). Mol. Biol. Cell.

[cit153] Schwartz A. L., Geuze H. J., Lodish H. F. (1982). Philos. Trans. R. Soc., B.

[cit154] Brown J., O'Callaghan C. A., Marshall A. S., Gilbert R. J., Siebold C., Gordon S., Brown G. D., Jones E. Y. (2007). Protein Sci..

[cit155] Nagae M., Morita-Matsumoto K., Kato M., Kaneko M. K., Kato Y., Yamaguchi Y. (2014). Structure.

[cit156] Silva-Martin N., Bartual S. G., Ramirez-Aportela E., Chacon P., Park C. G., Hermoso J. A. (2014). Structure.

[cit157] Munoz-Garcia J. C., Chabrol E., Vives R. R., Thomas A., de Paz J. L., Rojo J., Imberty A., Fieschi F., Nieto P. M., Angulo J. (2015). J. Am. Chem. Soc..

[cit158] Hanske J., Wawrzinek R., Geissner A., Wamhoff E. C., Sellrie K., Schmidt H., Seeberger P. H., Rademacher C. (2017). ChemBioChem.

[cit159] Egger J., Weckerle C., Cutting B., Schwardt O., Rabbani S., Lemme K., Ernst B. (2013). J. Am. Chem. Soc..

[cit160] Aretz J., Baukmann H., Shanina E., Hanske J., Wawrzinek R., Zapol'skii V. A., Seeberger P. H., Kaufmann D. E., Rademacher C. (2017). Angew. Chem., Int. Ed..

[cit161] Aretz J., Anumala U. R., Fuchsberger F. F., Molavi N., Ziebart N., Zhang H., Nazare M., Rademacher C. (2018). J. Am. Chem. Soc..

[cit162] Wawrzinek R., Wamhoff E. C., Lefebre J., Rentzsch M., Bachem G., Domeniconi G., Schulze J., Fuchsberger F. F., Zhang H., Modenutti C., Schnirch L., Marti M. A., Schwardt O., Brautigam M., Guberman M., Hauck D., Seeberger P. H., Seitz O., Titz A., Ernst B., Rademacher C. (2021). J. Am. Chem. Soc..

[cit163] Huysamen C., Willment J. A., Dennehy K. M., Brown G. D. (2008). J. Biol. Chem..

[cit164] Shrimpton R. E., Butler M., Morel A.-S., Eren E., Hue S. S., Ritter M. A. (2009). Mol. Immunol..

[cit165] Kavanagh P. L., Fasipe T. A., Wun T. (2022). JAMA.

[cit166] Laubli H., Borsig L. (2010). Semin. Cancer Biol..

[cit167] Somers W. S., Tang J., Shaw G. D., Camphausen R. T. (2000). Cell.

[cit168] Binder F. P., Lemme K., Preston R. C., Ernst B. (2012). Angew. Chem., Int. Ed..

[cit169] Bedard P. W., Kaila N. (2010). Expert Opin. Ther. Pat..

[cit170] Kaila N., Thomas B. E. t (2002). Med. Res. Rev..

[cit171] Scheffler K., Brisson J. R., Weisemann R., Magnani J. L., Wong W. T., Ernst B., Peters T. (1997). J. Biomol. NMR.

[cit172] Norman K. E., Anderson G. P., Kolb H. C., Ley K., Ernst B. (1998). Blood.

[cit173] Schwizer D., Patton J. T., Cutting B., Smiesko M., Wagner B., Kato A., Weckerle C., Binder F. P., Rabbani S., Schwardt O., Magnani J. L., Ernst B. (2012). Chemistry.

[cit174] Chang J., Patton J. T., Sarkar A., Ernst B., Magnani J. L., Frenette P. S. (2010). Blood.

[cit175] Telen M. J., Wun T., McCavit T. L., De Castro L. M., Krishnamurti L., Lanzkron S., Hsu L. L., Smith W. R., Rhee S., Magnani J. L., Thackray H. (2015). Blood.

[cit176] Magnani J. L. , Presented in part at the ACS Spring Meeting 2021, 2021

[cit177] Dätwyler P., Jiang X., Wagner B., Varga N., Mühlethaler T., Hostettler K., Rabbani S., Schwardt O., Ernst B. (2022). ChemMedChem.

[cit178] Peterson J., Baek M.-G., Locatelli-Hoops S., Lee J.-W., Deng L., Stewart D. A., Smith T. A., Myers D. D., Fogler W. E., Magnani J. L. (2018). Blood.

[cit179] GlycoMimetics: GMI-1687, https://glycomimetics.com/pipeline/programs/gmi-1687/)

[cit180] Steele M. M., Radhakrishnan P., Magnani J. L., Hollingsworth M. A. (2014). Cancer Res..

[cit181] Muz B., Abdelghafer A., Markovic M., Yavner J., Melam A., Salama N. N., Azab A. K. (2021). Cancers.

[cit182] Barbier V., Erbani J., Fiveash C., Davies J. M., Tay J., Tallack M. R., Lowe J., Magnani J. L., Pattabiraman D. R., Perkins A. C., Lisle J., Rasko J. E. J., Levesque J.-P., Winkler I. G. (2020). Nat. Commun..

[cit183] Magnani J. L. and Fogler W. E., WO2019108750A1, 2019

[cit184] Gerlach L. O., Skerlj R. T., Bridger G. J., Schwartz T. W. (2001). J. Biol. Chem..

[cit185] Gravina G. L., Mancini A., Colapietro A., Monache S. D., Angelucci A., Calgani A., Fogler W. E., Magnani J. L., Festuccia C. (2015). Cancer Res..

[cit186] Steele M. M., Fogler W. E., Magnani J. L., Hollingsworth M. A. (2015). Cancer Res..

[cit187] Zhang W., Patel N., Fogler W. E., Magnani J. L., Andreeff M. (2015). Blood.

[cit188] Kogan T. P., Dupre B., Bui H., McAbee K. L., Kassir J. M., Scott I. L., Hu X., Vanderslice P., Beck P. J., Dixon R. A. (1998). J. Med. Chem..

[cit189] Hicks A. E., Abbitt K. B., Dodd P., Ridger V. C., Hellewell P. G., Norman K. E. (2005). J. Leukoc Biol..

[cit190] Beeh K. M., Beier J., Meyer M., Buhl R., Zahlten R., Wolff G. (2006). Pulm. Pharmacol. Ther..

[cit191] Watz H., Bock D., Meyer M., Schierhorn K., Vollhardt K., Woischwill C., Pedersen F., Kirsten A., Beeh K. M., Meyer-Sabellek W., Magnussen H., Beier J. (2013). Pulm. Pharmacol. Ther..

[cit192] Kirsten A., Watz H., Kretschmar G., Pedersen F., Bock D., Meyer-Sabellek W., Magnussen H. (2011). Pulm. Pharmacol. Ther..

[cit193] Kaila N., Janz K., Huang A., Moretto A., DeBernardo S., Bedard P. W., Tam S., Clerin V., Keith J. C., Tsao D. H. H., Sushkova N., Shaw G. D., Camphausen R. T., Schaub R. G., Wang Q. (2007). J. Med. Chem..

[cit194] Bedard P. W., Clerin V., Sushkova N., Tchernychev B., Antrilli T., Resmini C., Keith J. C., Hennan J. K., Kaila N., DeBernardo S. (2007). J. Pharmacol. Exp. Ther..

[cit195] Japp A. G., Chelliah R., Tattersall L., Lang N. N., Meng X., Weisel K., Katz A., Burt D., Fox K. A. A., Feuerstein G. Z., Connolly T. M., Newby D. E. (2013). J. Am. Heart Assoc..

[cit196] Geijtenbeek T. B., Torensma R., van Vliet S. J., van Duijnhoven G. C., Adema G. J., van Kooyk Y., Figdor C. G. (2000). Cell.

[cit197] Curtis B. M., Scharnowske S., Watson A. J. (1992). Proc. Natl. Acad. Sci. U. S. A..

[cit198] Chaipan C., Soilleux E. J., Simpson P., Hofmann H., Gramberg T., Marzi A., Geier M., Stewart E. A., Eisemann J., Steinkasserer A., Suzuki-Inoue K., Fuller G. L., Pearce A. C., Watson S. P., Hoxie J. A., Baribaud F., Pohlmann S. (2006). J. Virol..

[cit199] van Kooyk Y., Geijtenbeek T. B. H. (2003). Nat. Rev. Immunol..

[cit200] Geijtenbeek T. B., Kwon D. S., Torensma R., van Vliet S. J., van Duijnhoven G. C., Middel J., Cornelissen I. L., Nottet H. S., KewalRamani V. N., Littman D. R., Figdor C. G., van Kooyk Y. (2000). Cell.

[cit201] van Kooyk Y., Geijtenbeek T. B. (2003). Nat. Rev. Immunol..

[cit202] Geijtenbeek T. B., Van Vliet S. J., Koppel E. A., Sanchez-Hernandez M., Vandenbroucke-Grauls C. M., Appelmelk B., Van Kooyk Y. (2003). J. Exp. Med..

[cit203] Colmenares M., Puig-Kroger A., Pello O. M., Corbi A. L., Rivas L. (2002). J. Biol. Chem..

[cit204] Cambi A., Gijzen K., de Vries J., Torensma R., Joosten B., Adema G. J., Netea M. G., Kullberg B. J., Romani L., Figdor C. G. (2003). Eur. J. Immunol..

[cit205] Lu Q., Liu J., Zhao S., Gomez Castro M. F., Laurent-Rolle M., Dong J., Ran X., Damani-Yokota P., Tang H., Karakousi T., Son J., Kaczmarek M. E., Zhang Z., Yeung S. T., McCune B. T., Chen R. E., Tang F., Ren X., Chen X., Hsu J. C. C., Teplova M., Huang B., Deng H., Long Z., Mudianto T., Jin S., Lin P., Du J., Zang R., Su T. T., Herrera A., Zhou M., Yan R., Cui J., Zhu J., Zhou Q., Wang T., Ma J., Koralov S. B., Zhang Z., Aifantis I., Segal L. N., Diamond M. S., Khanna K. M., Stapleford K. A., Cresswell P., Liu Y., Ding S., Xie Q., Wang J. (2021). Immunity.

[cit206] Garber K. C., Wangkanont K., Carlson E. E., Kiessling L. L. (2010). Chem. Commun..

[cit207] Mari S., Serrano-Gomez D., Canada F. J., Corbi A. L., Jimenez-Barbero J. (2004). Angew. Chem..

[cit208] Thepaut M., Guzzi C., Sutkeviciute I., Sattin S., Ribeiro-Viana R., Varga N., Chabrol E., Rojo J., Bernardi A., Angulo J., Nieto P. M., Fieschi F. (2013). J. Am. Chem. Soc..

[cit209] Tomasic T., Hajsek D., Svajger U., Luzar J., Obermajer N., Petit-Haertlein I., Fieschi F., Anderluh M. (2014). Eur. J. Med. Chem..

[cit210] Medve L., Achilli S., Guzman-Caldentey J., Thepaut M., Senaldi L., Le Roy A., Sattin S., Ebel C., Vives C., Martin-Santamaria S., Bernardi A., Fieschi F. (2019). Chemistry.

[cit211] Mitchell D. A., Jones N. A., Hunter S. J., Cook J. M. D., Jenkinson S. F., Wormald M. R., Dwek R. A., Fleet G. W. J. (2007). Tetrahedron-Asymmetr.

[cit212] Bernardi A., Arosio D., Manzoni L., Micheli F., Pasquarello A., Seneci P. (2001). J. Org. Chem..

[cit213] Sutkeviciute I., Thepaut M., Sattin S., Berzi A., McGeagh J., Grudinin S., Weiser J., Le Roy A., Reina J. J., Rojo J., Clerici M., Bernardi A., Ebel C., Fieschi F. (2014). ACS Chem. Biol..

[cit214] Holla A., Skerra A. (2011). Protein Eng. Des. Sel..

[cit215] Guo Y., Feinberg H., Conroy E., Mitchell D. A., Alvarez R., Blixt O., Taylor M. E., Weis W. I., Drickamer K. (2004). Nat. Struct. Mol. Biol..

[cit216] Andreini M., Doknic D., Sutkeviciute I., Reina J. J., Duan J., Chabrol E., Thepaut M., Moroni E., Doro F., Belvisi L., Weiser J., Rojo J., Fieschi F., Bernardi A. (2011). Org. Biomol. Chem..

[cit217] Reina J. J., Sattin S., Invernizzi D., Mari S., Martínez-Prats L., Tabarani G., Fieschi F., Delgado R., Nieto P. M., Rojo J., Bernardi A. (2007). ChemMedChem.

[cit218] Varga N., Sutkeviciute I., Guzzi C., McGeagh J., Petit-Haertlein I., Gugliotta S., Weiser J., Angulo J., Fieschi F., Bernardi A. (2013). Chem. – Eur. J..

[cit219] Cramer J., Lakkaichi A., Aliu B., Jakob R. P., Klein S., Cattaneo I., Jiang X., Rabbani S., Schwardt O., Zimmer G., Ciancaglini M., Abreu Mota T., Maier T., Ernst B. (2021). J. Am. Chem. Soc..

[cit220] Ng S., Lin E., Kitov P. I., Tjhung K. F., Gerlits O. O., Deng L., Kasper B., Sood A., Paschal B. M., Zhang P., Ling C. C., Klassen J. S., Noren C. J., Mahal L. K., Woods R. J., Coates L., Derda R. (2015). J. Am. Chem. Soc..

[cit221] Thepaut M., Luczkowiak J., Vives C., Labiod N., Bally I., Lasala F., Grimoire Y., Fenel D., Sattin S., Thielens N., Schoehn G., Bernardi A., Delgado R., Fieschi F. (2021). PLoS Pathog.

[cit222] Timpano G., Tabarani G., Anderluh M., Invernizzi D., Vasile F., Potenza D., Nieto P. M., Rojo J., Fieschi F., Bernardi A. (2008). ChemBioChem.

[cit223] Bertolotti B., Oroszová B., Sutkeviciute I., Kniežo L., Fieschi F., Parkan K., Lovyová Z., Kašáková M., Moravcová J. (2016). Carbohydr. Res..

[cit224] Henrick K., Bawumia S., Barboni E. A., Mehul B., Hughes R. C. (1998). Glycobiology.

[cit225] Barondes S. H., Castronovo V., Cooper D. N. W., Cummings R. D., Drickamer K., Felzi T., Gitt M. A., Hirabayashi J., Hughes C., Kasai K.-I., Leffler H., Liu F.-T., Lotan R., Mercurio A. M., Monsigny M., Pillai S., Poirer F., Raz A., Rigby P. W. J., Rini J. M., Wang J. L. (1994). Cell.

[cit226] Modenutti C. P., Capurro J. I. B., Di Lella S., Martí M. A. (2019). Front. Chem..

[cit227] Ayona D., Fournier P.-E., Henrissat B., Desnues B. (2020). Front. Immunol..

[cit228] Arthur C. M. , Rodrigues L. C., Baruffi M. D., Sullivan H. C., Heimburg-Molinaro J., Smith D. F., Cummings R. D. and Stowell S. R., Galectins, Springer, 2015, pp. 115–131 10.1007/978-1-4939-1396-1_8 PMC5755360 25253137

[cit229] Le Mercier M., Mathieu V., Haibe-Kains B., Bontempi G., Mijatovic T., Decaestecker C., Kiss R., Lefranc F. (2008). J. Neuropathol. Exp. Neurol..

[cit230] Kiefer M. C., Brauer M. J., Powers V. C., Wu J. J., Umansky S. R., Tomei L. D., Barr P. J. (1995). Nature.

[cit231] Nakahara S., Oka N., Raz A. (2005). Apoptosis.

[cit232] Cummings R. D. , Liu F.-T., Rabinovich G. A., Stowell S. R. and Vasta G. R., Essentials of glycobiology, Cold Spring Harbor Laboratory Press, 4th edn, 2022, ch. 36 10.1101/glycobiology.4e.36

[cit233] Liu F. T., Rabinovich G. A. (2010). Ann. NY Acad. Sci..

[cit234] Thiemann S., Baum L. G. (2016). Annu. Rev. Immunol..

[cit235] Ilarregui J., Bianco G., Toscano M., Rabinovich G. (2005). Ann. Rheum. Dis..

[cit236] Lahm H., André S., Hoeflich A., Kaltner H., Siebert H.-C., Sordat B., von der Lieth C.-W., Wolf E., Gabius H.-J. (2003). Glycoconjugate J..

[cit237] Girotti M. R., Salatino M., Dalotto-Moreno T., Rabinovich G. A. (2020). J. Exp. Med..

[cit238] Takenaka Y., Fukumori T., Raz A. (2002). Glycoconj. J..

[cit239] Liu F.-T., Rabinovich G. A. (2005). Nat. Rev. Cancer.

[cit240] Camby I., Belot N., Lefranc F., Sadeghi N., de Launoit Y., Kaltner H., Musette S., Darro F., Danguy A., Salmon I., Gabius H.-J., Kiss R. (2002). J. Neuropathol. Exp. Neurol..

[cit241] Hittelet A., Legendre H., Nagy N., Bronckart Y., Pector J.-C., Salmon I., Yeaton P., Gabius H.-J., Kiss R., Camby I. (2003). Int. J. Cancer.

[cit242] Cagnoni A. J., Perez Saez J. M., Rabinovich G. A., Mariño K. V. (2016). Front. Oncol..

[cit243] Leffler H., Carlsson S., Hedlund M., Qian Y., Poirier F. (2002). Glycoconjugate J..

[cit244] Hsieh T.-J., Lin H.-Y., Tu Z., Huang B.-S., Wu S.-C., Lin C.-H. (2015). PLoS One.

[cit245] Chan Y.-C., Lin H.-Y., Tu Z., Kuo Y.-H., Hsu S.-T. D., Lin C.-H. (2018). Int. J. Mol. Sci..

[cit246] Oberg C. T., Leffler H., Nilsson U. J. (2011). Chimia (Aarau).

[cit247] Blanchard H., Yu X., Collins P. M., Bum-Erdene K. (2014). Expert Opin. Ther. Pat..

[cit248] Blanchard H., Bum-Erdene K., Bohari M. H., Yu X. (2016). Expert Opin. Ther. Pat..

[cit249] Girard A., Magnani J. L. (2018). Trends Glycosci. Gly..

[cit250] Argüeso P., Panjwani N. (2011). Exp. Eye Res..

[cit251] Henderson N. C., Mackinnon A. C., Farnworth S. L., Poirier F., Russo F. P., Iredale J. P., Haslett C., Simpson K. J., Sethi T. (2006). Proc. Natl. Acad. Sci. U. S. A..

[cit252] Henderson N. C., Mackinnon A. C., Farnworth S. L., Kipari T., Haslett C., Iredale J. P., Liu F. T., Hughes J., Sethi T. (2008). Am. J. Pathol..

[cit253] Nishi Y., Sano H., Kawashima T., Okada T., Kuroda T., Kikkawa K., Kawashima S., Tanabe M., Goto T., Matsuzawa Y., Matsumura R., Tomioka H., Liu F.-T., Shirai K. (2007). Allergol. Int..

[cit254] MacKinnon A. C., Gibbons M. A., Farnworth S. L., Leffler H., Nilsson U. J., Delaine T., Simpson A. J., Forbes S. J., Hirani N., Gauldie J., Sethi T. (2012). Am. J. Resp. Crit. Care Med..

[cit255] Zou J., Glinsky V. V., Landon L. A., Matthews L., Deutscher S. L. (2005). Carcinogenesis.

[cit256] Newton-Northup J. R., Dickerson M. T., Ma L., Besch-Williford C. L., Deutscher S. L. (2013). Clin. Exp. Metastasis.

[cit257] Yang Y., Zhou Z., He S., Fan T., Jin Y., Zhu X., Chen C., Zhang Z.-R., Huang Y. (2012). Biomaterials.

[cit258] Sun W., Li L., Yang Q., Shan W., Zhang Z., Huang Y. (2015). Mol. Pharm..

[cit259] Sun W., Li L., Li L.-J., Yang Q.-Q., Zhang Z.-R., Huang Y. (2017). Acta Pharmacol. Sin..

[cit260] Zhang W., Xu P., Zhang H. (2015). Trends Food Sci. Technol..

[cit261] Chauhan D., Li G., Podar K., Hideshima T., Neri P., He D., Mitsiades N., Richardson P., Chang Y., Schindler J., Carver B., Anderson K. C. (2005). Cancer Res..

[cit262] Grous J. J., Redfern C. H., Mahadevan D., Schindler J. (2006). J. Clin. Oncol..

[cit263] Cotter F., Smith D. A., Boyd T. E., Richards D. A., Alemany C., Loesch D., Salogub G., Tidmarsh G. F., Gammon G. M., Gribben J. (2009). J. Clin. Oncol..

[cit264] Jolla L. , La Jolla Pharmaceutical Company Reports Positive, Top-Line Results from Phase 2 Clinical Trial of GCS-100 in Chronic Kidney Disease, https://www.sec.gov/Archives/edgar/data/920465/000092046514000012/pressreleasedatamar10.htm)

[cit265] Traber P. G., Zomer E. (2013). PLoS One.

[cit266] Traber P. G., Chou H., Zomer E., Hong F., Klyosov A., Fiel M.-I., Friedman S. L. (2013). PLoS One.

[cit267] Chalasani N., Abdelmalek M. F., Garcia-Tsao G., Vuppalanchi R., Alkhouri N., Rinella M., Noureddin M., Pyko M., Shiffman M., Sanyal A., Allgood A., Shlevin H., Horton R., Zomer E., Irish W., Goodman Z., Harrison S. A., Traber P. G., Abdelmalek M., Balart L., Borg B., Chalasani N., Charlton M., Conjeevaram H., Fuchs M., Ghalib R., Gholam P., Halegoua-De Marzio D., Harrison S., Jue C., Kemmer N., Kowdley K., Lai M., Lawitz E., Loomba R., Noureddin M., Paredes A., Rinella M., Rockey D., Rodriguez M., Rubin R., Ryan M., Sanyal A., Scanga A., Sepe T., Shiffman M., Shiffman M., Tetri B., Thuluvath P., Torres D., Vierling J., Wattacheril J., Weiland A., Zogg D. (2020). Gastroenterology.

[cit268] Curti B. D., Koguchi Y., Leidner R. S., Rolig A. S., Sturgill E. R., Sun Z., Wu Y., Rajamanickam V., Bernard B., Hilgart-Martiszus I. (2021). J. Immunother. Cancer.

[cit269] Klyosov A. , Zomer E. and Platt D., Glycobiology and Drug Design, American Chemical Society, 2012, vol. 1102, ch. 4, pp. 89–130

[cit270] Sörme P., Qian Y., Nyholm P.-G., Leffler H., Nilsson U. J. (2002). ChemBioChem.

[cit271] Sörme P., Arnoux P., Kahl-Knutsson B., Leffler H., Rini J. M., Nilsson U. J. (2005). J. Am. Chem. Soc..

[cit272] Öberg C. T., Leffler H., Nilsson U. J. (2008). J. Med. Chem..

[cit273] Öberg C. T., Noresson A.-L., Leffler H., Nilsson U. J. (2011). Chem. – Eur. J..

[cit274] Fort S., Kim H.-S., Hindsgaul O. (2006). J. Org. Chem..

[cit275] van Hattum H., Branderhorst H. M., Moret E. E., Nilsson U. J., Leffler H., Pieters R. J. (2013). J. Med. Chem..

[cit276] Cumpstey I., Salomonsson E., Sundin A., Leffler H., Nilsson U. J. (2007). ChemBioChem.

[cit277] Gallivan J. P., Dougherty D. A. (2000). J. Am. Chem. Soc..

[cit278] Cumpstey I., Sundin A., Leffler H., Nilsson U. J. (2005). Angew. Chem., Int. Ed..

[cit279] Salameh B. A., Cumpstey I., Sundin A., Leffler H., Nilsson U. J. (2010). Bioorg. Med. Chem..

[cit280] Henderson N. , Sethi T., Mackinnon A., Leffler H. and Nilsson U., Canadian Pat., CA2794066C, 2017

[cit281] Hirani N., Nicol L., MacKinnon A. C., Ford P., Schambye H., Pedersen, Nilsson U., Leffler H., Thomas T., Knott O., Gibbons M., Simpson J., Maher T. (2016). QJM: Int. J. Med..

[cit282] Hirani N., MacKinnon A. C., Nicol L., Ford P., Schambye H., Pedersen A., Nilsson U. J., Leffler H., Sethi T., Tantawi S., Gravelle L., Slack R. J., Mills R., Karmakar U., Humphries D., Zetterberg F., Keeling L., Paul L., Molyneaux P. L., Li F., Funston W., Forrest I. A., Simpson A. J., Gibbons M. A., Maher T. M. (2021). Eur. Respir. J..

[cit283] Gaughan E., Quinn T., Bruce A., Antonelli J., Young V., Mair J., Akram A., Hirani N., Koch O., Mackintosh C., Norrie J., Dear J. W., Dhaliwal K. (2021). BMJ Open.

[cit284] Rajput V. K., MacKinnon A., Mandal S., Collins P., Blanchard H., Leffler H., Sethi T., Schambye H., Mukhopadhyay B., Nilsson U. J. (2016). J. Med. Chem..

[cit285] Bertuzzi S., Quintana J. I., Ardá A., Gimeno A., Jiménez-Barbero J. (2020). Front. Chem..

[cit286] Zetterberg F. R., Peterson K., Johnsson R. E., Brimert T., Håkansson M., Logan D. T., Leffler H., Nilsson U. J. (2018). ChemMedChem.

[cit287] Zetterberg F. R., MacKinnon A., Brimert T., Gravelle L., Johnsson R. E., Kahl-Knutson B., Leffler H., Nilsson U. J., Pedersen A., Peterson K., Roper J. A., Schambye H., Slack R. J., Tantawi S. (2022). J. Med. Chem..

[cit288] Brimert T. , Johnsson R., Leffler H., Nilsson U. and Zetterberg F., WO2016120403, 2016

[cit289] Zetterberg F. , Nilsson U. and Leffler H., WO2018011094, 2018

[cit290] Galecto Announces First Patient Treated in Phase 2 Trial of Oral Galectin-3 Inhibitor GB1211 in Liver CirrhosisGalecto now has three ongoing Phase 2 clinical trials with three different drug candidates in three high value indications, https://www.biospace.com/article/releases/galecto-announces-first-patient-treated-in-phase-2-trial-of-oral-galectin-3-inhibitor-gb1211-in-liver-cirrhosisgalecto-now-has-three-ongoing-phase-2-clinical-trials-with-three-different-drug-candidates-in-three-high-value-indications/

[cit291] Liu C., Jalagam P. R., Feng J., Wang W., Raja T., Sura M. R., Manepalli R. K. V. L. P., Aliphedi B. R., Medavarapu S., Nair S. K., Muthalagu V., Natesan R., Gupta A., Beno B., Panda M., Ghosh K., Shukla J. K., Sale H., Haldar P., Kalidindi N., Shah D., Patel D., Mathur A., Ellsworth B. A., Cheng D., Regueiro-Ren A. (2022). J. Med. Chem..

[cit292] Delaine T., Collins P., MacKinnon A., Sharma G., Stegmayr J., Rajput V. K., Mandal S., Cumpstey I., Larumbe A., Salameh B. A., Kahl-Knutsson B., van Hattum H., van Scherpenzeel M., Pieters R. J., Sethi T., Schambye H., Oredsson S., Leffler H., Blanchard H., Nilsson U. J. (2016). ChemBioChem.

[cit293] Thijssen V. L., Heusschen R., Caers J., Griffioen A. W. (2015). Biochim. Biophys. Acta, Rev. Cancer.

[cit294] Perillo N. L., Marcus M. E., Baum L. G. (1998). J. Mol. Med..

[cit295] Rubinstein N., Alvarez M., Zwirner N. W., Toscano M. A., Ilarregui J. M., Bravo A., Mordoh J., Fainboim L., Podhajcer O. L., Rabinovich G. A. (2004). Cancer Cell.

[cit296] D'Haene N., Sauvage S., Maris C., Adanja I., Le Mercier M., Decaestecker C., Baum L., Salmon I. (2013). PLoS One.

[cit297] Toscano M. A., Bianco G. A., Ilarregui J. M., Croci D. O., Correale J., Hernandez J. D., Zwirner N. W., Poirier F., Riley E. M., Baum L. G., Rabinovich G. A. (2007). Nat. Immunol..

[cit298] Ouellet M., Mercier S., Pelletier I., Bounou S., Roy J., Hirabayashi J., Sato S., Tremblay M. J. (2005). J. Immunol..

[cit299] St-Pierre C., Manya H., Ouellet M., Clark G. F., Endo T., Tremblay M. J., Sato S. (2011). J. Virol..

[cit300] St-Pierre C., Ouellet M., Giguère D., Ohtake R., Roy R., Sato S., Tremblay M. J. (2012). Antimicrob. Agents Chemother..

[cit301] Peterson K., Collins P. M., Huang X., Kahl-Knutsson B., Essén S., Zetterberg F. R., Oredsson S., Leffler H., Blanchard H., Nilsson U. J. (2018). RSC Adv..

[cit302] Pal K. B., Mahanti M., Leffler H., Nilsson U. J. (2019). Int. J. Mol. Sci..

[cit303] Dahlqvist A., Furevi A., Warlin N., Leffler H., Nilsson U. J. (2019). Beilstein J. Org. Chem..

[cit304] Wang J. B., Wang M. D., Li E. X., Dong D. F. (2012). Peptides.

[cit305] Griffioen A. W., van der Schaft D. W. J., Barendsz-Janson A. F., Cox A., Boudier H. A. J. S., Hillen H. F. P., Mayo K. H. (2001). Biochem. J..

[cit306] Brandwijk R. J. M. G. E., Dings R. P. M., van der Linden E., Mayo K. H., Thijssen V. L. J. L., Griffioen A. W. (2006). Biochem. Biophys. Res. Commun..

[cit307] Salomonsson E., Thijssen V. L., Griffioen A. W., Nilsson U. J., Leffler H. (2011). J. Biol. Chem..

[cit308] Mayo K. H., Dings R. P. M., Flader C., Nesmelova I., Hargittai B., van der Schaft D. W. J., van Eijk L. I., Walek D., Haseman J., Hoye T. R., Griffioen A. W. (2003). J. Biol. Chem..

[cit309] Dings R. P. M., Chen X., Hellebrekers D. M. E. I., van Eijk L. I., Zhang Y., Hoye T. R., Griffioen A. W., Mayo K. H. (2006). JNCI: J. Natl. Cancer I..

[cit310] Dings R. P. M., Miller M. C., Nesmelova I., Astorgues-Xerri L., Kumar N., Serova M., Chen X., Raymond E., Hoye T. R., Mayo K. H. (2012). J. Med. Chem..

[cit311] Astorgues-Xerri L., Riveiro M. E., Tijeras-Raballand A., Serova M., Rabinovich G. A., Bieche I., Vidaud M., de Gramont A., Martinet M., Cvitkovic E., Faivre S., Raymond E. (2014). Eur. J. Cancer.

[cit312] Zucchetti M., Bonezzi K., Frapolli R., Sala F., Borsotti P., Zangarini M., Cvitkovic E., Noel K., Ubezio P., Giavazzi R., D’Incalci M., Taraboletti G. (2013). Cancer Chemother. Pharmacol..

[cit313] Rezai K., Durand S., Lachaux N., Raymond E., Herait P., Lokiec F. (2013). Cancer Res..

[cit314] Yang R., Sun L., Li C.-F., Wang Y.-H., Yao J., Li H., Yan M., Chang W.-C., Hsu J.-M., Cha J.-H., Hsu J. L., Chou C.-W., Sun X., Deng Y., Chou C.-K., Yu D., Hung M.-C. (2021). Nat. Commun..

[cit315] Bailly C., Thuru X., Quesnel B. (2021). Cancers.

[cit316] Mandal S., Rajput V. K., Sundin A. P., Leffler H., Mukhopadhyay B., Nilsson U. J. (2016). Can. J. Chem..

[cit317] Mahanti M., Pal K. B., Sundin A. P., Leffler H., Nilsson U. J. (2020). ACS Med. Chem. Lett..

[cit318] Chen W.-S., Cao Z., Sugaya S., Lopez M. J., Sendra V. G., Laver N., Leffler H., Nilsson U. J., Fu J., Song J., Xia L., Hamrah P., Panjwani N. (2016). Nat. Commun..

[cit319] Tribulatti M. V., Carabelli J., Prato C. A., Campetella O. (2019). Glycobiology.

[cit320] Sampson J. F., Suryawanshi A., Chen W.-S., Rabinovich G. A., Panjwani N. (2016). Immunol. Cell Biol..

[cit321] Bohari M. H., Yu X., Kishor C., Patel B., Go R. M., Eslampanah Seyedi H. A., Vinik Y., Grice I. D., Zick Y., Blanchard H. (2018). ChemMedChem.

[cit322] Patel B., Kishor C., Houston T. A., Shatz-Azoulay H., Zick Y., Vinik Y., Blanchard H. (2020). J. Med. Chem..

[cit323] Wu C., Yong C., Zhong Q., Wang Z., Nilsson U. J., Zhang Y. (2020). RSC Adv..

[cit324] Hassan M., van Klaveren S., Håkansson M., Diehl C., Kovačič R., Baussière F., Sundin A. P., Dernovšek J., Walse B., Zetterberg F., Leffler H., Anderluh M., Tomašič T., Jakopin Ž., Nilsson U. J. (2021). Eur. J. Med. Chem..

[cit325] Hassan M., Baussière F., Guzelj S., Sundin A. P., Håkansson M., Kovačič R., Leffler H., Tomašič T., Anderluh M., Jakopin Ž., Nilsson U. J. (2021). ACS Med. Chem. Lett..

[cit326] van Klaveren S., Sundin A. P., Jakopin Ž., Anderluh M., Leffler H., Nilsson U. J., Tomašič T. (2022). ChemMedChem.

[cit327] Girardi B., Manna M., Van Klaveren S., Tomašič T., Jakopin Ž., Leffler H., Nilsson U. J., Ricklin D., Mravljak J., Schwardt O., Anderluh M. (2022). ChemMedChem.

[cit328] Varki A. (1992). Glycobiology.

[cit329] Macauley M. S., Crocker P. R., Paulson J. C. (2014). Nat. Rev. Immunol..

[cit330] Duan S., Paulson J. C. (2020). Annu. Rev. Immunol..

[cit331] Zhu Y., Yao S., Chen L. (2011). Immunity.

[cit332] Ereno-Orbea J., Sicard T., Cui H., Mazhab-Jafari M. T., Benlekbir S., Guarne A., Rubinstein J. L., Julien J. P. (2017). Nat. Commun..

[cit333] Pronker M. F., Lemstra S., Snijder J., Heck A. J. R., Thies-Weesie D. M. E., Pasterkamp R. J., Janssen B. J. C. (2016). Nat. Commun..

[cit334] Angata T. (2020). Adv. Exp. Med. Biol..

[cit335] Angata T., Nycholat C. M., Macauley M. S. (2015). Trends Pharmacol. Sci..

[cit336] Kiwamoto T., Kawasaki N., Paulson J. C., Bochner B. S. (2012). Pharmacol. Ther..

[cit337] Wang J., Sun J., Liu L. N., Flies D. B., Nie X., Toki M., Zhang J., Song C., Zarr M., Zhou X., Han X., Archer K. A., O'Neill T., Herbst R. S., Boto A. N., Sanmamed M. F., Langermann S., Rimm D. L., Chen L. (2019). Nat. Med..

[cit338] DiJoseph J. F., Armellino D. C., Boghaert E. R., Khandke K., Dougher M. M., Sridharan L., Kunz A., Hamann P. R., Gorovits B., Udata C., Moran J. K., Popplewell A. G., Stephens S., Frost P., Damle N. K. (2004). Blood.

[cit339] van Der Velden V. H., te Marvelde J. G., Hoogeveen P. G., Bernstein I. D., Houtsmuller A. B., Berger M. S., van Dongen J. J. (2001). Blood.

[cit340] Miles L. A., Hermans S. J., Crespi G. A. N., Gooi J. H., Doughty L., Nero T. L., Markulić J., Ebneth A., Wroblowski B., Oehlrich D., Trabanco A. A., Rives M. L., Royaux I., Hancock N. C., Parker M. W. (2019). iScience.

[cit341] Lenza M. P., Atxabal U., Oyenarte I., Jimenez-Barbero J., Ereno-Orbea J. (2020). Cells.

[cit342] Angata T., Varki A. (2002). Chem. Rev..

[cit343] Chen X., Varki A. (2010). ACS Chem. Biol..

[cit344] Kelm S., Brossmer R., Isecke R., Gross H. J., Strenge K., Schauer R. (1998). Eur. J. Biochem..

[cit345] Kelm S., Madge P., Islam T., Bennett R., Koliwer-Brandl H., Waespy M., von Itzstein M., Haselhorst T. (2013). Angew. Chem., Int. Ed..

[cit346] Prescher H., Schweizer A., Kuhfeldt E., Nitschke L., Brossmer R. (2017). ChemBioChem.

[cit347] Rillahan C. D., Schwartz E., Rademacher C., McBride R., Rangarajan J., Fokin V. V., Paulson J. C. (2013). ACS Chem. Biol..

[cit348] Zhuravleva M. A., Trandem K., Sun P. D. (2008). J. Mol. Biol..

[cit349] Attrill H., Imamura A., Sharma R. S., Kiso M., Crocker P. R., Van Aalten D. M. (2006). J. Biol. Chem..

[cit350] Zaccai N. R., Maenaka K., Maenaka T., Crocker P. R., Brossmer R., Kelm S., Jones E. Y. (2003). Structure.

[cit351] Collins B. E., Blixt O., Han S., Duong B., Li H., Nathan J. K., Bovin N., Paulson J. C. (2006). J. Immunol..

[cit352] Zeng Y., Rademacher C., Nycholat C. M., Futakawa S., Lemme K., Ernst B., Paulson J. C. (2011). Bioorg. Med. Chem. Lett..

[cit353] Nycholat C. M., Rademacher C., Kawasaki N., Paulson J. C. (2012). J. Am. Chem. Soc..

[cit354] Cagnoni A. J., Perez Saez J. M., Rabinovich G. A., Marino K. V. (2016). Front. Oncol..

[cit355] Liu G. j, Jia L. y, Xing G. w (2019). Asian J. Org. Chem..

[cit356] Nycholat C. M., Duan S., Knuplez E., Worth C., Elich M., Yao A., O’Sullivan J., McBride R., Wei Y., Fernandes S. M., Zhu Z., Schnaar R. L., Bochner B. S., Paulson J. C. (2019). J. Am. Chem. Soc..

[cit357] Collins B. E., Blixt O., DeSieno A. R., Bovin N., Marth J. D., Paulson J. C. (2004). Proc. Natl. Acad. Sci. U. S. A..

[cit358] Han S., Collins B. E., Bengtson P., Paulson J. C. (2005). Nat. Chem. Biol..

[cit359] May A. P., Robinson R. C., Vinson M., Crocker P. R., Jones E. Y. (1998). Mol. Cell.

[cit360] Kelm S., Gerlach J., Brossmer R., Danzer C. P., Nitschke L. (2002). J. Exp. Med..

[cit361] Blixt O., Han S., Liao L., Zeng Y., Hoffmann J., Futakawa S., Paulson J. C. (2008). J. Am. Chem. Soc..

[cit362] Chen W. C., Kawasaki N., Nycholat C. M., Han S., Pilotte J., Crocker P. R., Paulson J. C. (2012). PLoS One.

[cit363] Kawasaki N., Vela J. L., Nycholat C. M., Rademacher C., Khurana A., van Rooijen N., Crocker P. R., Kronenberg M., Paulson J. C. (2013). Proc. Natl. Acad. Sci. U. S. A..

[cit364] Edgar L. J., Kawasaki N., Nycholat C. M., Paulson J. C. (2019). Cell Chem. Biol..

[cit365] Duan S., Arlian B. M., Nycholat C. M., Wei Y., Tateno H., Smith S. A., Macauley M. S., Zhu Z., Bochner B. S., Paulson J. C. (2021). J. Immunol..

[cit366] Collins B. E., Blixt O., Han S., Duong B., Li H., Nathan J. K., Bovin N., Paulson J. C. (2006). J. Immunol..

[cit367] Macauley M. S., Pfrengle F., Rademacher C., Nycholat C. M., Gale A. J., von Drygalski A., Paulson J. C. (2013). J. Clin. Invest..

[cit368] Hardy L. C., Smeekens J., Raghuwanshi D., Sarkar S., Daskhan G. C., Rogers S., Nycholat C., Maleki S., Burks A. W., Paulson J. C., Macauley M. S., Kulis M. D. (2022). J. Allergy Clin. Immunol..

[cit369] Forgione R. E., Nieto F. F., Di Carluccio C., Milanesi F., Fruscella M., Papi F., Nativi C., Molinaro A., Palladino P., Scarano S., Minunni M., Montefiori M., Civera M., Sattin S., Francesconi O., Marchetti R., Silipo A. (2022). Chembiochem.

[cit370] Kim B., Shin J., Kiziltepe T., Bilgicer B. (2020). Nanoscale.

[cit371] Miethke M., Pieroni M., Weber T., Brönstrup M., Hammann P., Halby L., Arimondo P. B., Glaser P., Aigle B., Bode H. B., Moreira R., Li Y., Luzhetskyy A., Medema M. H., Pernodet J. L., Stadler M., Tormo J. R., Genilloud O., Truman A. W., Weissman K. J., Takano E., Sabatini S., Stegmann E., Brötz-Oesterhelt H., Wohlleben W., Seemann M., Empting M., Hirsch A. K. H., Loretz B., Lehr C. M., Titz A., Herrmann J., Jaeger T., Alt S., Hesterkamp T., Winterhalter M., Schiefer A., Pfarr K., Hoerauf A., Graz H., Graz M., Lindvall M., Ramurthy S., Karlén A., van Dongen M., Petkovic H., Keller A., Peyrane F., Donadio S., Fraisse L., Piddock L. J. V., Gilbert I. H., Moser H. E., Müller R. (2021). Nat. Rev. Chem..

[cit372] Calvert M. B., Jumde V. R., Titz A. (2018). Beilstein J. Org. Chem..

[cit373] Clatworthy A. E., Pierson E., Hung D. T. (2007). Nat. Chem. Biol..

[cit374] Mulvey G. L., Marcato P., Kitov P. I., Sadowska J., Bundle D. R., Armstrong G. D. (2003). J. Infect. Dis..

[cit375] Nicolle L. E. (2002). Clin. Microbiol. Newsl..

[cit376] Harding G. K., Ronald A. R. (1994). Int. J. Antimicrob. Agents.

[cit377] Flores-Mireles A. L., Walker J. N., Caparon M., Hultgren S. J. (2015). Nat. Rev. Microbiol..

[cit378] Ronald A. R. (2002). Am. J. Med..

[cit379] Eto D. S., Jones T. A., Sundsbak J. L., Mulvey M. A. (2007). PLoS Pathog..

[cit380] Abgottspon D., Ernst B. (2012). Chimia (Aarau).

[cit381] Wright K. J., Seed P. C., Hultgren S. J. (2007). Cell. Microbiol..

[cit382] Sivignon A., Bouckaert J., Bernard J., Gouin S. G., Barnich N. (2017). Expert Opin. Ther. Tar..

[cit383] Chevalier G., Laveissière A., Desachy G., Barnich N., Sivignon A., Maresca M., Nicoletti C., Di Pasquale E., Martinez-Medina M., Simpson K. W., Yajnik V., Sokol H., s. investigators M., Plassais J., Strozzi F., Cervino A., Morra R., Bonny C. (2021). Microbiome.

[cit384] Hahn E., Wild P., Hermanns U., Sebbel P., Glockshuber R., Häner M., Taschner N., Burkhard P., Aebi U., Müller S. A. (2002). J. Mol. Biol..

[cit385] Waksman G., Hultgren S. J. (2009). Nat. Rev. Microbiol..

[cit386] Choudhury D., Thompson A., Stojanoff V., Langermann S., Pinkner J., Hultgren S. J., Knight S. D. (1999). Science.

[cit387] Remaut H., Rose R. J., Hannan T. J., Hultgren S. J., Radford S. E., Ashcroft A. E., Waksman G. (2006). Mol. Cell.

[cit388] Aprikian P., Tchesnokova V., Kidd B., Yakovenko O., Yarov-Yarovoy V., Trinchina E., Vogel V., Thomas W., Sokurenko E. (2007). J. Biol. Chem..

[cit389] Sauer M. M., Jakob R. P., Eras J., Baday S., Eriş D., Navarra G., Bernèche S., Ernst B., Maier T., Glockshuber R. (2016). Nat. Commun..

[cit390] Sauer M. M., Jakob R. P., Luber T., Canonica F., Navarra G., Ernst B., Unverzagt C., Maier T., Glockshuber R. (2019). J. Am. Chem. Soc..

[cit391] Thomas W. E., Nilsson L. M., Forero M., Sokurenko E. V., Vogel V. (2004). Mol. Microbiol..

[cit392] Le Trong I., Aprikian P., Kidd B. A., Forero-Shelton M., Tchesnokova V., Rajagopal P., Rodriguez V., Interlandi G., Klevit R., Vogel V., Stenkamp R. E., Sokurenko E. V., Thomas W. E. (2010). Cell.

[cit393] Zhou G., Mo W.-J., Sebbel P., Min G., Neubert T. A., Glockshuber R., Wu X.-R., Sun T.-T., Kong X.-P. (2001). J. Cell Sci..

[cit394] Barnich N., Carvalho F. A., Glasser A.-L., Darcha C., Jantscheff P., Allez M., Peeters H., Bommelaer G., Desreumaux P., Colombel J. F., Darfeuille-Michaud A. (2007). J. Clin. Invest..

[cit395] Hung C. S., Bouckaert J., Hung D., Pinkner J., Widberg C., DeFusco A., Auguste C. G., Strouse R., Langermann S., Waksman G., Hultgren S. J. (2002). Mol. Microbiol..

[cit396] Vandemaele F., Vandekerchove D., Vereecken M., Derijcke J., Dho-Moulin M., Goddeeris B. M. (2003). Vet. Res..

[cit397] Vandemaele F. J., Hensen S. M., Goddeeris B. M. (2004). Vet. Microbiol..

[cit398] Abraham S. N., Sun D., Dale J. B., Beachey E. H. (1988). Nature.

[cit399] Connell I., Agace W., Klemm P., Schembri M., Mårild S., Svanborg C. (1996). Proc. Natl. Acad. Sci. U. S. A..

[cit400] Langermann S., Palaszynski S., Barnhart M., Auguste G., Pinkner J. S., Burlein J., Barren P., Koenig S., Leath S., Jones C. H., Hultgren S. J. (1997). Science.

[cit401] Thankavel K., Madison B., Ikeda T., Malaviya R., Shah A. H., Arumugam P. M., Abraham S. N. (1997). J. Clin. Invest..

[cit402] Bouckaert J., Berglund J., Schembri M., De Genst E., Cools L., Wuhrer M., Hung C. S., Pinkner J., Slättegård R., Zavialov A., Choudhury D., Langermann S., Hultgren S. J., Wyns L., Klemm P., Oscarson S., Knight S. D., De Greve H. (2005). Mol. Microbiol..

[cit403] Bouckaert J., Mackenzie J., de Paz J. L., Chipwaza B., Choudhury D., Zavialov A., Mannerstedt K., Anderson J., Piérard D., Wyns L., Seeberger P. H., Oscarson S., De Greve H., Knight S. D. (2006). Mol. Microbiol..

[cit404] Wellens A., Garofalo C., Nguyen H., Van Gerven N., Slättegård R., Hernalsteens J. P., Wyns L., Oscarson S., De Greve H., Hultgren S., Bouckaert J. (2008). PLOS One.

[cit405] Wellens A., Lahmann M., Touaibia M., Vaucher J., Oscarson S., Roy R., Remaut H., Bouckaert J. (2012). Biochemistry.

[cit406] Bouckaert J., Berglund J., Schembri M., De Genst E., Cools L., Wuhrer M., Hung C. S., Pinkner J., Slättegård R., Zavialov A., Choudhury D., Langermann S., Hultgren S. J., Wyns L., Klemm P., Oscarson S., Knight S. D., De Greve H. (2005). Mol. Microbiol..

[cit407] Mydock-McGrane L. K., Hannan T. J., Janetka J. W. (2017). Expert Opin. Drug Dis..

[cit408] Remaut H., Tang C., Henderson N. S., Pinkner J. S., Wang T., Hultgren S. J., Thanassi D. G., Waksman G., Li H. (2008). Cell.

[cit409] Han Z., Pinkner J. S., Ford B., Obermann R., Nolan W., Wildman S. A., Hobbs D., Ellenberger T., Cusumano C. K., Hultgren S. J., Janetka J. W. (2010). J. Med. Chem..

[cit410] Sager C. P., Fiege B., Zihlmann P., Vannam R., Rabbani S., Jakob R. P., Preston R. C., Zalewski A., Maier T., Peczuh M. W., Ernst B. (2018). Chem. Sci..

[cit411] Ofek I., Mirelman D., Sharon N. (1977). Nature.

[cit412] Aronson M., Medalia O., Schori L., Mirelman D., Sharon N., Ofek I. (1979). J. Infect. Dis..

[cit413] Firon N., Ofek I., Sharon N. (1983). Carbohyd. Res..

[cit414] Firon N., Ashkenazi S., Mirelman D., Ofek I., Sharon N. (1987). Infect. Immun..

[cit415] Han Z., Pinkner J. S., Ford B., Chorell E., Crowley J. M., Cusumano C. K., Campbell S., Henderson J. P., Hultgren S. J., Janetka J. W. (2012). J. Med. Chem..

[cit416] Jarvis C., Han Z., Kalas V., Klein R., Pinkner J. S., Ford B., Binkley J., Cusumano C. K., Cusumano Z., Mydock-McGrane L., Hultgren S. J., Janetka J. W. (2016). ChemMedChem.

[cit417] Cusumano C. K., Pinkner J. S., Han Z., Greene S. E., Ford B. A., Crowley J. R., Henderson J. P., Janetka J. W., Hultgren S. J. (2011). Sci. Transl. Med..

[cit418] Totsika M., Kostakioti M., Hannan T. J., Upton M., Beatson S. A., Janetka J. W., Hultgren S. J., Schembri M. A. (2013). J. Infect. Dis.

[cit419] Kleeb S., Pang L., Mayer K., Eris D., Sigl A., Preston R. C., Zihlmann P., Sharpe T., Jakob R. P., Abgottspon D., Hutter A. S., Scharenberg M., Jiang X., Navarra G., Rabbani S., Smiesko M., Lüdin N., Bezençon J., Schwardt O., Maier T., Ernst B. (2015). J. Med. Chem..

[cit420] Schönemann W., Cramer J., Mühlethaler T., Fiege B., Silbermann M., Rabbani S., Dätwyler P., Zihlmann P., Jakob R. P., Sager C. P., Smieško M., Schwardt O., Maier T., Ernst B. (2019). ChemMedChem.

[cit421] Schwardt O., Rabbani S., Hartmann M., Abgottspon D., Wittwer M., Kleeb S., Zalewski A., Smieško M., Cutting B., Ernst B. (2011). Bioorg. Med. Chem..

[cit422] Jiang X., Abgottspon D., Kleeb S., Rabbani S., Scharenberg M., Wittwer M., Haug M., Schwardt O., Ernst B. (2012). J. Med. Chem..

[cit423] Scharenberg M., Schwardt O., Rabbani S., Ernst B. (2012). J. Med. Chem..

[cit424] Mydock-McGrane L., Cusumano Z., Han Z., Binkley J., Kostakioti M., Hannan T., Pinkner J. S., Klein R., Kalas V., Crowley J., Rath N. P., Hultgren S. J., Janetka J. W. (2016). J. Med. Chem..

[cit425] Kleeb S., Jiang X., Frei P., Sigl A., Bezençon J., Bamberger K., Schwardt O., Ernst B. (2016). J. Med. Chem..

[cit426] Sattigeri J. A., Garg M., Bhateja P., Soni A., Rauf A. R. A., Gupta M., Deshmukh M. S., Jain T., Alekar N., Barman T. K., Jha P., Chaira T., Bambal R. B., Upadhyay D. J., Nishi T. (2018). Bioorg. Med. Chem. Lett..

[cit427] Mousavifar L., Touaibia M., Roy R. (2018). Acc. Chem. Res..

[cit428] Brument S., Sivignon A., Dumych T. I., Moreau N., Roos G., Guérardel Y., Chalopin T., Deniaud D., Bilyy R. O., Darfeuille-Michaud A., Bouckaert J., Gouin S. G. (2013). J. Med. Chem..

[cit429] Chalopin T., Alvarez Dorta D., Sivignon A., Caudan M., Dumych T. I., Bilyy R. O., Deniaud D., Barnich N., Bouckaert J., Gouin S. G. (2016). Org. Biomol. Chem..

[cit430] Sivignon A., Yan X., Alvarez Dorta D., Bonnet R., Bouckaert J., Fleury E., Bernard J., Gouin S. G., Darfeuille-Michaud A., Barnich N. (2015). mBio.

[cit431] Alvarez Dorta D., Sivignon A., Chalopin T., Dumych T. I., Roos G., Bilyy R. O., Deniaud D., Krammer E.-M., de Ruyck J., Lensink M. F., Bouckaert J., Barnich N., Gouin S. G. (2016). ChemBioChem.

[cit432] Bennani Y. L. and Liu B., WO2014055474, 2014

[cit433] Bennani Y. L. , Cadilhac C., Das S. K., Dietrich E., Gallant J., Liu B., Pereira O. Z., Ramtohul Y. K., Reddy T. J., Vaillancourt L., Yannopoulos C. and Vallee F., WO2013134415, 2013

[cit434] Dietrich E. , Poisson C., Gallant M., Lessard S., Liu B., Das S. K., Ramtohul Y., Reddy T. J., Martel J., Vallee F. and Levesque J.-F., WO2014165107, 2014

[cit435] Ramtohul Y. , Das P. K., Cadilhac C., Reddy T. J., Gallant M., Liu B., Dietrich E., Vallee F., Martel J. and Poisson C., WO2014100158A1, 2014

[cit436] Gallant M. , Truchon J.-F., Reddy T. J., Dietrich E., Vaillancourt L. and Vallee F., WO2016199105, 2016

[cit437] Klein R. D., Hultgren S. J. (2020). Nat. Rev. Microbiol..

[cit438] Poole K. (2011). Fron. Microbiol..

[cit439] Wagner S., Sommer R., Hinsberger S., Lu C., Hartmann R. W., Empting M., Titz A. (2016). J. Med. Chem..

[cit440] Chemani C., Imberty A., de Bentzmann S., Pierre M., Wimmerová M., Guery B. P., Faure K. (2009). Infect. Immun..

[cit441] Tielker D., Hacker S., Loris R., Strathmann M., Wingender J., Wilhelm S., Rosenau F., Jaeger K. E. (2005). Microbiology.

[cit442] Diggle S. P., Stacey R. E., Dodd C., Cámara M., Williams P., Winzer K. (2006). Environ. Microbiol..

[cit443] Gilboa-Garber N. , Methods in Enzymology, Academic Press, 1982, vol. 83, pp. 378–385 10.1016/0076-6879(82)83034-6 6808301

[cit444] Adam E. C., Mitchell B. S., Schumacher D. U., Grant G., Schumacher U. (1997). Am. J. Respir. Crit. Care Med..

[cit445] Bajolet-Laudinat O., Girod-de Bentzmann S., Tournier J. M., Madoulet C., Plotkowski M. C., Chippaux C., Puchelle E. (1994). Infect. Immun..

[cit446] Zahorska E., Rosato F., Stober K., Kuhaudomlarp S., Meiers J., Hauck D., Reith D., Gillon E., Rox K., Imberty A., Römer W., Titz A. (2023). Angew. Chem., Int. Ed..

[cit447] Thuenauer R., Landi A., Trefzer A., Altmann S., Wehrum S., Eierhoff T., Diedrich B., Dengjel J., Nyström A., Imberty A., Römer W. (2020). mBio.

[cit448] Zheng S., Eierhoff T., Aigal S., Brandel A., Thuenauer R., de Bentzmann S., Imberty A., Römer W. (2017). Biochim. Biophys. Acta, Mol. Cell Res..

[cit449] Cott C., Thuenauer R., Landi A., Kühn K., Juillot S., Imberty A., Madl J., Eierhoff T., Römer W. (2016). Biochim. Biophys. Acta.

[cit450] Wilhelm I., Levit-Zerdoun E., Jakob J., Villringer S., Frensch M., Übelhart R., Landi A., Müller P., Imberty A., Thuenauer R., Claudinon J., Jumaa H., Reth M., Eibel H., Hobeika E., Römer W. (2019). Sci. Signal..

[cit451] Sponsel J., Guo Y., Hamzam L., Lavanant A. C., Pérez-Riverón A., Partiot E., Muller Q., Rottura J., Gaudin R., Hauck D., Titz A., Flacher V., Römer W., Mueller C. G. (2023). EMBO Rep..

[cit452] Eierhoff T., Bastian B., Thuenauer R., Madl J., Audfray A., Aigal S., Juillot S., Rydell G. E., Müller S., de Bentzmann S., Imberty A., Fleck C., Römer W. (2014). Proc. Natl. Acad. Sci. U. S. A..

[cit453] von Bismarck P., Schneppenheim R., Schumacher U. (2001). Klin. Padiatr..

[cit454] Hauber H. P., Schulz M., Pforte A., Mack D., Zabel P., Schumacher U. (2008). Int. J. Med. Sci..

[cit455] Bucior I., Abbott J., Song Y., Matthay M. A., Engel J. N. (2013). Am. J. Physiol.: Lung Cell. Mol. Physiol..

[cit456] Blanchard B., Nurisso A., Hollville E., Tétaud C., Wiels J., Pokorná M., Wimmerová M., Varrot A., Imberty A. (2008). J. Mol. Biol..

[cit457] Cioci G., Mitchell E. P., Gautier C., Wimmerová M., Sudakevitz D., Pérez S., Gilboa-Garber N., Imberty A. (2003). FEBS Lett..

[cit458] Garber N., Guempel U., Belz A., Gilboa-Garber N., Doyle R. J. (1992). Biochim. Biophys. Acta.

[cit459] Kadam R. U., Garg D., Schwartz J., Visini R., Sattler M., Stocker A., Darbre T., Reymond J. L. (2013). ACS Chem. Biol..

[cit460] Rodrigue J., Ganne G., Blanchard B., Saucier C., Giguère D., Shiao T. C., Varrot A., Imberty A., Roy R. (2013). Org. Biomol. Chem..

[cit461] Joachim I., Rikker S., Hauck D., Ponader D., Boden S., Sommer R., Hartmann L., Titz A. (2016). Org. Biomol. Chem..

[cit462] Siebs E., Shanina E., Kuhaudomlarp S., da Silva Figueiredo Celestino Gomes P., Fortin C., Seeberger P. H., Rognan D., Rademacher C., Imberty A., Titz A. (2022). ChemBioChem.

[cit463] Novoa A., Eierhoff T., Topin J., Varrot A., Barluenga S., Imberty A., Römer W., Winssinger N. (2014). Angew. Chem., Int. Ed..

[cit464] Chabre Y. M., Giguère D., Blanchard B., Rodrigue J., Rocheleau S., Neault M., Rauthu S., Papadopoulos A., Arnold A. A., Imberty A., Roy R. (2011). Chemistry.

[cit465] Denavit V., Lainé D., Bouzriba C., Shanina E., Gillon É., Fortin S., Rademacher C., Imberty A., Giguère D. (2019). Chemistry.

[cit466] Shanina E., Siebs E., Zhang H., Varón Silva D., Joachim I., Titz A., Rademacher C. (2021). Glycobiology.

[cit467] Scharenberg M., Jiang X., Pang L., Navarra G., Rabbani S., Binder F., Schwardt O., Ernst B. (2014). ChemMedChem.

[cit468] Copeland R. A., Pompliano D. L., Meek T. D. (2006). Nat. Rev. Drug Discov..

[cit469] Sommer R., Hauck D., Varrot A., Wagner S., Audfray A., Prestel A., Möller H. M., Imberty A., Titz A. (2015). ChemistryOpen.

[cit470] Sommer R., Wagner S., Rox K., Varrot A., Hauck D., Wamhoff E. C., Schreiber J., Ryckmans T., Brunner T., Rademacher C., Hartmann R. W., Brönstrup M., Imberty A., Titz A. (2018). J. Am. Chem. Soc..

[cit471] Zahorska E., Kuhaudomlarp S., Minervini S., Yousaf S., Lepsik M., Kinsinger T., Hirsch A. K. H., Imberty A., Titz A. (2020). Chem. Commun..

[cit472] Bauer R. A. (2015). Drug Discov. Today.

[cit473] Wagner S., Hauck D., Hoffmann M., Sommer R., Joachim I., Müller R., Imberty A., Varrot A., Titz A. (2017). Angew. Chem., Int. Ed..

[cit474] Reymond J. L., Bergmann M., Darbre T. (2013). Chem. Soc. Rev..

[cit475] Kadam R. U., Bergmann M., Hurley M., Garg D., Cacciarini M., Swiderska M. A., Nativi C., Sattler M., Smyth A. R., Williams P., Cámara M., Stocker A., Darbre T., Reymond J. L. (2011). Angew. Chem., Int. Ed..

[cit476] Boukerb A. M., Rousset A., Galanos N., Méar J. B., Thépaut M., Grandjean T., Gillon E., Cecioni S., Abderrahmen C., Faure K., Redelberger D., Kipnis E., Dessein R., Havet S., Darblade B., Matthews S. E., de Bentzmann S., Guéry B., Cournoyer B., Imberty A., Vidal S. (2014). J. Med. Chem..

[cit477] Pertici F., Pieters R. J. (2012). Chem. Commun..

[cit478] Pertici F., de Mol N. J., Kemmink J., Pieters R. J. (2013). Chemistry.

[cit479] Visini R., Jin X., Bergmann M., Michaud G., Pertici F., Fu O., Pukin A., Branson T. R., Thies-Weesie D. M., Kemmink J., Gillon E., Imberty A., Stocker A., Darbre T., Pieters R. J., Reymond J. L. (2015). ACS Chem. Biol..

[cit480] Yu G., Vicini A. C., Pieters R. J. (2019). J. Org. Chem..

[cit481] Imberty A., Wimmerová M., Mitchell E. P., Gilboa-Garber N. (2004). Microbes Infect..

[cit482] Perret S., Sabin C., Dumon C., Pokorná M., Gautier C., Galanina O., Ilia S., Bovin N., Nicaise M., Desmadril M., Gilboa-Garber N., Wimmerová M., Mitchell E. P., Imberty A. (2005). Biochem. J..

[cit483] Mitchell E., Houles C., Sudakevitz D., Wimmerova M., Gautier C., Pérez S., Wu A. M., Gilboa-Garber N., Imberty A. (2002). Nat. Struct. Biol..

[cit484] Mitchell E. P., Sabin C., Snajdrová L., Pokorná M., Perret S., Gautier C., Hofr C., Gilboa-Garber N., Koca J., Wimmerová M., Imberty A. (2005). Proteins.

[cit485] Gajdos L., Blakeley M. P., Haertlein M., Forsyth V. T., Devos J. M., Imberty A. (2022). Nat. Commun..

[cit486] Klockgether J., Cramer N., Wiehlmann L., Davenport C. F., Tümmler B. (2011). Front. Microbiol..

[cit487] Sommer R., Wagner S., Varrot A., Nycholat C. M., Khaledi A., Häussler S., Paulson J. C., Imberty A., Titz A. (2016). Chem. Sci..

[cit488] Boukerb A. M., Decor A., Ribun S., Tabaroni R., Rousset A., Commin L., Buff S., Doléans-Jordheim A., Vidal S., Varrot A., Imberty A., Cournoyer B. (2016). Front. Microbiol..

[cit489] Meiers J., Siebs E., Zahorska E., Titz A. (2019). Curr. Opin. Chem. Biol..

[cit490] Marotte K., Sabin C., Préville C., Moumé-Pymbock M., Wimmerová M., Mitchell E. P., Imberty A., Roy R. (2007). ChemMedChem.

[cit491] Andreini M., Anderluh M., Audfray A., Bernardi A., Imberty A. (2010). Carbohydr. Res..

[cit492] Johansson E. M., Crusz S. A., Kolomiets E., Buts L., Kadam R. U., Cacciarini M., Bartels K. M., Diggle S. P., Cámara M., Williams P., Loris R., Nativi C., Rosenau F., Jaeger K. E., Darbre T., Reymond J. L. (2008). Chem. Biol..

[cit493] Hauck D., Joachim I., Frommeyer B., Varrot A., Philipp B., Möller H. M., Imberty A., Exner T. E., Titz A. (2013). ACS Chem. Biol..

[cit494] Loris R., Tielker D., Jaeger K. E., Wyns L. (2003). J. Mol. Biol..

[cit495] Hofmann A., Sommer R., Hauck D., Stifel J., Göttker-Schnetmann I., Titz A. (2015). Carbohydr. Res..

[cit496] Sommer R., Exner T. E., Titz A. (2014). PLoS One.

[cit497] Sommer R., Rox K., Wagner S., Hauck D., Henrikus S. S., Newsad S., Arnold T., Ryckmans T., Brönstrup M., Imberty A., Varrot A., Hartmann R. W., Titz A. (2019). J. Med. Chem..

[cit498] Mała P., Siebs E., Meiers J., Rox K., Varrot A., Imberty A., Titz A. (2022). J. Med. Chem..

[cit499] Michaud G., Visini R., Bergmann M., Salerno G., Bosco R., Gillon E., Richichi B., Nativi C., Imberty A., Stocker A., Darbre T., Reymond J. L. (2016). Chem. Sci..

[cit500] Mahenthiralingam E., Baldwin A., Dowson C. G. (2008). J. Appl. Microbiol..

[cit501] Audfray A., Claudinon J., Abounit S., Ruvoën-Clouet N., Larson G., Smith D. F., Wimmerová M., Le Pendu J., Römer W., Varrot A., Imberty A. (2012). J. Biol. Chem..

[cit502] Richichi B., Imberty A., Gillon E., Bosco R., Sutkeviciute I., Fieschi F., Nativi C. (2013). Org. Biomol. Chem..

[cit503] Kuhaudomlarp S., Cerofolini L., Santarsia S., Gillon E., Fallarini S., Lombardi G., Denis M., Giuntini S., Valori C., Fragai M., Imberty A., Dondoni A., Nativi C. (2020). Chem. Sci..

[cit504] Dingjan T., Gillon É., Imberty A., Pérez S., Titz A., Ramsland P. A., Yuriev E. (2018). J. Chem. Inf. Model..

[cit505] Inhülsen S., Aguilar C., Schmid N., Suppiger A., Riedel K., Eberl L. (2012). MicrobiologyOpen.

[cit506] Lameignere E., Malinovská L., Sláviková M., Duchaud E., Mitchell E. P., Varrot A., Sedo O., Imberty A., Wimmerová M. (2008). Biochem. J..

[cit507] Lameignere E., Shiao T. C., Roy R., Wimmerova M., Dubreuil F., Varrot A., Imberty A. (2010). Glycobiology.

[cit508] Marchetti R., Malinovska L., Lameignère E., Adamova L., de Castro C., Cioci G., Stanetty C., Kosma P., Molinaro A., Wimmerova M., Imberty A., Silipo A. (2012). Glycobiology.

[cit509] Beshr G., Sommer R., Hauck D., Siebert D. C. B., Hofmann A., Imberty A., Titz A. (2016). MedChemComm.

[cit510] Sulák O., Cioci G., Delia M., Lahmann M., Varrot A., Imberty A., Wimmerová M. (2010). Structure.

[cit511] Sulák O., Cioci G., Lameignère E., Balloy V., Round A., Gutsche I., Malinovská L., Chignard M., Kosma P., Aubert D. F., Marolda C. L., Valvano M. A., Wimmerová M., Imberty A. (2011). PLoS Pathog..

[cit512] Lal K., Bermeo R., Cramer J., Vasile F., Ernst B., Imberty A., Bernardi A., Varrot A., Belvisi L. (2021). Chemistry.

[cit513] Bermeo R., Lal K., Ruggeri D., Lanaro D., Mazzotta S., Vasile F., Imberty A., Belvisi L., Varrot A., Bernardi A. (2022). ACS Chem. Biol..

[cit514] Thompson A. J., de Vries R. P., Paulson J. C. (2019). Curr. Opin. Virol..

[cit515] Long J. S., Mistry B., Haslam S. M., Barclay W. S. (2019). Nat. Rev. Microbiol..

[cit516] Blaum B. S. and Stehle T., in Advances in Carbohydrate Chemistry and Biochemistry, ed. D. C. Baker, Academic Press, 2019, vol. 76, pp. 65–111 10.1016/bs.accb.2018.09.004 30851744

[cit517] Stencel-Baerenwald J. E., Reiss K., Reiter D. M., Stehle T., Dermody T. S. (2014). Nat. Rev. Microbiol..

[cit518] Papp I., Sieben C., Ludwig K., Roskamp M., Böttcher C., Schlecht S., Herrmann A., Haag R. (2010). Small.

[cit519] Lu W., Du W., Somovilla V. J., Yu G., Haksar D., de Vries E., Boons G.-J., de Vries R. P., de Haan C. A. M., Pieters R. J. (2019). J. Med. Chem..

[cit520] Nie C., Stadtmüller M., Parshad B., Wallert M., Ahmadi V., Kerkhoff Y., Bhatia S., Block S., Cheng C., Wolff T. (2021). Sci. Adv..

[cit521] Sauter N. K., Hanson J. E., Glick G. D., Brown J. H., Crowther R. L., Park S. J., Skehel J. J., Wiley D. C. (1992). Biochemistry.

[cit522] Herrler G., Gross H. J., Imhof A., Brossmer R., Milks G., Paulson J. C. (1992). J. Biol. Chem..

[cit523] Brossmer R., Isecke R., Herrler G. (1993). FEBS Lett..

[cit524] Schuster M. C., Mann D. A., Buchholz T. J., Johnson K. M., Thomas W. D., Kiessling L. L. (2003). Org. Lett..

[cit525] Garber K. C. A., Wangkanont K., Carlson E. E., Kiessling L. L. (2010). Chem. Commun..

[cit526] Grim J. C., Garber K. C. A., Kiessling L. L. (2011). Org. Lett..

[cit527] Prost L. R., Grim J. C., Tonelli M., Kiessling L. L. (2012). ACS Chem. Biol..

[cit528] Kranich R., Busemann A. S., Bock D., Schroeter-Maas S., Beyer D., Heinemann B., Meyer M., Schierhorn K., Zahlten R., Wolff G., Aydt E. M. (2007). J. Med. Chem..

[cit529] Baell J. B., Nissink J. W. M. (2018). ACS Chem. Biol..

[cit530] Baell J. B., Holloway G. A. (2010). J. Med. Chem..

[cit531] Hopkins A. L., Groom C. R., Alex A. (2004). Drug Discov. Today.

[cit532] Shanina E., Kuhaudomlarp S., Siebs E., Fuchsberger F. F., Denis M., da Silva Figueiredo Celestino Gomes P., Clausen M. H., Seeberger P. H., Rognan D., Titz A., Imberty A., Rademacher C. (2022). Commun. Chem..

[cit533] Milne J. C., Lambert P. D., Schenk S., Carney D. P., Smith J. J., Gagne D. J., Jin L., Boss O., Perni R. B., Vu C. B., Bemis J. E., Xie R., Disch J. S., Ng P. Y., Nunes J. J., Lynch A. V., Yang H., Galonek H., Israelian K., Choy W., Iffland A., Lavu S., Medvedik O., Sinclair D. A., Olefsky J. M., Jirousek M. R., Elliott P. J., Westphal C. H. (2007). Nature.

[cit534] Generoso S. F., Giustiniano M., La Regina G., Bottone S., Passacantilli S., Di Maro S., Cassese H., Bruno A., Mallardo M., Dentice M., Silvestri R., Marinelli L., Sarnataro D., Bonatti S., Novellino E., Stornaiuolo M. (2015). Nat. Chem. Biol..

[cit535] Bagal S. K., Omoto K., Blakemore D. C., Bungay P. J., Bilsland J. G., Clarke P. J., Corbett M. S., Cronin C. N., Cui J. J., Dias R., Flanagan N. J., Greasley S. E., Grimley R., Johnson E., Fengas D., Kitching L., Kraus M. L., McAlpine I., Nagata A., Waldron G. J., Warmus J. S. (2019). J. Med. Chem..

[cit536] Nussinov R., Tsai C. J. (2013). Cell.

[cit537] Lu X., Smaill J. B., Ding K. (2020). Angew. Chem., Int. Ed..

[cit538] Lu S., Shen Q., Zhang J. (2019). Acc. Chem. Res..

[cit539] Kalas V., Pinkner J. S., Hannan T. J., Hibbing M. E., Dodson K. W., Holehouse A. S., Zhang H., Tolia N. H., Gross M. L., Pappu R. V., Janetka J., Hultgren S. J. (2017). Sci. Adv..

[cit540] Kisiela D. I., Magala P., Interlandi G., Carlucci L. A., Ramos A., Tchesnokova V., Basanta B., Yarov-Yarovoy V., Avagyan H., Hovhannisyan A., Thomas W. E., Stenkamp R. E., Klevit R. E., Sokurenko E. V. (2021). PLoS Pathog..

[cit541] Rodriguez V. B., Kidd B. A., Interlandi G., Tchesnokova V., Sokurenko E. V., Thomas W. E. (2013). J. Biol. Chem..

[cit542] Tchesnokova V., Aprikian P., Yakovenko O., Larock C., Kidd B., Vogel V., Thomas W., Sokurenko E. (2008). J. Biol. Chem..

[cit543] Tchesnokova V., Aprikian P., Kisiela D., Gowey S., Korotkova N., Thomas W., Sokurenko E. (2011). Infect. Immun..

[cit544] Rabbani S., Fiege B., Eris D., Silbermann M., Jakob R. P., Navarra G., Maier T., Ernst B. (2018). J. Biol. Chem..

[cit545] Krammer E. M., de Ruyck J., Roos G., Bouckaert J., Lensink M. F. (2018). Molecules.

[cit546] Sauer M. M., Jakob R. P., Eras J., Baday S., Eriş D., Navarra G., Bernèche S., Ernst B., Maier T., Glockshuber R. (2016). Nat. Commun..

[cit547] Heim J. B., Hodnik V., Heggelund J. E., Anderluh G., Krengel U. (2019). Sci. Rep..

[cit548] Shanina E., Kuhaudomlarp S., Lal K., Seeberger P. H., Imberty A., Rademacher C. (2022). Angew. Chem., Int. Ed..

[cit549] Dings R. P., Miller M. C., Nesmelova I., Astorgues-Xerri L., Kumar N., Serova M., Chen X., Raymond E., Hoye T. R., Mayo K. H. (2012). J. Med. Chem..

[cit550] Shih T. C., Liu R., Fung G., Bhardwaj G., Ghosh P. M., Lam K. S. (2017). Mol. Cancer Ther..

[cit551] Shih T. C., Fan Y., Kiss S., Li X., Deng X. N., Liu R., Chen X. J., Carney R., Chen A., Ghosh P. M., Lam K. S. (2019). Neuro Oncol..

[cit552] Kahsai A. W., Cui J., Kaniskan H. Ü., Garner P. P., Fenteany G. (2008). J. Biol. Chem..

[cit553] Pham N. T. H., Letourneau M., Fortier M., Begin G., Al-Abdul-Wahid M. S., Pucci F., Folch B., Rooman M., Chatenet D., St-Pierre Y., Lague P., Calmettes C., Doucet N. (2021). J. Biol. Chem..

[cit554] Kahsai A. W., Zhu S., Wardrop D. J., Lane W. S., Fenteany G. (2006). Chem. Biol..

[cit555] Onizuka T., Shimizu H., Moriwaki Y., Nakano T., Kanai S., Shimada I., Takahashi H. (2012). FEBS J..

[cit556] Wragg S., Drickamer K. (1999). J. Biol. Chem..

[cit557] Feinberg H., Torgersen D., Drickamer K., Weis W. I. (2000). J. Biol. Chem..

[cit558] Gramberg T., Soilleux E., Fisch T., Lalor P. F., Hofmann H., Wheeldon S., Cotterill A., Wegele A., Winkler T., Adams D. H., Pohlmann S. (2008). Virology.

[cit559] Probert F., Mitchell D. A., Dixon A. M. (2014). FEBS J..

[cit560] Ng K. K., Weis W. I. (1998). Biochemistry.

[cit561] Poget S. F., Freund S. M., Howard M. J., Bycroft M. (2001). Biochemistry.

[cit562] Nielbo S., Thomsen J. K., Graversen J. H., Jensen P. H., Etzerodt M., Poulsen F. M., Thogersen H. C. (2004). Biochemistry.

[cit563] Marcelo F., Supekar N., Corzana F., van der Horst J. C., Vuist I. M., Live D., Boons G. P. H., Smith D. F., van Vliet S. J. (2019). J. Biol. Chem..

[cit564] Jegouzo S. A., Feinberg H., Dungarwalla T., Drickamer K., Weis W. I., Taylor M. E. (2015). J. Biol. Chem..

[cit565] Nagae M., Yamanaka K., Hanashima S., Ikeda A., Morita-Matsumoto K., Satoh T., Matsumoto N., Yamamoto K., Yamaguchi Y. (2013). J. Biol. Chem..

[cit566] Hardy J. A., Wells J. A. (2004). Curr. Opin. Struct. Biol..

[cit567] Lu S., Ji M., Ni D., Zhang J. (2018). Drug Discov. Today.

[cit568] Aretz J., Wamhoff E. C., Hanske J., Heymann D., Rademacher C. (2014). Front. Immunol..

[cit569] Keller B. G., Rademacher C. (2020). Curr. Opin. Struct. Biol..

[cit570] Finger E. B., Puri K. D., Alon R., Lawrence M. B., von Andrian U. H., Springer T. A. (1996). Nature.

[cit571] Lawrence M. B., Kansas G. S., Kunkel E. J., Ley K. (1997). J. Cell Biol..

[cit572] Waldron T. T., Springer T. A. (2009). Proc. Natl. Acad. Sci. U. S. A..

[cit573] Marshall B. T., Long M., Piper J. W., Yago T., McEver R. P., Zhu C. (2003). Nature.

[cit574] Sarangapani K. K., Yago T., Klopocki A. G., Lawrence M. B., Fieger C. B., Rosen S. D., McEver R. P., Zhu C. (2004). J. Biol. Chem..

[cit575] Lu S., Zhang Y., Long M. (2010). PLoS One.

[cit576] Lu S., Chen S., Mao D., Zhang Y., Long M. (2015). PLoS One.

[cit577] Erbe D. V., Wolitzky B. A., Presta L. G., Norton C. R., Ramos R. J., Burns D. K., Rumberger J. M., Rao B. N., Foxall C., Brandley B. K. (1992). et al.. J. Cell Biol..

[cit578] Aleisa F. A., Sakashita K., Lee J. M., AbuSamra D. B., Al Alwan B., Nozue S., Tehseen M., Hamdan S. M., Habuchi S., Kusakabe T., Merzaban J. S. (2020). J. Biol. Chem..

[cit579] Wang X., Bie L., Fei J., Gao J. (2020). J. Chem. Inf. Model..

[cit580] Brandley B. K., Kiso M., Abbas S., Nikrad P., Srivasatava O., Foxall C., Oda Y., Hasegawa A. (1993). Glycobiology.

[cit581] Ramphal J. Y., Zheng Z. L., Perez C., Walker L. E., DeFrees S. A., Gaeta F. C. (1994). J. Med. Chem..

[cit582] Hanske J., Aleksić S., Ballaschk M., Jurk M., Shanina E., Beerbaum M., Schmieder P., Keller B. G., Rademacher C. (2016). J. Am. Chem. Soc..

[cit583] Joswig J. O., Anders J., Zhang H., Rademacher C., Keller B. G. (2021). J. Biol. Chem..

[cit584] Zhang H., Modenutti C., Nekkanti Y. P. K., Denis M., Bermejo I. A., Lefèbre J., Che K., Kim D., Kagelmacher M., Kurzbach D., Nazaré M., Rademacher C. (2022). ACS Chem. Biol..

[cit585] Chatwell L., Holla A., Kaufer B. B., Skerra A. (2008). Mol. Immunol..

[cit586] Oda T., Yanagisawa H., Shinmori H., Ogawa Y., Kawamura T. (2022). eLife.

[cit587] Borrok M. J., Kiessling L. L. (2007). J. Am. Chem. Soc..

[cit588] Mangold S. L., Prost L. R., Kiessling L. L. (2012). Chem. Sci..

[cit589] Troelsen N. S., Shanina E., Gonzalez-Romero D., Dankova D., Jensen I. S. A., Sniady K. J., Nami F., Zhang H., Rademacher C., Cuenda A., Gotfredsen C. H., Clausen M. H. (2020). Angew. Chem., Int. Ed..

[cit590] Schulze J., Baukmann H., Wawrzinek R., Fuchsberger F. F., Specker E., Aretz J., Nazare M., Rademacher C. (2018). ACS Chem. Biol..

[cit591] Zhang H., Danek O., Makarov D., Radl S., Kim D., Ledvinka J., Vychodilova K., Hlavac J., Lefebre J., Denis M., Rademacher C., Menova P. (2022). ACS Med. Chem. Lett..

[cit592] Prescher H., Schweizer A., Frank M., Kuhfeldt E., Ring J., Nitschke L. (2022). J. Med. Chem..

[cit593] Schnapp G., Neubauer H., Büttner F. H., Handschuh S., Lingard I., Heilker R., Klinder K., Prestle J., Walter R., Wolff M., Zeeb M., Debaene F., Nar H., Fiegen D. (2020). Commun. Chem..

[cit594] Yamakawa N., Yasuda Y., Yoshimura A., Goshima A., Crocker P. R., Vergoten G., Nishiura Y., Takahashi T., Hanashima S., Matsumoto K., Yamaguchi Y., Tanaka H., Kitajima K., Sato C. (2020). Sci. Rep..

[cit595] van Dongen M. J. P., Kadam R. U., Juraszek J., Lawson E., Brandenburg B., Schmitz F., Schepens W. B. G., Stoops B., van Diepen H. A., Jongeneelen M., Tang C., Vermond J., van Eijgen-Obregoso Real A., Blokland S., Garg D., Yu W., Goutier W., Lanckacker E., Klap J. M., Peeters D. C. G., Wu J., Buyck C., Jonckers T. H. M., Roymans D., Roevens P., Vogels R., Koudstaal W., Friesen R. H. E., Raboisson P., Dhanak D., Goudsmit J., Wilson I. A. (2019). Science.

[cit596] Tang G., Lin X., Qiu Z., Li W., Zhu L., Wang L., Li S., Li H., Lin W., Yang M., Guo T., Chen L., Lee D., Wu J. Z., Yang W. (2011). ACS Med. Chem. Lett..

[cit597] White K. M., De Jesus P., Chen Z., Abreu, Jr. P., Barile E., Mak P. A., Anderson P., Nguyen Q. T., Inoue A., Stertz S., Koenig R., Pellecchia M., Palese P., Kuhen K., García-Sastre A., Chanda S. K., Shaw M. L. (2015). ACS Infect. Dis..

[cit598] Yao Y., Kadam R. U., Lee C. D., Woehl J. L., Wu N. C., Zhu X., Kitamura S., Wilson I. A., Wolan D. W. (2020). Proc. Natl. Acad. Sci. U. S. A..

[cit599] Antanasijevic A., Durst M. A., Cheng H., Gaisina I. N., Perez J. T., Manicassamy B., Rong L., Lavie A., Caffrey M. (2020). Life Sci. Alliance.

[cit600] Liu H. Y., Yang P. L. (2021). Annu. Rev. Virol..

[cit601] Civera M., Moroni E., Sorrentino L., Vasile F., Sattin S. (2021). Eur. J. Org. Chem..

[cit602] P. K. Grewal , in Methods in Enzymology, ed. M. Fukuda, Academic Press, 2010, vol. 479, pp. 223–241

[cit603] Henis Y. I., Katzir Z., Shia M. A., Lodish H. F. (1990). J. Cell Biol..

[cit604] Bischoff J., Lodish H. F. (1987). J. Biol. Chem..

[cit605] Spiess M. (1990). Biochemistry.

[cit606] Schwartz A. L., Fridovich S. E., Lodish H. F. (1982). J. Biol. Chem..

[cit607] Weigel P. H., Oka J. A. (1983). J. Biol. Chem..

[cit608] Ivanenkov Y. A., Maklakova S. Y., Beloglazkina E. K., Zyk N. V., Nazarenko A. G., Tonevitsky A. G., Kotelianski V. E. M., Majouga A. G. (2017). Russ. Chem. Rev..

[cit609] Large D. E., Soucy J. R., Hebert J., Auguste D. T. (2019). Adv. Ther..

[cit610] Dutta R., Mahato R. I. (2017). Pharmacol. Therapeut..

[cit611] Singh L., Indermun S., Govender M., Kumar P., Du Toit L. C., Choonara Y. E., Pillay V. (2018). Viruses.

[cit612] Elberry M. H., Darwish N. H. E., Mousa S. A. (2017). Virol. J..

[cit613] Borrmann S., Matuschewski K. (2011). Trends Mol. Med..

[cit614] Mamidyala S. K., Dutta S., Chrunyk B. A., Préville C., Wang H., Withka J. M., McColl A., Subashi T. A., Hawrylik S. J., Griffor M. C., Kim S., Pfefferkorn J. A., Price D. A., Menhaji-Klotz E., Mascitti V., Finn M. G. (2012). J. Am. Chem. Soc..

[cit615] Wu J., Nantz M. H., Zern M. A. (2002). Front. Biosci..

[cit616] Ruiz N. I., Drickamer K. (1996). Glycobiology.

[cit617] Connolly D. T., Townsend R. R., Kawaguchi K., Bell W. R., Lee Y. C. (1982). J. Biol. Chem..

[cit618] Kolatkar A. R., Leung A. K., Isecke R., Brossmer R., Drickamer K., Weis W. I. (1998). J. Biol. Chem..

[cit619] Lee Y. C., Townsend R. R., Hardy M. R., Lönngren J., Arnarp J., Haraldsson M., Lönn H. (1983). J. Biol. Chem..

[cit620] Westerlind U., Westman J., Törnquist E., Smith C. I. E., Oscarson S., Lahmann M., Norberg T. (2004). Glycoconj. J..

[cit621] Biessen E. A. L., Beuting D. M., Roelen H. C. P. F., van de Marel G. A., Van Boom J. H., Van Berkel T. J. C. (1995). J. Med. Chem..

[cit622] Huang X., Leroux J.-C., Castagner B. (2017). Bioconjugate Chem..

[cit623] Warrier D. U., Dhanabalan A. K., Krishnasamy G., Kolge H., Ghormade V., Gupta C. R., Ambre P. K., Shinde U. A. (2022). Int. J. Biol. Macromol..

[cit624] Tanaka T., Fujishima Y., Hamano S., Kaneo Y. (2004). Eur. J. Pharm. Sci..

[cit625] Kaneo Y., Tanaka T., Nakano T., Yamaguchi Y. (2001). J. Controlled Release.

[cit626] Lee Y. C. (1992). FASEB J..

[cit627] Liras S. , Mascitti V. and Thuma B., US20160207953A1, 2016

[cit628] Sanhueza C. A., Baksh M. M., Thuma B., Roy M. D., Dutta S., Préville C., Chrunyk B. A., Beaumont K., Dullea R., Ammirati M., Liu S., Gebhard D., Finley J. E., Salatto C. T., King-Ahmad A., Stock I., Atkinson K., Reidich B., Lin W., Kumar R., Tu M., Menhaji-Klotz E., Price D. A., Liras S., Finn M. G., Mascitti V. (2017). J. Am. Chem. Soc..

[cit629] Liras S. , Mascitti V. and Thuma B., US9340553B2, 2016

[cit630] Meier M., Bider M. D., Malashkevich V. N., Spiess M., Burkhard P. (2000). J. Mol. Biol..

[cit631] Gonçalves M., Mignani S., Rodrigues J., Tomás H. (2020). J. Controlled Release.

[cit632] Hashida M., Nishikawa M., Yamashita F., Takakura Y. (2001). Adv. Drug Deliv. Rev..

[cit633] Akamatsu K., Imai M., Yamasaki Y., Nishikawa M., Takakura Y., Hashida M. (1998). J. Drug Target..

[cit634] Wang S., Cheng L., Yu F., Pan W., Zhang J. (2006). Int. J. Pharm..

[cit635] Hopewell J. W., Duncan R., Wilding D., Chakrabarti K. (2001). Hum. Exp. Toxicol..

[cit636] Julyan P. J., Seymour L. W., Ferry D. R., Daryani S., Boivin C. M., Doran J., David M., Anderson D., Christodoulou C., Young A. M., Hesslewood S., Kerr D. J. (1999). J. Controlled Release.

[cit637] Seymour L. W., Ferry D. R., Anderson D., Hesslewood S., Julyan P. J., Poyner R., Doran J., Young A. M., Burtles S., Kerr D. J. (2002). J. Clin. Oncol..

[cit638] Böttger R., Pauli G., Chao P.-H., Al Fayez N., Hohenwarter L., Li S.-D. (2020). Adv. Drug Delivery Rev..

[cit639] Craparo E. F., Triolo D., Pitarresi G., Giammona G., Cavallaro G. (2013). Biomacromolecules.

[cit640] Zou Y., Song Y., Yang W., Meng F., Liu H., Zhong Z. (2014). J. Controlled Release.

[cit641] Medina S. H., Tekumalla V., Chevliakov M. V., Shewach D. S., Ensminger W. D., El-Sayed M. E. H. (2011). Biomaterials.

[cit642] Hayashi Y., Higashi T., Motoyama K., Mori Y., Jono H., Ando Y., Arima H. (2013). J. Drug Target..

[cit643] Cui H., Zhu X., Li S., Wang P., Fang J. (2021). ACS Omega.

[cit644] Nair J. K., Willoughby J. L. S., Chan A., Charisse K., Alam M. R., Wang Q., Hoekstra M., Kandasamy P., Kel’in A. V., Milstein S., Taneja N., O’Shea J., Shaikh S., Zhang L., van der Sluis R. J., Jung M. E., Akinc A., Hutabarat R., Kuchimanchi S., Fitzgerald K., Zimmermann T., van Berkel T. J. C., Maier M. A., Rajeev K. G., Manoharan M. (2014). J. Am. Chem. Soc..

[cit645] Prakash T. P., Graham M. J., Yu J., Carty R., Low A., Chappell A., Schmidt K., Zhao C., Aghajan M., Murray H. F., Riney S., Booten S. L., Murray S. F., Gaus H., Crosby J., Lima W. F., Guo S., Monia B. P., Swayze E. E., Seth P. P. (2014). Nucleic Acids Res..

[cit646] Nair J. K., Attarwala H., Sehgal A., Wang Q., Aluri K., Zhang X., Gao M., Liu J., Indrakanti R., Schofield S., Kretschmer P., Brown C. R., Gupta S., Willoughby J. L. S., Boshar J. A., Jadhav V., Charisse K., Zimmermann T., Fitzgerald K., Manoharan M., Rajeev K. G., Akinc A., Hutabarat R., Maier M. A. (2017). Nucleic Acids Res..

[cit647] Hassler M. R., Turanov A. A., Alterman J. F., Haraszti R. A., Coles A. H., Osborn M. F., Echeverria D., Nikan M., Salomon W. E., Roux L., Godinho B., Davis S. M., Morrissey D. V., Zamore P. D., Karumanchi S. A., Moore M. J., Aronin N., Khvorova A. (2018). Nucleic Acids Res..

[cit648] Schlegel M. K., Foster D. J., Kel’in A. V., Zlatev I., Bisbe A., Jayaraman M., Lackey J. G., Rajeev K. G., Charissé K., Harp J., Pallan P. S., Maier M. A., Egli M., Manoharan M. (2017). J. Am. Chem. Soc..

[cit649] Janas M. M., Schlegel M. K., Harbison C. E., Yilmaz V. O., Jiang Y., Parmar R., Zlatev I., Castoreno A., Xu H., Shulga-Morskaya S., Rajeev K. G., Manoharan M., Keirstead N. D., Maier M. A., Jadhav V. (2018). Nat. Commun..

[cit650] Debacker A. J., Voutila J., Catley M., Blakey D., Habib N. (2020). Mol. Ther..

[cit651] Weingärtner A., Bethge L., Weiss L., Sternberger M., Lindholm M. W. (2020). Mol. Ther. Nucleic Acids.

[cit652] Gregoriadis G., Morell A. G., Sternlieb I., Scheinberg I. H. (1970). J. Biol. Chem..

[cit653] Zimmermann T. S., Karsten V., Chan A., Chiesa J., Boyce M., Bettencourt B. R., Hutabarat R., Nochur S., Vaishnaw A., Gollob J. (2017). Mol. Ther..

[cit654] Alnylam-Our pipeline, https://www.alnylam.com/alnylam-rnai-pipeline, (accessed September 2022, 2022)

[cit655] Dicerna Pipeline, https://dicerna.com/pipeline/, (accessed September 2022, 2022)

[cit656] Novo Nordisk R & D Pipeline, https://www.novonordisk.com/science-and-technology/r-d-pipeline.html, (accessed September 2022, 2022)

[cit657] Silence Therapeutics-Our pipeline, https://silence-therapeutics.com/our-pipeline/default.aspx, (accessed September 2022, 2022)

[cit658] Arbutus Biopharma-Pipeline, https://www.arbutusbio.com/pipeline/, (accessed September 2022, 2022)

[cit659] Balwani M., Sardh E., Ventura P., Peiró P. A., Rees D. C., Stölzel U., Bissell D. M., Bonkovsky H. L., Windyga J., Anderson K. E., Parker C., Silver S. M., Keel S. B., Wang J.-D., Stein P. E., Harper P., Vassiliou D., Wang B., Phillips J., Ivanova A., Langendonk J. G., Kauppinen R., Minder E., Horie Y., Penz C., Chen J., Liu S., Ko J. J., Sweetser M. T., Garg P., Vaishnaw A., Kim J. B., Simon A. R., Gouya L. (2020). New Engl. J. Med..

[cit660] Syed Y. Y. (2021). Drugs.

[cit661] Marchi R., Duarte L., Ke A., Hl B., Al E. (2020). Hematol. Transfus. Cell Ther..

[cit662] Garrelfs S. F., Frishberg Y., Hulton S. A., Koren M. J., O’Riordan W. D., Cochat P., Deschênes G., Shasha-Lavsky H., Saland J. M., van’t Hoff W. G., Fuster D. G., Magen D., Moochhala S. H., Schalk G., Simkova E., Groothoff J. W., Sas D. J., Meliambro K. A., Lu J., Sweetser M. T., Garg P. P., Vaishnaw A. K., Gansner J. M., McGregor T. L., Lieske J. C. (2021). New Engl. J. Med..

[cit663] Frishberg Y., Deschênes G., Groothoff J. W., Hulton S.-A., Magen D., Harambat J., G. van’t Hoff W., Lorch U., Milliner D. S., Lieske J. C., Haslett P., Garg P. P., Vaishnaw A. K., Talamudupula S., Lu J., Habtemariam B. A., Erbe D. V., McGregor T. L., Cochat P. (2021). Clin. J. Am. Soc. Nephrol..

[cit664] Erger F., Beck B. B. (2021). Nat. Rev. Nephrol..

[cit665] Massy Z. A., Drueke T. B. (2022). Kidney Int..

[cit666] Raal F. J., Kallend D., Ray K. K., Turner T., Koenig W., Wright R. S., Wijngaard P. L. J., Curcio D., Jaros M. J., Leiter L. A., Kastelein J. J. P. (2020). N. Engl. J. Med..

[cit667] Ray K. K., Wright R. S., Kallend D., Koenig W., Leiter L. A., Raal F. J., Bisch J. A., Richardson T., Jaros M., Wijngaard P. L. J., Kastelein J. J. P. (2020). N. Engl. J. Med..

[cit668] Lamb Y. N. (2021). Drugs.

[cit669] Habtemariam B. A., Karsten V., Attarwala H., Goel V., Melch M., Clausen V. A., Garg P., Vaishnaw A. K., Sweetser M. T., Robbie G. J., Vest J. (2021). Clin. Pharmacol. Ther..

[cit670] Bekes M., Langley D. R., Crews C. M. (2022). Nat. Rev. Drug Discov..

[cit671] Ding Y., Xing D., Fei Y., Lu B. (2022). Chem. Soc. Rev..

[cit672] Nalawansha D. A., Crews C. M. (2020). Cell Chem. Biol..

[cit673] Banik S. M., Pedram K., Wisnovsky S., Ahn G., Riley N. M., Bertozzi C. R. (2020). Nature.

[cit674] Zhang X., Liu H., He J., Ou C., Donahue T. C., Muthana M. M., Su L., Wang L. X. (2022). ACS Chem. Biol..

[cit675] Caianiello D. F., Zhang M., Ray J. D., Howell R. A., Swartzel J. C., Branham E. M. J., Chirkin E., Sabbasani V. R., Gong A. Z., McDonald D. M., Muthusamy V., Spiegel D. A. (2021). Nat. Chem. Biol..

[cit676] Ahn G., Banik S. M., Miller C. L., Riley N. M., Cochran J. R., Bertozzi C. R. (2021). Nat. Chem. Biol..

[cit677] Gary-Bobo M., Nirde P., Jeanjean A., Morere A., Garcia M. (2007). Curr. Med. Chem..

[cit678] Tong P. Y., Gregory W., Kornfeld S. (1989). J. Biol. Chem..

[cit679] Zhou Y., Teng P., Montgomery N. T., Li X., Tang W. (2021). ACS Cent. Sci..

[cit680] Caianiello D. F., Zhang M., Ray J. D., Howell R. A., Swartzel J. C., Branham E. M. J., Chirkin E., Sabbasani V. R., Gong A. Z., McDonald D. M., Muthusamy V., Spiegel D. A. (2021). Nat. Chem. Biol..

[cit681] Liras S. , Mascitti V., Thuma B. A. and Rouet R., WO2021155317A1, 2021

[cit682] Saulnier M. G. , Chen J. J., Karra S., Sprott K. T., Wiles J. A. and Ray S., WO2021155317, 2021

[cit683] Tozzo E. , Presented in part at the 9th Drug Discovery Strategic Summit (DDSS), 2022

[cit684] Olson L. J., Zhang J., Lee Y. C., Dahms N. M., Kim J. J. (1999). J. Biol. Chem..

[cit685] Duong B. H., Tian H., Ota T., Completo G., Han S., Vela J. L., Ota M., Kubitz M., Bovin N., Paulson J. C., Nemazee D. (2010). J. Exp. Med..

[cit686] Chen W. C., Completo G. C., Sigal D. S., Crocker P. R., Saven A., Paulson J. C. (2010). Blood.

[cit687] Bednar K. J., Nycholat C. M., Rao T. S., Paulson J. C., Fung-Leung W. P., Macauley M. S. (2019). ACS Chem. Biol..

[cit688] Spiller F., Nycholat C. M., Kikuchi C., Paulson J. C., Macauley M. S. (2018). J. Immunol..

[cit689] Pang L., Macauley M. S., Arlian B. M., Nycholat C. M., Paulson J. C. (2017). ChemBioChem.

[cit690] Bednar K. J., Shanina E., Ballet R., Connors E. P., Duan S., Juan J., Arlian B. M., Kulis M. D., Butcher E. C., Fung-Leung W. P., Rao T. S., Paulson J. C., Macauley M. S. (2017). J. Immunol..

[cit691] Srivastava A., Arlian B. M., Pang L., Kishimoto T. K., Paulson J. C. (2021). ACS Chem. Biol..

[cit692] Peng W., Paulson J. C. (2017). J. Am. Chem. Soc..

[cit693] Islam M., Arlian B. M., Pfrengle F., Duan S., Smith S. A., Paulson J. C. (2022). J. Am. Chem. Soc..

[cit694] Harumoto T., Iwai H., Tanigawa M., Kubo T., Atsumi T., Tsutsumi K., Takashima M., Destito G., Soloff R., Tomizuka K., Nycholat C., Paulson J., Uehara K. (2022). ACS Chem. Biol..

[cit695] Orgel K. A., Duan S., Wright B. L., Maleki S. J., Wolf J. C., Vickery B. P., Burks A. W., Paulson J. C., Kulis M. D., Macauley M. S. (2017). J. Allergy Clin. Immunol..

[cit696] Hong S., Yu C., Wang P., Shi Y., Cao W., Cheng B., Chapla D. G., Ma Y., Li J., Rodrigues E., Narimatsu Y., Yates, 3rd J. R., Chen X., Clausen H., Moremen K. W., Macauley M. S., Paulson J. C., Wu P. (2021). Angew. Chem., Int. Ed..

[cit697] Malissen B., Tamoutounour S., Henri S. (2014). Nat. Rev. Immunol..

[cit698] Wong E., Montoya B., Stotesbury C., Ferez M., Xu R. H., Sigal L. J. (2019). Cell Rep..

[cit699] de Witte L., Nabatov A., Pion M., Fluitsma D., de Jong M. A., de Gruijl T., Piguet V., van Kooyk Y., Geijtenbeek T. B. (2007). Nat. Med..

[cit700] van der Vlist M., de Witte L., de Vries R. D., Litjens M., de Jong M. A., Fluitsma D., de Swart R. L., Geijtenbeek T. B. (2011). Eur. J. Immunol..

[cit701] Ng W. C., Londrigan S. L., Nasr N., Cunningham A. L., Turville S., Brooks A. G., Reading P. C. (2015). J. Virol..

[cit702] de Jong M. A., Vriend L. E., Theelen B., Taylor M. E., Fluitsma D., Boekhout T., Geijtenbeek T. B. (2010). Mol. Immunol..

[cit703] Hunger R. E., Sieling P. A., Ochoa M. T., Sugaya M., Burdick A. E., Rea T. H., Brennan P. J., Belisle J. T., Blauvelt A., Porcelli S. A., Modlin R. L. (2004). J. Clin. Invest..

[cit704] McDermott R., Bausinger H., Fricker D., Spehner D., Proamer F., Lipsker D., Cazenave J. P., Goud B., De La Salle H., Salamero J., Hanau D. (2004). J. Invest. Dermatol..

[cit705] Idoyaga J., Lubkin A., Fiorese C., Lahoud M. H., Caminschi I., Huang Y., Rodriguez A., Clausen B. E., Park C. G., Trumpfheller C., Steinman R. M. (2011). Proc. Natl. Acad. Sci. U. S. A..

[cit706] Idoyaga J., Suda N., Suda K., Park C. G., Steinman R. M. (2009). Proc. Natl. Acad. Sci. U. S. A..

[cit707] Stoitzner P., Tripp C. H., Eberhart A., Price K. M., Jung J. Y., Bursch L., Ronchese F., Romani N. (2006). Proc. Natl. Acad. Sci. U. S. A..

[cit708] Kervevan J., Bouteau A., Lanza J. S., Hammoudi A., Zurawski S., Surenaud M., Dieudonne L., Bonnet M., Lefebvre C., Hocini H., Marlin R., Guguin A., Hersant B., Hermeziu O., Menu E., Lacabaratz C., Lelievre J. D., Zurawski G., Godot V., Henri S., Igyarto B. Z., Levy Y., Cardinaud S. (2021). PLoS Pathog.

[cit709] Tripp C. H., Voit H., An A., Seidl-Philipp M., Krapf J., Sigl S., Romani N., Del Frari B., Stoitzner P. (2021). Exp. Dermatol..

[cit710] Klechevsky E., Morita R., Liu M., Cao Y., Coquery S., Thompson-Snipes L., Briere F., Chaussabel D., Zurawski G., Palucka A. K., Reiter Y., Banchereau J., Ueno H. (2008). Immunity.

[cit711] Wamhoff E. C., Schulze J., Bellmann L., Rentzsch M., Bachem G., Fuchsberger F. F., Rademacher J., Hermann M., Del Frari B., van Dalen R., Hartmann D., van Sorge N. M., Seitz O., Stoitzner P., Rademacher C. (2019). ACS Cent. Sci..

[cit712] Bachem G., Wamhoff E. C., Silberreis K., Kim D., Baukmann H., Fuchsberger F., Dernedde J., Rademacher C., Seitz O. (2020). Angew. Chem., Int. Ed..

[cit713] Schulze J., Rentzsch M., Kim D., Bellmann L., Stoitzner P., Rademacher C. (2019). Biochemistry.

[cit714] Bellmann L., Zelle-Rieser C., Milne P., Resteu A., Tripp C. H., Hermann-Kleiter N., Zaderer V., Wilflingseder D., Hortnagl P., Theochari M., Schulze J., Rentzsch M., Del Frari B., Collin M., Rademacher C., Romani N., Stoitzner P. (2021). J. Invest. Dermatol..

[cit715] Rentzsch M., Wawrzinek R., Zelle-Rieser C., Strandt H., Bellmann L., Fuchsberger F. F., Schulze J., Busmann J., Rademacher J., Sigl S., Del Frari B., Stoitzner P., Rademacher C. (2021). Front. Immunol..

[cit716] van Kasteren S. I., Campbell S. J., Serres S., Anthony D. C., Sibson N. R., Davis B. G. (2009). Proc. Natl. Acad. Sci. U. S. A..

[cit717] Shchegravina E. S., Sachkova A. A., Usova S. D., Nyuchev A. V., Gracheva Y. A., Fedorov A. Y. (2021). Russ. J. Bioorg. Chem..

[cit718] Magnani J. L. , Peterson J. M., Fogler W. E. and Baek M.-G., WO2022061168A1, 2022

[cit719] Page M. G. P. (2013). Ann. NY Acad. Sci..

[cit720] Klahn P., Brönstrup M. (2017). Nat. Prod. Rep..

[cit721] Lehar S. M., Pillow T., Xu M., Staben L., Kajihara K. K., Vandlen R., DePalatis L., Raab H., Hazenbos W. L., Hiroshi Morisaki J., Kim J., Park S., Darwish M., Lee B.-C., Hernandez H., Loyet K. M., Lupardus P., Fong R., Yan D., Chalouni C., Luis E., Khalfin Y., Plise E., Cheong J., Lyssikatos J. P., Strandh M., Koefoed K., Andersen P. S., Flygare J. A., Wah Tan M., Brown E. J., Mariathasan S. (2015). Nature.

[cit722] Meiers J., Zahorska E., Röhrig T., Hauck D., Wagner S., Titz A. (2020). J. Med. Chem..

[cit723] Meiers J., Rox K., Titz A. (2022). J. Med. Chem..

[cit724] Metelkina O., Huck B., O'Connor J. S., Koch M., Manz A., Lehr C. M., Titz A. (2022). J. Mater. Chem. B.

